# Ortho‐Substituent Effects on Halogen Bond Geometry for N‐Haloimide⋯2‐Substituted Pyridine Complexes

**DOI:** 10.1002/advs.202307208

**Published:** 2023-12-07

**Authors:** Shilin Yu, J. Mikko Rautiainen, Parveen Kumar, Lorenzo Gentiluomo, Jas S. Ward, Kari Rissanen, Rakesh Puttreddy

**Affiliations:** ^1^ Department of Chemistry University of Jyvaskyla P.O. Box 35 Jyvaskyla 40014 Finland

**Keywords:** halogen bond, haloimide, ortho, saccharin, sigma hole

## Abstract

The nature of (imide)N–X⋯N(pyridine) halogen‐bonded complexes formed by six N‐haloimides and sixteen 2‐substituted pyridines are studied using X‐ray crystallography (68 crystal structures), Density Functional Theory (DFT) (86 complexation energies), and NMR spectroscopy (90 association constants). Strong halogen bond (XB) donors such as N‐iodosuccinimide form only 1:1 haloimide:pyridine crystalline complexes, but even stronger N‐iodosaccharin forms 1:1 haloimide:pyridine and three other distinct complexes. In 1:1 haloimide:pyridine crystalline complexes, the haloimide's N─X bond exhibits an unusual bond bending feature that is larger for stronger N‐haloimides. DFT complexation energies (Δ*E*_XB_) for iodoimide–pyridine complexes range from −44 to −99 kJ mol^−1^, while for N‐bromoimide–pyridine, they are between −31 and −77 kJ mol^−1^. The Δ*E*_XB_ of I⋯N XBs in 1:1 iodosaccharin:pyridine complexes are the largest of their kind, but they are substantially smaller than those in [*bis*(saccharinato)iodine(I)]pyridinium salts (−576 kJ mol^−1^), formed by N‐iodosaccharin and pyridines. The NMR association constants and Δ*E*_XB_ energies of 1:1 haloimide:pyridine complexes do not correlate as these complexes in solution are heavily influenced by secondary interactions, which DFT studies do not account for. Association constants follow the σ‐hole strengths of N‐haloimides, which agree with DFT and crystallography data. The haloimide:2‐(*N*,*N*‐dimethylamino)pyridine complex undergoes a halogenation reaction resulting in 5‐iodo‐2‐dimethylaminopyridine.

## Graphical abstract

Comprehensive analysis of N‐haloimides and 2‐substituted pyridine N−X···N halogen‐bonded complexes (68 crystal structures, 102 Density Functional Theory (DFT) optimized structures, and 90 ^1^H NMR association constants) reveals unusual packing forces induced N−X bond bendings and N···X···N motifs symmetrization in crystals, and DFT bond energies tunability on sigma‐hole strengths.

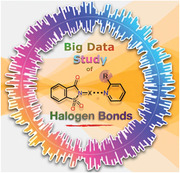

## 1 Introduction

Non‐covalent interactions (NCIs) are ubiquitous and pivotal in controlling the structural integrity, dynamics, stability, and properties of functional materials^[^ ^1^, ^2^ ^]^ as well as chemical^[^ ^3^ ^]^ and biological systems.^[^ ^4^ ^]^ Despite having lower strengths and being less directional than covalent bonds, NCIs have become increasingly valuable over time.^[^ ^5^ ^]^ A variety of NCIs are available for controlling molecules, which can be chosen based on their geometry and bonding properties. Among these, halogen bonding^[^ ^6^ ^]^ has received a lot of interest as an alternative to hydrogen bonding.^[^ ^7^ ^]^ Halogen bonding is an R─X⋯Y type attractive interaction, where X generally represents iodine or bromine, and Y can be any kind of Lewis base (e.g., N, O, S).^[^ ^6^ ^]^ This interaction is based on the occurrence of a σ‐hole, a region of lower electron density on the extension of an R─X bond, as a result of the anisotropic charge distribution around the X‐atom. It is demonstrated that the directionality of the halogen bond (XB) interaction is determined by the size of the σ‐hole.^[^ ^8^ ^]^ Clark and Heßelmann explained this preference by performing natural bond order analysis on alkyl halides and proposing an approximate *s* ^2^ *p*_x_^2^ *p*_y_^2^ *p*_z_^1^ configuration (where z is the direction of the R─X bond) for a head‐on interaction of a halogen's deficient electron density site or σ‐hole with nucleophiles.^[^ ^9^ ^]^

Over the past few decades, XB crystal engineering has focused on using a variety of XB donors to interact with N/O/S‐heterocycles, which has been extremely beneficial in the rational design of functional materials.^[^ ^10^, ^11^ ^]^ Previously studied XB complexes can be broadly divided into three classes: 1) R─X⋯Y, where the halogen is bound to a non‐fluorinated organic backbone, 2) R_F_─X⋯Y, where the halogen is bound to a fluorinated organic structure, and 3) Y⋯X⁺⋯Y complexes, where the halogen carrying a positive charge is trapped between two Lewis bases. The utility of these three classes is well‐known. For instance, class 1 neutral C─I···N halogen‐bonded systems have been used in the synthesis of phosphorescent materials,^[^ ^12^, ^13^ ^]^ the reversible nature and fluorine content of the class 2 C_F_─I···N XBs make them suitable for liquid crystals^[^ ^14^ ^]^ and functional materials,^[^ ^15^ ^]^ and class 3 [N···I···N]⁺ XBs are used in the preparation of supramolecular capsules,^[^ ^16^, ^17^, ^18^ ^]^ helicates,^[^ ^19^ ^]^ and porous structures^[^ ^20^ ^]^ as well as halogenating reagents due to the reactivity of iodine in them.^[^ ^21^ ^]^

Haloimides are a unique class of XB donors with N─X functionality situated between two electron withdrawing C═O or C═O and SO_2_ groups, and their complexes belong to class 1. A growing interest in haloimide complexes is fueled by the work of Fourmigué and co‐workers, who has shown that the very strongly polarized N─I bond of N‐iodosaccharin (NISac) will dissociate when combined with a highly nucleophilic 4‐(*N*,*N*‐dimethylamino)pyridine (DMAP), resulting in an iodopyridinium cation and an N‐saccharinate anion, that is, formation of a salt.^[^ ^22^ ^]^ In contrast, when mixed with a simple pyridine, the NISac produces a co‐crystal with a modestly polarized N─I bond. Since then, studies on the N─I bond lengthening and I⋯N bond shortening features in (imide)N─I⋯N complexes using different pyridines have gained a lot of attention. From the viewpoint of haloimides, only three XB complex types have been studied: N‐haloimide‐*p*‐substituted pyridines,^[^ ^23^ ^─^ ^25^ ^]^ *bis*(N‐imidato)halogen(I) cationic salts,^[^ ^26^ ^]^ and N‐haloimide pyridine N‐oxide complexes,^[^ ^27^ ^]^ all aiming to examine N─I and I⋯N lengthening and shortening features in (imide)N─I⋯N motifs.

**Figure 1:**
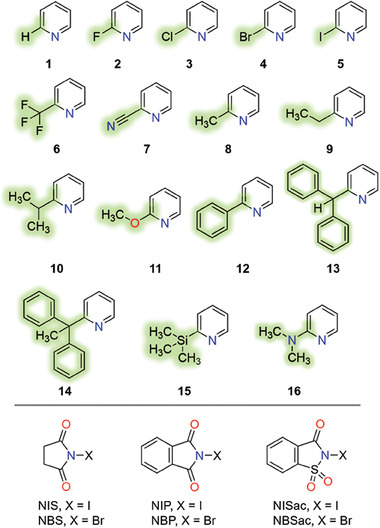
List of 2‐substituted pyridines (**1**–**16**) as XB acceptors and N‐halomides: N‐iodosuccinimide (NIS), N‐bromosuccinimide(NBS), N‐iodophthalimide (NIP), and N‐bromophthalimide (NBP), N‐iodosaccharin (NISac), and N‐bromosaccharin (NBSac) as XB donors.

Some of the most fundamental questions in this line of research were: when combined with *ortho*‐substituted pyridines, how does changing the identity of the X‐atom in the N─X group affect the XB strengths? What geometry variations would these donor–acceptor partners exhibit when made using analogous 2‐substituted pyridines? What is the sensitivity of the N─X bond with respect to the donor and acceptor? The answers to these questions may not be apparent from an evaluation of a limited number of XB complexes since some donor–acceptor partners may produce small changes to bond parameters while others may have significant influence. The systematic investigation of the XB parameters and structural changes of donors and acceptors in 96 complexes formed by two N‐halosuccinimides, two N‐halophthalimides, and two N‐halosaccharins against sixteen 2‐substituted pyridines are shown in **Figure** **1**. X‐ray crystallography, computational studies, and solution NMR are used to explain these findings.

## 2 Results and Discussion

**Figure 2:**
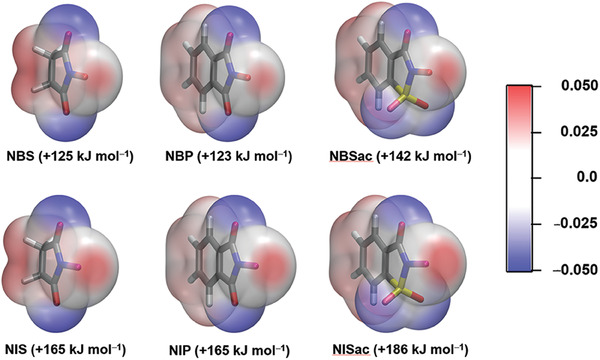
Computed electrostatic potential surfaces (ESP) at the PBE0‐D3/def2‐TZVP level of theory projected on the 0.001 au electron density surfaces of N‐haloimides with *V*_S,max_ values for NBS, NBP, NBSac (top: left‐to‐right), and NIS, NIP, and NISac (bottom: left‐to‐right).

As a preliminary approach to gauging the XB donor strengths of the N‐bonded halogens their σ‐hole sizes can be illustrated by plotting the molecular electrostatic potentials of N‐haloimides and calculating their *V*_s,max_ values. The comparison allows observation of the σ‐hole dependence on the halogen as well as the imide structure (**Figure** **2**). In general, iodines have larger σ‐hole size than bromines, and halosaccharins have the largest *V*_s,max_ values, decreasing in the order NISac > NIS ≥ NIP for N‐iodoimides and NBSac > NBS ≥ NBP for N‐bromoimides. Figure 2 provides some useful insights: i) although succinimides and phthalimides have very different electronic ring structures, there is no big difference in their σ‐hole sizes. Note that in their structures the N─X group is situated between the two C═O groups. ii) The *V*_s,max_ difference between NBP and NBSac is 14 kJmol^−1^ and between NIP and NISac is 21 kJmol^−1^. Both phthalimide and saccharin have aromatic rings, but the former has N─X group between C═O groups, and the latter between C═O and SO_2_ groups. These findings may suggest that the magnitude of *V*_s,max_ values are significantly influenced by the type of the groups present adjacent to the N─X group rather than the aromatic ring.

### 2.1 X‐Ray Crystallography

**Figure 3:**
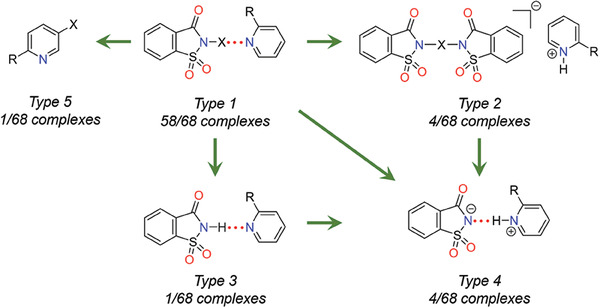
A summary of types of X‐ray crystal structures of halogen‐ and hydrogen‐bonded complexes presented using the saccharin donor.

Sixty‐eight crystal structures were crystallized from acetone using a 1:1 equivalent donor:acceptor ratio. Crystallization experiments resulted in five distinct types of complexes: the desired 1:1 halogen‐bonded (58 structures, type 1), [*bis*(N‐imidato)halogen(I)]pyridinium (4 structures, type 2), the neutral (1 structure, type 3) and salt (4 structures, type 4) hydrogen‐bonded co‐crystals, and a halogenated derivative (1 structure, type 5). NIS, NIP, NBS, and NBP only form type 1 complexes, while NISac and NBSac produce types 1–4. The four types of saccharin complexes can be used to provide a general explanation of how the type 1 parent complex transforms during the crystallization processes. Type 2 is the ligand exchange reaction of a pyridine with an in situ formed saccharinate anion; analogous type 2 complexes with different pyridinium cations have been reported in the literature.^[^ ^26^ ^]^ Type 3 is the consequence of iodine exchange with hydrogen, or proton abstraction by an in situ formed saccharinate anion that leads to the hydrogen‐bonded complex. Type 4 formation could be mediated by one or more pathways; for instance, one pathway is type 2 N–X bonds breaking to give saccharinate anions, and saccharinate then hydrogen bonding with the protonated pyridinic nitrogen.^[^ ^28^ ^]^ The second pathway is proton abstraction by pyridine from saccharin, that is, via type 3 to type 2. Note that i) types 1 and 3 are co‐crystals while types 2 and 4 are salts, and ii) only NISac produces type 2 structures, which can be attributed to a strong σ‐hole. However, considering that a type 3 with NIS has been reported,^[^ ^29^ ^]^ the large σ‐hole explanation appears less feasible, implying that the complexation outcome is influenced by packing and crystallization factors. Nevertheless, **Figure** **3** demonstrates the potential of haloimides and Lewis bases, when combined, to generate halogen(I) ions for halogenation in organic reactions.^[^ ^30^ ^]^ Combining NISac and 2‐(*N*,*N*‐dimethylamino)pyridine produced the type 5 crystal structure, and the results of these findings are discussed with the help of further solution NMR studies.

A detailed structure analysis for type 1 structures was carried out to investigate the bond parameters. All complexes have short X···N distances that are smaller than the sum of the X‐ and N‐atom van der Waals radii (Br + N = 3.40 Å, I + N = 3.53 Å), and **∠**N–X···N that range from 169° to 180° (see Tables S1–S6, Supporting Information). The examination of the asymmetric unit cells reveals that 51 out of 58 structures contain one 1:1 XB complex, while the others deviate from the 1:1 stoichiometry. The X···N distances in these seven additional structures are essentially identical, differing just by a maximum of ≈0.07 Å. For instance, NBS‐**1** asymmetric unit has four crystallographically different 1:1 adducts and their Br···N distances are 2.444(2), 2.499(2), 2.424(2), and 2.417(2) Å. NIS‐**11** is the only structure whose asymmetric unit contains a second NIS molecule participating in N–I···O = C (2.654 Å) halogen bonding with the carbonyl oxygen of the 1:1 XB complex (Figure S2, Supporting Information).

**Figure 4:**
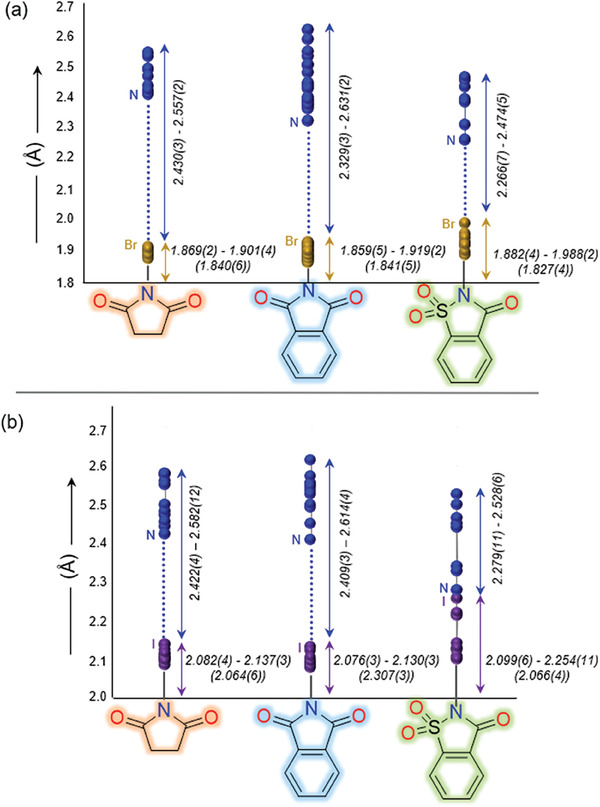
Comparison of N─X and X···N (X = Br, I) bond elongations and shortenings, a) in N‐bromoimide and b) N‐iodoimide complexes. Uncomplexed N‐haloimide N─X bond lengths are shown in parentheses (color code: Br, gold, I, purple, and N, blue dots). Note: The data in the figure corresponds to 58 crystal structures. The mean of N−X bond distances is 2.008 ± 0.003 Å, and X⋯N is 2.472 ± 0.003 Å.

**Figure** **4**a,b shows how halogen and nitrogen atoms are distributed within the XB (imide)N─X···N motifs. The imide N─X bond elongation defined as, Δ(N─X) = (N─X)_complex_ – (N─X)_ligand_, is in the range of 0.03–0.07 Å for NBS, 0.02–0.08 Å for NBP, 0.06–0.16 Å for NBSac, 0.02–0.07 Å for NIS, 0.04–0.09 Å for NIP, and 0.03–0.19 Å for NISac complexes (Tables S1–S6, Supporting Information). The Δ(N─X) of halogens are tightly clustered at a single location except for NISac. NISac‐**1** (0.188 Å) and NBSac‐**9** (0.161 Å) have the largest Δ(N─X) values among iodoimide‐ and bromoimide complexes, respectively. The N─X···N patterns show a broad dispersion of pyridine nitrogen distances. Pyridines with electron‐donating substituents (─CH_3_, ─Et) are typically found at one end of the distribution (near to halogen), whereas those with electron‐withdrawing substituents (─F, ─Cl, ─CF_3_) are found at the other end of the distribution (Figure 4).

For iodoimide complexes, I···N distances range from 2.279(11) to 2.614(4) Å, and for bromoimides, Br···N distances range from 2.266(2) to 2.631(2) Å. NISac‐**1** has the shortest I···N distance [2.279(2) Å], which is surprising given that nitrogen in simple pyridine is less nucleophilic than, for instance, 2‐ethylpyridine^[^ ^31^ ^]^ in NISac‐**9**, which has a longer I···N distance of 2.325(18) Å. Two different N‐bromoimide complexes were identified for NBSac‐**5**; the first has a Br···N distance of 2.392(2) Å and the second of 2.266(7) Å. The latter has the shortest Br···N distance of all the bromoimide complexes and is same as the NBSac‐**9**. The varied distances of NBSac‐**5**, as well as similar Br···N distances in NBSac‐**5** and −**9** despite their very different *ortho*‐substituents, can be attributed to the role of packing forces influencing the bond properties.

**Figure 5:**
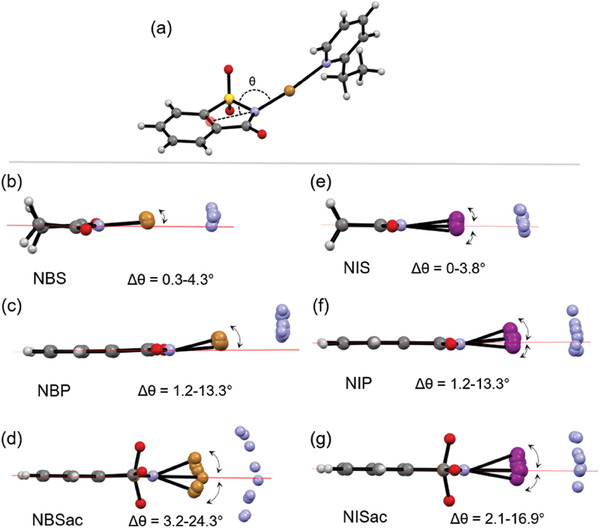
X‐ray crystal structural data. a) Defining the bending angle between the centroid of two carbons, nitrogen, and the bromine in NBSac‐**9**. Overlay structures displaying lΔ*θ*l (=180°– *θ*°_complex_) of (imide)N─X bonds in (b) NBS, (c) NBP, (d) NBSac, (e) NIS, (f) NIP, and (g) NISac XB complexes. Note that data in the figure correspond to 58 crystal structures. The mean of the bond bending angles is 6.6 ± 0.1°.

Some of the type 1 complexes exhibit notable N─X bond bendings. To examine this, bond bending angle (*θ*) is defined between the centroid of two carbons, imide nitrogen and the halogen as shown in **Figure** **5**a, and the absolute lΔ*θ*l = 180° – *θ*°_complex_ is used to explore N─X deviations from linearity or co‐planarity. The Δ*θ* are in the range of 0.3–4.3° for NBS, 1.2–13.3° for NBP, 3.2–24.3° for NBSac, 0–3.8° for NIS, 2.7–14.8° for NIP and 2.1–16.9° for NISac complexes. Note that the Δ*θ* values concomitantly increase as the σ‐hole strength of the XB donor increases (Figure 5b–g). These Δ*θ* values are larger than those reported for haloimide‐*meta*‐ and *para*‐substituted pyridine halogen‐bonded complexes that are in the range of 0.7–3.7° for NBS, 0–8.8.8° for NBP, 2.2° for NBSac, 6.7° for NIS, and 5.8–7.5° for NISac complexes (Table S8, Supporting Information). The C─H···O and C_π_···O interactions involving the oxygen of C═O and SO_2_, and π–π stacking between 1:1 donor:acceptor adducts are accountable for N─X bond bendings. Furthermore, packing analysis revealed that the C═O and SO_2_ oxygens exhibit multidentate C─H···O and C_π_···O contacts, which are abundant in succinimide complexes due to acidic ─CH_2_─ protons (for details, see Figure S3, Supporting Information). Contrarily, π–π contacts are abundant in phthalimide and saccharin complexes because of the π‐system. The π–π interactions in succinimide complexes are observed between the donors and acceptors, but in others, they are observed between the XB donors. Only in complexes of iodosuccinimide‐2‐chloro‐, bromo‐, and iodopyridines, C─Cl···O = C (3.073 Å), C─Br···O = C (3.025 Å), and C─l···O = C (3.011 Å) XBs between pyridinic *ortho*‐halogen and NIS carbonyl oxygen, and in iodosaccharin‐2‐iodopyridine, a C─l···OSO (2.972 Å) XB between the *ortho*‐iodine and SO_2_ oxygen, are observed. Note that C─X···O═C distances of succinimide complexes decrease as the electronegativity of the halogen decreases (Figure S4, Supporting Information).

If the N─X bendings are influenced by packing forces, then, N─X and X···N distances should not have a correlation. To verify this, the N─X distances are plotted against X···N distances of type 1 complexes. The N─X versus X···N correlations with a slope of −2.65 for Br···N and −1.76 for I···N halogen bonds has *R* ^2^ = 0.692 for the Br and *R* ^2^ = 0.853 for I‐complexes (Figure S5a,b, Supporting Information). The negative slope indicates an inverse relationship, viz. the N─X bond length increases while the X···N distance decreases. Evidently, iodoimide complexes are less affected by the non‐XB interactions compared to bromoimide. For the literature *meta*‐ and *para*‐substituted pyridines, the N─X versus X···N distances have a strong correlation with *R* ^2^ = 0.912 for Br‐ and 0.921 for I‐complexes (Figure S5c,d, Supporting Information). Overall, N─X bendings and N─X versus X···N correlations are more pronounced for *ortho*‐substituted pyridines than for *meta*‐ and *para*‐substituted pyridine complexes in the solid state.

**Figure 6:**
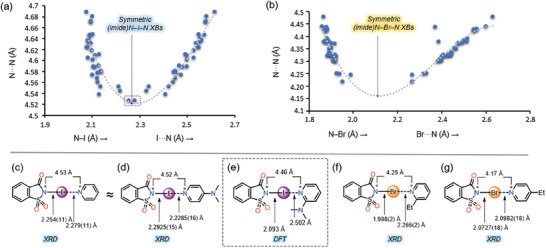
Correlation of (imide)N─I and I···N versus (imide)N···N(Py) (a), and (imide)N─Br and Br···N versus (imide)N···N(Py) (b). Comparison of symmetric (imide)N─I···N bonding situations in NISac‐**1** (c), NISac‐DMAP (d), and DFT optimized NISac‐**16** (e). Comparison of asymmetric (imide)N─Br···N bonding situations in NBSac‐**9** (f) and NISac‐4‐ethylpyridine (g). The symmetric bonds in figures (a) and (b) were determined by fitting the data to third‐order polynomial using the least squares method [for (imide)N─I─N, *y* = −2.3896*x* ^3^ + 18.704*x* ^2^ − 48.056*x* + 45.181, and for (imide)N─Br─N, *y* = −3.8841*x* ^3^ + 27.732*x* ^2^ − 65.192*x* + 54.739].

Steiner has examined the correlations between O─H···N and O···N distances in O─H···N hydrogen‐bonded systems.^[^ ^32^ ^]^ They found that when plotting O···N against O─H···N distances, a curved path was observed. The midpoint of this curve indicated symmetric O···H···N hydrogen‐bonded systems. In the correlation between O─H and H···O distances and O···N separations, the O─H bond lengthened as the H···N distance decreased. Eventually, a symmetric O···H···N geometry was achieved at an O···N separation of around ≈2.50 Å. At this point, the H‐atom was equidistant from the N and O atoms at ≈1.25 Å. The same approach was used to analyze the symmetry of N─X···N halogen‐bonded systems. Plots of N─X and X···N distances against N─X···N distances were constructed for iodoimide and bromoimide complexes. A parabolic curve was observed, and the minimum of this curve occurred when N─X = X···N (**Figure** **6**). For bromoimide complexes, the minimum was reached at a (imide)N···N distance of about ≈4.16 Å, while for iodoimide, it was approximately 4.52 Å. In the case of iodoimide systems, a near symmetric N···I···N bonding situation was achieved for NISac‐**1**, with a small difference of only 0.03 Å between N─I and I···N distances. The N─X···N bond parameters of NISac‐**1** are close to values reported for NISac‐DMAP, where the iodine has ′jumped′ toward the pyridinic nitrogen side. Note the significantly different N─I and I···N distances between reported NISac‐DMAP and DFT structure NISac‐**16** (Figure 6d,e). Bromoimide systems were unable to reach a symmetric N···Br···N bonding situation due to bromine's weak σ‐hole strength. Note the significant difference in N─Br and Br···N distances in NBSac‐ethylpyridine complexes, when the ethyl group is *ortho*‐ and *para*‐ to the pyridinic nitrogen (Figure 6f,g).

### 2.2 Computational Studies

Type 1 XB complexes were optimized using the PBE0‐D3/def2‐TZVP^[^ ^33^, ^34^, ^35^, ^36^, ^37^, ^38^, ^39^, ^40^ ^]^ method and Gaussian 16 program, which we have used in our previous halogen bonding studies.^[^ ^27^, ^41^, ^42^ ^]^ The gas‐phase optimized structures consistently have longer X···N distances than those in crystal structures. The optimized I···N distances for iodoimide complexes range from 2.419 to 2.694 Å (see Tables S1–S3, Supporting Information), while the Br···N distances for bromoimides range from 2.385 to 2.672 Å (see Tables S5–S7, Supporting Information). The NISac‐**15** (2.419 Å) has the shortest I···N distance, but it is crystallographically characterised as type 2. The next shortest I···N distance (2.447 Å) is calculated for NISac‐**1**, which agrees with the shortest experimental I···N distance (2.279(11) Å). The calculated shortest Br···N distance (2.385 Å) for NBSac‐**9** (2.266(2) Å) matches the experimental observation. The XB of NBSac‐**5**, which was short in the crystal structure, has been calculated to be 2.506 Å, which is the intermediate length of bromoimide complexes. To get optimized XB bond parameters that are closer to crystal structure bond parameters the structures can be optimized using polarized continuum model (PCM) and for example, CHCl_3_ as solvent (see Table S4, Supporting Information). However, the relative trends in the XB bond parameters remain essentially the same as in the gas phase optimizations. The gas phase optimized structures have smaller N─X bond elongations than crystal structures. The Δ(N─X) elongations of optimized structures are in the range of 0.03–0.05 Å for NBS, 0.02–0.05 Å for NBP, 0.04–0.08 Å for NBSac, 0.04–0.07 Å for NIS, 0.04–0.07 Å for NIP, and 0.06–0.11 Å for NISac complexes. The largest deviations between optimized and experimental elongations are observed for NISac‐**1** (0.188 Å experimental versus 0.094 Å optimized) and NBSac‐**7** (0.303 Å experimental versus 0.036 Å optimized).

The optimized structures do not exhibit large deviations of the N─X bond linearity. The Δ*θ* values for halosuccinimides and halophthalimides are all below 1.1°. For halosaccharins, the Δ*θ* values are slightly larger but remain below 3.0°, with an exception for NBSac‐**13** (7.3°) and NBSac‐**14** (5.1°), which contain the bulky substituents that can result in steric strain on the XB and subsequent bond bending. This additional evidence demonstrates that the large Δ*θ* values are caused by packing forces and secondary interactions to other XB complexes in the solid‐state crystals.

**Figure 7:**
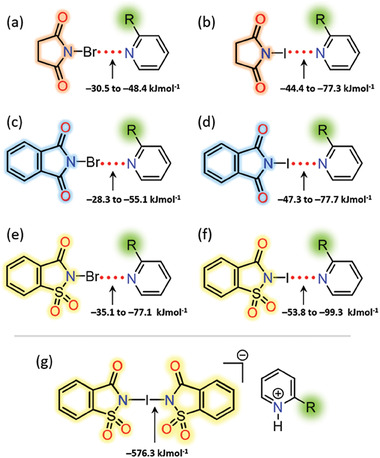
DFT optimized XB complexation energies (Δ*E*_XB_) data of (a,b) halosuccinimide‐pyridine, (c,d) halophthalimide‐pyridine, and (e,f) halosaccharin‐pyridine, and (g) [*bis*(N‐imidato)iodine(I)]pyridinium complexes.

The gas‐phase DFT XB complexation energies (Δ*E*_XB_) are summarized in **Figure** **7**. In general, for the same XB acceptor, the energy trend follows the σ‐hole strength, that is, halosaccharin >> halosuccinimide ≥ halophthalimide (Table S9, Supporting Information). Pyridines with electron withdrawing trifluoromethyl group have the smallest Δ*E*_XB_ values while those with bulky ─CH(Ph)_2_ and ─SiMe_3_ groups have the largest Δ*E*_XB_ values. Overall, the X···N energies are in the range of −28 kJ mol^−1^ to −99 kJ mol^−1^, and they are 8 kJ mol^−1^ to 39 kJ mol^−1^ larger than those values reported for halosuccinimide‐*para*‐substituted pyridines.^[^ ^24^ ^]^ The Δ*E*_XB_ energies of negatively charged [N─I─N]^−^ XB complexes [e.g., (NSac)_2_I‐**12H**] were computed in addition to 1:1 haloimide:pyridine neutral XB complexes. The [N─I─N]ˉ complexes have an overall complexation energy of −1152.6 kJ mol^−1^ or −576.3 kJ mol^−1^ per I─N halogen bond. Their optimized structures have I─N distances of 2.251 Å.

**Figure 8:**
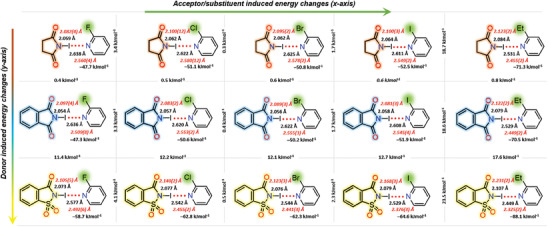
DFT optimized structural data of (top row) succinimide–pyridine, (middle row) phthalimide–pyridine, and (bottom row) saccharin–pyridine complexes. The bond parameters of crystal structures are shown in red italics for comparison.

DFT data of iodoimide complexes of type I crystal structures are compiled in **Figure** **8** to examine the influence of the imide scaffold and pyridinic substituents on I···N distances and Δ*E*_XB_ values. This analysis reveals interesting trends and insightful conclusions: i) in succinimide‐, phthalimide‐ and saccharin‐2‐halopyridine series, that is along the *x*‐axis, the overall Δ*E*_XB_ values follow the order F < Cl ≤ Br < I < Et. For iodoimide‐2‐halopyridines, the Δ*E*_XB_ differences from complex‐to‐complex are not greater than 4.1 kJ mol^−1^. The maximum Δ*E*_XB_ difference is observed between NISac‐**2** and NISac‐**3**. The Δ*E*_XB_ values of iodoimide‐2‐ethylpyridine are 15.3–18.4, 15.3–18.2, and 19.4–23 kJ mol^−1^ larger than their iodoimide‐2‐halopyridines. Smaller variations in Δ*E*_XB_ values between iodoimide‐2‐halopyridines can be related to the pyridinic nitrogen's weak nucleophilicity, which is caused by the electron‐withdrawing halogen substituents and the sudden “jump” in Δ*E*_XB_ values for iodoimide‐2‐ethylpyridine to the electron donating ethyl substituent. ii) Iodine's electron accepting capability is significantly impacted by the imide scaffold and Δ*E*_XB_ values follow the σ‐hole strength. The Δ*E*_XB_ differences between succinimide and phthalimide complexes are less than 0.6 kJ mol^−1^ along the *y*‐axis, but they are ≈11–18 kJ mol^−1^ between phthalimide and saccharin complexes. This comparison demonstrates that while tuning XBs is possible, tuning XBs with N‐haloimides is more reliable than tuning XBs with pyridines and is especially not effective with electron withdrawing groups.

### 2.3 Solution NMR Studies

The ^15^N NMR coordination shift, Δδ^15^N_coord_, is a useful tool for measuring the strengths of coordination^[^ ^43^ ^]^ and halogen‐bonded^[^ ^44^, ^45^, ^46^, ^47^ ^]^ complexes. It is defined as the difference between the δ^15^N chemical shift of a halogen‐bonded complex and that of its free ligand. A larger absolute coordination shift (∣Δδ^15^N_coord_∣) indicates a stronger interaction, and such characteristic shifts have previously been successfully implemented to discern structural information in similar halogen‐bonded systems.^[^ ^44^, ^45^, ^46^, ^47^ ^]^ In this study, we were able to successfully determine the Δδ^15^N_coord_ for NIS and NBS, but for other XB donors, it was not viable due to absence of protons at the 3‐position. Instead, the coordination shifts of the pyridinic nitrogen atoms were used to compare the strengths of X···N XBs, with a larger Δδ^15^N_coord_ value indicating a stronger X···N interaction. The Δδ^15^N_coord_ magnitudes of pyridinic nitrogens followed the halogen's σ‐hole strength order: NISac > NIS ≥ NIP for I···N halogen bonds and NBSac > NBS ≥ NBP. Note that despite the same σ‐hole strengths of N‐iodosuccinimide (165 kJ mol^−1^) and N‐iodophthalimide (165 kJ mol^−1^) donors, the Δδ^15^N_coord_ of pyridinic nitrogen atoms in iodosuccinimide complexes are significantly larger than iodophthalimide (Table S10, Supporting Information). For example, ∣Δδ^15^N_coord_∣ of NIS‐**1** is 41.5 ppm and that of NIP‐**1** is 0.7 ppm. The Δδ^15^N_coord_ values of bromosuccinimide and bromophthalimide complexes are smaller, ranging from 0.4 to 6 ppm and 0.1 to 4.5 ppm, respectively. Overall, the Δδ^15^N_coord_ values of iodoimide complexes are larger than bromoimide, which is consistent with the fact that as the halogen size decreases (I > Br > Cl > F), the XB donating properties decrease.

Association constants (*K*_XB_) are determined in CDCl_3_ from changes in haloimide proton resonances caused by the XB complexation. A 6–10 mm N‐haloimide solutions were titrated using ≈0.15 m pyridine stock solutions (for details, see Supporting Information). The *K*_XB_ values for a 1:1 donor:acceptor binding model were established using the online Bindfit program^[^ ^48^ ^]^ (Tables S11, Supporting Information). The *K*_XB_ values range from 4 to 3494 m ^−1^ for NIS, 7 to 2790 m ^−1^ for NIP, 236 to 144 459 m ^−1^ for NISac, and 1 to 394 m ^−1^ for NBSac. For NBS and NBP complexes *K*_XB_ values are small (≈1–5 m ^−1^) and are within in the fitting errors due to weak binding (for details, see Table S11, Supporting Information). The concentration of pyridines has an impact on *K*_XB_ values. For instance, *K*_XB_ values of NBS**‐8**, NBP‐**8**, and NBSac‐**8** titrated by using ≈0.15 m 2‐methylpyridine (**8**) solutions are 5, 4, and 332 m ^−1^, respectively, while those titrated with 1 m 2‐methylpyridine (**8**) solutions are 21, 19, and 423 m ^−1^, respectively. Estimation of *K*_XB_ values for haloimide‐2‐dimethylaminopyridine complexes were unsuccessful due to signal broadening. Single crystals formed from the NISac‐**16** titration sample were characterized by X‐ray diffraction to be a halogenated product, 5‐iodo‐2‐dimethylaminopyridine (**16**‐**I**). Note that in their crystallization studies, Fourmigué and co‐workers also obtained protonated 3‐bromo‐4‐dimethylaminopyridine and saccharinate as co‐crystals by mixing DMAP and NBSac.^[^ ^25^ ^]^

**Figure 9:**
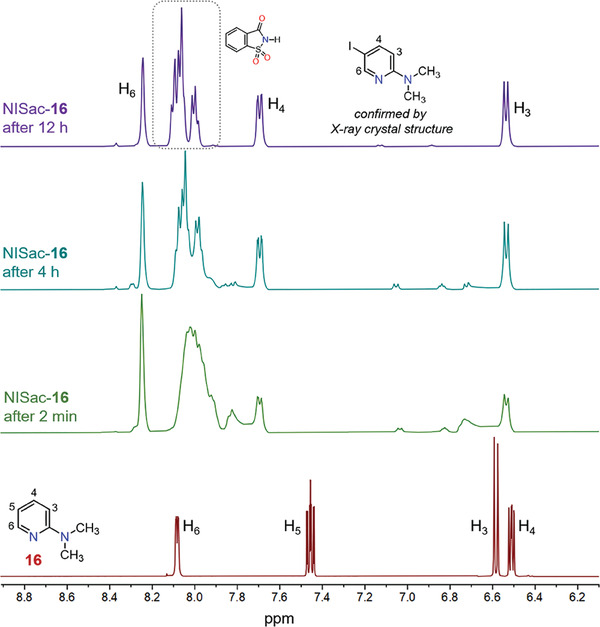
^1^H NMR stack spectra of **16** and NISac‐**16** in acetone‐d_6_ (298 K, 500 MHz).

A 1:1 equivalent of NISac‐**16** was monitored by ^1^H and ^1^H─^15^N HMBC NMR spectroscopy at 298 K conditions. The halogenated product begins to form as soon as the donor and acceptor components are mixed, as seen in **Figure** **9**. The presence of broad ^1^H NMR signals in the initial spectra indicates either the coexistence of multiple complexes or a rapid exchange of complexes on the NMR time scale. The ^1^H NMR signals related to 5‐iodo‐2‐dimethylaminopyridine and saccharin are separated after ≈12 h. The δ^15^N values of pyridinic and ‐NMe_2_ nitrogen in **16** and the halogenated product are −110 and −319 ppm and −105 and −315 ppm, respectively (Figure S102, Supporting Information).

The calculated Δ*E*_XB_ and experimental association constants logarithmic *K*_XB_ values of NIS, NIP, NISac, and NBSac complexes were examined for correlation (Figure S5a, Supporting Information). While the correlation exhibits a rough trend, the data points are significantly scattered around the trendline resulting in a weak linear correlation (*R* ^2^ = 0.760). The strongest measured association constants, such as NISac‐**5** (log *K*_XB_ measured 4.65 versus predicted 2.5) and NISac‐**12** (log *K*_XB_ measured 5.10 versus predicted 3.5), are those with the largest deviations from the overall trend. Since the calculated Δ*E*_XB_ show largest deviations for the NISac‐**Z** series, the complexation was also modelled by calculating free energies of complexation, $\Delta G_{\mathrm{XB}}^{\mathrm{PCM}}$ in chloroform solution using the Polarizable Continuum Model (PCM) method (Table S4, Supporting Information). However, the comparison of log *K*_XB_ and $\Delta G_{\mathrm{XB}}^{\mathrm{PCM}}$ (Figure S5b, Supporting Information) did not show any correlation (*R* ^2^ = 0.189). The deviations indicate that the simple computational 1:1 model is unable to accurately describe the complex binding situations that arise in solution.

**Figure 10:**
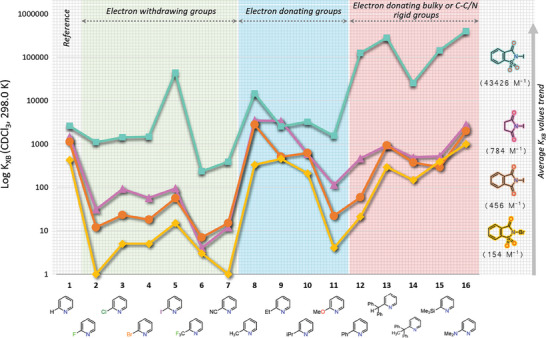
Chart displaying *K*_XB_ values trend of N‐iodosaccharin‐ (cyan), N‐iodosuccinimide‐ (fuchia‐violet), N‐iodophthalimide‐ (orange) and N‐bromosaccharin‐ (marigold) complexes. Determinations of the *K*_XB_ values of the haloimide‐2‐(N,N‐dimethylamino)pyridine complexes were unsuccessful due to ^1^H NMR signal broadening and arbitrary values are included in the chart for comparison purposes. Their *K*_XB_ values are not included in the average. Note: There are 60 binding constants in the figure. Their mean value is 11 205 m ^−1^. Note that the standard deviations (SDs) of several NISac complex association constants exceed their association values due to large fitting errors, as a result SD values are not depicted in the figure. See Table S11 (Supporting Information) for fitting errors.

The logarithmic *K*_XB_ values of NIS, NIP, NISac, and NBSac complexes were plotted in the stack mode as shown in **Figure** **10** and their average *K*_XB_ values follow the computed XB donor's σ‐hole strength. Note that the *K*_XB_ values of haloimide‐2‐dimethylaminopyridine complexes are not included in the average, and their dummy data points in the chart are included for reference. Large *K*_XB_ values of iodosaccharin complexes clearly suggest that it's iodine has a stronger electron accepting power among the iodoimide donors. The distribution of *K*_XB_ values for NBSac, NIP, and NIS complexes is steady, whereas the pattern for NISac complexes exhibits abrupt changes, which could be combinedly attributed to the strong XB complexation ability of NISac and secondary interactions. Within in the electron withdrawing groups panel, *K*_XB_ values of pyridines with electron withdrawing groups are small with an exception for 2‐iodopyridine.

## 3 Conclusion

In conclusion, the N─X⋯N (X = I, Br) halogen‐bonded complexes formed by three N‐bromoimide and three N‐iodoimide halogen bond (XB) donors and sixteen 2‐susbstituted pyridines were investigated through experimental and DFT studies. The large data set (68 crystal structures, 86 gas‐phase DFT optimized structures, and 90 NMR association constants) was used to investigate XB properties methodically based on XB donors and acceptors. These donor–acceptor partners produced essentially three types of crystalline complexes: fifty‐eight 1:1 haloimide:pyridine, four [*bis*(N‐imidato)iodine(I)]pyridinium and five hydrogen‐bonded complexes. In the solid‐state structures, the I⋯N distances of 1:1 N‐iodoimide‐pyridine complexes varied from 2.279(11) to 2.614(4) Å, whereas Br⋯N distances of N‐bromoimide‐pyridine complexes ranged from 2.266(2) to 2.631(2) Å. The 1:1 haloimide:pyridine halogen‐bonded complexes form an intricate network of secondary interactions such as hydrogen bonds and π–π contacts, inducing (imide)N−X bond bending. The N─X distances plotted against X···N distances of 1:1 haloimide:*ortho*‐substituted pyridine complexes show lower correlation values than the literature *meta*‐ and *para*‐substituted pyridine complexes, indicating the XB parameters in the former are more affected by packing forces than the latter. Packing analysis revealed that the C═O and SO_2_ oxygen exhibits multidentate C─H···O and C_π_···O contacts, which are prevalent in succinimide complexes due to acidic ─CH_2_─ protons, and π─π contacts in phthalimide and saccharin complexes due to their π‐system.

DFT XB interaction energies for N‐iodoimide‐pyridine complexes range from −44 to −99 kJ mol^−1^, while for N‐bromoimide‐pyridine complexes, they are between −31 and −77 kJ mol^−1^. These bond energies are significantly smaller than N─I bond energies calculated for [N─I─N]ˉ XBs (−576.3 kJ mol^−1^) in [*bis*(N‐imidato)halogen(I)]pyridinium complexes. The Δ*E*_DFT_ energies of 1:1 haloimide:pyridine complexes follow the order: N‐iodosaccharin > N‐iodosuccinimide ≧ N‐iodophthalimide > N‐bromosaccharin > N‐bromosuccinimide ≧ N‐bromophthalimide. DFT analysis revealed that while tuning of N−X···N XBs is possible, tuning XBs with N‐haloimides is more effective than tuning XBs with respect to substituents of pyridines and is notably ineffective with electron withdrawing groups. This discovery suggests that the halogen sigma‐hole on N‐haloimides governs the tunability and overrides the weaker electronic properties of pyridinic substituents. Even though the N−X⋯N monodentate halogen bond is the potent and major non‐covalent interaction, secondary bonding characteristics of XB donor structures, as revealed by crystal structures, and solvation may be even higher in solution which could explain the lack of correlation between DFT energies and solution data. The trends of NMR association constants have been calculated independently for each of the N‐haloimide‐PyNO and their average association constants follow the σ‐hole strengths of XB donors, which is in agreement with DFT and solid‐state X‐ray crystallography data.

## 4 Experimental Section

### Crystallography Data

Deposition Numbers 2297265–2297275 (for NIS series), 2297327–2297337 (for NIP series), 2297287–2297294 (for NISac series), 2297296–2297301 (for NBS series), 2297306–2297318 (for NBP series), 2297338–2297346 (for NBSac series), 2297348 (for (NSac)_2_I‐12H), 2297349 (for (NSac)_2_I‐**15**H), 2297350 (for (NSac)_2_I‐**15**Ha), 2297351 (for (NSac)_2_I‐10H), 2297352 (for NHSac‐**5**), 2297353 (for NSac‐**1**H), 2297354 (for NSac‐**16**Ha), 2297355 (for NSac‐**8**H), 2297356 (for NSac‐**16**H), and 2297358 (for **16‐I**) contain the supplementary crystallography data for this paper. These data are provided free of charge by the joint Cambridge Crystallographic Data Centre and Fachinformationszentrum Karlsruhe Access Structures service.

### Statistical Analysis

Olex 2^[^ ^49^ ^]^ and Mercury^[^ ^50^ ^]^ were employed for data extraction of XB parameters from 68 X‐ray crystal structures. Microsoft Excel was used for the correlation analysis of Figures 6 and 10. The means of N−X bond distances are 2.008 ± 0.003 Å, X⋯N are 2.472 ± 0.003 Å, and bond bending angles are 6.6 ± 0.1°. There are 102 DFT energies (86 gas‐phase and 16 with solvent model). The DFT linear correlation tests have been carried out by least squares fits using the OriginPro 2017 program. There are 90 binding constants in Figure 10. At least 20 ^1^H NMR experiments were performed to estimate the 1:1 binding model of each halogen‐bonded complex. Titration data was fitted into a 1:1 binding model using the Nelder‐Mead (Simplex) method with the “subtract initial values” option ticked available in the online Bindfit software.^[^ ^48^ ^]^ We employed a minimum of 40 data points of phthalimide and saccharin complexes, and a minimum of 20 data points of succinimide complexes to determine whether or not the binding model is 1:1 donor:acceptor. The mean of association constant values is 11 205 M^−1^ (for SDs, see Table S11, Supporting Information). The outliers are not excluded from the analysis because they are a part of the study.

## Conflict of Interest

The authors declare no conflict of interest.

## Supporting information

Supporting Information

## Data Availability

The data that support the findings of this study are available from the corresponding author upon reasonable request.
