# Supplementary material for: Ortho‐Substituent Effects on Halogen Bond Geometry for N‐Haloimide⋯2‐Substituted Pyridine Complexes
Source: Adv Sci (Weinh). 2023 Dec 7;11(6):2307208. doi: 10.1002/advs.202307208 (PMC10853718; doi:10.1002/advs.202307208)
Supplement: Supplementary file 1 — Supporting Information [file ADVS-11-2307208-s001.pdf]

ADVANCED SCIENCE
Open Access

Supporting Information

for *Adv. Sci.*, DOI 10.1002/advs.202307208

Ortho-Substituent Effects on Halogen Bond Geometry for N-Haloimide⋯2-Substituted Pyridine Complexes

Shilin Yu, J. Mikko Rautiainen, Parveen Kumar, Lorenzo Gentiluomo, Jas S. Ward, Kari Rissanen and Rakesh Puttreddy**

Supporting Information

Ortho-substituent Effects on Halogen Bond Geometry for N-Haloimide···2-Substituted pyridine Complexes

Shilin Yu, J. Mikko Rautiainen, Parveen Kumar, Lorenzo Gentiluomo, Jas S. Ward, Kari Rissanen, Rakesh Puttreddy

Department of Chemistry, University of Jyväskylä, Jyväskylä, Finland, FI-40014.

Table of Contents

1. General information *Page 2*

2. X-ray Crystallography *Page 3*

3. Density Functional Theory Studies *Page 22*

4. Solution NMR *Page 24*

5. References *Page 95*

1. General information

Pyridine (>99%) was purchased from VWR International GmbH. 2-Ethylpyridine (>98%), 2-phenylpyridine (>98%), 2-(dimethylamine)pyridine (>98%), N-iodosuccinimide (>99%), N-iodophthalimide (>98%), N-iodosaccharin (>98%), N-bromophthalimide (>95%), N-bromosaccharin (>98%) were purchased from TCI. 2-Fluoropyridine (98%), 2-cyanopyridine (99%), 2-chloropyridine (99%), 2-bromopyridine (99%), 2-methylpyridine (98%), 2-trimethylsilylpyridine (>98%), N-bromosuccinimide (>99%) and acetone (>99.9%) were purchased from sigma, whereas 2-methoxypyridine (98%) from Acros, 2-isopropylpyridine (95%) and 2-iodopyridine (97%) from Fluorochem, and 2-trifluoromethylpyridine (98%) from abcr chemicals. 2-Diphenylmethylpyridine and 2-(1,1-diphenylethyl)pyridine are synthesised following the literature. All reagents and solvents for synthesis were purchased from commercial sources and used without further purification. $CDCl_3$ (99.80% D, <0.01% water) was purchased from Eurisotop.

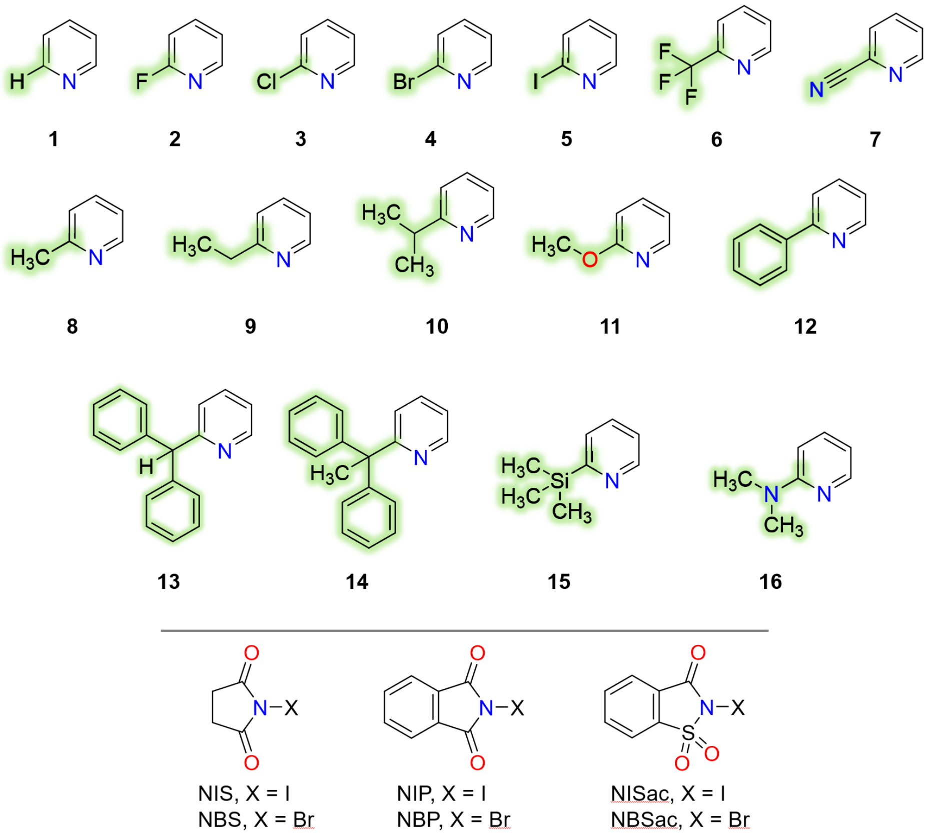

Figure S1. List of 2-substituted pyridines (**1** - **16**) as XB acceptors and N-halomides: N-iodosuccinimide (NIS), N-bromosuccinimide (NBS), N-iodophthalimide (NIP), and N-bromophthalimide (NBP), N-iodosaccharin (NISac), and N-bromosaccharin (NBSac) as XB donors.

2. X-Ray crystallography

The single-crystal X-ray data for NIS-**1**, NIS-**2**, NIS-**5**, NIS-**8**, NISac-**6**, NISac-**11**, NBS-**1**, NBS-**4**, NBS-**8**, NBS-**9**, NBP-**3**, NBP-**4**, NBP-**8**, NBP-**12**, NBP-**13**, NBSac-**2**, NBSac-**4**, NBSac-**5**, NBSac-**5**a, $(NISac)_2$I-**12**H, $(NISac)_2$I-**15**H, $(NISac)_2$I-**15**Ha, NHSac-**5**, NSac-**1**H, NSac-**16**H, and NSac-**16**Ha were measured at 170K on a Bruker-Nonius Kappa CCD diffractometer equipped with an APEX-II CCD detector using graphite-monochromated Mo-Kα (λ = 0.71073 Å) radiation. Single-crystal X-ray data for NIS-**3,** NIS-**4,** NIS-**6**, NIS-**9**, NIS-**11**, NISac-**3**, NBS-**3**, NBP-**6**, NBP-**7**, NBSac-**3,** and $(NISac)_2$I-**10**H were measured either 120K or 170K on a Rigaku SuperNova Single source diffractometer equipped with an Eos CCD detector using mirror-monochromated Mo-*K*α (*λ* = 0.71073 Å) radiation. The X-ray crystal structure data for NIS-**12**, NIS-**13**, NIP-**1**, NIP-**2**, NIP-**3**, NIP-**5,** NIP-**6,** NIP-**12**, NIP-**13**, NISac-**2**, NISac-**4**, NBP-**1**, NBP-**2**, NBP-**5**, NBP-**9**, NBP-**10**, NBP-**11**, NBSac-**6**, NBSac-**13**, and NBSac-**14** were collected at 120 K, using an Rigaku SuperNova four-circle diffractometer equipped with an Hybrid Pixel Array Detector (detector type: HyPix-Arc 100) and Cu-K_α (λ = 1.54184 Å) radiation. Whereas the data for NIP-**4**, NIP-**8**, NIP-**9**, NIP-**14**, NISac-**5,** NISac-**7,** NISac-**9,** NBS-**5**, NBSac-**9**, and NSac-**8**H were collected at 120 K using an XtaLAB Synergy R four-circle diffractometer equipped with a Hybrid Pixel Array Detector (detector type: HyPix-Arc 100). Diffraction type is PhotonJet R (Cu, λ = 1.54184 Å) X-ray source. CrysAlisPro was used for the data collection and reduction, and the intensities were absorption corrected using a gaussian face index absorption correction method for the data collection carried out using Rigaku diffractometers. For the data obtained using Bruker Nonius Kappa diffractometer, the processing was performed using the program COLLECT and HKL DENZO AND SCALEPACK.[6] The structure was solved with direct methods (SHELXS)[7,8] and refined by full-matrix least squares on F^2 using the *OLEX2* software[9], which utilizes the SHELXL-2015 module.[7,8]All structures were solved by intrinsic phasing (SHELXT)[2] and refined by full-matrix least squares on F^2 using the OLEX2[3], utilizing the SHELXL-2015 module.[4,5] Anisotropic displacement parameters were assigned to non-H atoms and isotropic displacement parameters for all H atoms were constrained to multiples of the equivalent displacement parameters of their parent atoms with U_{iso}(H) = 1.2 U_{eq}(parent atom). Single crystal data and CCDC numbers of all new structures are included below. These data can be obtained free of charge via http://www.ccdc.cam.ac.uk/conts/retrieving.html (or from the CCDC, 12 Union Road, Cambridge CB2 1EZ, UK; Fax: +44 1223 336033; E-mail: deposit@ccdc.cam.ac.uk).

1) Crystal data for NIS-**1** [CCDC-2297270]: Sum formula: $C_9H_9IN_2O_2$, M = 304.08, colourless block, 0.28 x 0.26 x 0.26 mm^3, orthorhombic, space group C*cce*, a = 7.6331(15) Å, b = 20.056(4) Å, c = 27.136(5) Å, α = 90°, β = 90°, γ = 90°, V = 4154.2(14) Å^3, Z = 16, Dc = 1.945 g/cm^3, F000 = 2336, μ= 3.059 mm^{-1}, T = 170 K, θ_{max} = 25.249°, 13835 total reflections, 1738 with Io > 2σ(Io), R_{int} = 0.0304, 1874 data, 131 parameters, 0 restraints, GooF = 1.217, R = 0.0209 and wR= 0.0414 [Io > 2σ(Io)], R = 0.0243 and wR= 0.0424 (all reflections), 0.267<dΔρ< -0.292 e/Å^3.

2) Crystal data for NIS-**2** [CCDC-2297268]: Sum formula: $C_9H_8FIN_2O_2$, M = 322.07, colourless block, 0.17 x 0.12 x 0.07 mm^3, orthorhombic, space group P$2_12_12_1$, a = 5.9336(12) Å, b = 8.8973(18) Å, c = 20.295(4) Å, α = 90°, β = 90°, γ = 90°, V = 1071.4(4) Å^3, Z = 4, Dc = 1.997 g/cm^3, F000 = 616, μ= 2.984 mm^{-1}, T = 170 K, θ_{max} = 25.242°, 5237 total reflections, 1794 with Io > 2σ(Io), R_{int} = 0.0371, 1934 data, 136 parameters, 0 restraints, GooF = 1.014, R = 0.0261 and wR= 0.0440 [Io > 2σ(Io)], R = 0.0298 and wR= 0.0448 (all reflections), 0.356<dΔρ< -0.283e/Å^3.

3) Crystal data for NIS-**3** [CCDC-2297274]: Sum formula: $C_9H_8ClIN_2O_2$, M = 338.52, colourless block, 0.103 x 0.05 x 0.034 mm^3, orthorhombic, space group P*bca*, a = 10.9926(11) Å, b = 7.6239(9) Å, c = 25.772(4) Å, α = 90°, β = 90°, γ = 90°, V = 2159.9(5) Å^3, Z = 8, Dc = 2.082 g/cm^3, F000 = 1296, μ= 3.193 mm^{-1}, T = 120 K, θ_{max} = 25.246°, 13851 total reflections, 1675 with Io > 2σ(Io), R_{int} = 0.0896, 1955 data, 124 parameters, 12 restraints, GooF = 1.263, R = 0.0971 and wR= 0.2105 [Io > 2σ(Io)], R = 0.1056 and wR= 0.2145 (all reflections), 3.029<dΔρ< -3.733e/Å^3.

4) Crystal data for NIS-**4** [CCDC-2297275]: Sum formula: $C_9H_8BrIN_2O_2$, M = 382.98, colourless block, 0.218 x 0.113 x 0.031 mm^3, orthorhombic, space group P*bca*, a = 11.0084(2) Å, b = 7.73508(14) Å, c = 25.9301(5) Å, α = 90°, β = 90°, γ = 90°, V = 2207.96(7) Å^3, Z = 8, Dc = 2.304 g/cm^3, F000 = 1440, μ= 6.501 mm^{-1}, T = 120 K, θ_{max} = 25.246°, 11331 total reflections, 1776 with Io > 2σ(Io), R_{int} = 0.0389, 1999 data, 136 parameters, 0 restraints, GooF = 0.0193, R = 0.0971 and wR= 0.0408 [Io > 2σ(Io)], R = 0.0245 and wR= 0.0434 (all reflections), 0.389 <dΔρ< -0.439 e/Å^3.

5) Crystal data for NIS-**5** [CCDC-2297273]: Sum formula: $C_9H_8I_2N_2O_2$, M = 429.97, colourless block, 0.24 x 0.17 x 0.16 mm^3, orthorhombic, space group P*bca*, a = 11.095(2) Å, b = 8.1428(16) Å, c = 26.146(5) Å, α = 90°, β = 90°, γ = 90°, V = 2362.3(8) Å^3, Z = 8, Dc = 2.418g/cm^3, F000 = 1584, μ= 5.308 mm^{-1}, T = 120 K, θ_{max} = 25.248°, 19077 total reflections, 1978 with Io > 2σ(Io), R_{int} = 0.0461, 2140 data, 136 parameters, 0 restraints, GooF = 1.087, R = 0.0195 and wR= 0.0398 [Io > 2σ(Io)], R = 0.0227 and wR= 0.0410 (all reflections), 0.332<dΔρ< -0.440 e/Å^3.

6) Crystal data for NIS-**6** [CCDC- 2297267]: Sum formula: $C_{10}H_8F_3IN_2O_2$, M = 372.08, colourless block, 0.126x 0.084x0.064 mm^3, triclinic, space group P-1, a = 7.6569(5) Å, b = 8.5366(5) Å, c = 10.7553(5) Å, α = 70.796(4)°, β = 83.671(4)°, γ = 64.566(6)°, V = 599.15(6) Å^3, Z = 2, Dc = 2.062 g/cm^3, F000 = 356, μ= 2.707mm^{-1}, T = 120 K, θ_{max} = 29.032°, 10509 total reflections, 2535 with Io > 2σ(Io), R_{int} = 0.0466, 2852 data, 163 parameters, 0 restraints, GooF = 1.074, R = 0.0320 and wR= 0.0701 [Io > 2σ(Io)], R = 0.0395 and wR= 0.0750 (all reflections), 1.231 <dΔρ< -0.756 e/Å^3.

7) Crystal data for NIS-**8** [CCDC-2297269]: Sum formula: $C_{10}H_{11}IN_2O_2$, M = 318.11, colourless block, 0.28 x 0.17 x 0.11 mm^3, orthorhombic, space group P$2_12_12_1$, a = 8.7985(18) Å, b = 6.2308(12) Å, c = 20.982(4) Å, α = 90°, β = 90°, γ = 90°, V = 1150.3(4)Å^3, Z = 4, Dc = 1.837 g/cm^3, F000 = 616, μ= 2.767 mm^{-1}, T = 170 K, θ_{max} = 25.250°, 23483 total reflections, 2059 with Io > 2σ(Io), R_{int} = 0.0466, 2092 data, 137 parameters, 0 restraints, GooF = 1.053, R = 0.0201 and wR= 0.0492 [Io > 2σ(Io)], R = 0.0206 and wR= 0.0494 (all reflections), 0.657<dΔρ<-0.350 e/Å^3.

8) Crystal data for NIS-**9** [CCDC-2297265]: Sum formula: $C_{11}H_{13}IN_2O_2$, M = 332.13, colourless block, 0.171 x 0.107 x 0.064 mm^3, tetragonal, space group I4_1/a, a = 20.4816(3) Å, b = 20.4816(3) Å, c = 11.6092(3) Å, α = 90°, β = 90°, γ = 90°, V = 4870.01(19) Å^3, Z = 16, Dc = 1.812 g/cm^3, F000 = 2592, μ= 2.618 mm^{-1}, T = 170 K, θ_{max} = 25.243°, 8040 total reflections, 1854 with Io > 2σ(Io), R_{int} = 0.0337, 2199 data, 146 parameters, 0 restraints, GooF = 1.047, R = 0.0214 and wR= 0.0428 [Io > 2σ(Io)], R = 0.0287 and wR= 0.0461 (all reflections), 0.361 <dΔρ<-0.404 e/Å^3.

9) Crystal data for NIS-**11** [CCDC-2297272]: Sum formula: $C_{14}H_{15}I_2N_3O_5$, M = 559.09, colourless block, 0.139 x 0.094 x 0.078 mm^3, monoclinic, space group P2_1/c, a = 7.620(3) Å, b = 32.311(4) Å, c = 7.808(2) Å, α = 90°, β = 116.24(4)°, γ = 90°, V = 1724.2(9) Å^3, Z = 4, Dc = 2.154 g/cm^3, F000 = 1064, μ= 3.677 mm^{-1}, T = 170 K, θ_{max} = 25.249°, 9717 total reflections, 2746 with Io > 2σ(Io), R_{int} = 0.0386, 3120 data, 218 parameters, 0 restraints, GooF = 1.046, R = 0.0306 and wR= 0.0660 [Io > 2σ(Io)], R = 0.0371 and wR= 0.0700 (all reflections), 1.551<dΔρ<-0.907 e/Å^3.

10) Crystal data for NIS-**12** [CCDC-2297271]: Sum formula: $C_{15}H_{13}IN_2O_2$, M = 380.17, colourless block, 0.08 x 0.05 x 0.039 mm^3, monoclinic, space group P2_1/n, a = 8.37100(10) Å, b = 14.43480(10) Å, c = 11.62240(10) Å, α = 90°, β = 91.1510(10)°, γ = 90°, V = 1404.09(2) Å^3, Z = 4, Dc = 1.798 g/cm^3,

F000 = 744, μ= 17.951 mm^{-1}, T = 120 K, θ_{max} = 79.848°, 18458 total reflections, 2882 with Io > 2σ(Io), R_{int} = 0.0275, 3007 data, 181 parameters, 0 restraints, GooF = 1.107, R = 0.0217 and wR= 0.0587 [Io > 2σ(Io)], R = 0.0226 and wR= 0.0594 (all reflections), 0.378<dΔρ<-0.796 e/$Å^3$.

11) Crystal data for NIS-**13** [CCDC-2297266]: Sum formula: $C_{22}H_{19}IN_2O_2$, M = 470.29, colourless block, 0.14 x 0.13 x 0.12 mm^3, monoclinic, space group $P2_1/n$, a = 9.3071(19) Å, b = 14.349(3) Å, c = 14.998(3) Å, α = 90°, β = 104.76(3)°, γ = 90°, V = 1936.8(7) $Å^3$, Z = 4, Dc = 1.613 g/cm^3, F000 = 744, μ= 1.673 mm^{-1}, T = 170 K, θ_{max} = 25.248°, 12131 total reflections, 1921 with Io > 2σ(Io), R_{int} = 0.1646, 3498 data, 238 parameters, 0 restraints, GooF = 1.032, R = 0.0764 and wR= 0.1222 [Io > 2σ(Io)], R = 0.1637 and wR= 0.1479 (all reflections), 1.061<dΔρ<-0.844 e/$Å^3$.

12) Crystal data for NIP-**1** [CCDC-2297333]: Sum formula: $C_{13}H_9IN_2O_2$, M = 352.12, colourless block, 0.08x0.05x0.04mm^3, monoclinic, space group $P2_1/n$, a = 15.5195(3)Å, b = 4.89490(10)Å, c = 16.2965(3)Å, α = 90°, β = 92.922(2)°, γ = 90°, V = 1236.38(4)$Å^3$, Z = 4, Dc = 1.892g/cm^3, F000 = 680, μ= 20.327mm^{-1}, T = 170 K, θ_{max} = 80.006°, 8798 total reflections, 2385 with Io > 2σ(Io), R_{int} = 0.0400, 2620 data, 163 parameters, 0 restraints, GooF = 1.079, R = 0.0302 and wR= 0.0806 [Io > 2σ(Io)], R = 0.0335 and wR= 0.0827 (all reflections), 0.848<dΔρ<-1.093 e/$Å^3$.

13) Crystal data for NIP-**2** [CCDC-2297327]: Sum formula: $C_{13}H_8FIN_2O_2$, M = 370.11, colourless plate, 0.13x0.06x0.02 mm^3, monoclinic, space group $P2_1/n$, a = 15.7548(2)Å, b = 4.77590(10)Å, c = 16.7152(2)Å, α = 90°, β = 93.8410(10)°, γ = 90°, V = 1254.88(3)$Å^3$, Z = 4, Dc = 1.959g/cm^3, F000 = 712, μ= 20.185 mm^{-1}, T = 170 K, θ_{max} = 76.239°, 8278 total reflections, 2427 with Io > 2σ(Io), R_{int} = 0.0316, 2579 data, 151 parameters, 0 restraints, GooF = 1.107, R = 0.0366 and wR= 0.0986 [Io > 2σ(Io)], R = 0.0382 and wR= 0.0997 (all reflections), 1.419<dΔρ<-1.491 e/$Å^3$.

14) Crystal data for NIP-**3** [CCDC-2297334]: Sum formula: $C_{13}H_8ClIN_2O_2$, M = 386.56, colourless plate, 0.054x0.049x0.029 mm^3, triclinic, space group P-1, a = 8.0361(2)Å, b = 8.7865(2)Å, c = 9.9708(2)Å, α = 100.790(2)°, β = 91.074(2)°, γ = 107.116(2)°, V = 658.91(3)$Å^3$, Z = 1, Dc = 1.948g/cm^3, F000 = 372, μ= 20.964 mm^{-1}, T = 170 K, θ_{max} = 76.339°, 7183 total reflections, 2639 with Io > 2σ(Io), R_{int} = 0.0179, 2703 data, 172 parameters, 0 restraints, GooF = 1.063, R = 0.0144 and wR= 0.0370 [Io > 2σ(Io)], R = 0.0149 and wR= 0.0372 (all reflections), 0.373<dΔρ<-0.391 e/$Å^3$.

15) Crystal data for NIP-**4** [CCDC-2297329]: Sum formula: $C_{13}H_8BrIN_2O_2$, M = 431.02, colourless block, 0.07x0.054x0.021 mm^3, monoclinic, space group $P2_1/c$, a = 14.2203(2) Å, b = 13.41620(10) Å, c = 14.1484(2) Å, α = 90°, β = 99.5010(10) °, γ = 90°, V = 2662.24(6) $Å^3$, Z = 8, Dc = 2.151 g/cm^3, F000 = 1632, μ= 22.426 mm^{-1}, T = 120 K, θ_{max} = 76.823°, 41335 total reflections, 2639 with Io > 2σ(Io), R_{int} = 0.0446, 5554 data, 343 parameters, 0 restraints, GooF = 1.048, R = 0.0297 and wR= 0.0768 [Io > 2σ(Io)], R = 0.0320 and wR= 0.0781 (all reflections), 2.616 <dΔρ< -0.947 e/$Å^3$.

16) Crystal data for NIP-**5** [CCDC-2297328]: Sum formula: $C_{13}H_8I_2N_2O_2$, M = 478.01, colourless block, 0.07x0.06x0.03 mm^3, monoclinic, space group $P2_1/n$, a = 8.00690(10) Å, b = 20.1482(2) Å, c = 9.02500(10) Å, α = 90°, β = 103.3530(10) °, γ = 90°, V = 1416.59(3) $Å^3$, Z = 4, Dc = 2.241g/cm^3, F000 = 888, μ= 34.880 mm^{-1}, T = 120 K, θ_{max} = 79.695°, 10817 total reflections, 2761 with Io > 2σ(Io), R_{int} = 0.0380, 3002 data, 172 parameters, 0 restraints, GooF = 1.077, R = 0.0293 and wR= 0.0783 [Io > 2σ(Io)], R = 0.0319 and wR= 0.0797 (all reflections), 0.984<dΔρ<-1.039 e/$Å^3$.

17) Crystal data for NIP-**6** [CCDC-2297331]: Sum formula: $C_{14}H_8F_3IN_2O_2$, M = 420.12, colourless block, 0.173x0.086x0.049 mm^3, monoclinic, space group $P2_1/n$, a = 8.06450(10)Å, b = 16.6115(2)Å, c = 10.93320(10)Å, α = 90°, β = 101.7350(10)°, γ = 90°, V = 1434.04(3)$Å^3$, Z = 4, Dc = 1.946g/cm^3, F000 = 808, μ= 17.967 mm^{-1}, T = 120 K, θ_{max} = 79.620°, 10691 total reflections, 2852 with Io > 2σ(Io), R_{int} = 0.0379, 3034 data, 199 parameters, 0 restraints, GooF = 1.130, R = 0.0294 and wR= 0.0760 [Io > 2σ(Io)], R = 0.0312 and wR= 0.0772 (all reflections), 0.515<dΔρ<-0.852 e/$Å^3$.

18) Crystal data for NIP-**8** [CCDC-2297332]: Sum formula: $C_{14}H_{11}IN_2O_2$, M = 366.15, colourless block, 0.024x0.019x0.015 mm^3, monoclinic, space group $P2_1/c$, a = 11.8650(8)Å, b = 4.0903(3) Å, c = 27.3208(14) Å, α = 90°, β = 95.799(5)°, γ = 90°, V = 1319.13(15)$Å^3$, Z = 4, Dc = 1.844g/cm^3, F000 = 712, μ= 19.079 mm^{-1}, T = 120 K, θ_{max} = 79.176°, 17772 total reflections, 2561 with Io > 2σ(Io), R_{int} = 0.1193, 2916 data, 174 parameters, 0 restraints, GooF = 1.129, R = 0.0926 and wR= 0.2311 [Io > 2σ(Io)], R = 0.1074 and wR= 0.2509 (all reflections), 3.324<dΔρ<-2.063 e/$Å^3$.

19) Crystal data for NIP-**9** [CCDC-2297336]: Sum formula: $C_{15}H_{13}IN_2O_2$, M = 380.17, colourless block, 0.161x0.114x0.075 mm^3, orthorhombic space group P*2₁2₁2₁*, a = 8.12390(10)Å, b = 13.34730(10) Å, c = 13.42760(10) Å, α = 90°, β = 90°, γ = 90°, V = 1455.98(2)$Å^3$, Z = 4, Dc = 1.734g/cm^3, F000 = 744, μ= 17.311 mm^{-1}, T = 120 K, θ_{max} = 78.114°, 21185 total reflections, 3077 with Io > 2σ(Io), R_{int} = 0.0257, 3084 data, 182 parameters, 0 restraints, GooF = 1.087, R = 0.0134 and wR= 0.0340 [Io > 2σ(Io)], R = 0.0135 and wR= 0.0341 (all reflections), 0.342<dΔρ<-0.302 e/$Å^3$.

20) Crystal data for NIP-**12** [CCDC-2297330]: Sum formula: $C_{19}H_{13}IN_2O_2$, M = 428.21, colourless block, 0.1x0.09x0.07 mm^3, triclinic space group P-1, a = 8.4879(2) Å, b = 9.0871(2) Å, c = 11.4115(4) Å, α = 106.189(3)°, β = 98.364(2)°, γ = 93.869(2)°, V = 830.86(4)$Å^3$, Z = 2, Dc = 1.712 g/cm^3, F000 = 420, μ= 15.254 mm^{-1}, T = 120 K, θ_{max} = 79.563°, 9873 total reflections, 3331 with Io > 2σ(Io), R_{int} = 0.0420, 3509 data, 217 parameters, 0 restraints, GooF = 1.091, R = 0.0413 and wR= 0.1062 [Io > 2σ(Io)], R = 0.0429 and wR= 0.1072 (all reflections), 1.911<dΔρ<-1.125 e/$Å^3$.

21) Crystal data for NIP-**13** [CCDC-2297337]: Sum formula: $C_{26}H_{19}IN_2O_2$, M = 518.33, colourless block, 0.094x0.059x0.036 mm^3, triclinic space group P-1, a = 8.07700(10) Å, b = 10.56470(10) Å, c = 12.9041(2) Å, α = 93.3540(10)°, β = 102.5980(10)°, γ = 91.6500(10)°, V = 1071.79(2)$Å^3$, Z = 2, Dc = 1.606 g/cm^3, F000 = 516, μ= 11.943 mm^{-1}, T = 120 K, θ_{max} = 76.363°, 14827 total reflections, 4373 with Io > 2σ(Io), R_{int} = 0.0172, 4429 data, 280 parameters, 0 restraints, GooF = 1.140, R = 0.0186 and wR= 0.0460 [Io > 2σ(Io)], R = 0.0188 and wR= 0.0461 (all reflections), 0.336<dΔρ<-0.589 e/$Å^3$.

22) Crystal data for NIP-**14** [CCDC-2297335]: Sum formula: $C_{27}H_{21}IN_2O_2$, M = 532.36, colourless block, 0.122x0.07x0.058 mm^3, monoclinic space group $P2_1/c$, a = 11.47815(19)Å, b = 13.3347(3)Å, c = 14.7355(3)Å, α = 90°, β = 94.9703(15)°, γ = 90°, V = 2246.90(7)$Å^3$, Z = 4, Dc = 1.574 g/cm^3, F000 = 1064, μ= 11.410 mm^{-1}, T = 120 K, θ_{max} = 76.711°, 8294 total reflections, 7749 with Io > 2σ(Io), R_{int} = twin, 8294 data, 291 parameters, 0 restraints, GooF = 1.077, R = 0.0448 and wR= 0.1203 [Io > 2σ(Io)], R = 0.0464 and wR= 0.1219 (all reflections), 0.645<dΔρ<-1.058 e/$Å^3$.

23) Crystal data for NISac-**2** [CCDC-2297287]: Sum formula: $C_{12}H_8FIN_2O_3S$, M = 406.16, colourless plate, 0.097x0.061x0.021 mm^3, monoclinic space group $P2_1/m$, a = 7.3022(3)Å, b = 6.6219(2)Å, c = 13.8245(4)Å, α = 90°, β = 93.769(3)°, γ = 90°, V = 667.03(4)$Å^3$, Z = 2, Dc = 2.022 g/cm^3, F000 = 392, μ= 20.542 mm^{-1}, T = 120 K, θ_{max} = 78.601°, 3879 total reflections, 1413 with Io > 2σ(Io), R_{int} = 0.0528, 1502 data, 118 parameters, 0 restraints, GooF = 1.113, R = 0.0394 and wR= 0.1081 [Io > 2σ(Io)], R = 0.0414 and wR= 0.1097 (all reflections), 1.728 <dΔρ<-1.595 e/$Å^3$.

24) Crystal data for NISac-**3** [CCDC- 2297289]: Sum formula: $C_{12}H_8ClIN_2O_3S$, M = 422.61, colourless block, 0.077x0.048x0.039 mm^3, triclinic space group P-1, a = 7.6544(14)Å, b = 8.2902(12)Å, c = 11.7396(14)Å, α = 89.331(11)°, β = 71.252(14)°, γ = 88.928(13)°, V = 705.28(19) $Å^3$, Z = 2, Dc = 1.990 g/cm^3, F000 = 408, μ= 2.614 mm^{-1}, T = 120 K, θ_{max} = 25.250°, 5134 total reflections, 2245 with Io > 2σ(Io), R_{int} = 0.0557, 2550 data, 170 parameters, 18 restraints, GooF = 1.191, R = 0.0954 and wR= 0.2577 [Io > 2σ(Io)], R = 0.1063 and wR= 0.2644 (all reflections), 2.306 <dΔρ<-2.485 e/$Å^3$.

25) Crystal data for NISac-**4** [CCDC-2297293]: Sum formula: $C_{12}H_8BrIN_2O_3S$, M = 467.07, colourless block, 0.09x0.07x0.04 mm^3, orthorhombic space group P*bca*, a = 7.54700(8)Å, b = 14.57493(20)Å, c = 26.1769(3)Å, α = 90°, β = 90°, γ = 90°, V = 2879.38(6) $Å^3$, Z = 8, Dc = 2.155 g/cm^3, F000 = 1776,

μ= 22.175 mm^{-1}, T = 120 K, θ_{max} = 79.444°, 24371 total reflections, 2954 with Io > 2σ(Io), R_{int} = 0.0436, 3086 data, 186 parameters, 18 restraints, GooF = 1.096, R = 0.0260 and wR= 0.0694 [Io > 2σ(Io)], R = 0.0271 and wR= 0.0700 (all reflections), $0.644 < d\Delta\rho < -0.462$ e/Å^3.

26) Crystal data for NISac-**5** [CCDC-2297292]: Sum formula: $C_{12}H_8I_2N_2O_3S$, M = 514.06, colourless block, 0.06x0.05x0.04 mm^3, triclinic space group P-1, a = 7.8104(7) Å, b = 7.8674(5)Å, c = 13.5968(12)Å, α = 75.592(7)°, β = 78.108(7)°, γ = 64.596(8)°, V = 726.24(11) Å^3, Z = 2, Dc = 2.351g/cm^3, F000 = 480, μ= 35.446 mm^{-1}, T = 120 K, θ_{max} = 79.444°, 10596 total reflections, 2869 with Io > 2σ(Io), R_{int} = 0.0312, 3078 data, 181 parameters, 0 restraints, GooF = 1.056, R = 0.0246 and wR= 0.0642 [Io > 2σ(Io)], R = 0.0266 and wR= 0.0652 (all reflections), $0.640 < d\Delta\rho < -1.139$ e/Å^3.

27) Crystal data for NISac-**6** [CCDC-2297290]: Sum formula: $C_{13}H_8F_3IN_2O_3S$, M = 456.17, colourless block, 0.036x0.022x0.019 mm^3, triclinic space group P-1, a = 7.8380(16) Å, b = 8.5317(17)Å, c = 12.218(2)Å, α = 83.65(3)°, β = 81.29(3)°, γ = 65.81(3)°, V = 735.6(3) Å^3, Z = 2, Dc = 2.059g/cm^3, F000 = 440, μ= 2.367 mm^{-1}, T = 170 K, θ_{max} = 25.248°, 6156 total reflections, 2136 with Io > 2σ(Io), R_{int} = 0.0581, 2670 data, 208 parameters, 0 restraints, GooF = 1.093, R = 0.0569 and wR= 0.1020 [Io > 2σ(Io)], R = 0.0798 and wR= 0.1105 (all reflections), $0.781 < d\Delta\rho < -0.767$ e/Å^3.

28) Crystal data for NISac-**7** [CCDC-2297288]: Sum formula: $C_{13}H_8IN_3O_3S$, M = 413.18, colourless block, 0.03x0.023x0.014 mm^3, triclinic space group P-1, a = 7.9971(16) Å, b = 8.0226(16)Å, c = 11.988(2)Å, α = 89.73(3)°, β = 71.28(3)°, γ = 85.76(3)°, V = 726.3(3) Å^3, Z = 2, Dc = 1.889 g/cm^3, F000 = 400, μ= 2.361 mm^{-1}, T = 120 K, θ_{max} = 25.246°, 5963 total reflections, 2348 with Io > 2σ(Io), R_{int} = 0.0350, 2633 data, 190 parameters, 0 restraints, GooF = 1.087, R = 0.0349 and wR= 0.0733 [Io > 2σ(Io)], R = 0.0407 and wR= 0.0761 (all reflections), $0.517 < d\Delta\rho < -0.452$ e/Å^3.

29) Crystal data for NISac-**9** [CCDC-2297291]: Sum formula: $C_{13}H_8IN_3O_3S$, M = 413.18, colourless block, 0.03x0.023x0.014 mm^3, triclinic space group P-1, a = 7.9971(16) Å, b = 8.0226(16)Å, c = 11.988(2)Å, α = 89.73(3)°, β = 71.28(3)°, γ = 85.76(3)°, V = 726.3(3) Å^3, Z = 2, Dc = 1.889 g/cm^3, F000 = 400, μ= 2.361 mm^{-1}, T = 120 K, θ_{max} = 25.246°, 5963 total reflections, 2348 with Io > 2σ(Io), R_{int} = 0.0350, 2633 data, 190 parameters, 0 restraints, GooF = 1.087, R = 0.0349 and wR= 0.0733 [Io > 2σ(Io)], R = 0.0407 and wR= 0.0761 (all reflections), $0.517 < d\Delta\rho < -0.452$ e/Å^3.

30) Crystal data for NISac-**11** [CCDC-2297294]: Sum formula: $C_{13}H_{11}IN_2O_4S$, M = 418.20, colourless block, 0.3x0.18x0.13 mm^3, monoclinic space group P2$_1$/n, a = 7.9337(16) Å, b = 16.262(3) Å, c = 11.312(2) Å, α = 90°, β = 91.58(3)°, γ = 90°, V = 1458.9(5) Å^3, Z = 4, Dc = 1.904 g/cm^3, F000 = 816, μ= 2.355 mm^{-1}, T = 170 K, θ_{max} = 25.249°, 6447 total reflections, 2233 with Io > 2σ(Io), R_{int} = 0.0948, 2640 data, 192 parameters, 0 restraints, GooF = 1.085, R = 0.0349 and wR= 0.2639 [Io > 2σ(Io)], R = 0.1026 and wR= 0.2701 (all reflections), $7.880 < d\Delta\rho < -2.558$ e/Å^3.

31) Crystal data for NBS-**1** [CCDC-2297298]: Sum formula: $C_9H_9BrN_2O_2$, M = 257.09, colourless block, 0.3x0.18x0.13 mm^3, monoclinic space group P2$_1$/n, a = 7.3961(15) Å, b = 21.584(4) Å, c = 24.831(5) Å, α = 90°, β = 97.97(3)°, γ = 90°, V = 3925.7(14) Å^3, Z = 16, Dc = 1.740 g/cm^3, F000 = 2048, μ= 4.163 mm^{-1}, T = 170 K, θ_{max} = 25.250°, 104377 total reflections, 6162 with Io > 2σ(Io), R_{int} = 0.0494, 7102 data, 505 parameters, 0 restraints, GooF = 1.062, R = 0.0246 and wR= 0.0459 [Io > 2σ(Io)], R = 0.0325 and wR= 0.0486 (all reflections), $0.302 < d\Delta\rho < -0.338$e/Å^3.

32) Crystal data for NBS-**3** [CCDC-2297296]: Sum formula: $C_9H_8BrClN_2O_2$, M = 291.53, colourless block, 0.1x0.077x0.02 mm^3, orthorhombic space group P*bca*, a = 11.5530(3) Å, b = 7.9623(2) Å, c = 23.2624(5) Å, α = 90°, β = 90°, γ = 90°, V = 2139.87(9) Å^3, Z = 8, Dc = 1.810 g/cm^3, F000 = 1152, μ= 4.072 mm^{-1}, T = 170 K, θ_{max} = 29.634°, 9185 total reflections, 2101 with Io > 2σ(Io), R_{int} = 0.0278, 2708 data, 136 parameters, 0 restraints, GooF = 1.048, R = 0.0273 and wR= 0.0521 [Io > 2σ(Io)], R = 0.0446 and wR= 0.0570 (all reflections), $0.310 < d\Delta\rho < -0.372$/Å^3.

33) Crystal data for NBS-**4** [CCDC-2297297]: Sum formula: $C_9H_8Br_2N_2O_2$, M = 335.99, colourless block, 0.11x0.09x0.025 mm^3, orthorhombic space group P*bca*, a = 11.615(2)Å, b = 7.9801(16) Å, c = 23.403(5)Å, α = 90°, β = 90°, γ = 90°, V = 2169.2(8) $Å^3$, Z = 8, Dc = 2.058 g/cm^3, F000 = 1296, μ= 7.455 mm^{-1}, T = 170 K, θ_{max} = 25.244°, 10599 total reflections, 1345 with Io > 2σ(Io), R_{int} = 0.1019, 1960 data, 136 parameters, 0 restraints, GooF = 1.054, R = 0.0543 and wR= 0.0959 [Io > 2σ(Io)], R = 0.0921 and wR= 0.1087 (all reflections), 0.717<dΔρ<-0.690/$Å^3$.

34) Crystal data for NBS-**5** [CCDC-2297300]: Sum formula: $C_9H_8BrIN_2O_2$, M = 382.98, colourless block, 0.077x0.064x0.02 mm^3, orthorhombic space group P*bca*, a = 11.7666(4)Å, b = 8.1712(2) Å, c = 23.5011(8)Å, α = 90°, β = 90°, γ = 90°, V = 2259.57(12) $Å^3$, Z = 8, Dc = 2.252 g/cm^3, F000 = 1440, μ= 26.295 mm^{-1}, T = 120 K, θ_{max} = 78.483°, 21554 total reflections, 2289 with Io > 2σ(Io), R_{int} = 0.0519, 2415 data, 136 parameters, 0 restraints, GooF = 1.211, R = 0.0443 and wR= 0.1076 [Io > 2σ(Io)], R = 0.0464 and wR= 0.1085 (all reflections), 1.180<dΔρ<-1.134/$Å^3$.

35) Crystal data for NBS-**8** [CCDC-2297301]: Sum formula: $C_{10}H_{11}BrN_2O_2$, M = 271.12, colourless block, 0.12x0.1x0.1 mm^3, orthorhombic space group P*2₁2₁2₁*, a = 5.9809(12)Å, b = 8.9612(18) Å, c = 20.756(4)Å, α = 90°, β = 90°, γ = 90°, V = 1112.4(4) $Å^3$, Z = 4, Dc = 1.619 g/cm^3, F000 = 544, μ= 3.677 mm^{-1}, T = 170 K, θ_{max} = 25.250°, 21125 total reflections, 1943 with Io > 2σ(Io), R_{int} = 0.0434, 2009 data, 137 parameters, 0 restraints, GooF = 1.065, R = 0.0204 and wR= 0.0450 [Io > 2σ(Io)], R = 0.0220 and wR= 0.0455 (all reflections), 0.252<dΔρ<-0.176/$Å^3$.

36) Crystal data for NBS-**9** [CCDC-2297299]: Sum formula: $C_{11}H_{13}BrN_2O_2$, M = 285.14, colourless block, 0.284x0.24x0.068 mm^3, monoclinic space group $P2_1$, a = 9.2312(2)Å, b = 5.85165(13) Å, c = 11.3669(3)Å, α = 90°, β = 109.939(3)°, γ = 90°, V = 577.21(3)$Å^3$, Z = 4, Dc = 1.641 g/cm^3, F000 = 288, μ= 3.548 mm^{-1}, T = 170 K, θ_{max} = 25.246°, 4253 total reflections, 1989 with Io > 2σ(Io), R_{int} = 0.0300, 2046 data, 146 parameters, 1 restraints, GooF = 1.063, R = 0.0260 and wR= 0.0593 [Io > 2σ(Io)], R = 0.0277 and wR= 0.0605 (all reflections), 0.484<dΔρ<-0.329/$Å^3$.

37) Crystal data for NBP-**1** [CCDC-2297308]: Sum formula: $C_{13}H_9BrN_2O_2$, M = 305.13, colourless block, 0.12x0.07x0.04 mm^3, monoclinic space group $P2_1/n$, a = 14.2718(5)Å, b = 5.1259(2)Å, c = 16.5122(6)Å, α = 90°, β = 97.507(4)°, γ = 90°, V = 1197.61(8)$Å^3$, Z = 4, Dc = 1.692 g/cm^3, F000 = 608, μ= 4.652 mm^{-1}, T = 120 K, θ_{max} = 79.591°, 7788 total reflections, 1952 with Io > 2σ(Io), R_{int} = 0.0460, 2522 data, 163 parameters, 0 restraints, GooF = 1.127, R = 0.0514 and wR= 0.1460 [Io > 2σ(Io)], R = 0.0633 and wR= 0.1551 (all reflections), 0.839<dΔρ<-1.734/$Å^3$.

38) Crystal data for NBP-**2** [CCDC-2297306]: Sum formula: $C_{13}H_8BrFN_2O_2$, M = 323.12, colourless block, 0.08x0.04x0.03 mm^3, monoclinic space group $P2_1/c$, a = 4.12160(10)Å, b = 25.1715(5)Å, c = 11.6335(2)Å, α = 90°, β = 92.510(2)°, γ = 90°, V = 1205.78(4)$Å^3$, Z = 4, Dc = 1.780 g/cm^3, F000 = 640, μ= 4.786 mm^{-1}, T = 120 K, θ_{max} = 79.281°, 7798 total reflections, 2169 with Io > 2σ(Io), R_{int} = 0.0353, 2489 data, 172 parameters, 0 restraints, GooF = 1.132, R = 0.0330 and wR= 0.0754 [Io > 2σ(Io)], R = 0.0429 and wR= 0.0883 (all reflections), 0.478<dΔρ<-0.784/$Å^3$.

39) Crystal data for NBP-**3** [CCDC-2297311]: Sum formula: $C_{13}H_8BrClN_2O_2$, M = 339.57, colourless block, 0.024x0.023x0.023 mm^3, monoclinic space group $P2_1/c$, a = 14.079(3)Å, b = 13.204(3)Å, c = 13.898(3)Å, α = 90°, β = 100.06(3)°, γ = 90°, V = 2543.8(9)$Å^3$, Z = 8, Dc = 1.773 g/cm^3, F000 = 1344, μ= 3.440 mm^{-1}, T = 170 K, θ_{max} = 25.248°, 18882 total reflections, 3793with Io > 2σ(Io), R_{int} = 0.0387, 4593 data, 343 parameters, 0 restraints, GooF = 1.025, R = 0.0304 and wR= 0.0612 [Io > 2σ(Io)], R = 0.0431 and wR= 0.0657 (all reflections), 0.332<dΔρ<-0.306/$Å^3$.

40) Crystal data for NBP-**4** [CCDC-2297309]: Sum formula: $C_{13}H_8Br_2N_2O_2$, M = 384.03, colourless block, 0.019x0.017x0.017 mm^3, monoclinic space group $P2_1/c$, a = 14.125(3) Å, b = 13.378(3)Å, c = 13.951(3)Å, α = 90°, β = 99.81(3)°, γ = 90°, V = 2597.6(9)$Å^3$, Z = 8, Dc = 1.964 g/cm^3, F000 = 1488,

μ= 6.239 mm^{-1}, T = 170 K, θ_{max} = 25.250°, 13993 total reflections, 2977 with Io > 2σ(Io), R_{int} = 0.0800, 4705 data, 343 parameters, 0 restraints, GooF = 1.065, R = 0.0641 and wR= 0.1073 [Io > 2σ(Io)], R = 0.1147 and wR= 0.1223 (all reflections), 0.688<d$\Delta\rho$<-0.730/Å^3.

41) Crystal data for NBP-**5** [CCDC-2297315]: Sum formula: $C_{13}H_8BrIN_2O_2$, M = 431.02, colourless block, 0.012x0.08x0.05 mm^3, triclinic space group P-1, a = 8.1950(3)Å, b = 8.4808(3)Å, c = 10.9367(3)Å, α = 75.266(3)°, β = 76.867(3)°, γ = 67.854(3)°, V = 673.52(4)Å^3, Z = 2, Dc = 2.125 g/cm^3, F000 = 408, μ= 22.161mm^{-1}, T = 120 K, θ_{max} = 79.616°, 7954 total reflections, 2744 with Io > 2σ(Io), R_{int} = 0.0349, 2828 data, 172 parameters, 0 restraints, GooF = 1.097, R = 0.0315 and wR= 0.0829 [Io > 2σ(Io)], R = 0.0322 and wR= 0.0834 (all reflections), 0.896<d$\Delta\rho$<-1.105/Å^3.

42) Crystal data for NBP-**6** [CCDC-2297307]: Sum formula: $C_{14}H_8BrF_3N_2O_2$, M = 373.13, colourless block, 0.281x0.141x0.097 mm^3, monoclinic space group P2$_1$/c, a = 8.3190(4) Å, b = 23.8710(7) Å, c = 7.8504(4) Å, α = 90°, β = 115.497(6) °, γ = 90°, V = 1407.11(12) Å^3, Z = 4, Dc = 1.761 g/cm^3, F000 = 736, μ= 2.963 mm^{-1}, T = 120 K, θ_{max} = 25.250°, 10235 total reflections, 2271 with Io > 2σ(Io), R_{int} = 0.0351, 2549 data, 199 parameters, 0 restraints, GooF = 1.040, R = 0.0252 and wR= 0.0552 [Io > 2σ(Io)], R = 0.0308 and wR= 0.0581 (all reflections), 0.355 <d$\Delta\rho$< -0.304/Å^3.

43) Crystal data for NBP-**7** [CCDC-2297316]: Sum formula: $C_{14}H_8BrN_3O_2$, M = 330.14, colourless plate, 0.021x0.02x0.013 mm^3, triclinic space group P-1, a = 8.2492(16)Å, b = 8.6144(17)Å, c = 9.842(2)Å, α = 101.38(3)°, β = 95.88(3) °, γ = 106.01(3)°, V = 649.9(3) Å^3, Z = 2, Dc = 1.687g/cm^3, F000 = 328, μ= 3.167 mm^{-1}, T = 170 K, θ_{max} = 25.247°, 5075 total reflections, 1815 with Io > 2σ(Io), R_{int} = 0.0571, 2348 data, 181 parameters, 0 restraints, GooF = 1.041, R = 0.0621 and wR= 0.1162 [Io > 2σ(Io)], R = 0.0887 and wR= 0.1275 (all reflections), 0.641 <d$\Delta\rho$<-0.595/Å^3.

44) Crystal data for NBP-**8** [CCDC-2297312]: Sum formula: $C_{14}H_{11}BrN_2O_2$, M = 319.16, colourless plate, 0.17x0.15x0.08 mm^3, orthorhombic space group P*nna*, a = 14.223(3)Å, b = 19.742(4)Å, c = 18.532(4)Å, α = 90°, β = 90°, γ = 90°, V = 5203.6(18) Å^3, Z = 16, Dc = 1.630g/cm^3, F000 = 2560, μ= 3.158 mm^{-1}, T = 170 K, θ_{max} = 25.249°, 104247 total reflections, 3521 with Io > 2σ(Io), R_{int} = 0.0809, 4718 data, 345 parameters, 0 restraints, GooF = 1.016, R = 0.0307 and wR= 0.0629 [Io > 2σ(Io)], R = 0.0517 and wR= 0.0713 (all reflections), 0.366 <d$\Delta\rho$<-0.285/Å^3.

45) Crystal data for NBP-**9** [CCDC-2297318]: Sum formula: $C_{15}H_{13}BrN_2O_2$, M = 333.18, colourless block, 0.057x0.039x0.016 mm^3, monoclinic space group P2$_1$/n, a = 12.7634(4)Å, b = 8.6393(3)Å, c = 13.1886(4)Å, α = 90°, β = 107.488(4)°, γ = 90°, V = 1387.05(8)Å^3, Z = 4, Dc = 1.596g/cm^3, F000 = 672, μ= 4.069 mm^{-1}, T = 120 K, θ_{max} = 79.913°, 10469 total reflections, 2245 with Io > 2σ(Io), R_{int} = 0.0533, 2938 data, 182 parameters, 0 restraints, GooF = 1.064, R = 0.0400 and wR= 0.0885 [Io > 2σ(Io)], R = 0.0582 and wR= 0.0954 (all reflections), 0.378<d$\Delta\rho$<-0.625/Å^3.

46) Crystal data for NBP-**10** [CCDC-2297310]: Sum formula: $C_{16}H_{15}BrN_2O_2$, M = 347.21, colourless block, 0.1x0.08x0.06 mm^3, triclinic space group P-1, a = 8.4886(2)Å, b = 12.1249(3)Å, c = 15.6600(4)Å, α = 68.744(2)°, β = 88.188(2)°, γ = 89.816(2)°, V = 1501.32(7)Å^3, Z = 4, Dc = 1.536g/cm^3, F000 = 704, μ= 3.784 mm^{-1}, T = 120 K, θ_{max} = 80.890°, 41171 total reflections, 6027 with Io > 2σ(Io), R_{int} = 0.0602, 6355 data, 383 parameters, 0 restraints, GooF = 1.144, R = 0.0828 and wR= 0.1999 [Io > 2σ(Io)], R = 0.0850 and wR= 0.2013 (all reflections), 2.982<d$\Delta\rho$<-1.050/Å^3.

47) Crystal data for NBP-**11** [CCDC-2297314]: Sum formula: $C_{14}H_{11}BrN_2O_3$, M = 335.16, colourless block, 0.052x0.035x0.021 mm^3, monoclinic space group *P*2$_1$/m, a = 11.8151(2)Å, b = 4.02450(10)Å, c = 13.7118(2)Å, α = 90°, β = 91.0340(10)°, γ = 90°, V = 651.89(2)Å^3, Z = 2, Dc = 1.707 g/cm^3, F000 = 336, μ= 4.395 mm^{-1}, T = 120 K, θ_{max} = 77.929°, 7571 total reflections, 1439 with Io > 2σ(Io), R_{int} = 0.0287, 1539 data, 149 parameters, 0 restraints, GooF = 1.082, R = 0.0310 and wR= 0.0781 [Io > 2σ(Io)], R = 0.0335 and wR= 0.0792 (all reflections), 0.599 <d$\Delta\rho$<-0.780/Å^3.

48) Crystal data for NBP-**12** [CCDC-2297313]: Sum formula: $C_{19}H_{13}BrN_2O_2$, M = 381.22, colourless block, 0.016x0.015x0.015 mm^3, triclinic space group P-1, a = 8.4132(17)Å, b = 9.0911(18)Å, c = 11.335(2)Å, α = 107.41(3)°, β = 97.73(3)°, γ = 94.77(3)°, V = 812.8(3)Å^3, Z = 2, Dc = 1.558 g/cm^3, F000 = 384, μ= 2.542 mm^{-1}, T = 170 K, θ_{max} = 25.247°, 6620 total reflections, 2558 with Io > 2σ(Io), R_{int} = 0.0317, 2930 data, 217 parameters, 0 restraints, GooF = 1.049, R = 0.0310 and wR= 0.0621 [Io > 2σ(Io)], R = 0.0306 and wR= 0.0649 (all reflections), 0.287 <dΔρ<-0.293/Å^3.

49) Crystal data for NBP-**13** [CCDC-2297317]: Sum formula: $C_{26}H_{19}BrN_2O_2$, M = 471.34, colourless block, 0.29x0.16x0.1 mm^3, triclinic space group P-1, a = 8.1605(16)Å, b = 10.591(2)Å, c = 12.571(3)Å, α = 92.56(3)°, β = 101.63(3)°, γ = 90.74(3)°, V = 1062.9(4)Å^3, Z = 2, Dc = 1.473 g/cm^3, F000 = 480, μ= 2.542 mm^{-1}, T = 170 K, θ_{max} = 25.247°, 8384 total reflections, 3223 with Io > 2σ(Io), R_{int} = 0.0332, 3831 data, 280 parameters, 0 restraints, GooF = 1.047, R = 0.0401 and wR= 0.0781 [Io > 2σ(Io)], R = 0.0533 and wR= 0.0828 (all reflections), 0.290 <dΔρ<-0.361/Å^3.

50) Crystal data for NBSac-**2** [CCDC-2297346]: Sum formula: $C_{12}H_8BrFN_2O_3S$, M = 359.17, colourless block, 0.04x0.034x0.01 mm^3, orthorhombic space group P*nma*, a = 24.515(5)Å, b = 6.7797(14)Å, c = 7.8773(16)Å, α = 90°, β = 90°, γ = 90°, V = 1309.3(5)Å^3, Z = 2, Dc = 1.822 g/cm^3, F000 = 712, μ= 3.319 mm^{-1}, T = 170 K, θ_{max} = 25.244°, 8081 total reflections, 1092 with Io > 2σ(Io), R_{int} = 0.0454, 1289 data, 109 parameters, 24 restraints, GooF = 1.058, R = 0.0349 and wR= 0.0838 [Io > 2σ(Io)], R = 0.0456 and wR= 0.0882 (all reflections), 0.653 <dΔρ<-0.633/Å^3.

51) Crystal data for NBSac-**3** [CCDC-2297342]: Sum formula: $C_{12}H_8BrClN_2O_3S$, M = 375.62, colourless block, 0.216x0.136x0.087 mm^3, monoclinic space group $P2_1/c$, a = 8.1158(3)Å, b = 23.6854(8)Å, c = 7.6446(3)Å, α = 90°, β = 113.898(5)°, γ = 90°, V = 1343.53(10)Å^3, Z = 2, Dc = 1.857 g/cm^3, F000 = 744, μ= 3.421 mm^{-1}, T = 170 K, θ_{max} = 25.247°, 7356 total reflections, 2177 with Io > 2σ(Io), R_{int} = 0.0260, 2429 data, 181 parameters, 0 restraints, GooF = 1.057, R = 0.0229 and wR= 0.0472 [Io > 2σ(Io)], R = 0.0281 and wR= 0.0493 (all reflections), 0.357 <dΔρ<-0.346/Å^3.

52) Crystal data for NBSac-**4** [CCDC-2297340]: Sum formula: $C_{12}H_8Br_2N_2O_3S$, M = 420.08, colourless plate, 0.01x0.01x0.01 mm^3, monoclinic space group $P2_1/c$, a = 8.1907(16)Å, b = 23.803(5)Å, c = 7.8308(16)Å, α = 90°, β = 115.31(3)°, γ = 90°, V = 1380.2(6)Å^3, Z = 4, Dc = 2.022 g/cm^3, F000 = 816, μ= 6.032 mm^{-1}, T = 170 K, θ_{max} = 25.250°, 10371 total reflections, 1604 with Io > 2σ(Io), R_{int} = 0.1125, 2503 data, 181 parameters, 6 restraints, GooF = 1.054, R = 0.0698 and wR= 0.1132 [Io > 2σ(Io)], R = 0.1207 and wR= 0.1293 (all reflections), 0.896 <dΔρ<-0.711/Å^3.

53) Crystal data for NBSac-**5** [CCDC-2297338]: Sum formula: $C_{12}H_8BrIN_2O_3S$, M = 467.07, colourless plate, 0.18x0.14x0.14 mm^3, triclinic space group P-1, a = 7.6843(15)Å, b = 7.9698(16)Å, c = 13.624(3)Å, α = 75.74(3)°, β = 76.07(3)°, γ = 63.09(3)°, V = 713.0(3)Å^3, Z = 4, Dc = 2.175 g/cm^3, F000 = 444, μ= 5.201 mm^{-1}, T = 170 K, θ_{max} = 25.250°, 5165 total reflections, 2131 with Io > 2σ(Io), R_{int} = 0.0358, 2564 data, 181 parameters, 0 restraints, GooF = 1.177, R = 0.0485 and wR= 0.1110 [Io > 2σ(Io)], R = 0.0623 and wR= 0.1159 (all reflections), 1.007<dΔρ<-0.713/Å^3.

54) Crystal data for NBSac-**5a** [CCDC-2297344]: Sum formula: $C_{12}H_8BrIN_2O_3S$, M = 467.07, colourless plate, 0.016x0.014x0.014 mm^3, monoclinic space group $P2_1/c$, a = 8.3444(17)Å, b = 23.800(5)Å, c = 8.0893(16)Å, α = 90°, β = 117.32(3)°, γ = 90°, V = 1427.3(6)Å^3, Z = 4, Dc = 2.174 g/cm^3, F000 = 888, μ= 5.196 mm^{-1}, T = 170 K, θ_{max} = 25.245°, 11331 total reflections, 2097 with Io > 2σ(Io), R_{int} = 0.0566, 2578 data, 181 parameters, 0 restraints, GooF = 1.177, R = 0.0390 and wR= 0.0701 [Io > 2σ(Io)], R = 0.0548 and wR= 0.0747 (all reflections), 0.574<dΔρ<-0.638/Å^3.

55) Crystal data for NBSac-**6** [CCDC-2297339]: Sum formula: $C_{13}H_8BrF_3N_2O_3S$, M = 409.18, colourless block, 0.09x0.03x0.03 mm^3, triclinic space group P-1, a = 7.7643(2)Å, b = 8.3599(2)Å, c = 12.3063(5)Å, α = 83.758(3)°, β = 80.066(3)°, γ = 64.773(3)°, V = 711.21(4)Å^3, Z = 2, Dc = 1.911 g/cm^3,

F000 = 404, μ= 5.820 mm^{-1}, T = 120 K, θ_{max} = 79.516°, 8390 total reflections, 7909 with Io > 2σ(Io), R_{int} = twin, 8390 data, 209 parameters, 0 restraints, GooF = 1.151, R = 0.0354 and wR= 0.0970 [Io > 2σ(Io)], R = 0.0373 and wR= 0.0979 (all reflections), 1.340<dΔρ<-0.620/Å^3.

56) Crystal data for NBSac-**9** [CCDC-2297345]: Sum formula: $C_{14}H_{13}BrN_2O_3S$, M = 369.23, colourless block, 0.14x0.08x0.05 mm^3, monoclinic space group $P2_1/c$, a = 8.55670(10)Å, b = 23.9780(3)Å, c = 7.75550(10)Å, α = 90°, β = 116.634(2)°, γ = 90°, V = 1422.37(4)Å^3, Z = 4, Dc = 1.724 g/cm^3, F000 = 744, μ= 5.426 mm^{-1}, T = 120 K, θ_{max} = 78.168°, 27511 total reflections, 2926 with Io > 2σ(Io), R_{int} = 0.0343, 3024 data, 191 parameters, 0 restraints, GooF = 1.188, R = 0.0305 and wR= 0.0691 [Io > 2σ(Io)], R = 0.0316 and wR= 0.0696 (all reflections), 0.665<dΔρ<-0.427/Å^3.

57) Crystal data for NBSac-**13** [CCDC-2297343]: Sum formula: $C_{25}H_{19}BrN_2O_3S$, M = 507.39, colourless block, 0.094x0.058x0.034 mm^3, triclinic space group P-1, a = 8.3067(2)Å, b = 9.7906(3)Å, c = 14.6480(3)Å, α = 92.242(2)°, β = 96.857(2)°, γ = 112.351(2)°, V = 1089.26(5)Å^3, Z = 2, Dc = 1.547 g/cm^3, F000 = 516, μ= 3.725 mm^{-1}, T = 120 K, θ_{max} = 80.046°, 13696 total reflections, 4179 with Io > 2σ(Io), R_{int} = 0.0350, 4607 data, 289 parameters, 0 restraints, GooF = 1.091, R = 0.0385 and wR= 0.1046 [Io > 2σ(Io)], R = 0.0421 and wR= 0.1068 (all reflections), 0.946 <dΔρ<-0.673 /Å^3.

58) Crystal data for NBSac-**14** [CCDC-2297341]: Sum formula: $C_{26}H_{21}BrN_2O_3S$, M = 521.42, colourless block, 0.061x0.049x0.023 mm^3, monoclinic space group Cc, a = 18.7927(2) Å, b = 7.78420(10) Å, c = 16.3844(2) Å, α = 90°, β = 108.590(2) °, γ = 90°, V = 2271.75(5) Å^3, Z = 4, Dc = 1.525 g/cm^3, F000 = 1064, μ= 3.588 mm^{-1}, T = 120 K, θ_{max} = 79.823 °, 22391 total reflections, 4144 with Io > 2σ(Io), R_{int} = 0.0455, 4302 data, 299 parameters, 2 restraints, GooF = 1.109, R = 0.0395 and wR= 0.1020 [Io > 2σ(Io)], R = 0.0415 and wR= 0.1033 (all reflections), 0.484 <dΔρ< -0.779 /Å^3.

59) Crystal data for (NSac)$_2$I-**10**H [CCDC- 2297351]: Sum formula: $C_{22}H_{20}IN_3O_6S_2$, M = 613.43, colourless block, 0.403x0.259x0.168 mm^3, monoclinic space group $P2_1/n$, a = 13.4412(3) Å, b = 9.8116(3) Å, c = 17.9192(4) Å, α = 90°, β = 91.614(2) °, γ = 90°, V = 2362.24(10) Å^3, Z = 4, Dc = 1.725 g/cm^3, F000 = 1224, μ= 1.577 mm^{-1}, T = 120 K, θ_{max} = 26.371 °, 9787 total reflections, 4077 with Io > 2σ(Io), R_{int} = 0.0298, 4831 data, 309 parameters, 0 restraints, GooF = 1.066, R = 0.0684 and wR= 0.1609 [Io > 2σ(Io)], R = 0.0595 and wR= 0.1718 (all reflections), 3.051 <dΔρ< -1.761 /Å^3.

60) Crystal data for (NSac)$_2$I-**12**H [CCDC-2297348]: Sum formula: $C_{25}H_{18}IN_3O_6S_2$, M = 647.44, colourless block, 0.023x0.02x0.02 mm^3, monoclinic space group C2/c, a = 12.703(3)Å, b = 10.867(2)Å, c = 17.869(4)Å, α = 90°, β = 90.26(3)°, γ = 90°, V = 2466.9(9) Å^3, Z = 4, Dc = 1.743 g/cm^3, F000 = 1288, μ= 1.516 mm^{-1}, T = 170 K, θ_{max} = 28.881°, 11333 total reflections, 2682 with Io > 2σ(Io), R_{int} = 0.0318, 3237 data, 215 parameters, 6 restraints, GooF = 1.042, R = 0.0285 and wR= 0.0597 [Io > 2σ(Io)], R = 0.0384 and wR= 0.0631 (all reflections), 0.316 <dΔρ<-0.353 /Å^3.

61) Crystal data for (NSac)$_2$I-**15**H [CCDC-2297349]: Sum formula: $C_{22}H_{22}IN_3O_6S_2Si$, M = 647.44, colourless block, 0.032x0.024x0.02 mm^3, triclinic space group P-1, a = 8.0649(10)Å, b = 9.3955(11)Å, c = 16.8787(14)Å, α = 90.644(7)°, β = 90.165(7)°, γ = 94.442(4)°, V = 1275.0(2) Å^3, Z = 2, Dc = 1.676 g/cm^3, F000 = 644, μ= 1.510 mm^{-1}, T = 170 K, θ_{max} = 27.103°, 9133 total reflections, 4717 with Io > 2σ(Io), R_{int} = 0.0357, 5494 data, 319 parameters, 0 restraints, GooF = 1.110, R = 0.0625 and wR= 0.1962 [Io > 2σ(Io)], R = 0.0729 and wR= 0.2069 (all reflections), 1.210<dΔρ<-2.236 /Å^3.

62) Crystal data for (NSac)$_2$I-**15**Ha [CCDC-2297350]: Sum formula: $C_{22}H_{22}IN_3O_6S_2Si$, M = 647.44, colourless block, 0.14x0.12x0.06 mm^3, triclinic space group P-1, a = 9.9333(4)Å, b = 10.6041(4)Å, c = 13.3328(6)Å, α = 90.119(3)°, β = 110.410(2)°, γ = 102.292(2)°, V = 1281.58(9) Å^3, Z = 2, Dc = 1.668 g/cm^3, F000 = 644, μ= 1.502 mm^{-1}, T = 170 K, θ_{max} = 27.102°, 10295 total reflections, 4501 with Io > 2σ(Io), R_{int} = 0.0331, 5638 data, 319 parameters, 0 restraints, GooF = 1.072, R = 0.0404 and wR= 0.0888 [Io > 2σ(Io)], R = 0.0579 and wR= 0.0948 (all reflections), 0.457<dΔρ<-0.606 /Å^3.

63) Crystal data for NHSac-**5** [CCDC-2297352]: Sum formula: $C_{12}H_9IN_2O_3S$, M = 388.17, colourless block, 0.031x0.02x0.02 mm^3, triclinic space group P-1, a = 7.4689(15)Å, b = 7.7102(15)Å, c = 13.155(3)Å, α = 91.06(3)°, β = 100.12(3)°, γ = 116.40(3)°, V = 663.9(3)Å^3, Z = 2, Dc = 1.942 g/cm^3, F000 = 376, μ= 2.573 mm^{-1}, T = 170 K, θ_{max} = 25.240°, 16896 total reflections, 2169 with Io > 2σ(Io), R_{int} = 0.0452, 2400 data, 172 parameters, 0 restraints, GooF = 1.088, R = 0.0333 and wR= 0.0733 [Io > 2σ(Io)], R = 0.0397 and wR= 0.0757 (all reflections), 1.405<dΔρ<-0.318 /Å^3.

64) Crystal data for NSac-**1**H [CCDC-2297353]: Sum formula: $C_{24}H_{20}N_4O_6S_2$, M = 524.56, colourless block, 0.023x0.02x0.019 mm^3, triclinic space group P-1, a = 7.3768(15)Å, b = 12.921(3)Å, c = 14.121(3)Å, α = 65.19(3)°, β = 74.93(3)°, γ = 78.26(3)°, V = 1172.8(5)Å^3, Z = 2, Dc = 1.485 g/cm^3, F000 = 544, μ= 0.277 mm^{-1}, T = 170 K, θ_{max} = 25.249°, 7622 total reflections, 2706 with Io > 2σ(Io), R_{int} = 0.0435, 4218 data, 326 parameters, 0 restraints, GooF = 1.027, R = 0.0610 and wR= 0.1245 [Io > 2σ(Io)], R = 0.1092 and wR= 0.1447 (all reflections), 0.312<dΔρ< -0.323 /Å^3.

65) Crystal data for NSac-**8**H [CCDC-2297355]: Sum formula: $C_{13}H_{12}N_2O_3S$, M = 276.31, colourless block, 0.117x0.093x0.052 mm^3, triclinic space group P-1, a = 8.5121(2)Å, b = 12.4435(2)Å, c = 13.9677(3)Å, α = 107.9831(19)°, β = 99.204(2)°, γ = 106.347(2)°, V = 1299.98(6)Å^3, Z = 4, Dc = 1.412 g/cm^3, F000 = 576, μ= 2.278 mm^{-1}, T = 120 K, θ_{max} = 79.678°, 16299 total reflections, 4870 with Io > 2σ(Io), R_{int} = 0.0273, 5412 data, 345 parameters, 0 restraints, GooF = 1.113, R = 0.0413 and wR= 0.1174 [Io > 2σ(Io)], R = 0.0448 and wR= 0.1197 (all reflections), 0.282<dΔρ<-0.579 /Å^3.

66) Crystal data for NSac-**16**H [CCDC-2297356]: Sum formula: $C_{14}H_{15}N_3O_3S$, M = 305.35, colourless block, 0.42x0.16x0.12 mm^3, monoclinic space group P2$_1$/c, a = 7.1692(2)Å, b = 8.3894(2)Å, c = 23.4609(6)Å, α = 90°, β = 97.158(2)°, γ = 90°, V = 1400.06(6)Å^3, Z = 4, Dc = 1.449 g/cm^3, F000 = 640, μ= 0.245 mm^{-1}, T = 170 K, θ_{max} = 27.876°, 6336 total reflections, 2582 with Io > 2σ(Io), R_{int} = 0.0402, 3331 data, 195 parameters, 1 restraints, GooF = 1.052, R = 0.0491 and wR= 0.1050 [Io > 2σ(Io)], R = 0.0711 and wR= 0.1156 (all reflections), 0.383 <dΔρ< -0.347 /Å^3.

67) Crystal data for NSac-**16**Ha [CCDC-2297354]: Sum formula: $C_{21}H_{20}N_4O_6S_2$, M = 488.53, colourless block, 0.5x0.28x0.04 mm^3, triclinic space group P-1, a = 7.1498(12)Å, b = 12.8796(19)Å, c = 13.280(2)Å, α = 110.086(8)°, β = 101.467(8)°, γ = 95.339(8)°, V = 1108.2(3)Å^3, Z = 2, Dc = 1.464g/cm^3, F000 = 508, μ= 0.287 mm^{-1}, T = 170 K, θ_{max} = 27.096°, 7913 total reflections, 3004 with Io > 2σ(Io), R_{int} = 0.0566, 4760 data, 303 parameters, 0 restraints, GooF = 1.078, R = 0.0754 and wR= 0.1930 [Io > 2σ(Io)], R = 0.1250 and wR= 0.2270 (all reflections), 0.624 <dΔρ<-0.546 /Å^3.

68) Crystal data for **16-I** [CCDC-2297358]: Sum formula: $C_7H_9IN_2$, M = 248.06, colourless block, 0.02x0.019x0.019 mm^3, monoclinic space group P2$_1$/c, a = 5.9023(12)Å, b = 8.4518(17)Å, c = 16.929(3)Å, α = 90°, β = 98.55(3)°, γ = 90°, V = 835.1(3)Å^3, Z = 4, Dc = 1.973g/cm^3, F000 = 472, μ= 3.762 mm^{-1}, T = 170 K, θ_{max} = 25.241°, 2794 total reflections, 1300 with Io > 2σ(Io), R_{int} = 0.0489, 1402 data, 93 parameters, 0 restraints, GooF = 1.064, R = 0.0420 and wR= 0.1150 [Io > 2σ(Io)], R = 0.0451 and wR= 0.1182 (all reflections), 1.177 <dΔρ<-0.990 /Å^3.

Table S1. Comparison of Solid-state X-ray crystal structure XB parameters and PBE0-D3/def2-TZVP gas-phase optimized parameters of N-iodosuccinimide complexes

**Compl**	X-ray		DFT		**I···N (Å)**		**N–I–N (°)**		**(C–C)–N–X (°)**		**Bending angle Δθ (°)**		**(imide)N···N(Py) (Å)**	
	**N–I (Å)[a]**	**Δ(N–I) (Å)[b]**	**N–I (Å)[c]**	**Δ(N–I) (Å)[b]**	X-ray	DFT	X-ray	DFT	X-ray	DFT	X-ray	DFT	X-ray	DFT
NIS-**1**[d]	2.101(3)	0.037	2.082	0.067	2.473(3)	2.520	180.0	180.0	180.0	180.0	0	0	4.574	4.602
	2.137(3)	0.073	--	--	2.443(3)	--	180.0	--	180.0		0	--	4.579	
NIS-**2**	2.082(4)	0.018	2.059	0.044	2.560(4)	2.638	174.55(15)	174.7	179.02	179.8	0.98	0.2	4.637	4.693
NIS-**3**	2.100(12)	0.037	2.062	0.047	2.580(12)	2.622	176.8(5)	174.6	176.19	179.9	3.81	0.1	4.68	4.679
NIS-**4**	2.095(2)	0.031	2.062	0.047	2.578(2)	2.625	176.97(9)	174.6	176.79	179.9	3.21	0.1	4.672	4.681
NIS-**5**	2.100(3)	0.039	2.064	0.049	2.549(2)	2.611	177.33(10)	175.3	177.17	180.0	0	0	4.648	4.671
NIS-**6**	2.094(3)	0.03	2.053	0.038	2.570(3)	2.694	177.95(10)	172.5	179.33	179.7	0.67	0.3	4.670	4.737
NIS-**7**	-	--	2.056	0.041	-	2.647	-	177.0	-	179.5	--	0.5	-	4.701
NIS-**8**	2.112(4)	0.048	2.085	0.07	2.481(4)	2.523	175.44(17)	177.5	179.59	179.9	0.41	0.1	4.588	4.607
NIS-**9**	2.123(2)	0.059	2.084	0.069	2.455(2)	2.531	176.59(8)	177.3	176.59	179.8	0.41	0.2	4.577	4.614
NIS-**10**	-	--	2.083	0.068	-	2.538	-	176.9	-	179.8	--	0.2	-	4.619
NIS-**11**[e]	2.135(4)	0.071	2.077	0.062	2.422(4)	2.566	174.30(15)	175.4	176.96	179.9	3.04	0.1	4.552	4.639
	2.050(4)	-0.014	--		2.654(4)	--	170.96(12)	--	--	--	--	--	4.682 (N···O)[b]	--
NIS-**12**	2.114(2)	0.05	2.072	0.057	2.499(2)	2.595	175.92(7)	175.9	178.54	179.6	1.46	0.4	4.61	4.664
NIS-**13**	--	--	2.086	0.071	--	2.522	--	179.9	--	178.9	--	1.1	--	4.608
NIS-**14**	--	--	2.067	0.052	--	2.637	--	175.4	--	179.3	--	0.7	--	4.700
NIS-**15**	--	--	2.088	0.073	--	2.514	--	178.2	--	179.2	--	0.8	--	4.601
NIS-**16**	--	--	2.072	0.057	--	2.593	--	177.2	--	179.5	--	0.5	--	4.663

[a] Crystal structure $(N–I)_{ligand}$ = 2.064(6) Å; [b] $Δ(N–I) = (N–I)_{compl} – (N–I)_{ligand}$; [c] DFT structure $(N–I)_{ligand}$ = 2.015 Å; [d] The asymmetric unit contains two different NIS-**1** complexes. [e] Two XB donors are present in the asymmetric unit, one of which participates in XB with imide C=O group of the 1:1 NIS:**11** halogen-bonded complex.

Table S2. Comparison of Solid-state X-ray crystal structure XB parameters and PBE0-D3/def2-TZVP gas-phase optimized parameters of N-iodophthalimide complexes

Compl	X-ray		DFT		I···N (Å)		N–I–N (°)		(C–C)–N–X (°)		Bending angle Δθ (°)		(imide)N···N(Py) (Å)	
	N–I (Å)[a]	Δ(N–I) (Å)[b]	N–I (Å)[c]	Δ(N–I) (Å)[b]	X-ray	DFT	X-ray	DFT	X-ray	DFT	X-ray	DFT	X-ray	DFT
NIP-**1**	2.130(3)	0.093	2.077	0.07	2.409(3)	2.518	177.68(10)	180.0	177.28	180.0	2.72	0	4.538	4.595
NIP-**2**	2.097(4)	0.06	2.054	0.047	2.509(8)	2.636	177.3(5)	174.8	175.94	179.9	4.06	0.1	4.632	4.685
NIP-**3**	2.0833(15)	0.0463	2.057	0.05	2.553(2)	2.620	176.56(5)	174.7	165.21	179.9	14.79	0.1	4.634	4.672
NIP-**4**[d]	2.078(3)	0.041	2.056	0.049	2.547(3)	2.622	177.52(10)	174.7	170.05	179.9	9.95	0.1	4.625	4.673
	2.089(3)	0.052	--	---	2.555(3)	--	177.13(10)	--	168.3	--	11.7	--	4.642	--
NIP-**5**	2.081(3)	0.044	2.058	0.051	2.545(4)	2.608	176.47(13)	175.5	177.29	180.0	2.71	0	4.624	4.663
NIP-**6**	2.076(3)	0.039	2.047	0.04	2.614(4)	2.692	176.74(11)	172.7	177.06	179.7	2.94	0.3	4.688	4.729
NIP-**7**	--	--	2.050	0.043	-	2.645	--	176.8	-	179.6	--	0.4	--	4.693
NIP-**8**	2.124(12)	0.087	2.080	0.073	2.490(13)	2.520	172.2(6)	177.5	172.16	179.8	7.84	0.2	4.611	4.599
NIP-**9**	2.122(2)	0.085	2.079	0.072	2.449(2)	2.529	175.22(9)	177.4	176.9	179.8	3.1	0.2	4.567	4.607
NIP-**10**	--	--	2.078	0.071	-	2.536	--	176.9	-	179.8	--	0.2	--	4.612
NIP-**11**	--	--	2.072	0.065	-	2.563	--	175.4	-	179.9	--	0.1	--	4.631
NIP-**12**	2.083(4)	0.046	2.067	0.06	2.526(4)	2.592	175.04(14)	175.8	177.93	179.6	3.1	0.4	4.605	4.656
NIP-**13**	2.1038(16)	0.0668	2.081	0.074	2.4980(16)	2.521	177.68(6)	179.9	173.81	178.9	6.19	1.1	4.601	4.601
NIP-**14**	2.091(3)	0.054	2.061	0.054	2.574(3)	2.635	170.05(12)	175.4	170.03	179.0	9.97	1.0	4.647	4.693
NIP-**15**	--	--	2.082	0.075	--	2.515	--	178.0	--	179.2	--	0.8	--	4.596
NIP-**16**	--	--	2.067	0.06	--	2.592	--	177.0	--	179.5	--	0.5	--	4.657

[a]$(N–I)_{ligand}$ = 2.037(3) Å; [b]Δ(N–I) = $(N–I)_{compl}$ – $(N–I)_{ligand}$; [c]DFT structure $(N–I)_{ligand}$ = 2.007 Å; [d]The asymmetric unit contains two different NIS-**4** complexes.

Table S3. Comparison of Solid-state X-ray crystal structure XB parameters and PBE0-D3/def2-TZVP gas-phase optimized parameters of N-iodosaccharin complexes

**Compl**	X-ray		DFT		**I···N (Å)**		**N–I–N (°)**		**(C–C)–N–X (°)**		**Bending angle Δθ (°)**		**(imide)N···N(Py) (Å)**	
	**N–I (Å)[a]**	**Δ(N–I) (Å)[b]**	**N-I (Å)[c]**	**Δ(N–I) (Å)[b]**	X-ray	DFT	X-ray	DFT	X-ray	DFT	X-ray	DFT	X-ray	DFT
NISac-**1**	2.254(11)	0.188	2.102	0.094	2.279(11)	2.447	174.5(4)	180.0	167.41	177.6	12.59	2.4	4.527	4.549
NISac-**2**	2.105(5)	0.039	2.073	0.065	2.492(6)	2.557	172.8(2)	175.4	173.85	177.4	6.15	2.6	4.589	4.626
NISac-**3**	2.140(14)	0.074	2.077	0.069	2.455(15)	2.542	172.8(6)	175.2	175.62	177.5	4.38	2.5	4.581	4.614
NISac-**4**	2.123(3)	0.057	2.076	0.068	2.441(3)	2.544	176.35(10)	175.2	176.34	177.6	3.66	2.4	4.561	4.616
NISac-**5**	2.168(3)	0.1	2.079	0.071	2.376(3)	2.529	178.67(10)	176.0	163.93	177.7		2.3	4.544	4.606
NISac-**6**	2.099(6)	0.033	2.064	0.056	2.528(6)	2.609	172.9(3)	173.3	166.01	177.3	16.07	2.7	4.618	4.665
NISac-**7**	2.131(4)	0.065	2.068	0.06	2.440(4)	2.562	175.4(2)	175.2	177.62	177.1	2.38	2.9	4.568	4.627
NISac-**8**	-		2.106	0.098	-	2.447	-	177.7	-	177.8	--	2.2	-	4.553
NISac-**9**	2.231(19)	0.165	2.107	0.099	2.325(18)	2.449	175.4(7)	177.3	177.9	177.9	2.1	2.1	4.544	4.555
$(NSac)_2$I-**10**H	2.218(4)	0.152	2.104	0.096	2.280(4)	2.461	179.21(13)	177.2	169.48<br>169.23	178.0	10.52<br>10.77	2	4.497	4.564
NISac-**11**	2.211(11)	0.145	2.097	0.089	2.342(11)	2.485	178.8(4)	175.9	163.06	177.7	16.94	2.3	4.553	4.579
$(NSac)_2$I-**12**H	2.2639(19)	0.198	2.092	0.084	2.2639(19)	2.504	180.0	176.5	168.76	177.1	11.24	2.9	4.528	4.594
NISac-**13**	-	--	2.110	0.102	-	2.445	-	179.3	-	174.3	--	5.7	-	4.555
NISac-**14**	-	--	2.086	0.078	-	2.553	-	175.5	-	176.5	--	3.5	-	4.636
$(NSac)_2$I-**15**H[c]	2.274(5)	0.208	2.115	0.107	2.214(5)	2.419	178.89(18)	179.8	171.83<br>169.67	179.3	8.17<br>10.33	0.7	4.487	4.534
$(NSac)_2$I-**15**Ha[c]	2.283(3)	0.217	--	--	2.221(3)	-	176.97(12)	-	176.71<br>171.78	-	3.19<br>8.22	--	4.503	-
NISac-**16**	-	--	2.093	0.085	-	2.502	-	177.4	-	176.9	--	3.1	-	4.593

[a]$(N–I)_{ligand}$ = 2.066(4) Å; [b]Δ(N–I) = $(N–I)_{compl}$ – $(N–I)_{ligand}$; [c]DFT structure $(N–I)_{ligand}$ = 2.008 Å; [d]Two different XB complexes were obtained for $(NISac)_2$I-**15H**.

Table S4. Comparison of Solid-state X-ray crystal structure XB parameters and PBE0-D3/def2-TZVP/PCM[a] optimized parameters and free energies of complexation of N-iodosaccharin complexes in $CHCl_3$.

Compl	N-I (Å)		I···N (Å)		N–I–N (°)		(C–C)–N–X (°)		Bending angle Δθ (°)		(imide)N···N(Py) (Å)		$\Delta G_{complex}$(XB) (kJ mol^{-1})
	X-ray	DFT	X-ray	DFT	X-ray	DFT	X-ray	DFT	X-ray	DFT	X-ray	DFT	DFT
NISac-**1**	2.254(11)	2.154	2.279(11)	2.349	174.5(4)	179.9	167.41	177.8	12.59	2.2	4.527	4.503	-33.2
NISac-**2**	2.105(5)	2.110	2.492(6)	2.453	172.8(2)	176.6	173.85	177.7	6.15	2.3	4.589	4.561	-10.8
NISac-**3**	2.140(14)	2.114	2.455(15)	2.445	172.8(6)	176.3	175.62	177.9	4.38	2.1	4.581	4.556	-13.2
NISac-**4**	2.123(3)	2.112	2.441(3)	2.449	176.35(10)	176.3	176.34	177.9	3.66	2.1	4.561	4.559	-11.7
NISac-**5**	2.166(3)	2.116	2.378(3)	2.438	178.67(10)	176.9	163.93	178.0		2.0	4.544	4.552	-14.7
NISac-**6**	2.099(6)	2.095	2.528(6)	2.510	172.9(3)	175.2	166.01	177.7	16.07	2.3	4.618	4.601	-6.3
NISac-**7**	2.131(4)	2.105	2.440(4)	2.457	175.4(2)	177.6	177.62	177.7	2.38	2.3	4.568	4.561	-7.5
NISac-**8**	-	2.158	-	2.355	-	177.8	-	178.1	--	1.9	-	4.513	-31.1
NISac-**9**	2.231(19)	2.159	2.325(18)	2.358	175.4(7)	177.3	177.9	178.1	2.1	1.9	4.544	4.516	-31.8
NISac-**11**	2.211(11)	2.153	2.342(11)	2.373	178.8(4)	177.2	163.06	178.1	16.94	1.9	4.553	4.525	-29.9
NISac-**13**	--	2.147	-	2.380	-	176.3	-	177.9	--	2.1	-	4.525	-15.2
NISac-**14**	--	2.135	-	2.408	-	176.7	-	178.3	--	1.7	-	4.542	-20.8
NISac-**16**	--	2.149	-	2.376	-	178.7	-	176.9	--	3.1	-	4.525	-28.8

[a] Polarized continuum model was used as implemented in Gaussian16. See References 15-18.

Table S5. Comparison of Solid-state X-ray crystal structure XB parameters and PBE0-D3/def2-TZVP optimized parameters of N-bromosuccinimide complexes

Comp	X-ray		DFT		Br···N (Å)		N–Br–N (°)		(C-C)–N–Br (°)		Bending angle Δθ (°)		(imide)N···N(Py) (Å)	
	N–Br (Å)[a]	Δ(N–Br) (Å)[b]	N–Br (Å)[c]	Δ(N–Br) (Å)[b]	X-ray	DFT	X-ray	DFT	X-ray	DFT	X-ray	DFT	X-ray	DFT
NBS-**1**[d]	1.8923(19)	0.0523	1.873	0.047	2.444(2)	2.507	177.44(9)	180.0	178.74	180.0	1.26	0	4.335	4.380
	1.8876(19)	0.0476	--	--	2.499(2)		175.38(8)		179.26	--	0.74	--	4.383	
	1.8997(19)	0.0597	--	--	2.424(2)		179.45(8)		178.29	--	1.71	--	4.324	
	1.9064(19)	0.0664	--	--	2.417(2)		179.96(9)		176.56	--	3.44	--	4.321	
NBS-**2**	-	--	1.852	0.026	--	2.645	--	173.1	--	179.7	--	0.3	--	4.489
NBS-**3**	1.8691(17)	0.0291	1.855	0.029	2.557(2)	2.626	178.47(6)	173.6	175.53	179.7	4.47	0.3	4.426	4.474
NBS-**4**	1.876(6)	0.036	1.854	0.028	2.545(7)	2.629	179.7(3)	173.6	175.91	179.7	4.09	0.3	4.427	4.477
NBS-**5**	1.880(6)	0.04	1.856	0.03	2.503(7)	2.611	178.5(2)	174.5	177.04	179.8	2.96	0.2	4.384	4.462
NBS-**8**	1.898(3)	0.058	1.876	0.05	2.430(3)	2.500	175.92(11)	177.8	179.67	179.9	0.33	0.1	4.326	4.375
NBS-**9**	1.901(4)	0.061	1.876	0.05	2.476(4)	2.507	174.78(15)	177.9	175.72	179.8	4.28	0.2	4.373	4.381
NBS-**11**	-	--	1.865	0.039	--	2.566	--	175.0	--	179.9	--	0.1	--	4.427
NBS-**12**	-	--	1.862	0.036	--	2.588	--	176.5	--	179.6	--	0.4	--	4.449

[a]$(N–Br)_{ligand}$ = 1.840(6) Å; [b]Δ(N–Br) = $(N–Br)_{compl}$ – $(N–Br)_{ligand}$; [c]DFT structure $(N–Br)_{ligand}$ = 1.826 Å; [d]The asymmetric unit contains four crystallographically independent 1:1 XB complexes;

Table S6. Comparison of Solid-state X-ray crystal structure XB parameters and PBE0-D3/def2-TZVP optimized parameters of N-bromophthalimide complexes

Comp	X-ray		DFT		Br···N (Å)		N–Br–N (°)		(C–C)–N–Br (°)		Bending angle Δθ (°)		(imide)N···N(Py) (Å)	
	N–Br (Å)[a]	Δ(N–Br) (Å)[b]	N-Br (Å)[c]	Δ(N–Br) (Å)[b]	X-ray	DFT	X-ray	DFT	X-ray	DFT	X-ray	DFT	X-ray	DFT
NBP-**1**	1.924(3)	0.083	1.868	0.048	2.329(3)	2.505	177.51(13)	180.0	178.53	180.0	1.47	0	4.252	4.373
NBP-**2**	1.859(5)	0.018	1.848	0.028	2.607(3)	2.644	168.94(10)	173.4	172.75	179.7	7.25	0.3	4.44	4.484
NBP-**3**[d]	1.867(2)	0.026	1.850	0.03	2.513(3)	2.625	178.06(10)	173.9	171.3	179.7	7.25	0.3	4.38	4.469
	1.877(2)	0.036	--	--	2.544(2)		177.70(9)	--	169.81	--	10.19	--	4.42	
NBP-**4**[d]	1.872(7)	0.031	1.850	0.03	2.506(7)	2.628	177.9(3)	173.8	169.9	179.7	10.1	0.3	4.379	4.471
	1.878(7)	0.037	--	--	2.558(7)		177.4(3)		170.18	--	9.82	--	4.436	
NBP-**5**	1.883(3)	0.042	1.852	0.032	2.491(3)	2.609	178.27(14)	174.7	173.02	179.8	6.28	0.2	4.373	4.456
NBP-**6**	1.8659(18)	0.0249	1.845	0.025	2.631(2)	2.673	169.92(11)	171.4	169.38	179.6	10.62	0.4	4.48	4.506
NBP-**7**	1.872(7)	0.031	1.846	0.026	2.558(7)	2.655	175.12(18)	176.8	166.73	179.6	13.27	0.4	4.431	4.499
NBP-**8**[d]	1.913(2)	0.072	1.872	0.052	2.391(2)	2.498	178.97(9)	177.8	178.78	179.8	1.22	0.2	4.304	4.369
	1.919(2)	0.078	--	--	2.380(3)		177.88(10)		174.28	--	5.72	--	4.298	
NBP-**9**	1.918(3)	0.077	1.871	0.051	2.408(4)	2.505	176.74(12)	177.8	174.89	179.8	5.11	0.2	4.325	4.376
NBP-**10**[d]	1.894(4)	0.053	1.871	0.051	2.448(5)	2.511	173.41(17)	177.4	174.97	179.8	5.03	0.2	4.332	4.381
	1.902(4)	0.061	---	--	2.457(5)		174.02(17)		174.09	--	5.91	--	4.352	
NBP-**11**	1.908(4)	0.067	1.861	0.041	2.399(2)	2.563	175.03(13)	175.1	178.22	179.8	1.78	0.2	4.303	4.420
NBP-**12**	1.880(2)	0.039	1.858	0.038	2.516(2)	2.588	174.52(10)	176.3	178.85	179.6	1.15	0.4	4.392	4.443
NBP-**13**	1.892(3)	0.051	1.871	0.051	2.435(3)	2.504	176.65(10)	179.8	173.66	179.2	6.34	0.8	4.325	4.375

[a]$(N–Br)_{ligand}$ = 1.841(5) Å; [b]$\Delta(N–I) = (N–I)_{compl} – (N–I)_{ligand}$; [c]DFT structure $(N–Br)_{ligand}$ = 1.820 Å; [d]The asymmetric unit contains two crystallographically independent 1:1 XB complexes.

Table S7. Comparison of Solid-state X-ray crystal structure XB parameters and PBE0-D3/def2-TZVP optimized parameters of N-bromophthalimide complexes

Comp	X-ray		DFT		Br···N (Å)		N-Br-N (°)		(C–C)–N–Br (°)		Bending angle Δθ (°)		(imide)N···N(Py) (Å)	
	N–Br (Å)[a]	Δ(N–Br) (Å)[b]	N–Br (Å)[c]	Δ(N–Br) (Å)[b]	X-ray	DFT	X-ray	DFT	X-ray	DFT	X-ray	DFT	X-ray	DFT
NBSac-**1**	--	--	1.894	0.072	-	2.401	-	179.7	-	178.2	--	1.8	--	4.295
NBSac-**2**	1.882(4)	0.055	1.862	0.04	2.442(3)	2.555	177.5(2)	174.5	176.79	177.9	3.21	2.1	4.323	4.413
NBSac-**3**	1.9230(19)	0.096	1.866	0.044	2.401(2)	2.530	174.90(8)	174.7	158.26	178.0	21.74	2	4.319	4.392
NBSac-**4**	1.913(7)	0.086	1.866	0.044	2.395(6)	2.529	175.1(4)	174.7	158.63	178.0	21.37	2	4.303	4.391
NBSac-**5**[d]	1.920(4)	0.093	1.870	0.048	2.392(4)	2.506	175.1(2)	176.1	159.52	178.5	20.48	1.5	4.308	4.374
NBSac-**5a**[d]	1.952(7)	0.125	--	--	2.266(7)	--	178.4(3)	--	164.93	--	15.07	--	4.218	
NBSac-**6**	1.893(3)	0.066	1.858	0.036	2.463(3)	2.591	172.96(19)	172.5	164.15	177.9	15.85	2.1	4.349	4.439
NBSac-**7**	--	--	1.858	0.036	2.438	2.572	175.48	173.6	177.04	177.6	2.96	2.4	4.564	4.423
NBSac-**9**	1.988(2)	0.161	1.902	0.08	2.266(2)	2.385	173.09(10)	177.6	155.73	178.5	24.27	1.5	4.246	4.287
NBSac-**13**	1.938(2)	0.111	1.904	0.082	2.316(2)	2.387	174.28(9)	179.2	167.9	172.7	12.1	7.3	4.249	4.291
NBSac-**14**	1.916(5)	0.089	1.876	0.054	2.474(5)	2.517	169.60(16)	174.7	175.25	174.9	4.75	5.1	4.372	4.389
NBSac-**15**	--	--	1.917	0.095	-	2.332	-	179.6	-	179.6	--	0.4	--	4.250
NBSac-**16**	--	--	1.883	0.061	-	2.466	-	177.9	-	177.4	--	2.6	--	4.349

[a]$(N–Br)_{ligand}$ = 1.827(4) Å; [b]Δ(N–Br) = $(N–Br)_{compl}$ – $(N–Br)_{ligand}$; [c]DFT structure $(N–Br)_{ligand}$ = 1.822 Å; [d]Two different XB complexes were obtained for NISac-**5**.

Table S8. Literature X-ray crystal structure XB parameters of N-haloimide complexes

CSD Code	Donor	N–X (Å)[a]	X···N (Å)	N–X–N (°)	(C–C)–N–X (°)	bending angle (°)	(imide)N···N(Py) (Å)
SECYIL	NBS	1.898	2.439	176.24	178.8	1.2	4.334
SECYOR	NBS	1.885	2.501	174.22	179.11	0.89	4.381
SECYUX	NBS	1.897	2.418	175	179.28	0.72	4.31
SECZAE	NBS	1.844	2.558	171.72	176.28	3.72	4.39
SECZEI	NBS	1.873	2.491	176.07	179.29	0.71	4.362
SECZIM	NBS	1.868	2.597	176.27	176.49	3.51	4.462
MUPFUB	NBS	1.932	2.319	179.96	177.8	2.2	4.251
DEGTIW	NBS	1.885	2.471	176.36	177.06	2.94	4.354
VELVIU	NBP	1.88	2.494	174.58	171.92	8.08	4.369
VELVOA	NBP	1.906	2.425	179.26	178.1	1.9	4.331
		1.904	2.404	178.34	175.13	4.87	4.308
VELVUG	NBP	1.898	2.449	179.92	179.45	0.55	4.347
VELWAN	NBP	1.87	2.487	178.28	179.02	0.98	4.356
VELWER	NBP	1.888	2.454	175.42	171.22	8.78	4.338
VELWUH	NBP	1.894	2.476	179.2	178.14	1.86	4.37
VELXES	NBP	1.885	2.454	175.96	175.96	4.04	4.336
VELXIW	NBP	1.91	2.38	178.6	179.29	0.71	4.294
KECJAG	NBSac	2.0727	2.098	178.77	177.76	2.24	4.171
KUWHAN	NIS	2.116	2,484	180	180	0	4.599
		2.142	2.428	180	180	0	4.57
KUWHER	NIS	2.146	2.407	178.94	176,85	3.15	4.553
OGIWOS	NIS	2.117	2.493	180	180	0	4.609
		2.144	2.429	180	180	0	4.574
SECXEG	NIS	2.12	2.412	177.16	173.27	6.73	4.532
		2.145	2.408	177.67	178.85	1.15	4.552
SECXOQ	NIS	2.1	2.5	173.88	178.04	1.96	4.594
SECYAD	NIS	2.109	2.474	176.1	178.29	1.71	4.584
DECGUQ	NIS	2.086	2.502	175.86	178.96	1.04	4.585
DEGTAO	NIS	2.103	2.539	177.44	178.39	1.61	4.641
DEGTES	NIS	2.092	2.498	176.71	176.74	3.26	4.589
KUWHIV	NISac	2.22	2.305	178.18	174.19	5.81	4.523
KUWHUH	NISac	2.293	2.28	178.84	173.17	6.83	4.521
MUGDAU	NISac	2.254	2.279	174.5	167.41	12.59	4.527
OGIWUY	NISac	2.292	2.218	178.56	172.49	7.51	4.509

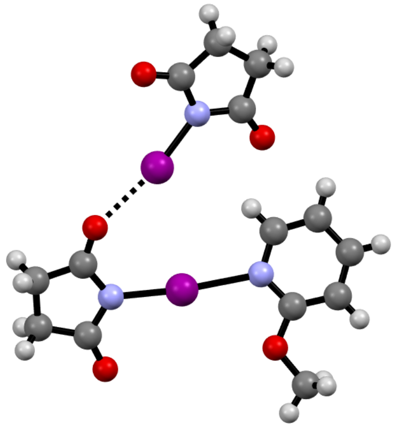

Figure S2. X-ray crystal structure of NIS-**11.** Two XB donors are present in the asymmetric unit, one of which participates in XB with imide C=O group of the NIS-**11** halogen-bonded complex.

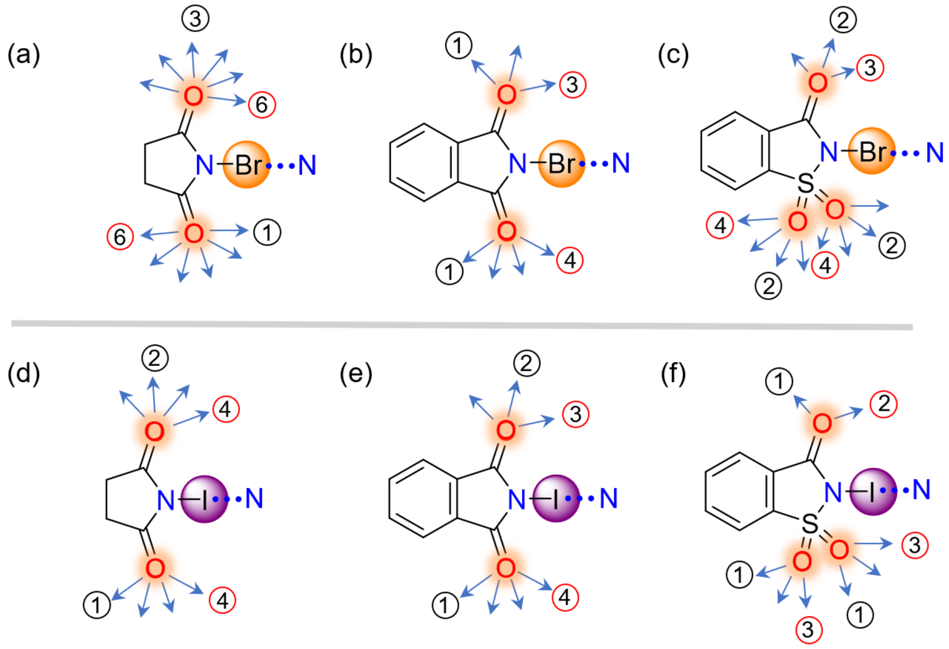

Figure S3. Polydentate coordination nature of XB donors in halogen-bonded complexes. The numbers inside black cirlces represent minimum denticity whereas those inside red circles represent maximum denticity. For instance, as seen in Figure S3d, the iodosuccinimide's first oxygen displays bidentate (minimum) and tetradentate (maximum) coordination modes, whereas the second oxygen displays monodentate (minimum) and tetradentate (maximum) coordination modes.

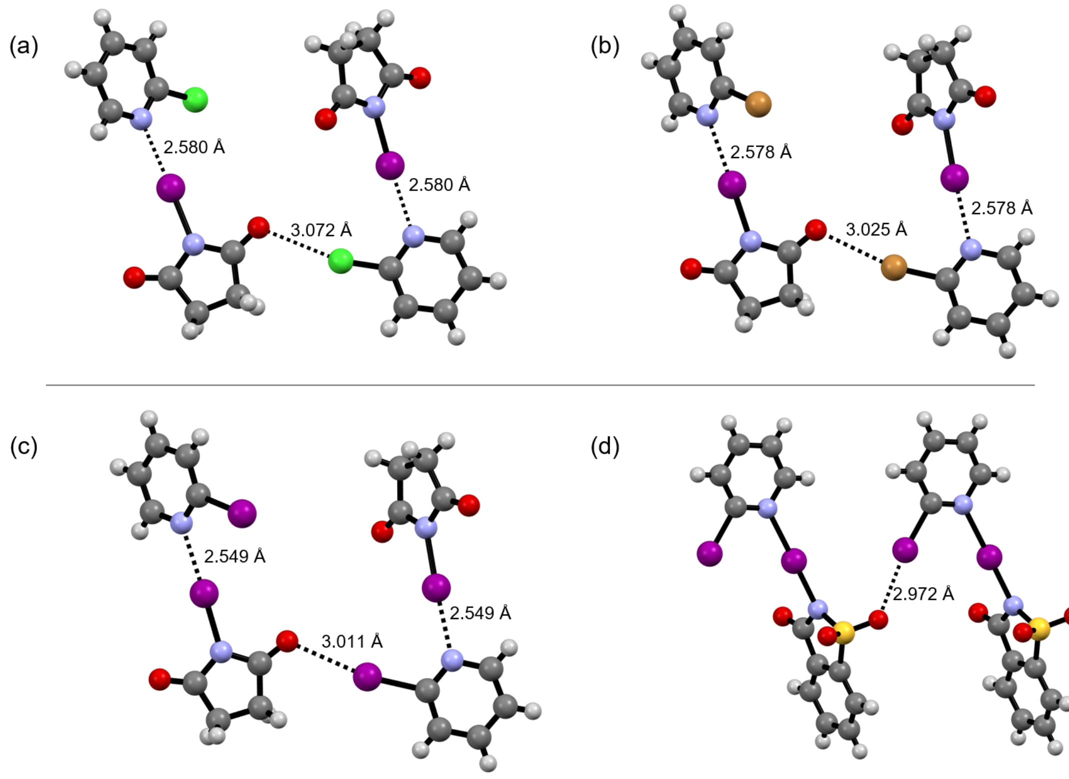

Figure S4. X-ray crystal structure of (a) NIS-**3**, (b) NIS-**4**, (c) NIS-**5**, and (d) NISac-**5** in ball and stick model displaying the C–X⋯O=C halogen bonding interactions

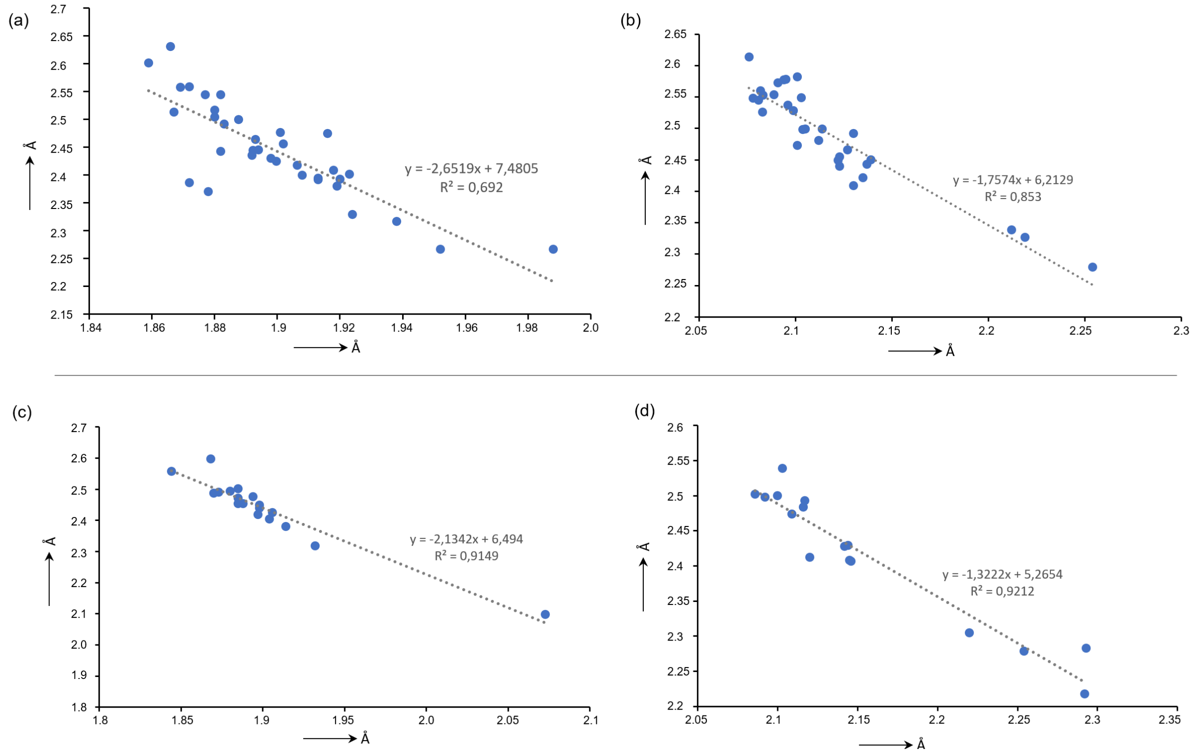

Figure S5. Correlation between N–X and X⋯N distances of (a) bromoimide-2-*ortho*-substituted pyridine complexes (b) iodoimide-2-*ortho*-substituted pyridine complexes and (c) bromoimide-2-*meta*/*para*-substituted pyridine complexes and (d) iodoimide-2-*meta*/*para*-substituted pyridine complexes.

3. Computational results

Optimized geometries and harmonic vibrational frequencies of all structures were derived by DFT calculations performed with PBE0 hybrid functional[10] employing def2-TZVP basis sets.[11] Dispersion interactions in the complex structures were treated with Grimme's empirical D3 correction version that used Becke-Johnson dampening.[12] Complexation energies corrected for basis set superposition errors were derived from counterpoise calculations.[13] Effect of solvent to XB structures in $CHCl_3$ solutions was modelled using polarized continuum model (PCM) calculations.[15-18] All calculations were run on Gaussian 16 program package.[14]

Table S9. Computed (PBE0-D3/def2-TZVP) halogen bonding interaction energies ΔE_{XB} [kJ mol^{-1}].

Compl	ΔE_{XB}	Compl	ΔE_{XB}	Compl	ΔE_{XB}	Compl	ΔE_{XB}	Compl	ΔE_{XB}	Compl	ΔE_{XB}
NIS-**1**	-65.5	NIP-**1**	-65.1	NISac-**1**	-81.3	NBSac-**1**	-56.9	NBP-**1**	-43.1	NBS-**1**	-43.1
NIS-**2**	-47.7	NIP-**2**	-47.3	NISac-**2**	-58.7	NBSac-**2**	-38.4	NBP-**2**	-30.4	NBS-**2**	-30.5
NIS-**3**	-51.1	NIP-**3**	-50.6	NISac-**3**	-62.8	NBSac-**3**	-41.8	NBP-**3**	-33.0	NBS-**3**	-33.1
NIS-**4**	-50.8	NIP-**4**	-50.2	NISac-**4**	-62.3	NBSac-**4**	-41.5	NBP-**4**	-32.8	NBS-**4**	-32.9
NIS-**5**	-52.5	NIP-**5**	-51.9	NISac-**5**	-64.6	NBSac-**5**	-43.5	NBP-**5**	-34.1	NBS-**5**	-34.2
NIS-**6**	-44.9	NIP-**6**	-44.4	NISac-**6**	-54.2	NBSac-**6**	-37.2	NBP-**6**	-30.1	NBS-**8**	-47.2
NIS-**7**	-44.4	NIP-**7**	-43.8	NISac-**7**	-53.8	NBSac-**7**	-35.1	NBP-**7**	-28.3	NBS-**9**	-48.4
NIS-**8**	-70.0	NIP-**8**	-69.5	NISac-**8**	-87.0	NBSac-**8**	-62.8	NBP-**8**	-47.1	NBS-**11**	-40.4
NIS-**9**	-71.3	NIP-**9**	-70.5	NISac-**9**	-88.1	NBSac-**9**	-63.8	NBP-**9**	-48.2	NBS-**12**	-42.0
NIS-**10**	-71.3	NIP-**10**	-70.5	NISac-**10**	-88.0	NBSac-**10**	-64.1	NBP-**10**	-48.4		
NIS-**11**	-63.1	NIP-**11**	-62.7	NISac-**11**	-79.3	NBSac-**11**	-53.3	NBP-**11**	-40.4		
NIS-**12**	-63.1	NIP-**12**	-62.3	NISac-**12**	-79.4	NBSac-**12**	-55.3	NBP-**12**	-41.8		
NIS-**13**	-78.9	NIP-**13**	-77.7	NISac-**13**	-98.0	NBSac-**13**	-73.1	NBP-**13**	-55.1		
NIS-**14**	-68.0	NIP-**14**	-67.7	NISac-**14**	-86.5	NBSac-**14**	-62.3				
NIS-**15**	-77.3	NIP-**15**	-76.1	NISac-**15**	-99.3	NBSac-**15**	-77.1				
NIS-**16**	-63.0	NIP-**16**	-62.2	NISac-**16**	-79.5	NBSac-**16**	-55.1				

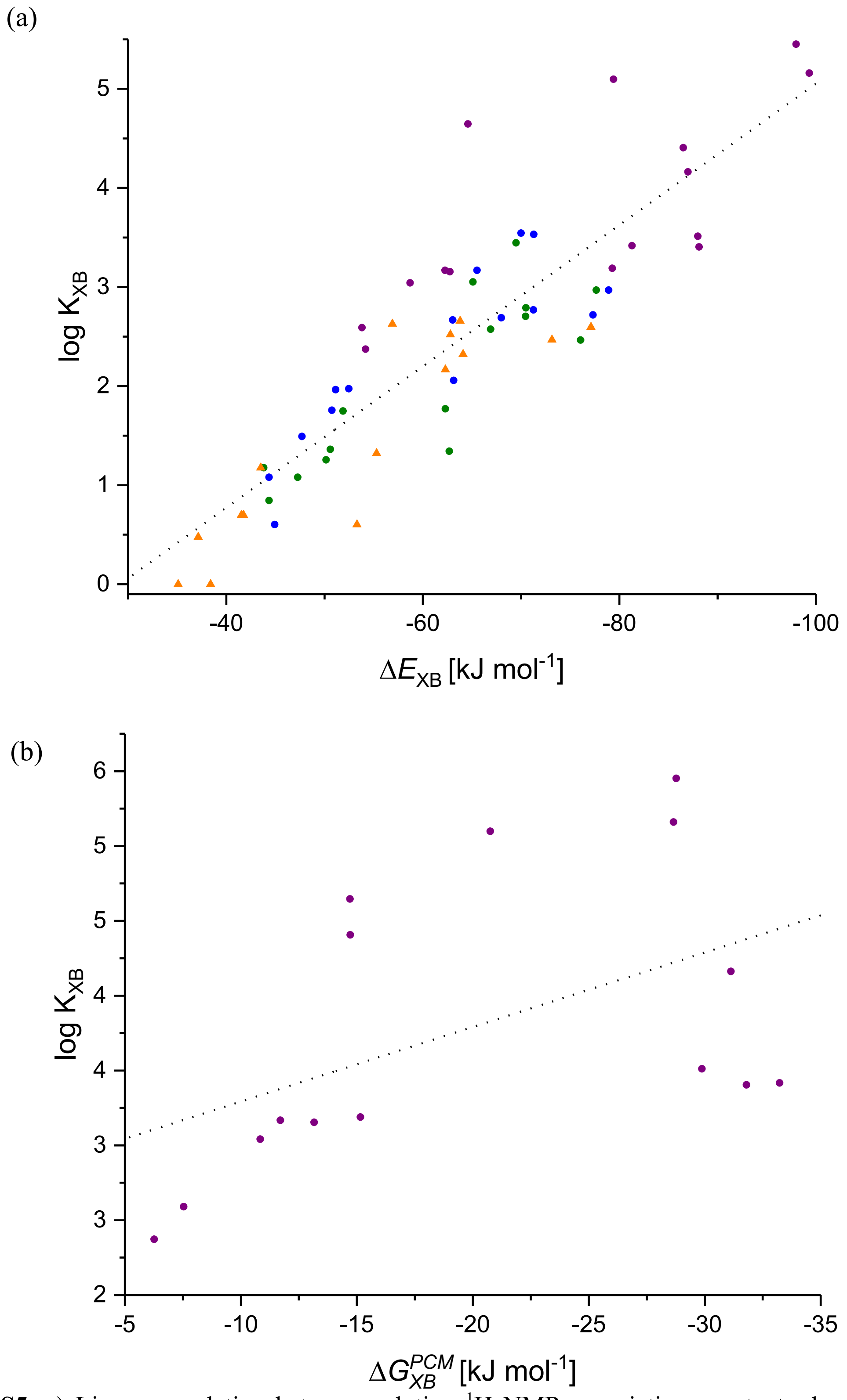

Figure S5. a) Linear correlation between solution ^{1}H NMR association constants, log K_{XB} and a) calculated complexation energies, ΔE_{XB} (R^2 = 0.760) for 60 XB complexes of NIS-**Z** (blue dots), NIP-**Z** (green dots), NISac-**Z** (purple dots) and NBSac-**Z** (orange triangles), b) calculated complexation free energies ΔG_{XB}^{PCM} (R^2 = 0.189) for fifteen NISac-**Z** complexes in $CHCl_3$ solution. Calculated values have been determined with PBE0-D3/def2-TZVP method.

4. Solution NMR

All the ^{15}N NMR spectra were obtained by mixing 1:1 molar ratio of respective XB donor and acceptor molecules in $CDCl_3$ and were recorded on a Bruker Avance III 500 MHz spectrometer equipped with prodigy probe. External ^{15}N reference used was 90% ^{15}N enriched nitromethane in a sealed tube. The ^{15}N spectra could be measured with one scan/increment. The signal was calibrated to 0 ppm, and the other samples were measured using the same parameters. Only the number of scans and the receiver gain were altered. When the sealed internal reference tube was measured inside the sample, the signal from the enriched reference was too strong, while the signal from the sample was too weak. Sine bell window function with zero shifts was used for apodization before Fourier transformation, and all the measurements were made either with 64 or 128 scans. The HMBC ^{1}H-^{15}N chemical shift changes for NIP, NBP, NISac and NBSac could not be obtained.

The titrations were performed in $CDCl_3$ at the temperatures. In an NMR tube, a 10 mM sample solution (volume 600 μL) of N-haloimide were prepared, and all titrations were performed with at least 20 data points up to 4.5-5.0 equivalents of the guest added, using 0.15 M stock solutions of pyridines dissolved in $CDCl_3$. Titration experiments were conducted using Bruker Avance III 500 MHz spectrometer. Titration data was fitted into a 1:1 binding model using online Bindfit software.[19] The association constants are summarized in Table S11. The chemical shifts of protons in donor were used to calculate the binding constants.

Table S10. Solution ^{15}N NMR of N-haloimide XB complexes ($CDCl_3$, 298.0K).

**Comp**	**$\Delta\delta N_{coord}{}^{a}$**	**Comp**	**$\Delta\delta N_{coord}{}^{a}$**	**Comp**	**$\Delta\delta N_{coord}{}^{a}$**	**Comp**	**$\Delta\delta N_{coord}{}^{a}$**	**Comp**	**$\Delta\delta N_{coord}{}^{a}$**	**Comp**	**$\Delta\delta N_{coord}{}^{a}$**
NIS-**1**	-41.5	NIP-**1**	-0.7	NISac-**1**	-63.1	NBS-**1**	-2.0	NBP-**1**	-0.7	NBSac-**1**	-23.2
NIS-**2**	-13.5	NIP-**2**	-2.6	NISac-**2**	-33.7	NBS-**2**	-1.0	NBP-**2**	-2.6	NBSac-**2**	-2.5
NIS-**3**	-16.9	NIP-**3**	-1.6	NISac-**3**	-40.8	NBS-**3**	-2.5	NBP-**3**	-1.6	NBSac-**3**	-3.7
NIS-**4**	-15.3	NIP-**4**	-2.5	NISac-**4**	-41.4	NBS-**4**	-1.6	NBP-**4**	-2.5	NBSac-**4**	-3.8
NIS-**5**	-20.2	NIP-**5**	-2.2	NISac-**5**	-46.6	NBS-**5**	-2.9	NBP-**5**	-2.2	NBSac-**5**	-8.6
NIS-**6**	-5.9	NIP-**6**	-2.4	NISac-**6**	-21.6	NBS-**6**	-5.9	NBP-**6**	-2.4	NBSac-**6**	-0.9
NIS-**7**	-10.1	NIP-**7**	-2.4	NISac-**7**	-25.6	NBS-**7**	-0.4	NBP-**7**	-2.4	NBSac-**7**	-1.7
NIS-**8**	-42	NIP-**8**	-4.5	NISac-**8**	-62.8	NBS-**8**	-7.2	NBP-**8**	-4.5	NBSac-**8**	-38.5
NIS-**9**	-40.2	NIP-**9**	-1.7	NISac-**9**	-62.2	NBS-**9**	-6.2	NBP-**9**	-1.7	NBSac-**9**	-32.6
NIS-**10**	-37.2	NIP-**10**	-4.1	NISac-**10**	-59.5	NBS-**10**	-3.7	NBP-**10**	-4.1	NBSac-**10**	-32.3
NIS-**11**	-14	NIP-**11**	-12.3	NISac-**11**	-42.8	NBS-**11**	-1.6	NBP-**11**	-1.0	NBSac-**11**	-2.8
NIS-**12**	-24	NIP-**12**	-24.0	NISac-**12**	-47.7	NBS-**12**	-0.9	NBP-**12**	-0.5	NBSac-**12**	-47.7
NIS-**13**	-35.6	NIP-**13**	-37.4	NISac-**13**	-51.5	NBS-**13**	-3.0	NBP-**13**	-3.0	NBSac-**13**	-24.7
NIS-**14**	-8.2	NIP-**14**	3.1	NISac-**14**	-43.7	NBS-**14**	0.5	NBP-**14**	0.08	NBSac-**14**	-4.1
NIS-**15**	-40.5	NIP-**15**	-37.8	NISac-**15**	-72.2	NBS-**15**	-5.3	NBP-**15**	-3.8	NBSac-**15**	-50.2
NIS-**16**	-2.6	NIP-**16**	-0.8	NISac-**16**	-50.2	NBS-**16**	-2.5	NBP-**16**	-0.7	NBSac-**16**	-73.5

[a]$\Delta\delta N_{coord} = \Delta\delta N_{complex} - \Delta\delta N_{pyridine}$.

Table S11. Solution ^{1}H NMR association constants of N-haloimide XB complexes ($CDCl_3$, 298.0K)

**Compl**	**Ka (M^{-1})**	**Compl**	**Ka (M^{-1})**	**Compl**	**Ka (M^{-1})**	**Compl**	**Ka (M^{-1})**	**Compl**	**Ka (M^{-1})**	**Compl**	**Ka (M^{-1})**
NIS-**1**	1471±718	NIP-**1**	1124±157	NISac-**1**	2610 ± 313	NBS-**1**	4±1	NBP-**1**	2	NBSac-**1**	424±18
NIS-**2**	31 ± 1	NIP-**2**	12±0.1	NISac-**2**	1100±21	NBS-**2**	1[a]	NBP-**2**	1[a]	NBSac-**2**	1[a]
NIS-**3**	92 ± 1	NIP-**3**	23±1	NISac-**3**	1424±47	NBS-**3**	1[a]	NBP-**3**	1[a]	NBSac-**3**	5±1
NIS-**4**	57 ± 1	NIP-**4**	18±1	NISac-**4**	1472±38	NBS-**4**	1[a]	NBP-**4**	1[a]	NBSac-**4**	5±1
NIS-**5**	94 ± 5	NIP-**5**	56±1	NISac-**5**	44272[b]	NBS-**5**	1[a]	NBP-**5**	1[a]	NBSac-**5**	15±1
NIS-**6**	4 ± 1	NIP-**6**	7±1	NISac-**6**	236±5	NBS-**6**	2[a]	NBP-**6**	1[a]	NBSac-**6**	3±1
NIS-**7**	12±1	NIP-**7**	15±1	NISac-**7**	389±7	NBS-**7**	1[a]	NBP-**7**	1[a]	NBSac-**7**	1[a]
NIS-**8**	3494±1555	NIP-**8**	2790±391	NISac-**8**	14522±3776	NBS-**8**	5±2 (21±1)[c]	NBP-**8** (19±1)[c]	4±1	NBSac-**8**	331±8 (423±23)[c]
NIS-**9**	3398±1611	NIP-**9**	506±37	NISac-**9**	2536±185	NBS-**9**	4±3	NBP-**9**	2[a]	NBSac-**9**	455±9
NIS-**10**	588±159	NIP-**10**	617±35	NISac-**10**	3250±374	NBS-**10**	2	NBP-**10**	1[a]	NBSac-**10**	210±4
NIS-**11**	114 ± 2	NIP-**11**	22±1	NISac-**11**	1545±142	NBS-**11**	1[a]	NBP-**11**	1[a]	NBSac-**11**	4±1
NIS-**12**	465 ± 32	NIP-**12**	59±1	NISac-**12**	125282[b]	NBS-**12**	1[a]	NBP-**12**	1[a]	NBSac-**12**	21±1
NIS-**13**	931±13	NIP-**13**	932±14	NISac-**13**	282807[b]	NBS-**13**	1[a]	NBP-**13**	2[a]	NBSac-**13**	294±2
NIS-**14**	490±59	NIP-**14**	375±11	NISac-**14**	25495[b]	NBS-**14**	1[a]	NBP-**14**	2[a]	NBSac-**14**	147±1
NIS-**15**	522±79	NIP-**15**	291±16	NISac-**15**	144459[b]	NBS-**15**	2[a]	NBP-**15**	2[a]	NBSac-**15**	394±12
NIS-**16**	--	NIP-**16**	--	NISac-**16**	--	NBS-**16**	--	NBP-**16**	--	NBSac-**16**	--

[a]Weak binding, values are shown for reference. [b]>100% excessive error, values are for reference only. [c]Association constants obtained by using 1M pyridine stock solutions.

5). ^{1}H-^{15}N HMBC-NMR Spectra for complexes NBS-1 - NISac-15

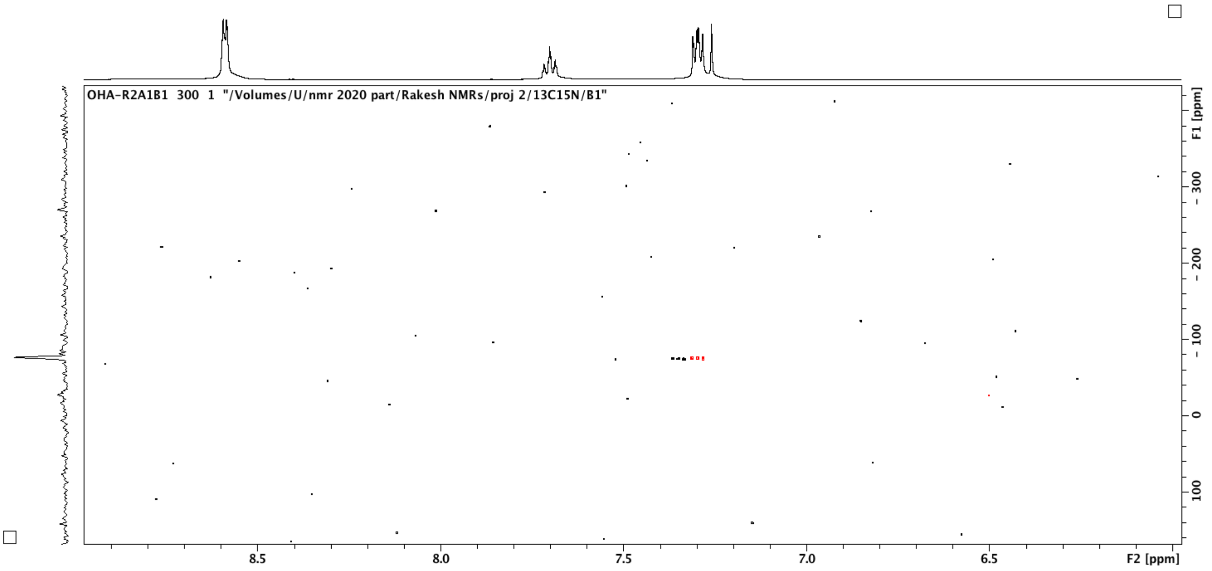

Figure S6. ^{1}H-^{15}N HMBC-NMR Spectra of **1** in black contour and 1:1 complex NBS-**1** in red contour in $CDCl_3$ at 303K.

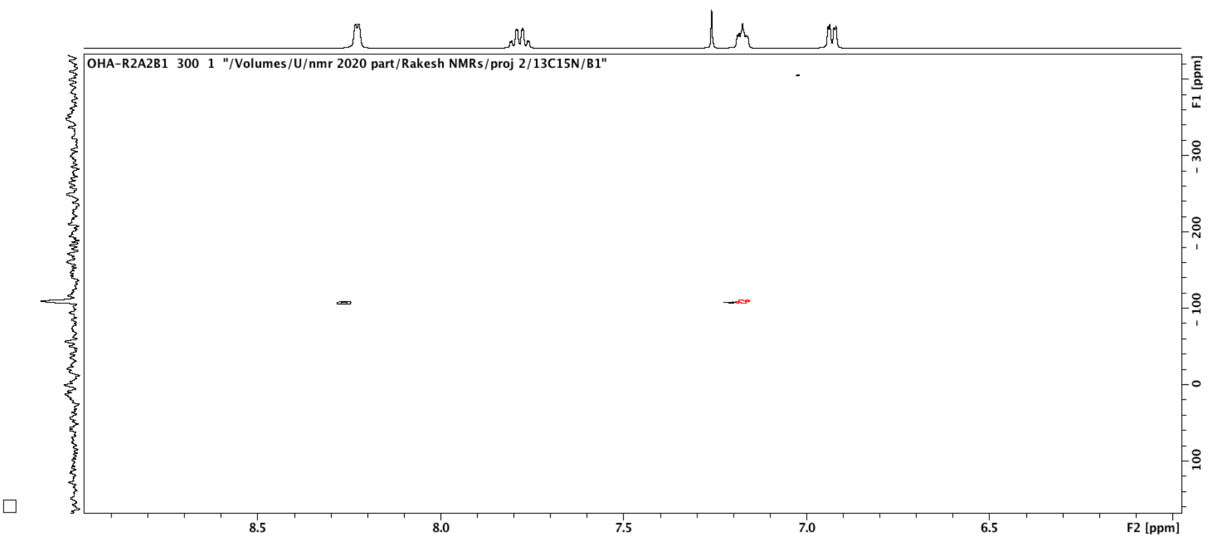

Figure S7. ^{1}H-^{15}N HMBC-NMR Spectra of **2** in black contour and 1:1 complex NBS-**2** in red contour in $CDCl_3$ at 303K.

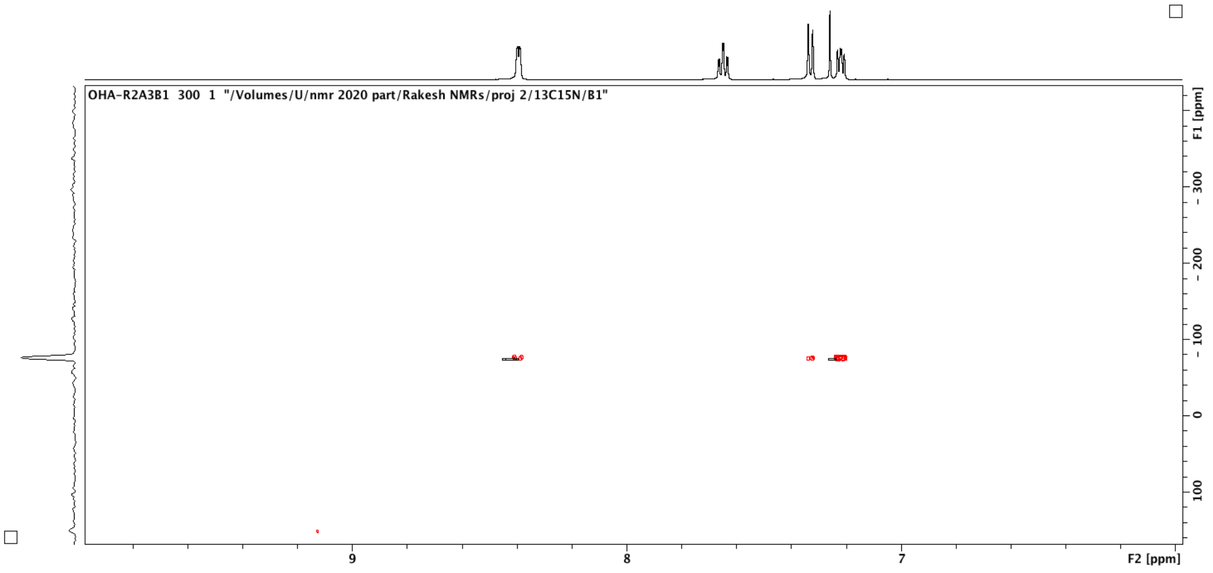

Figure S8. ^1H-^{15}N HMBC-NMR Spectra of **3** in black contour and 1:1 complex NBS-**3** in red contour in $CDCl_3$ at 303K.

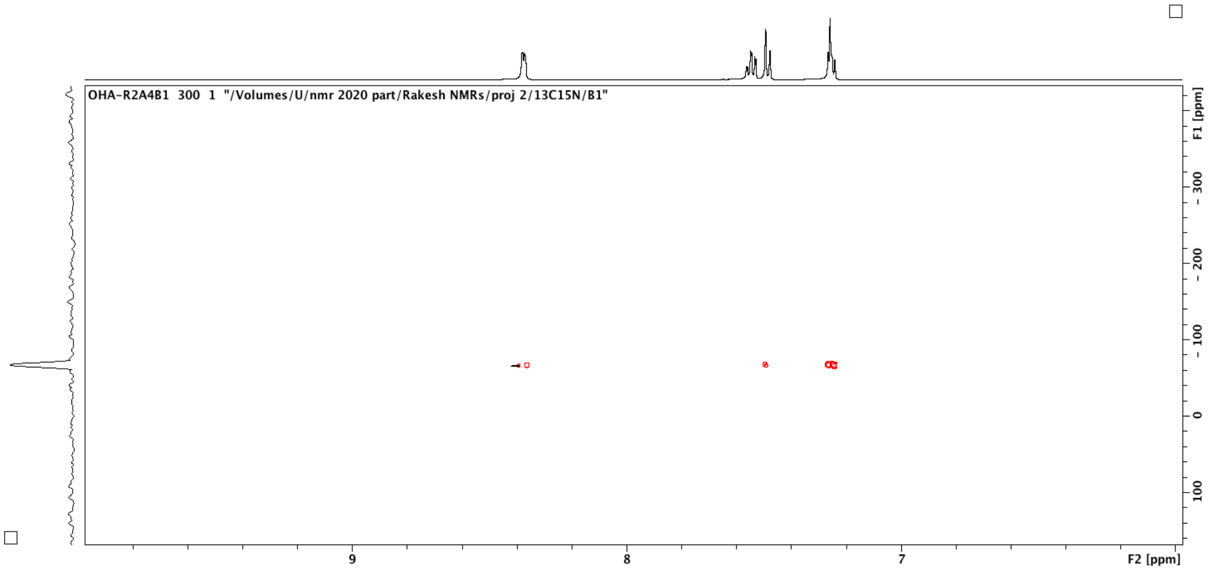

Figure S9. ^1H-^{15}N HMBC-NMR Spectra of **4** in black contour and 1:1 complex NBS-**4** in red contour in $CDCl_3$ at 303K.

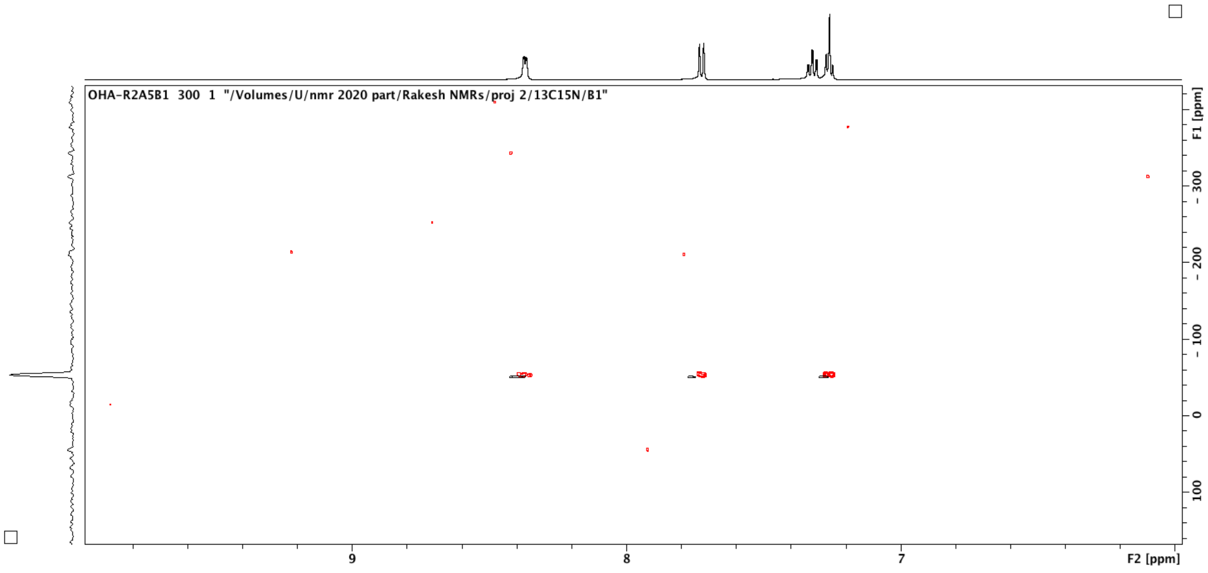

Figure S10. ^{1}H-^{15}N HMBC-NMR Spectra of **5** in black contour and 1:1 complex NBS-**5** in red contour in $CDCl_3$ at 303K.

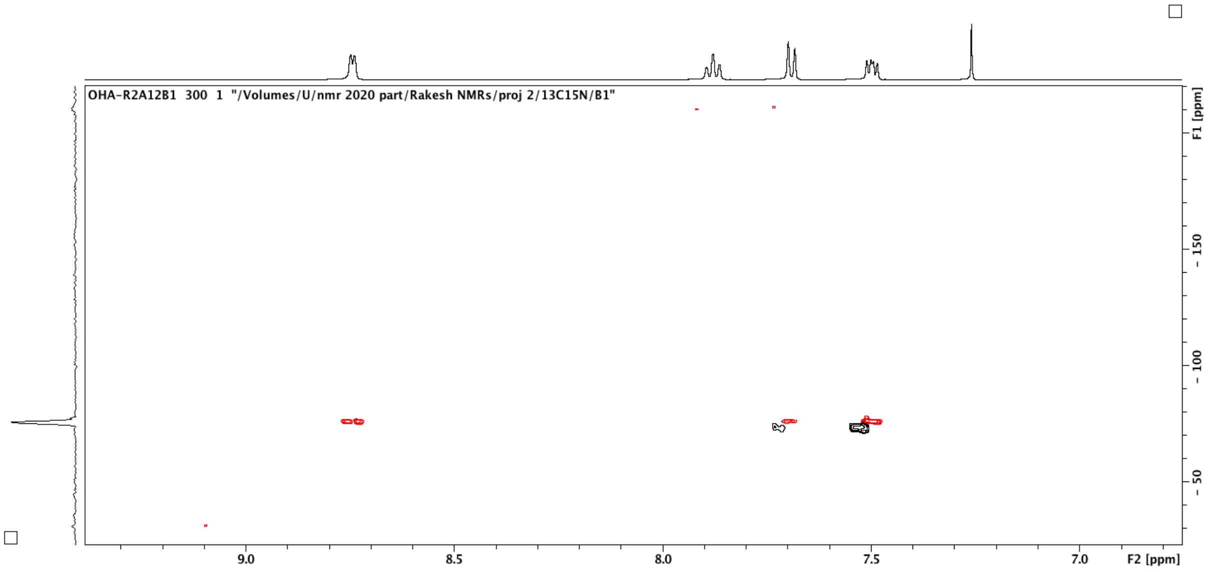

Figure S11. ^{1}H-^{15}N HMBC-NMR Spectra of **6** in black contour and 1:1 complex NBS-**6** in red contour in $CDCl_3$ at 303K.

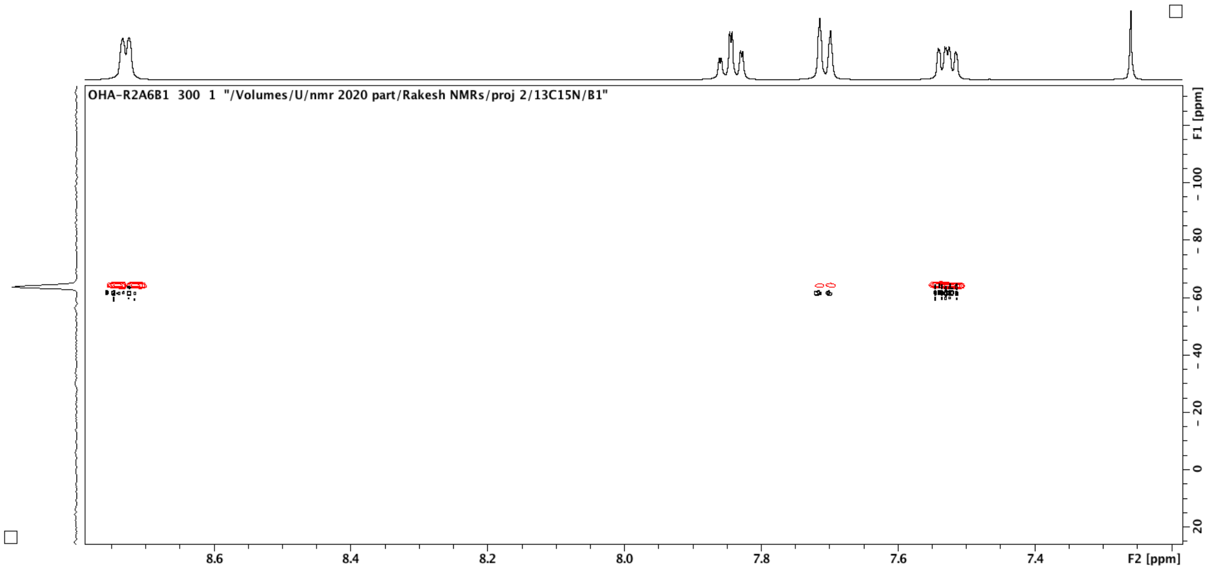

Figure S12. ^{1}H-^{15}N HMBC-NMR Spectra of **7** in black contour and 1:1 complex NBS-**7** in red contour in $CDCl_3$ at 303K.

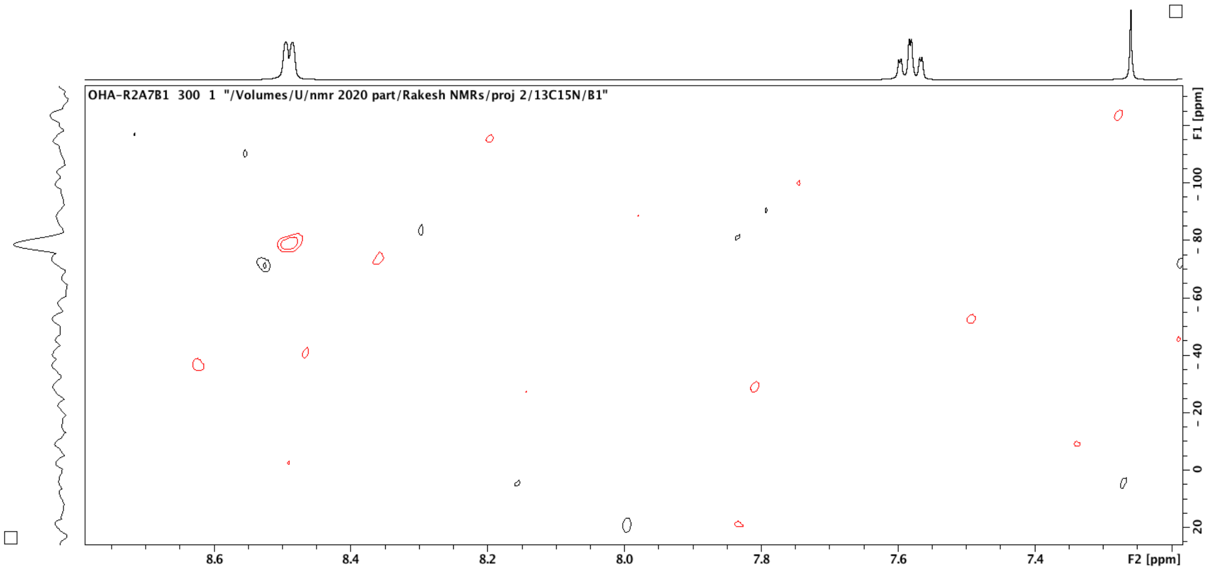

Figure S13. ^{1}H-^{15}N HMBC-NMR Spectra of **8** in black contour and 1:1 complex NBS-**8** in red contour in $CDCl_3$ at 303K.

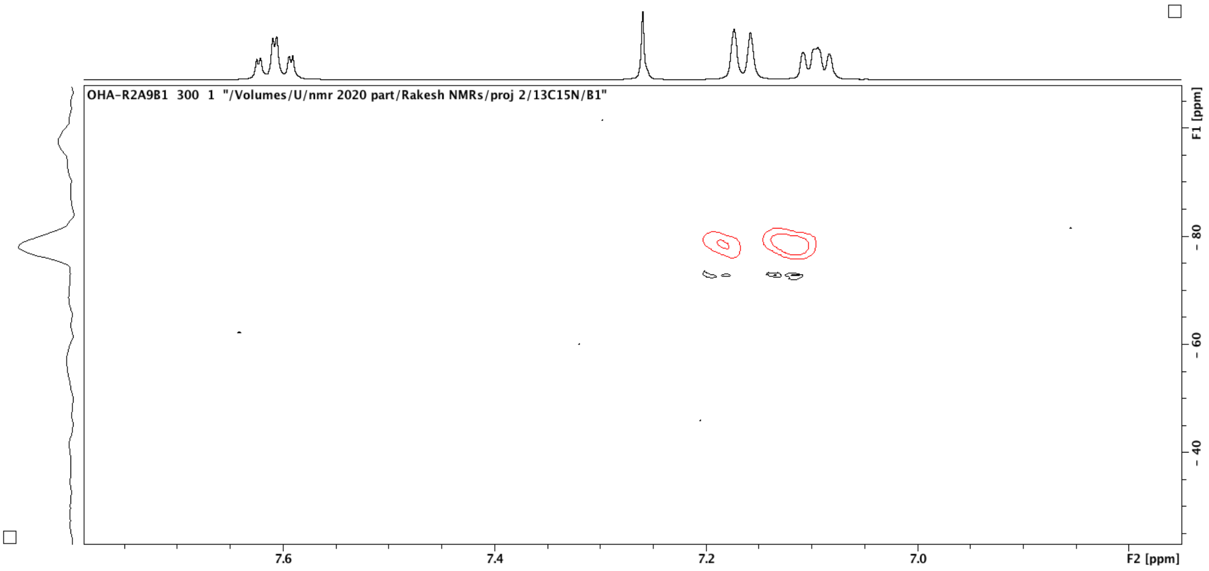

Figure S14. ^{1}H-^{15}N HMBC-NMR Spectra of **9** in black contour and 1:1 complex NBS-**9** in red contour in $CDCl_3$ at 303K.

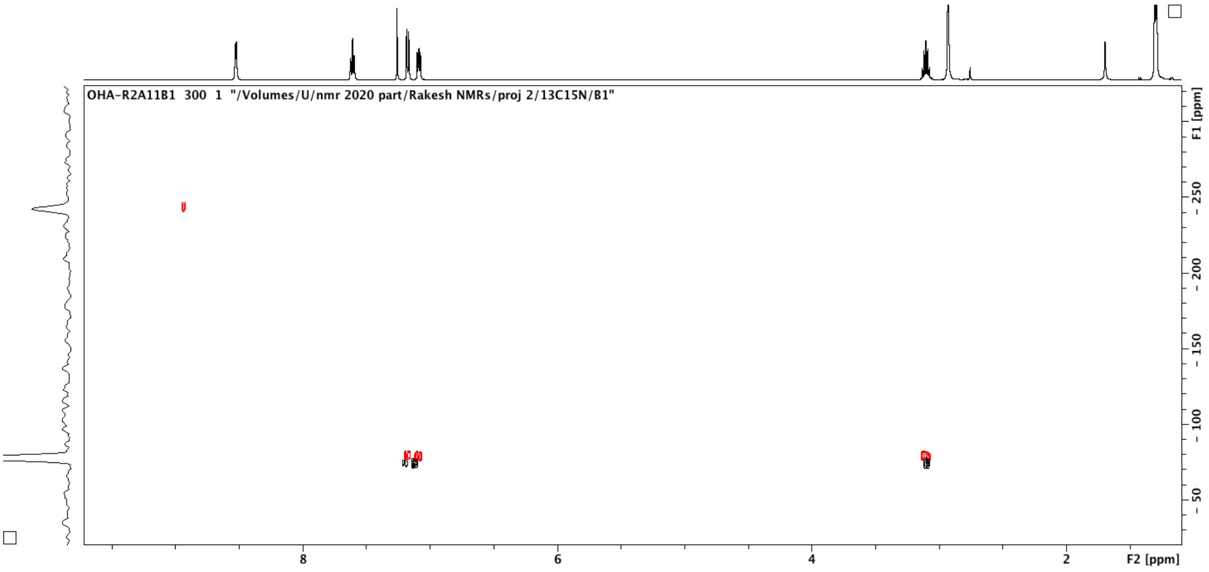

Figure S15. ^{1}H-^{15}N HMBC-NMR Spectra of **10** in black contour and 1:1 complex NBS-**10** in red contour in $CDCl_3$ at 303K.

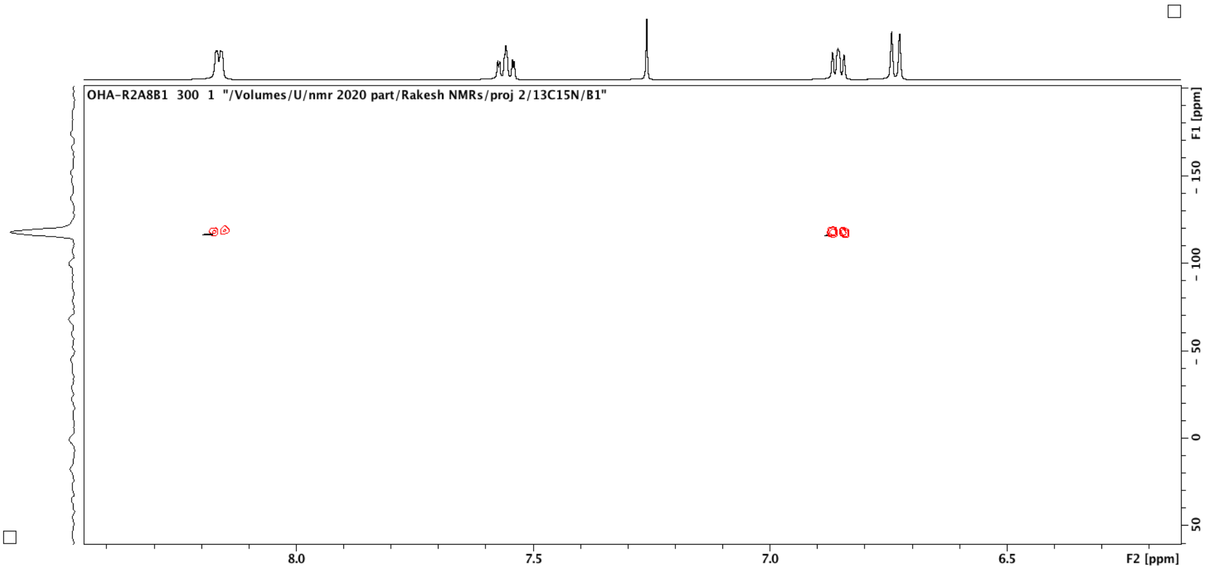

Figure S16. ^{1}H-^{15}N HMBC-NMR Spectra of **11** in black contour and 1:1 complex NBS-**11** in red contour in $CDCl_3$ at 303K.

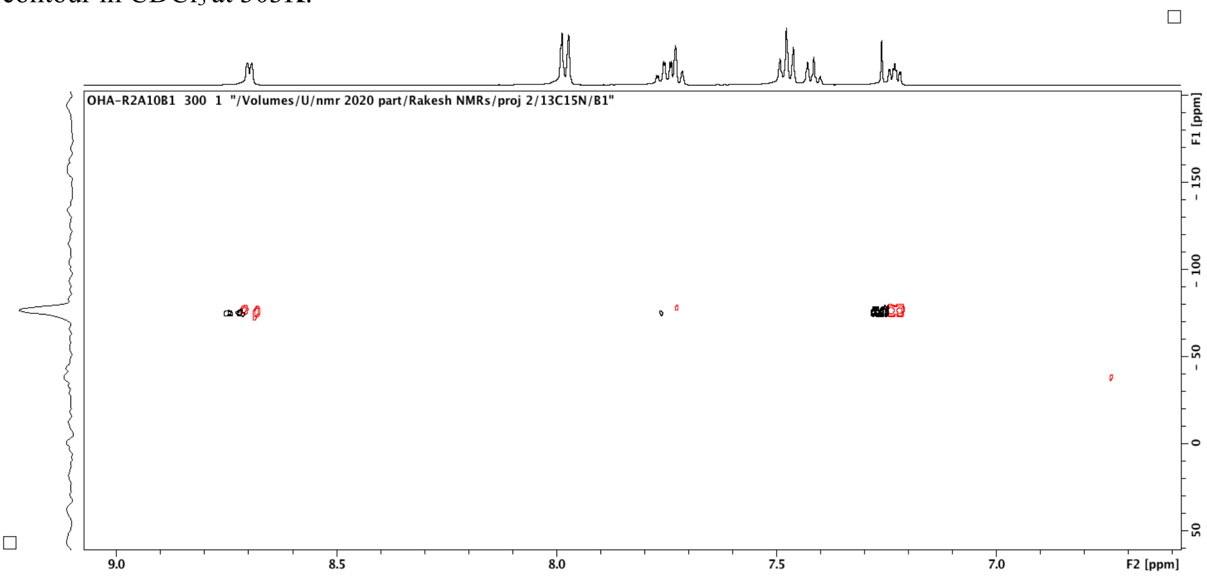

Figure S17. ^{1}H-^{15}N HMBC-NMR Spectra of **12** in black contour and 1:1 complex NBS-**12** in red contour in $CDCl_3$ at 303K.

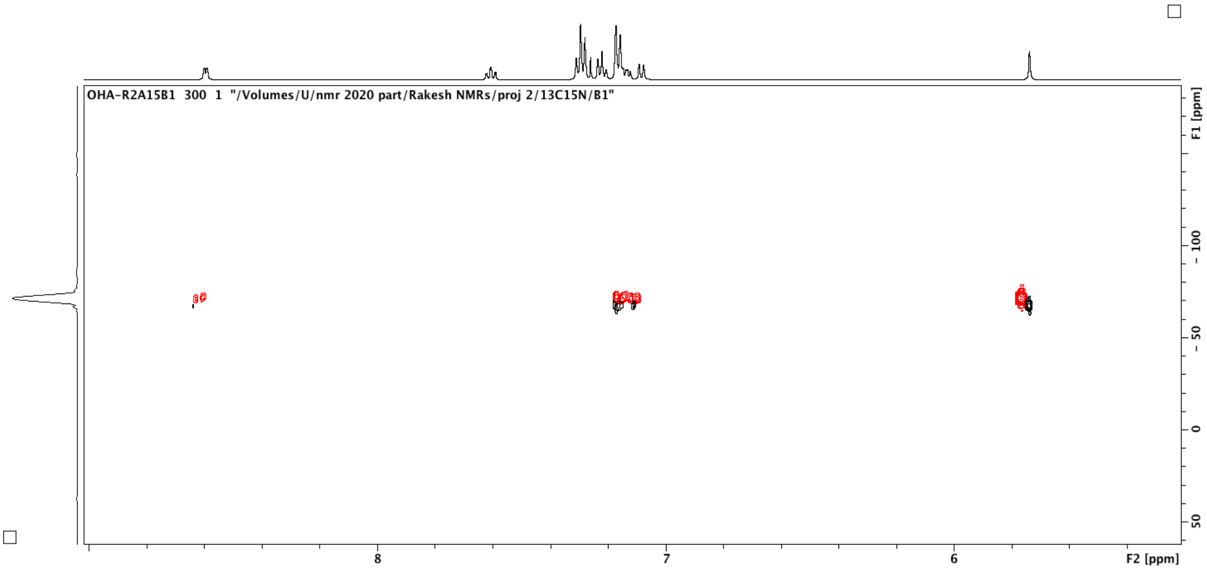

Figure S18. ^{1}H-^{15}N HMBC-NMR Spectra of **13** in black contour and 1:1 complex NBS-**13** in red contour in $CDCl_3$ at 303K.

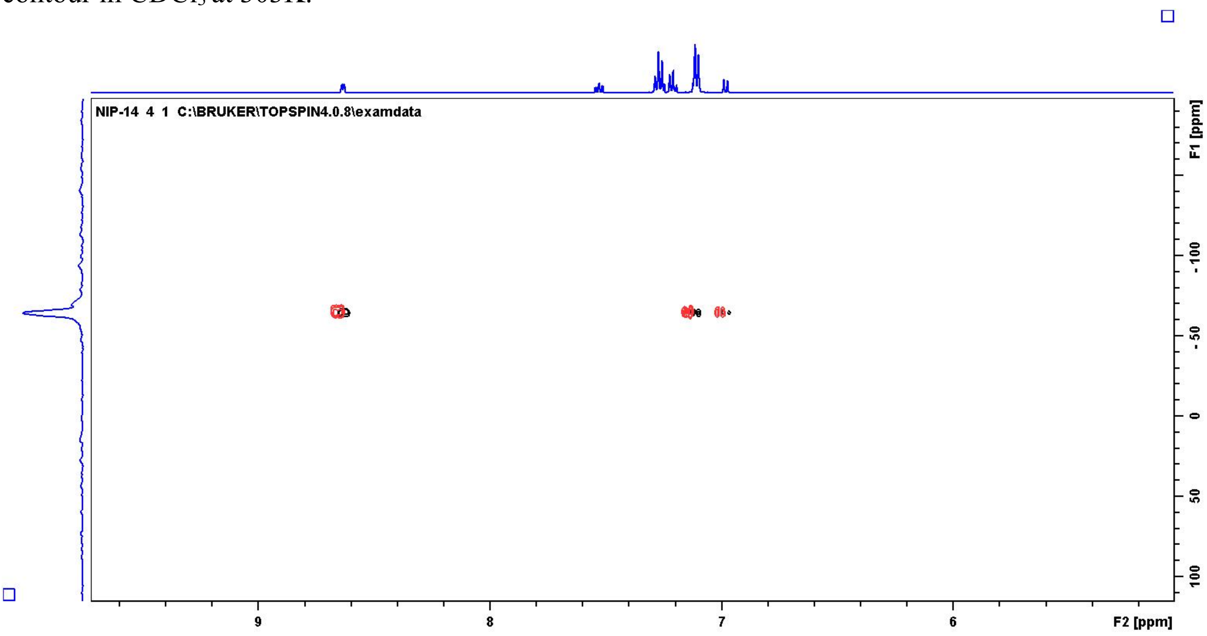

Figure S19. ^{1}H-^{15}N HMBC-NMR Spectra of **14** in black contour and 1:1 complex NBS-**14** in red contour in $CDCl_3$ at 303K, 500MHz.

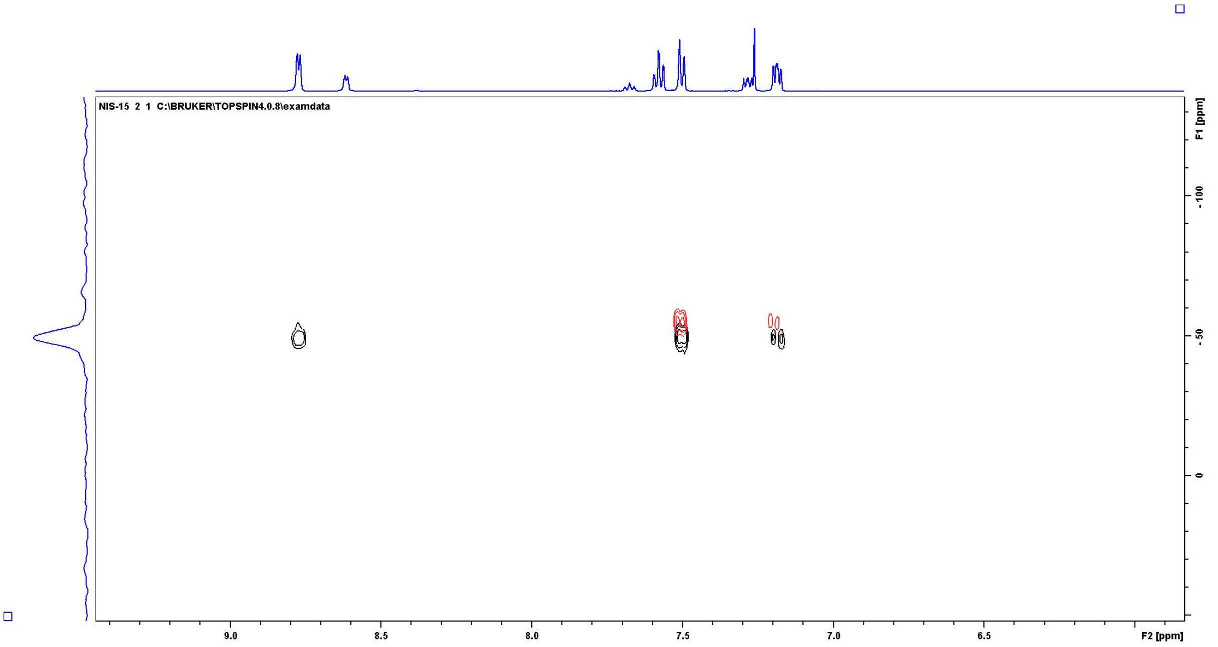

Figure S20. ^{1}H-^{15}N HMBC-NMR Spectra of **15** in black contour and 1:1 complex NBS-**15** in red contour in $CDCl_3$ at 303K, 500MHz.

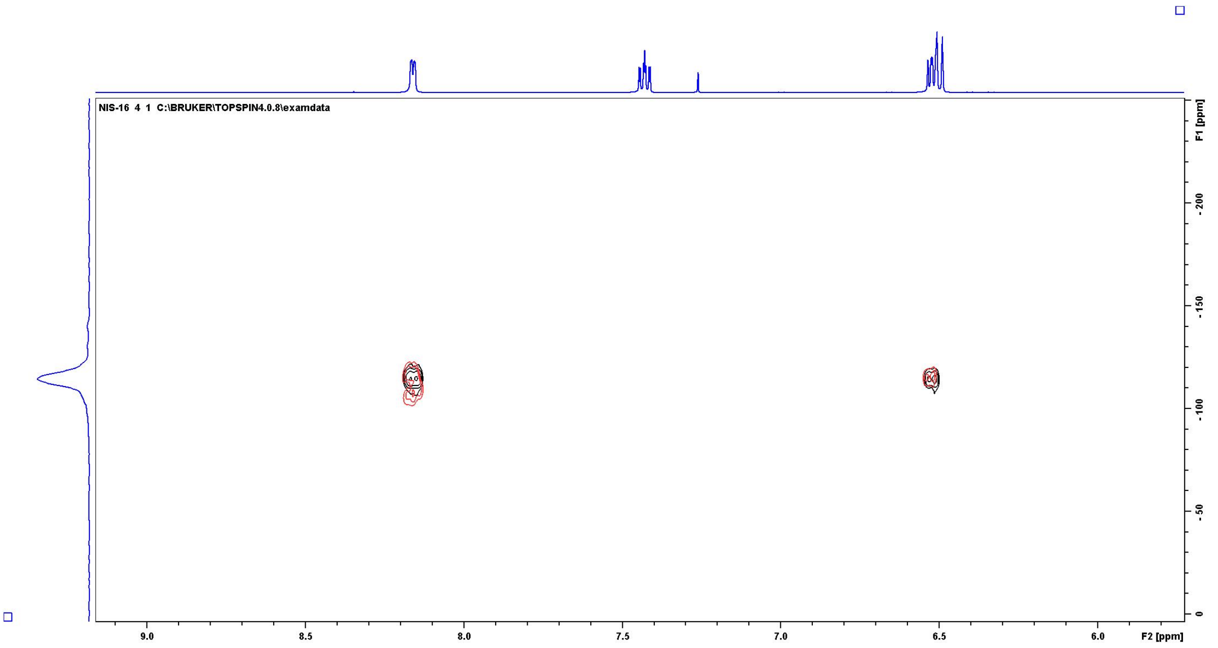

Figure S21. ^{1}H-^{15}N HMBC-NMR Spectra of **16** in black contour and 1:1 complex NBS-**16** in red contour in $CDCl_3$ at 303K, 500MHz.

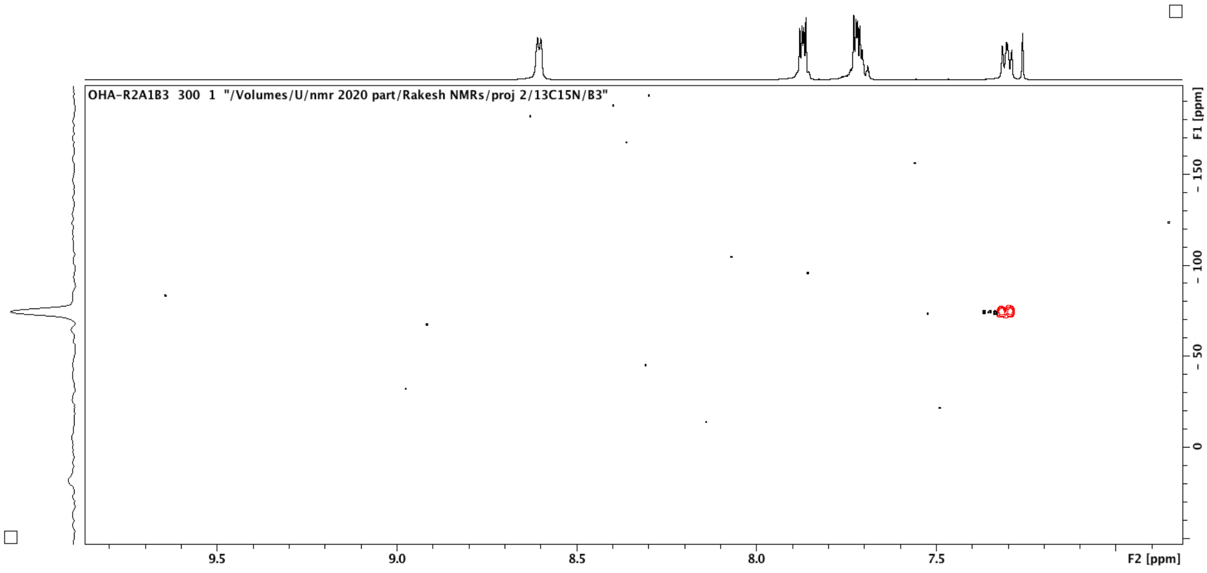

Figure S22. ^{1}H-^{15}N HMBC-NMR Spectra of **1** in black contour and 1:1 complex NBP-**1** in red contour in $CDCl_3$ at 303K.

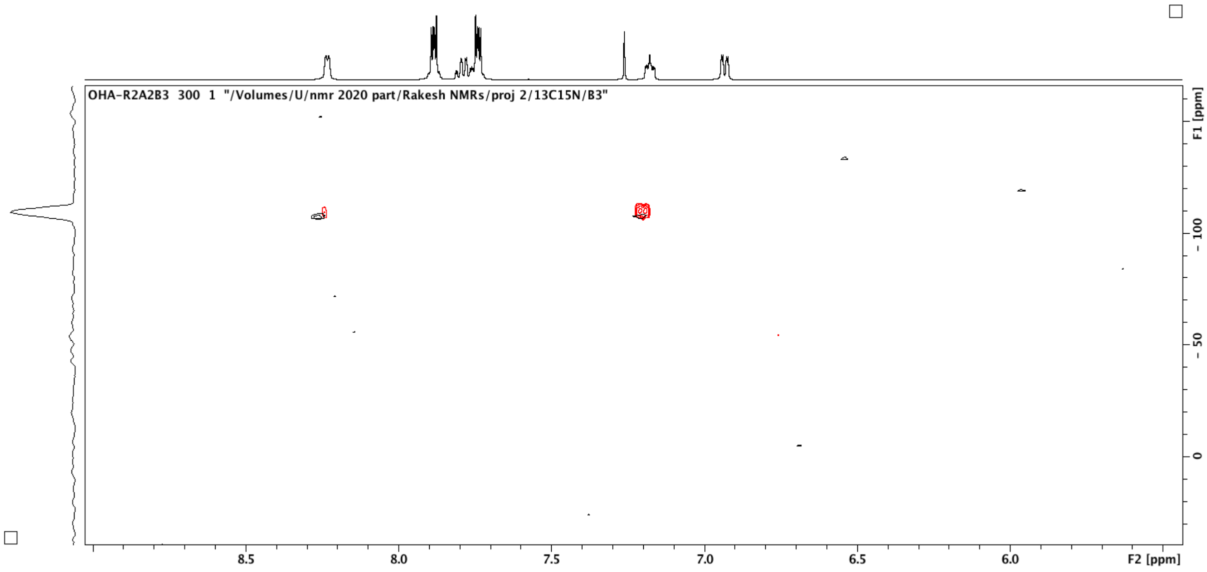

Figure S23. ^{1}H-^{15}N HMBC-NMR Spectra of **2** in black contour and 1:1 complex NBP-**2** in red contour in $CDCl_3$ at 303K.

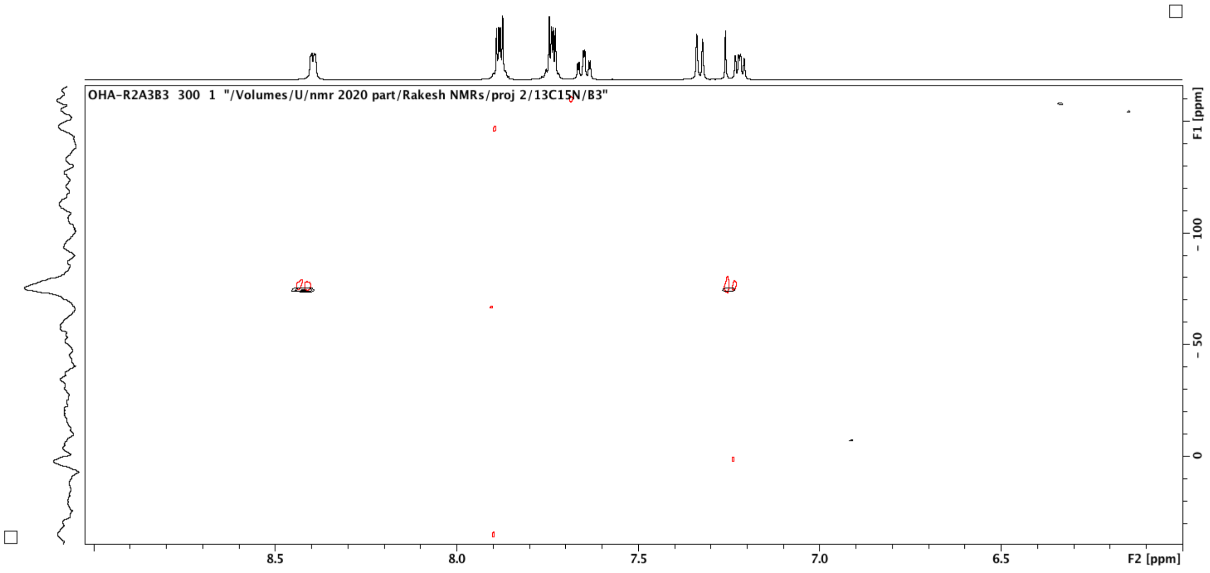

Figure S24. ^{1}H-^{15}N HMBC-NMR Spectra of **3** in black contour and 1:1 complex NBP-**3** in red contour in $CDCl_3$ at 303K.

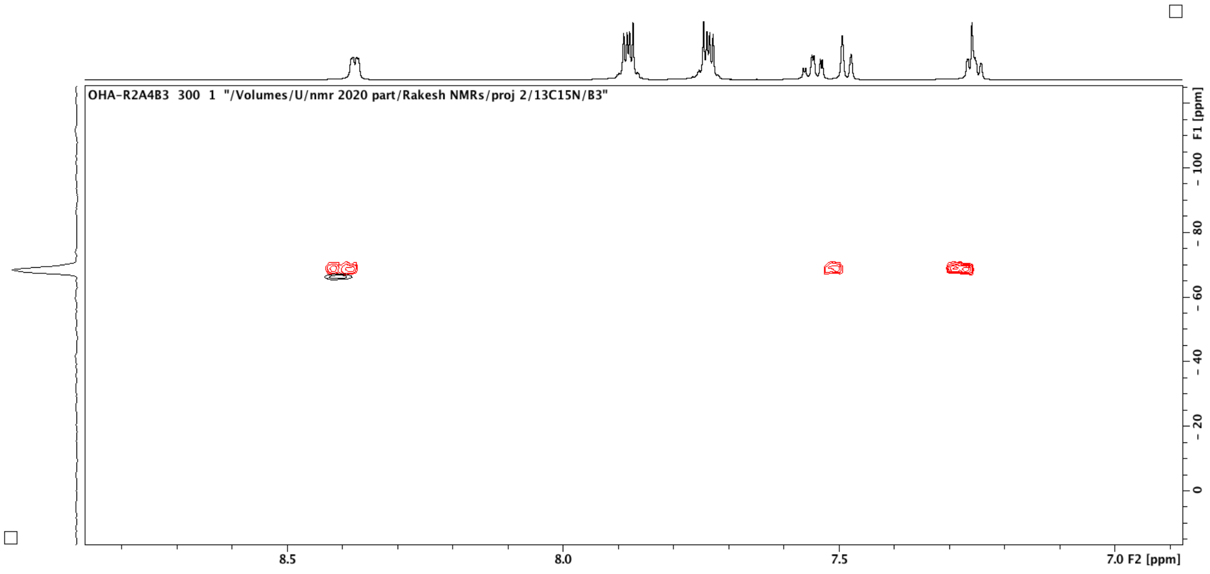

Figure S25. ^{1}H-^{15}N HMBC-NMR Spectra of **4** in black contour and 1:1 complex NBP-**4** in red contour in $CDCl_3$ at 303K.

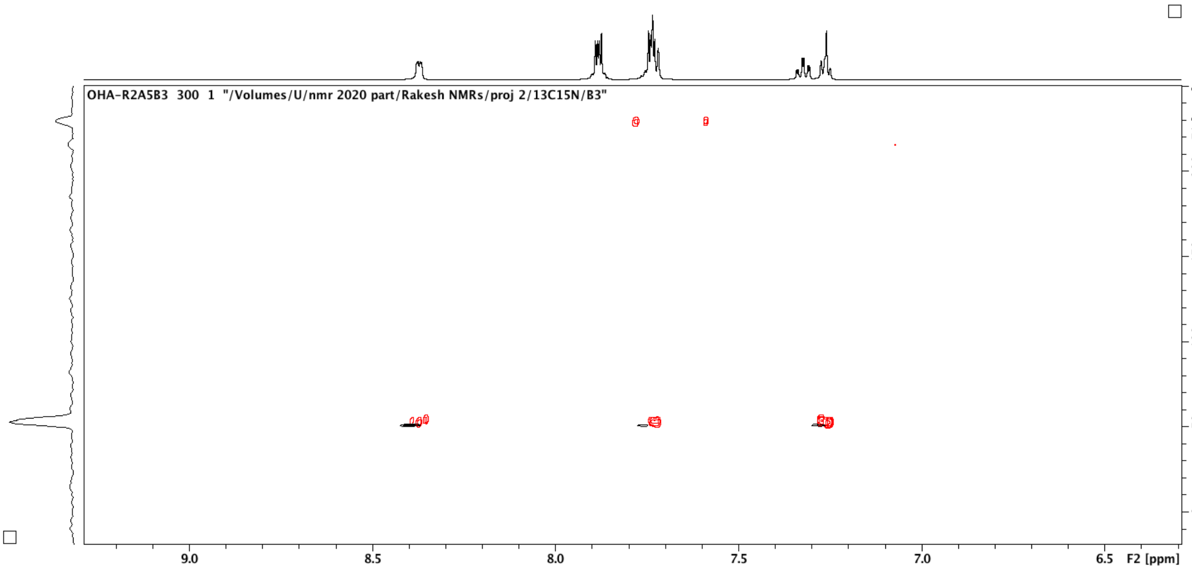

Figure S26. ^{1}H-^{15}N HMBC-NMR Spectra of **5** in black contour and 1:1 complex NBP-**5** in red contour in $CDCl_3$ at 303K.

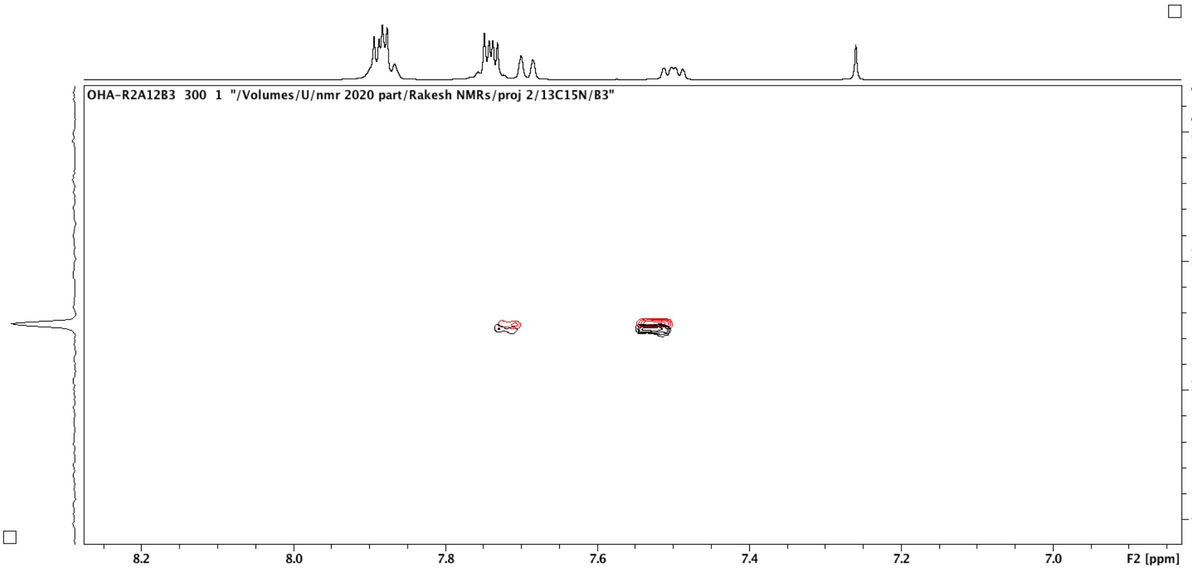

Figure S27. ^{1}H-^{15}N HMBC-NMR Spectra of **6** in black contour and 1:1 complex NBP-**6** in red contour in $CDCl_3$ at 303K.

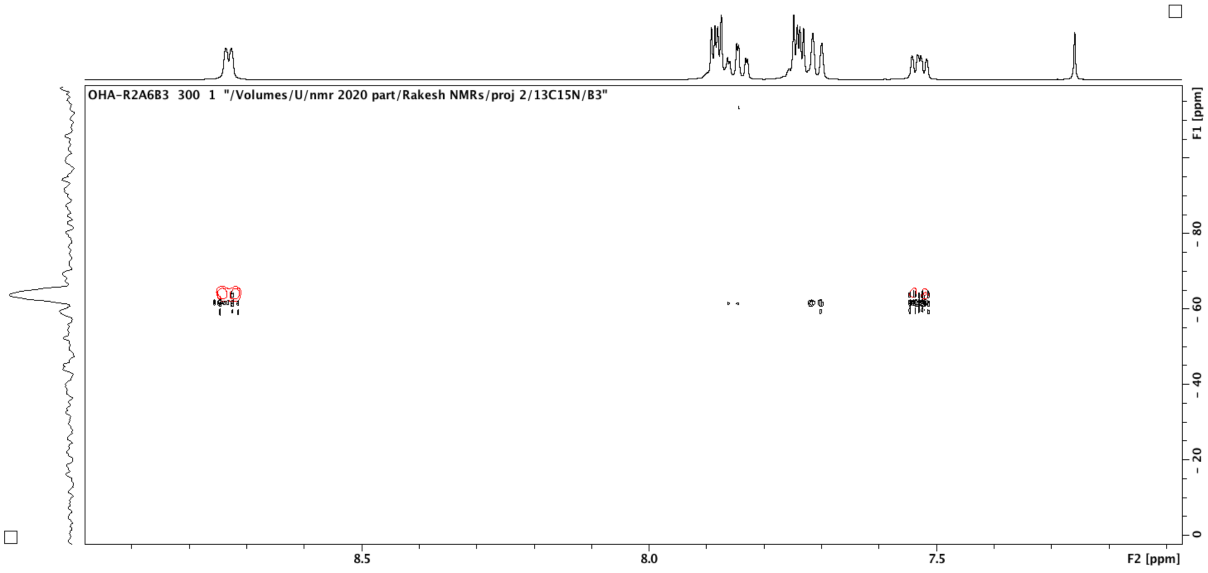

Figure S28. ^{1}H-^{15}N HMBC-NMR Spectra of **7** in black contour and 1:1 complex NBP-**7** in red contour in $CDCl_3$ at 303K.

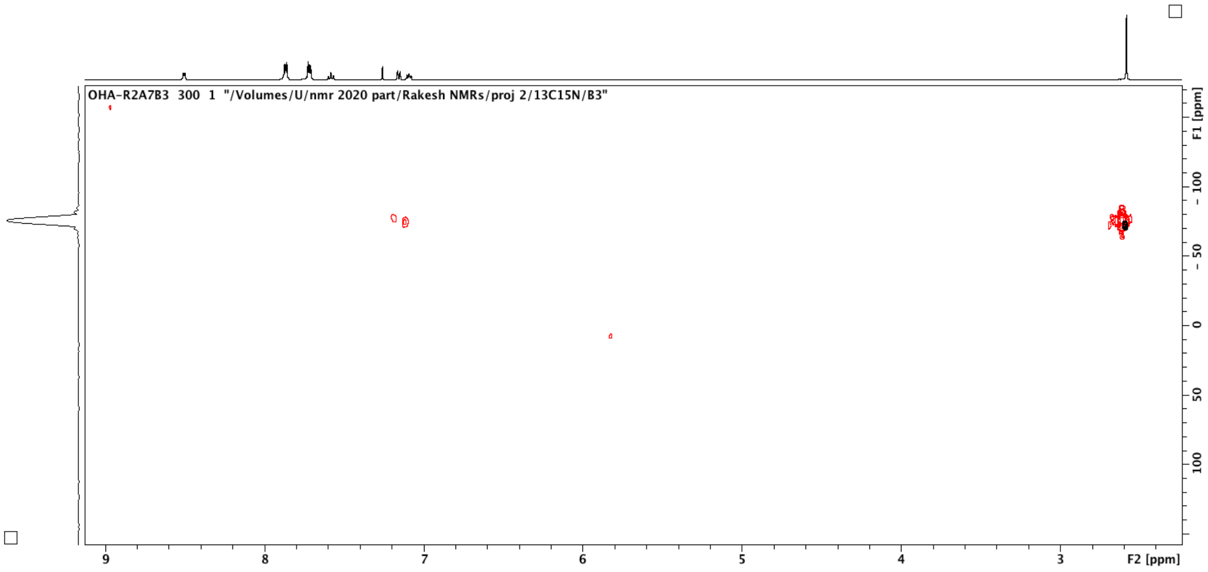

Figure S29. ^{1}H-^{15}N HMBC-NMR Spectra of **8** in black contour and 1:1 complex NBP-**8** in red contour in $CDCl_3$ at 303K.

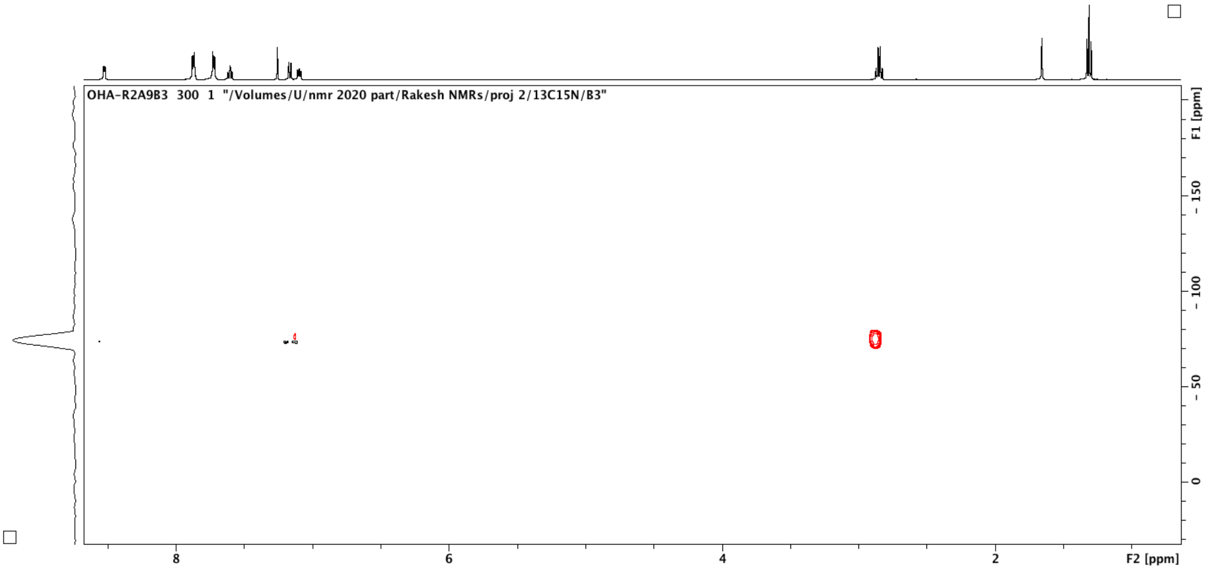

Figure S30. ^{1}H-^{15}N HMBC-NMR Spectra of **9** in black contour and 1:1 complex NBP-**9** in red contour in $CDCl_3$ at 303K.

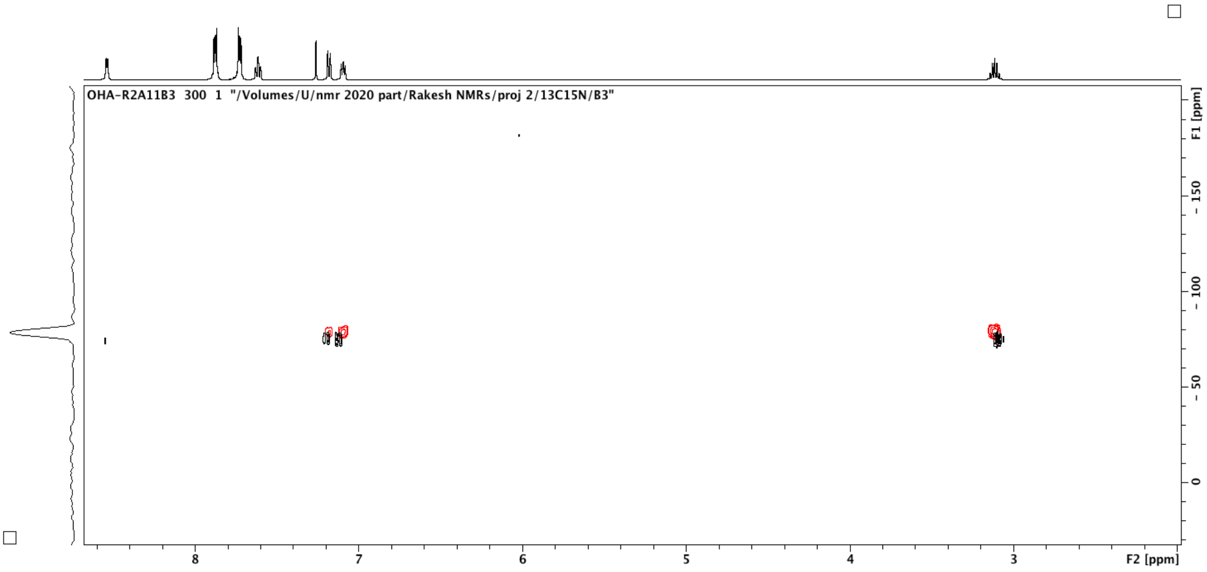

Figure S31. ^{1}H-^{15}N HMBC-NMR Spectra of **10** in black contour and 1:1 complex NBP-**10** in red contour in $CDCl_3$ at 303K.

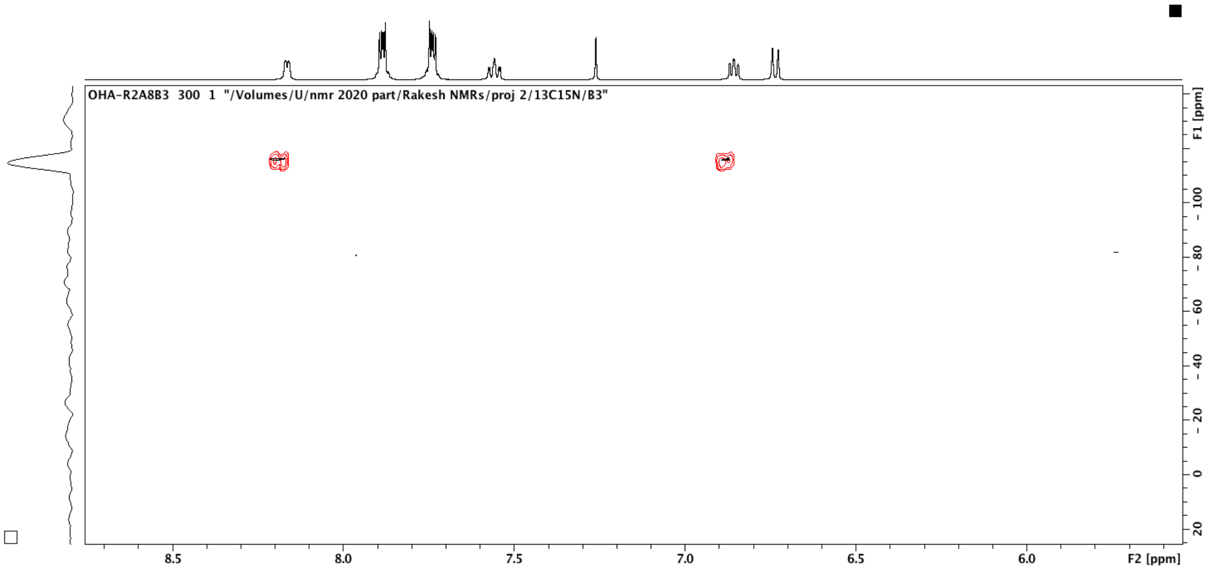

Figure S32. ^{1}H-^{15}N HMBC-NMR Spectra of **11** in black contour and 1:1 complex NBP-**11** in red contour in $CDCl_3$ at 303K.

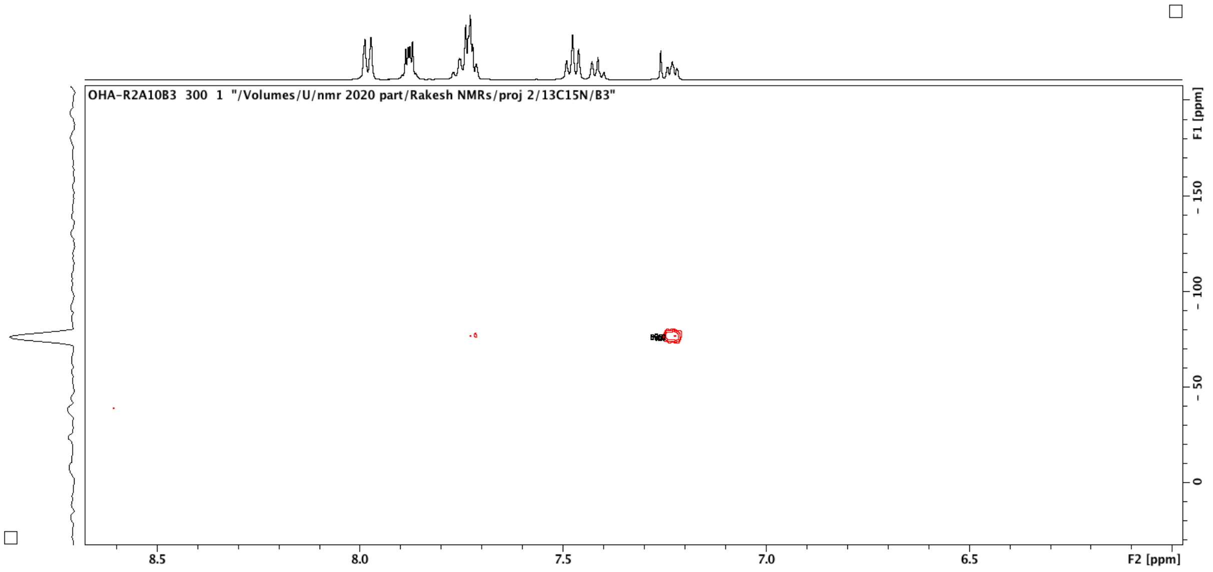

Figure S33. ^{1}H-^{15}N HMBC-NMR Spectra of **12** in black contour and 1:1 complex NBP-**12** in red contour in $CDCl_3$ at 303K.

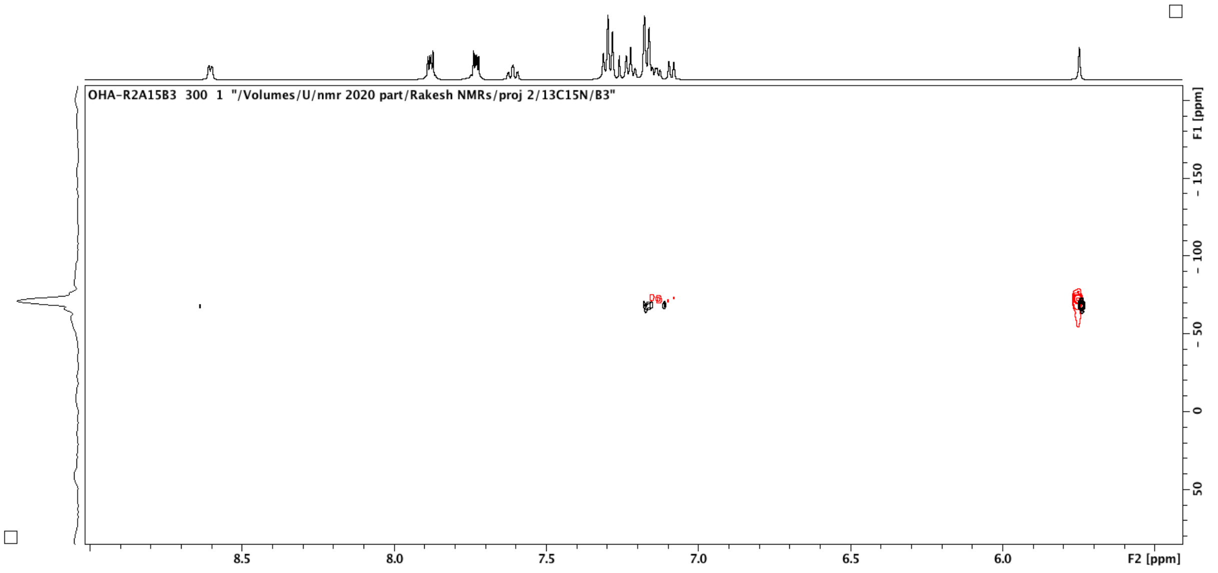

Figure S34. ^{1}H-^{15}N HMBC-NMR Spectra of **13** in black contour and 1:1 complex NBP-**13** in red contour in $CDCl_3$ at 303K.

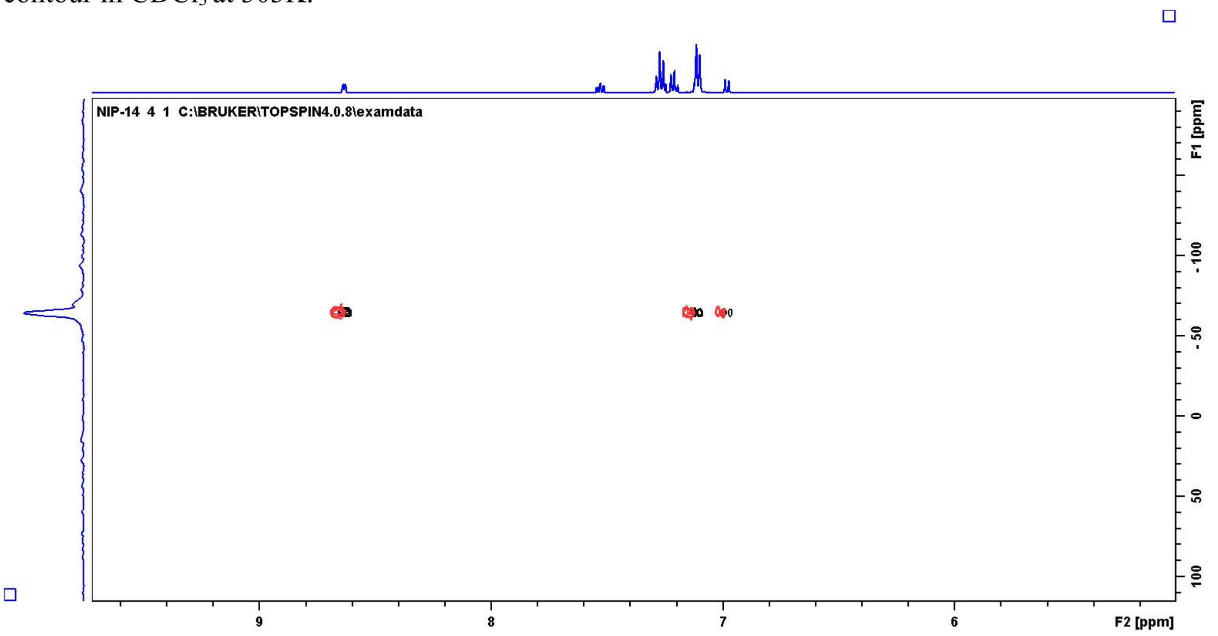

Figure S35. ^{1}H-^{15}N HMBC-NMR Spectra of **14** in black contour and 1:1 complex NBP-**14** in red contour in $CDCl_3$ at 303K.

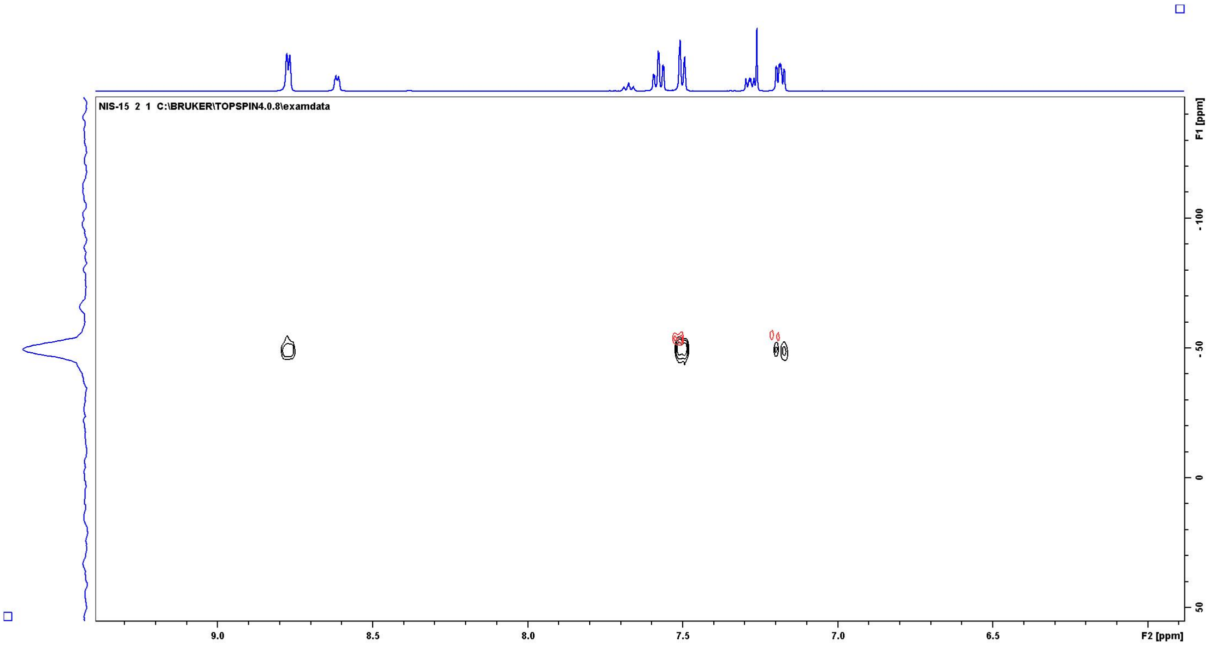

Figure S36. ^{1}H-^{15}N HMBC-NMR Spectra of **15** in black contour and 1:1 complex NBP-**15** in red contour in $CDCl_3$ at 303K.

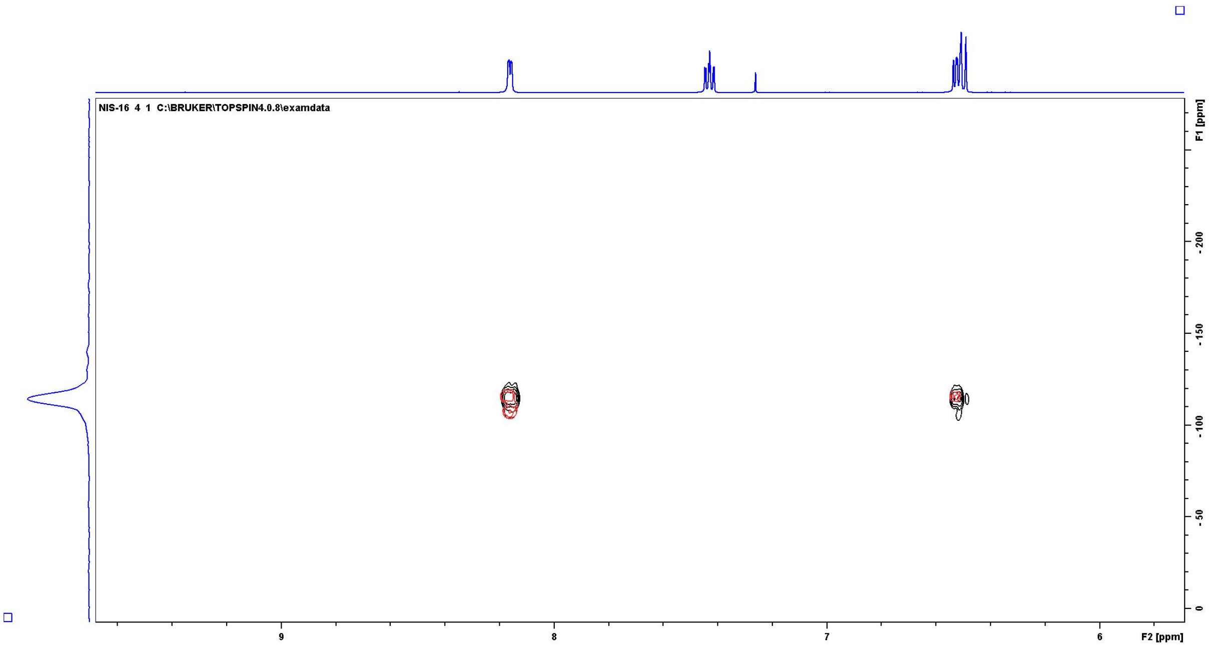

Figure S37. ^{1}H-^{15}N HMBC-NMR Spectra of **16** in black contour and 1:1 complex NBP-**16** in red contour in $CDCl_3$ at 303K.

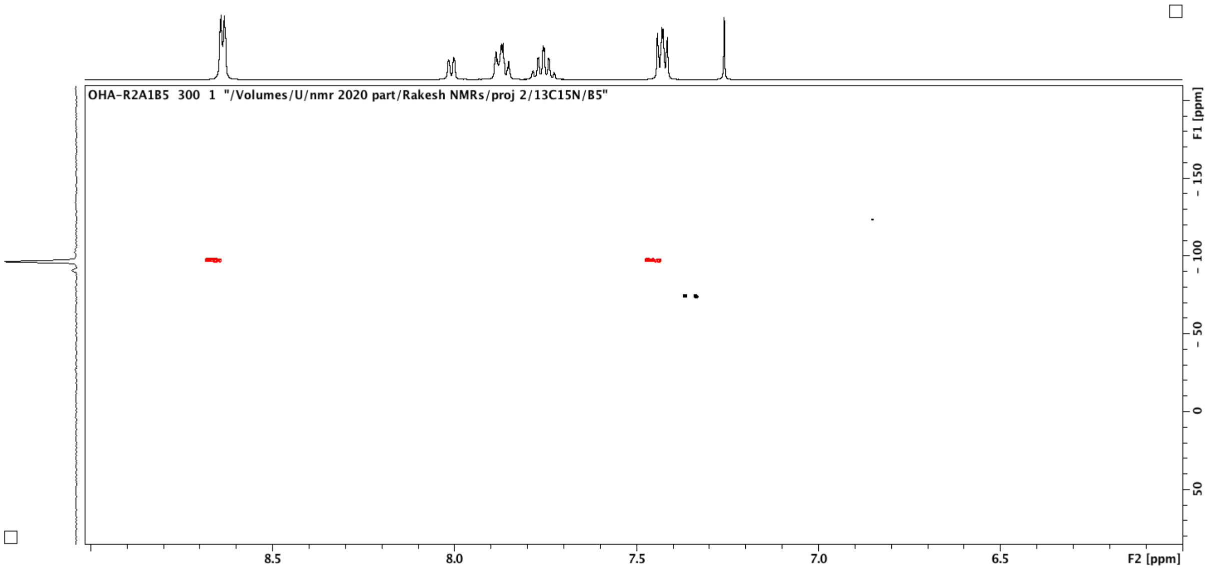

Figure S38. ^{1}H-^{15}N HMBC-NMR Spectra of **1** in black contour and 1:1 complex NBSac-**1** in red contour in $CDCl_3$ at 303K.

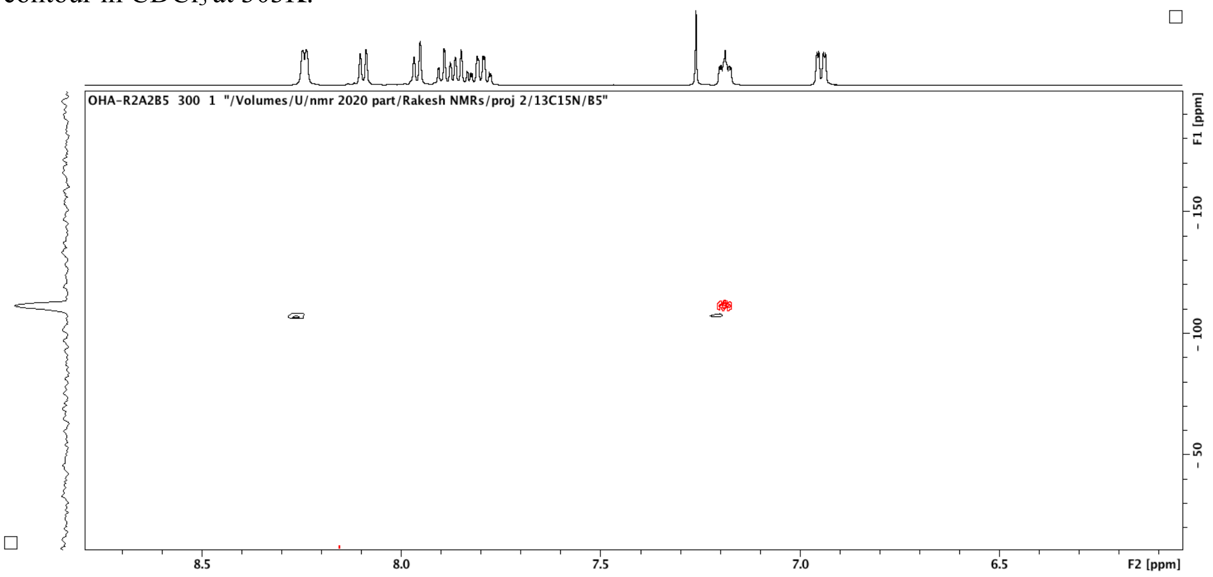

Figure S39. ^{1}H-^{15}N HMBC-NMR Spectra of **2** in black contour and 1:1 complex NBSac-**2** in red contour in $CDCl_3$ at 303K.

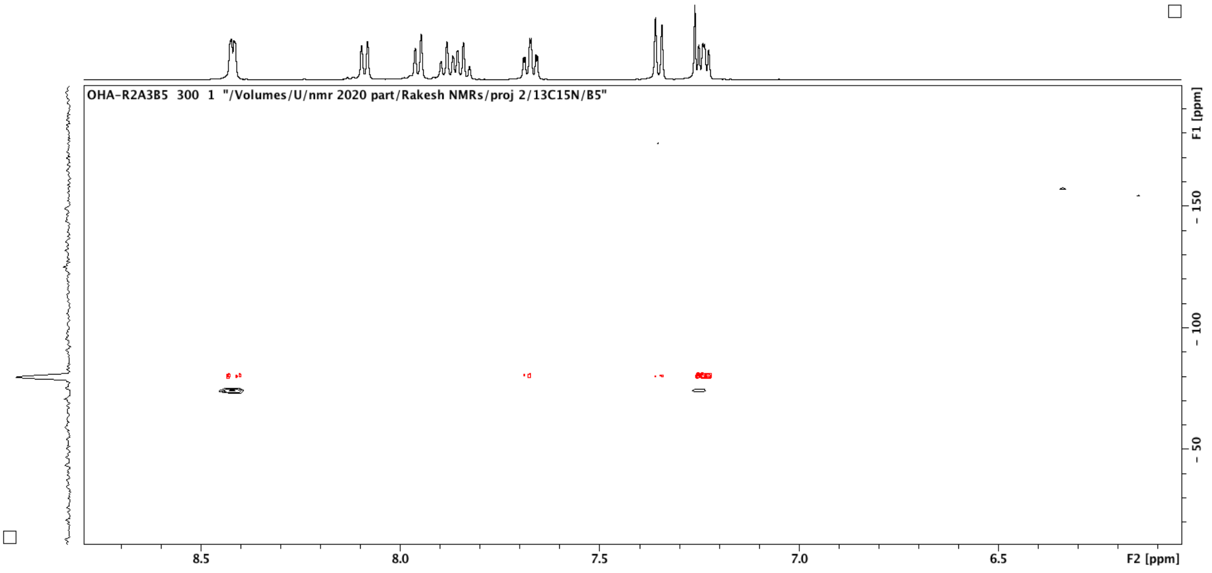

Figure S40. ^{1}H-^{15}N HMBC-NMR Spectra of **3** in black contour and 1:1 complex NBSac-**3** in red contour in $CDCl_3$ at 303K.

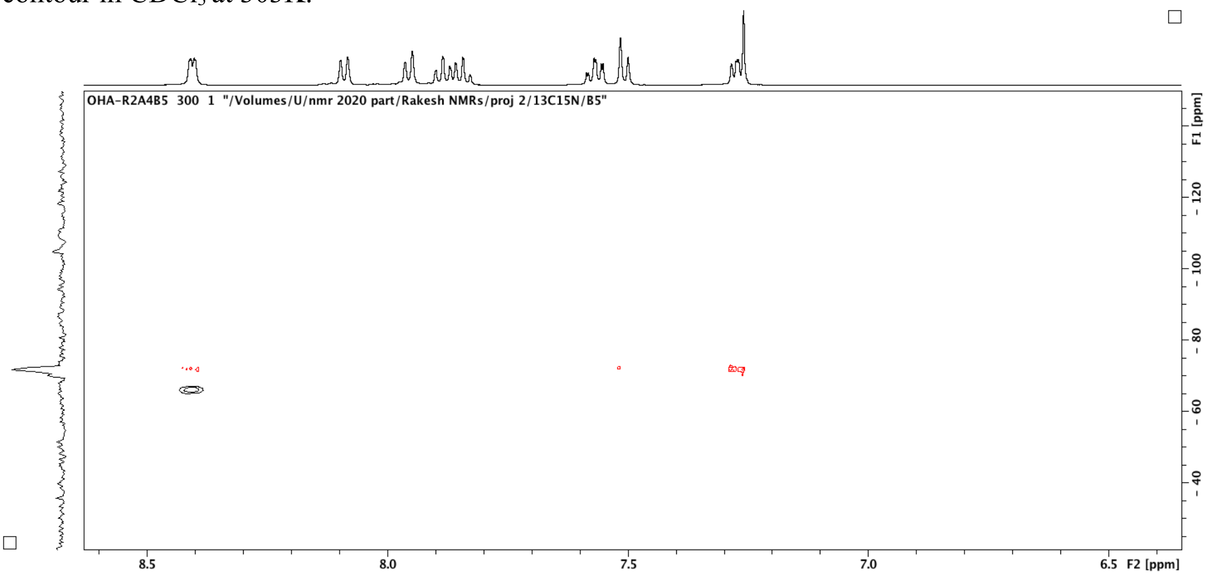

Figure S41. ^{1}H-^{15}N HMBC-NMR Spectra of **4** in black contour and 1:1 complex NBSac-**4** in red contour in $CDCl_3$ at 303K.

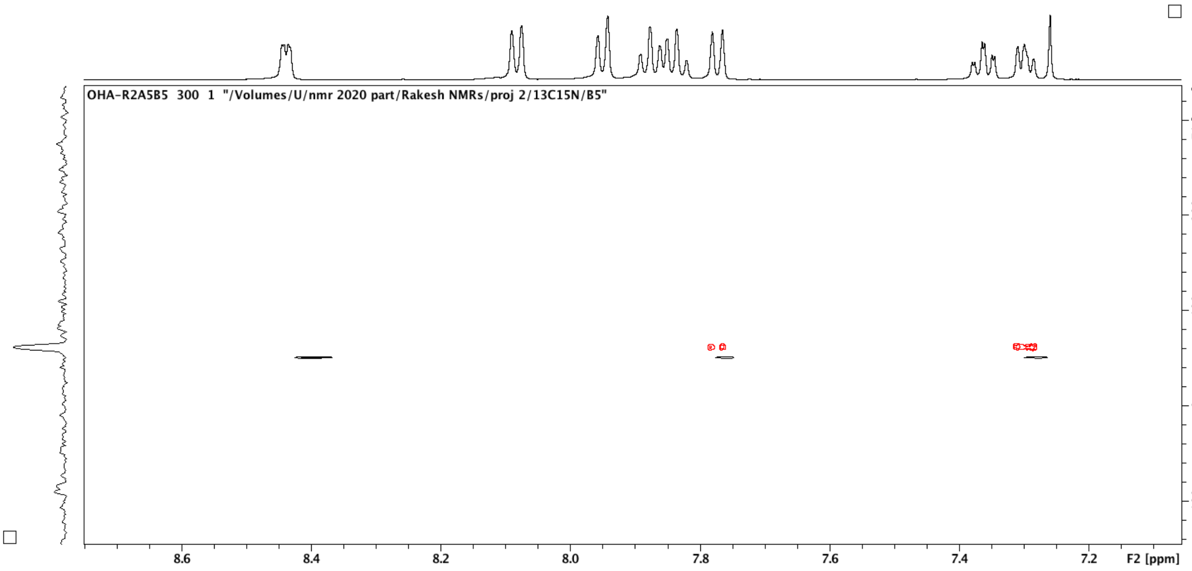

Figure S42. ^{1}H-^{15}N HMBC-NMR Spectra of **5** in black contour and 1:1 complex NBSac-**5** in red contour in $CDCl_3$ at 303K.

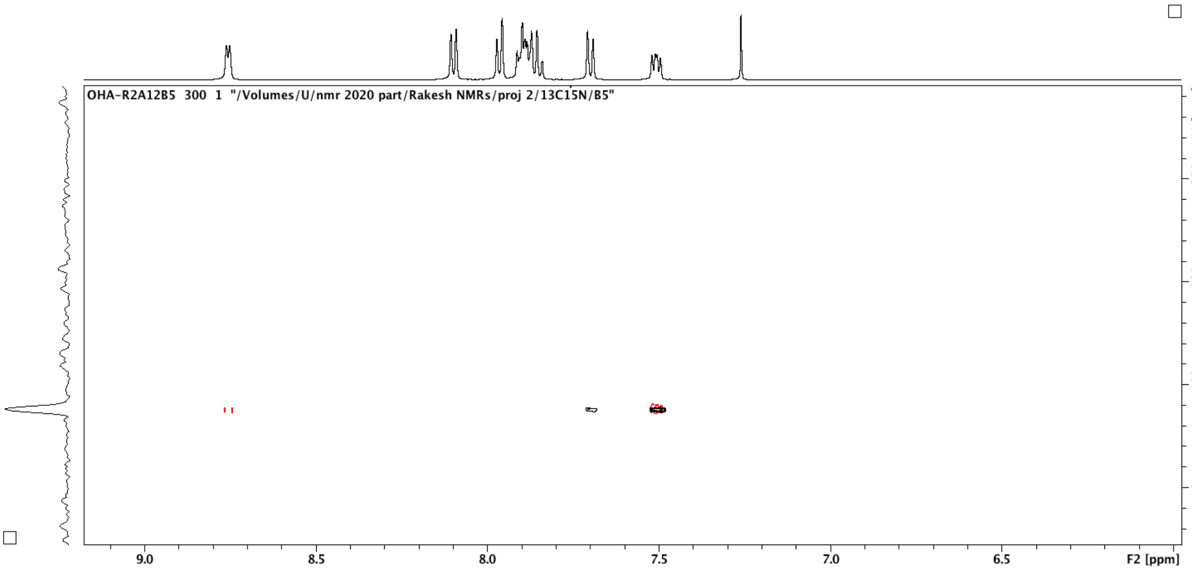

Figure S43. ^{1}H-^{15}N HMBC-NMR Spectra of **6** in black contour and 1:1 complex NBSac-**6** in red contour in $CDCl_3$ at 303K.

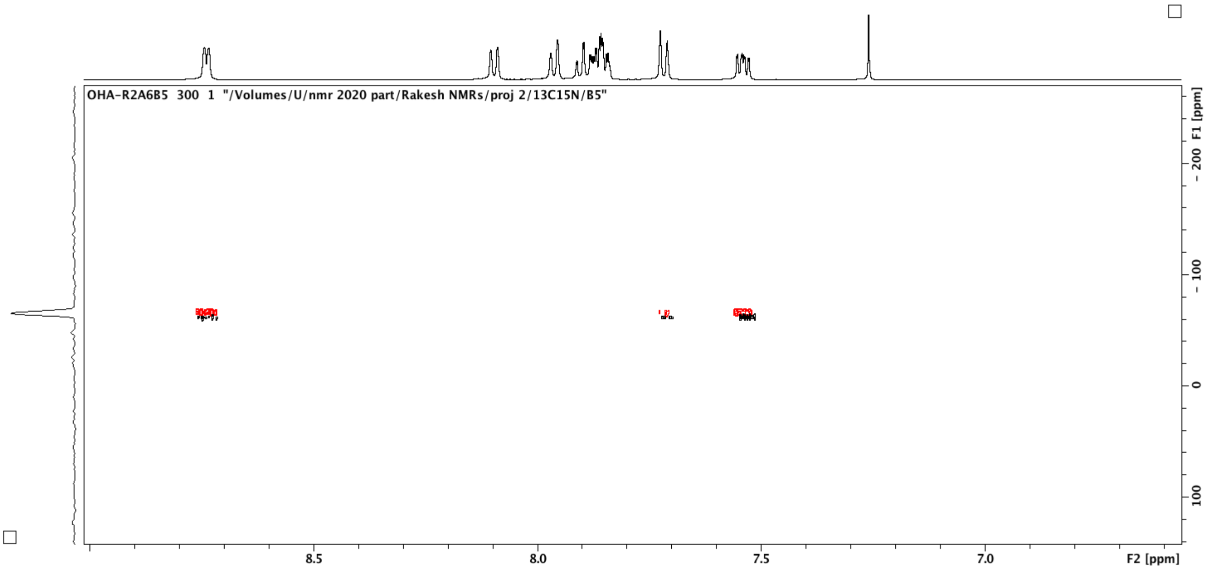

Figure S44. ^{1}H-^{15}N HMBC-NMR Spectra of **7** in black contour and 1:1 complex NBSac-**7** in red contour in $CDCl_3$ at 303K.

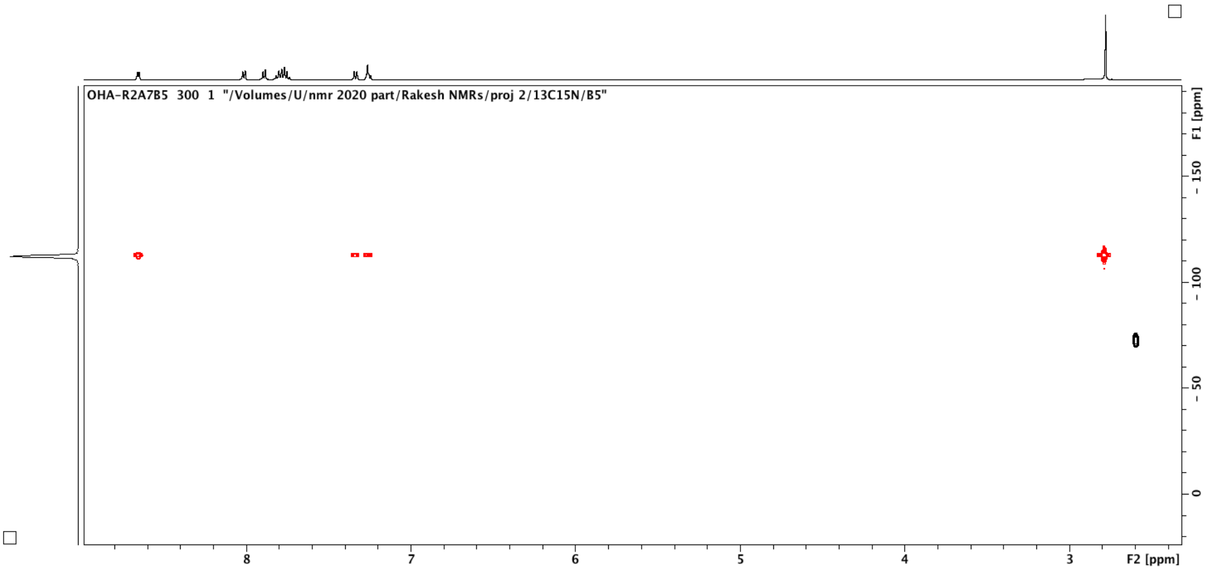

Figure S45. ^{1}H-^{15}N HMBC-NMR Spectra of **8** in black contour and 1:1 complex NBSac-**8** in red contour in $CDCl_3$ at 303K.

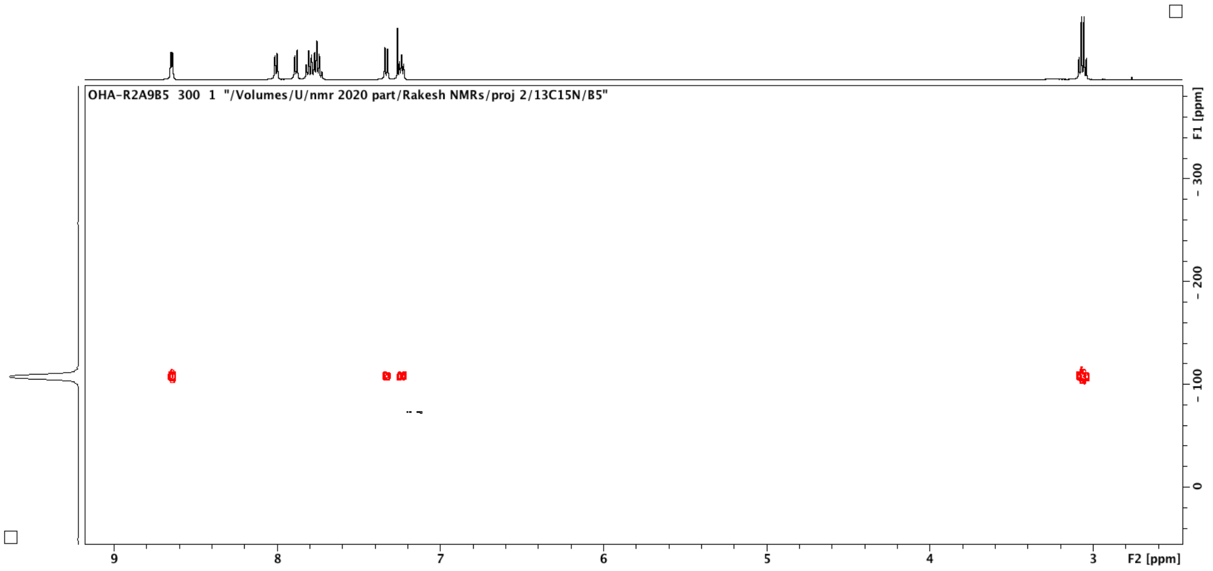

Figure S46. ^{1}H-^{15}N HMBC-NMR Spectra of **9** in black contour and 1:1 complex NBSac-**9** in red contour in $CDCl_3$ at 303K.

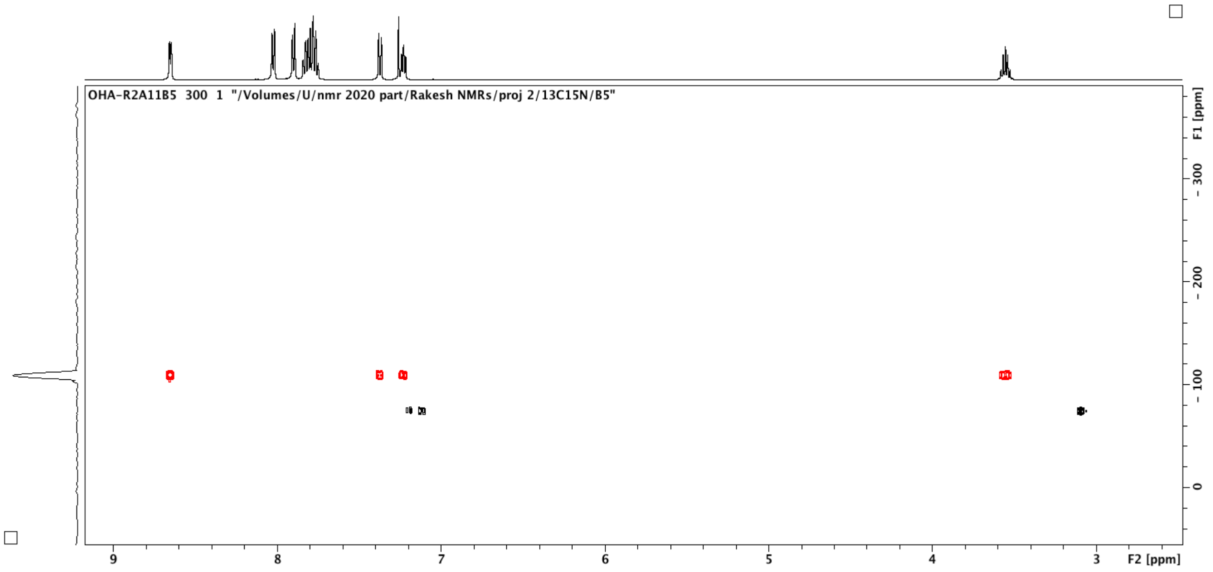

Figure S47. ^{1}H-^{15}N HMBC-NMR Spectra of **10** in black contour and 1:1 complex NBSac-**10** in red contour in $CDCl_3$ at 303K.

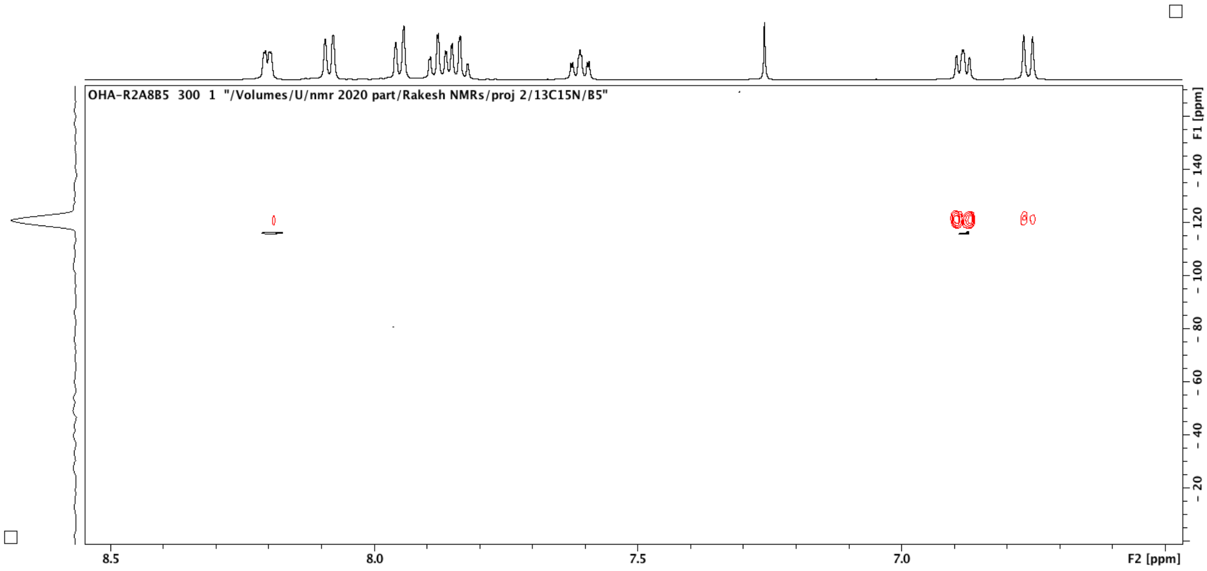

Figure S48. ^{1}H-^{15}N HMBC-NMR Spectra of **11** in black contour and 1:1 complex NBSac-**11** in red contour in $CDCl_3$ at 303K.

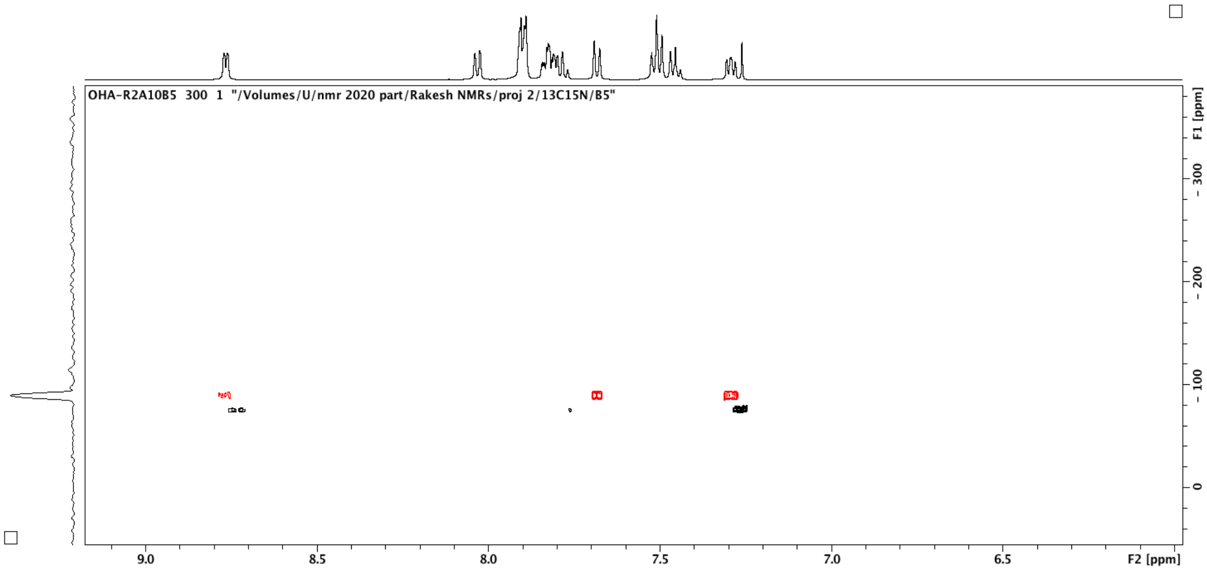

Figure S49. ^{1}H-^{15}N HMBC-NMR Spectra of **12** in black contour and 1:1 complex NBSac-**12** in red contour in $CDCl_3$ at 303K.

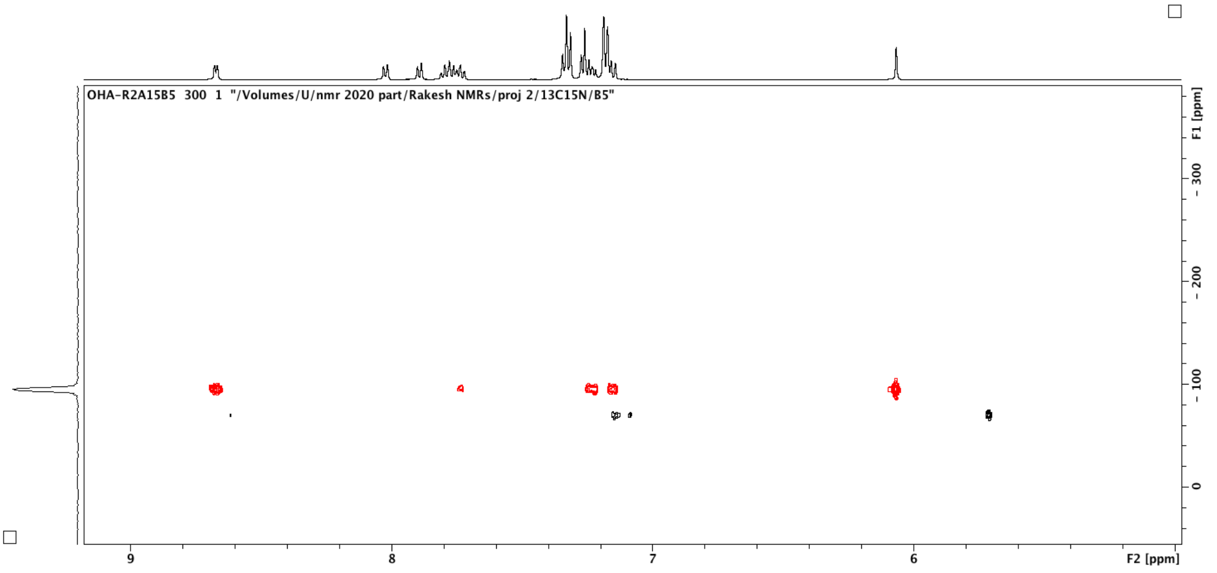

Figure S50. ^{1}H-^{15}N HMBC-NMR Spectra of **13** in black contour and 1:1 complex NBSac-**13** in red contour in $CDCl_3$ at 303K.

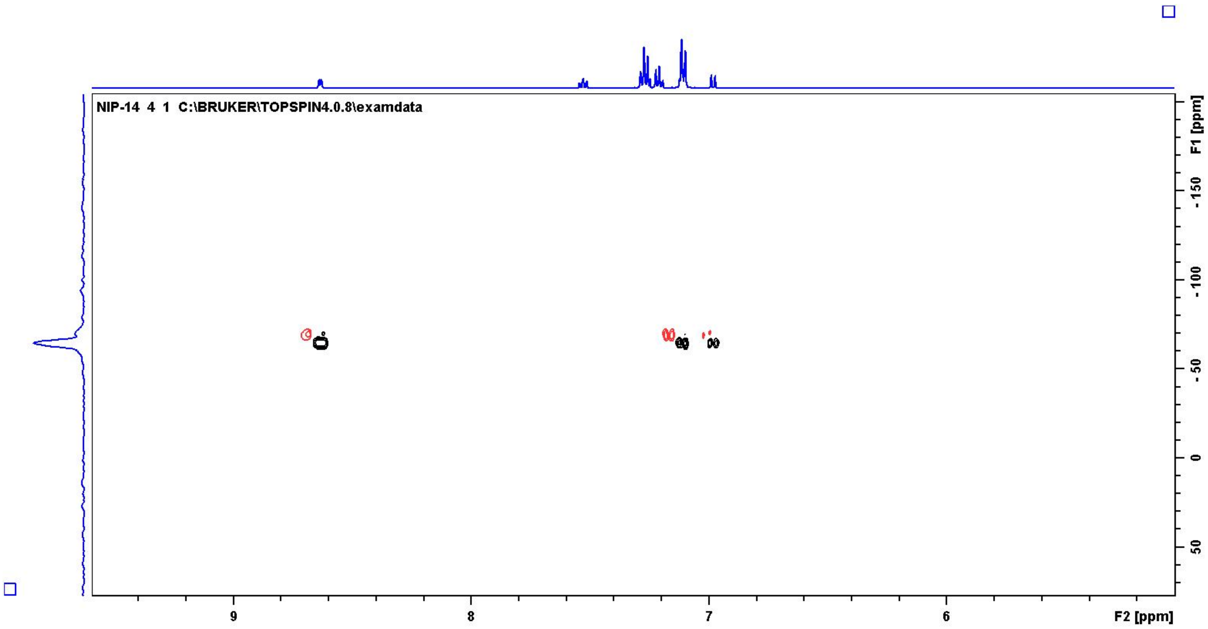

Figure S51. ^{1}H-^{15}N HMBC-NMR Spectra of **14** in black contour and 1:1 complex NBSac-**14** in red contour in $CDCl_3$ at 303K.

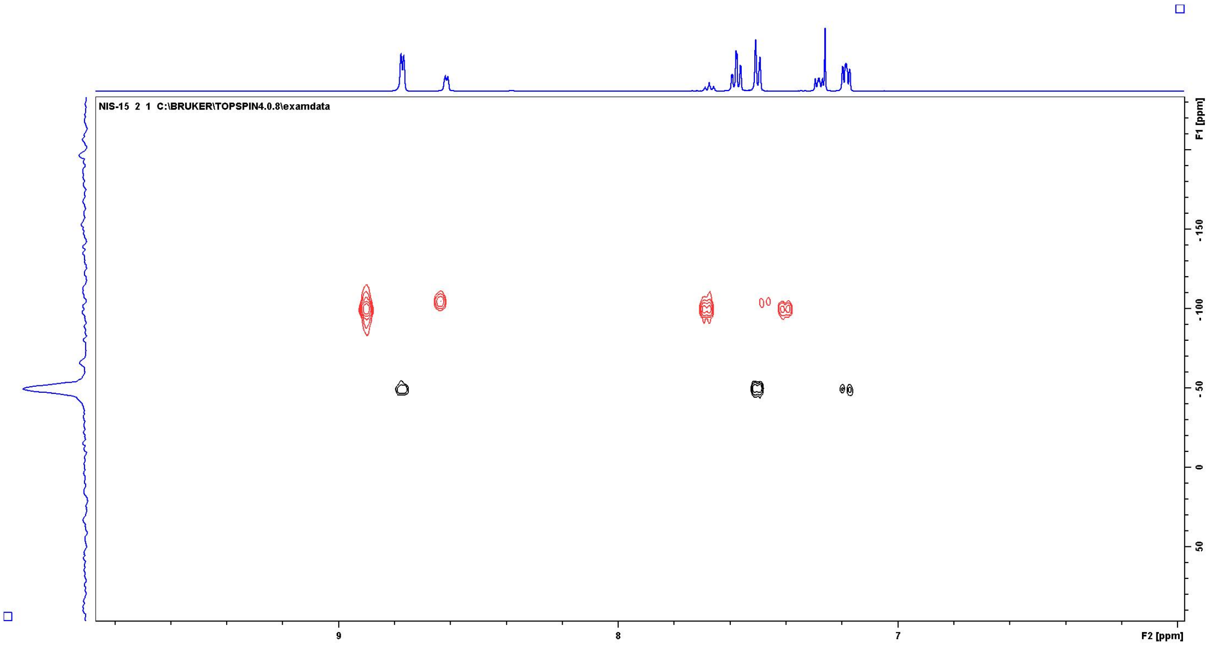

Figure S52. ^{1}H-^{15}N HMBC-NMR Spectra of **15** in black contour and 1:1 complex NBSac-**15** in red contour in $CDCl_3$ at 303K.

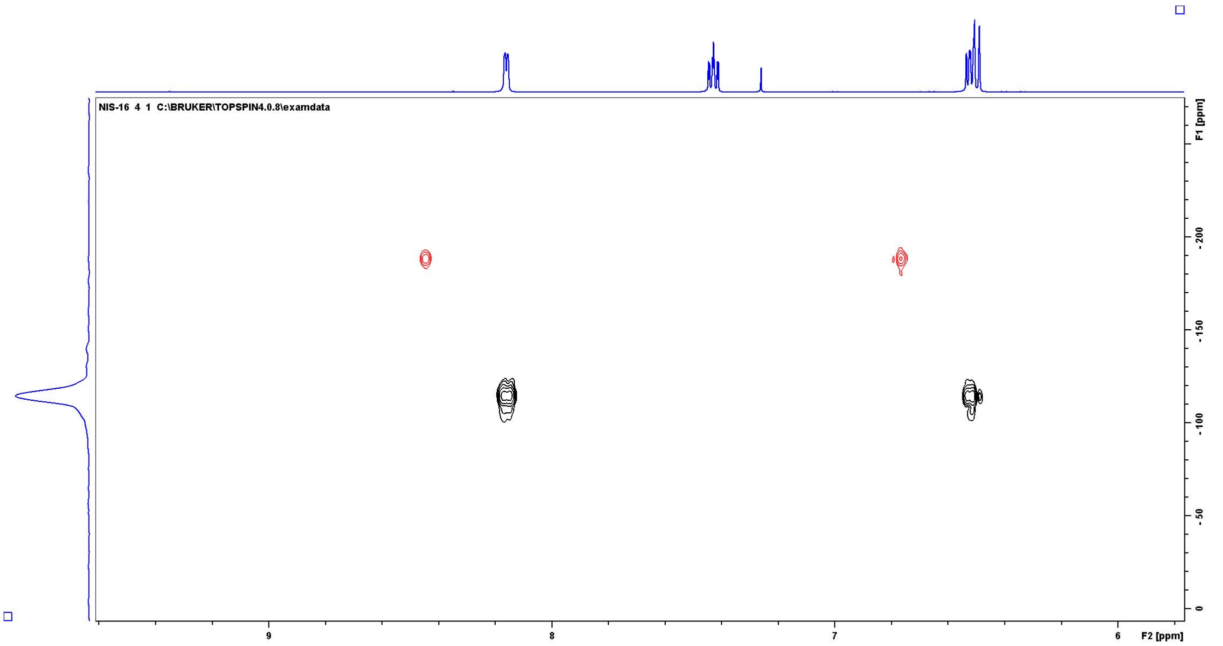

Figure S53. ^{1}H-^{15}N HMBC-NMR Spectra of **16** in black contour and 1:1 complex NBSac-**16** in red contour in $CDCl_3$ at 303K.

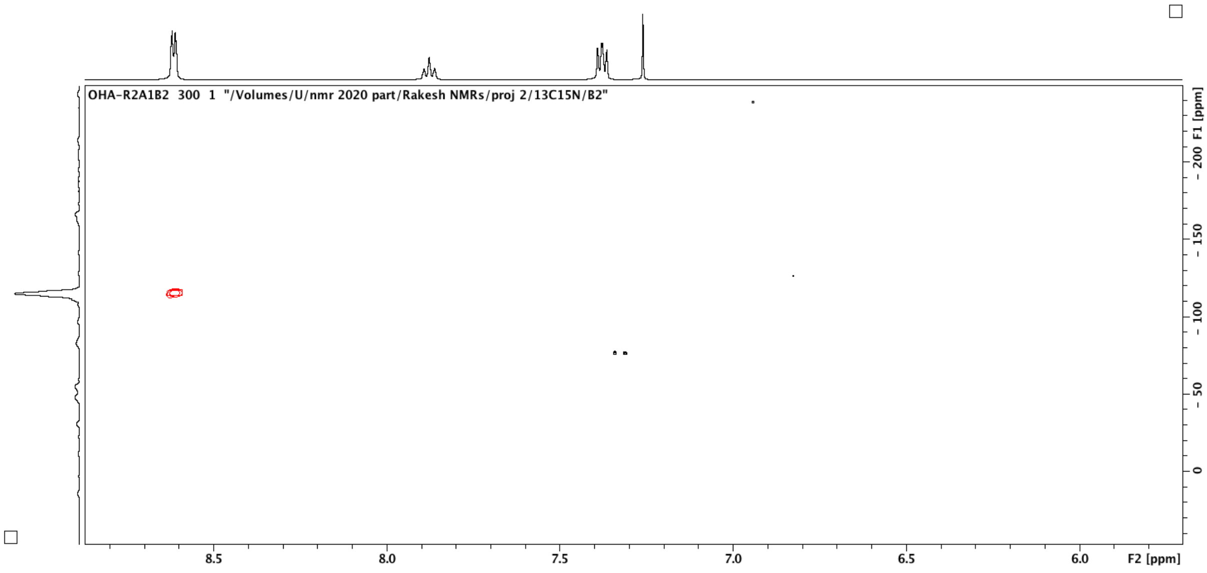

Figure S54. ^{1}H-^{15}N HMBC-NMR Spectra of **1** in black contour and 1:1 complex NIS-**1** in red contour in $CDCl_3$ at 303K.

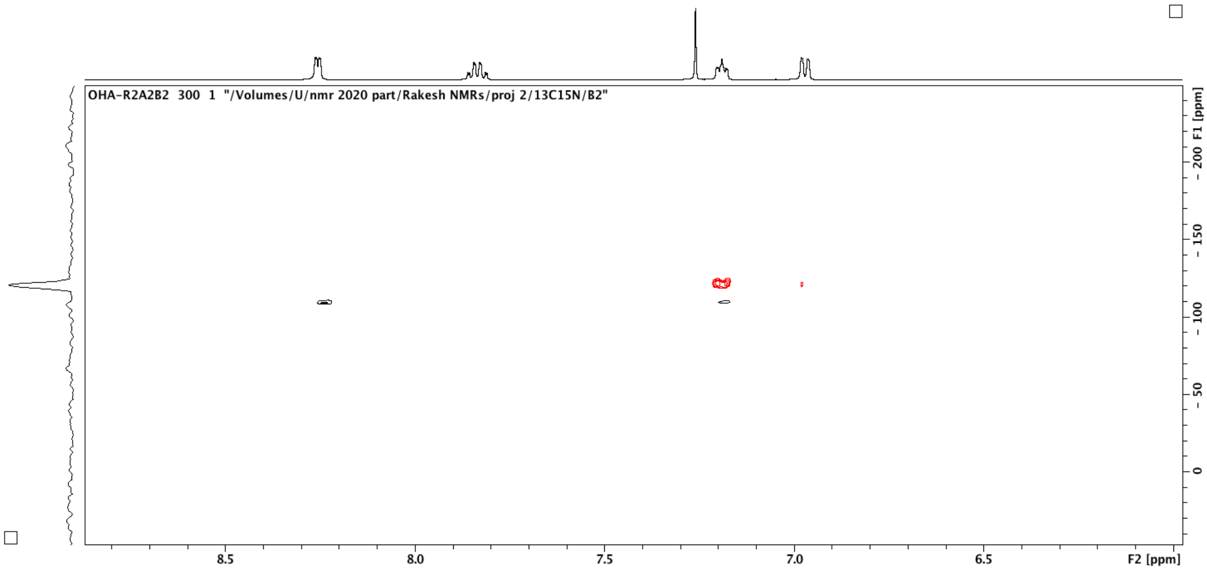

Figure S55. ^{1}H-^{15}N HMBC-NMR Spectra of **2** in black contour and 1:1 complex NIS-**2** in red contour in $CDCl_3$ at 303K.

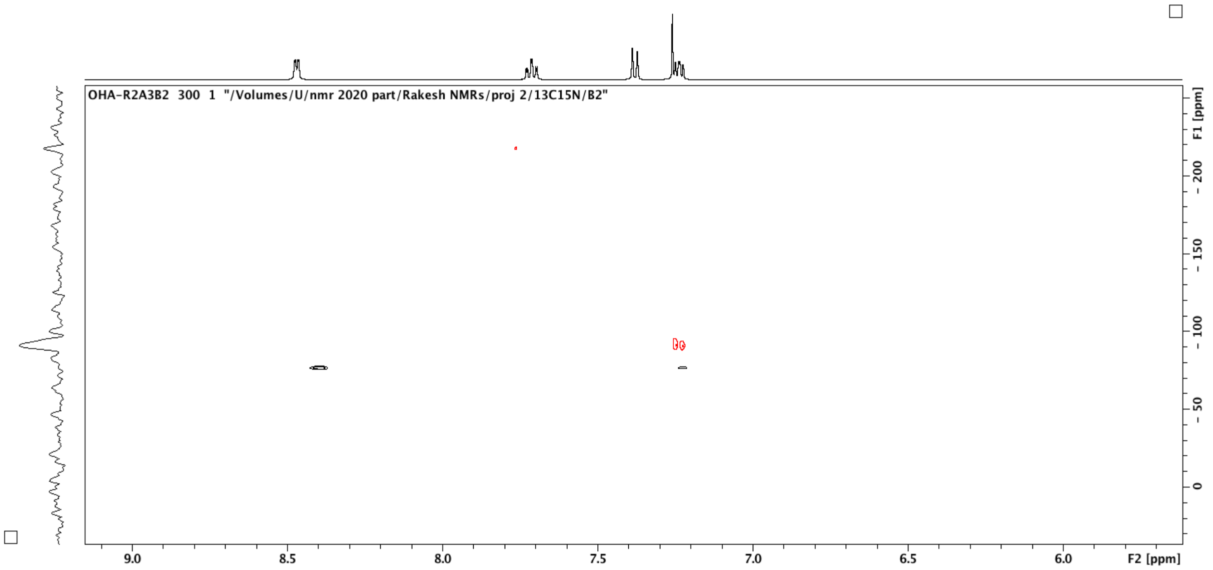

Figure S56. ^{1}H-^{15}N HMBC-NMR Spectra of **3** in black contour and 1:1 complex NIS-**3** in red contour in $CDCl_3$ at 303K.

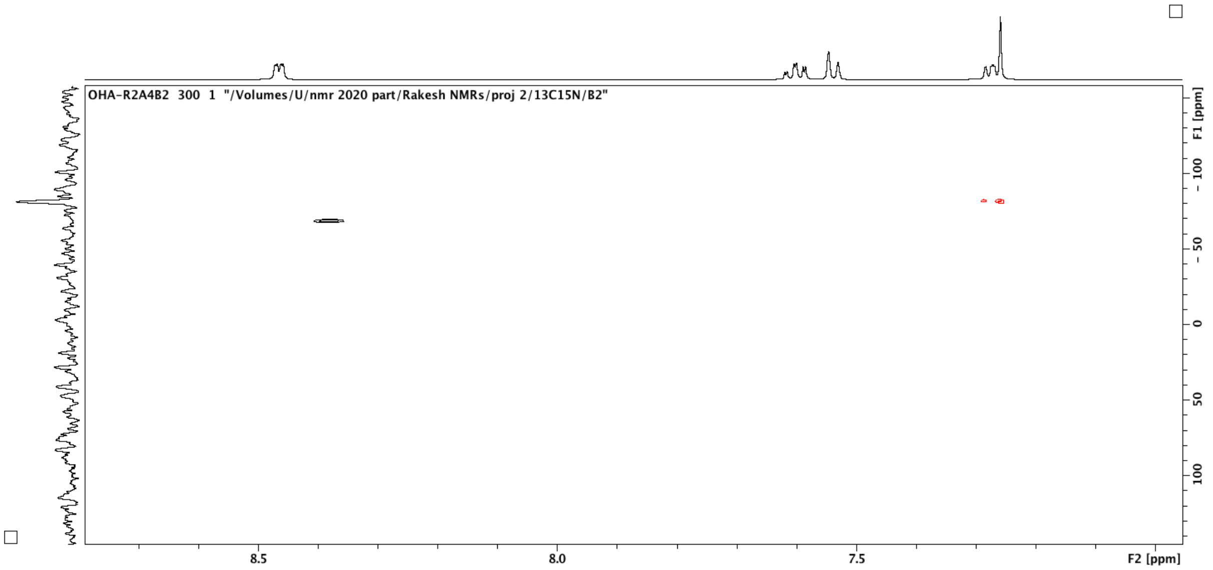

Figure S57. ^{1}H-^{15}N HMBC-NMR Spectra of **4** in black contour and 1:1 complex NIS-**4** in red contour in $CDCl_3$ at 303K.

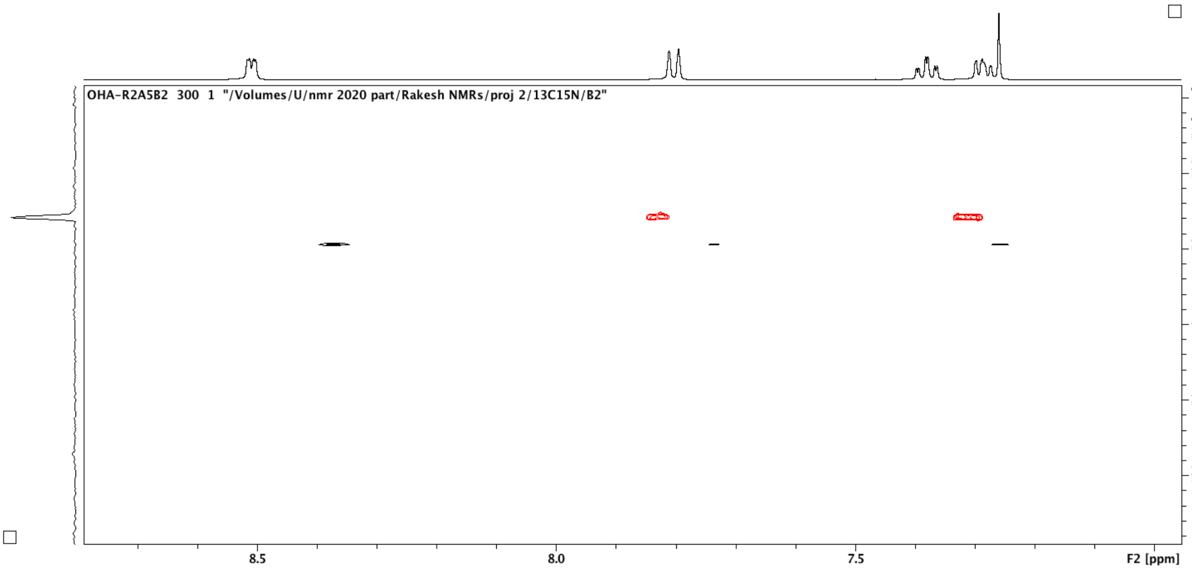

Figure S58. ^{1}H-^{15}N HMBC-NMR Spectra of **5** in black contour and 1:1 complex NIS-**5** in red contour in $CDCl_3$ at 303K.

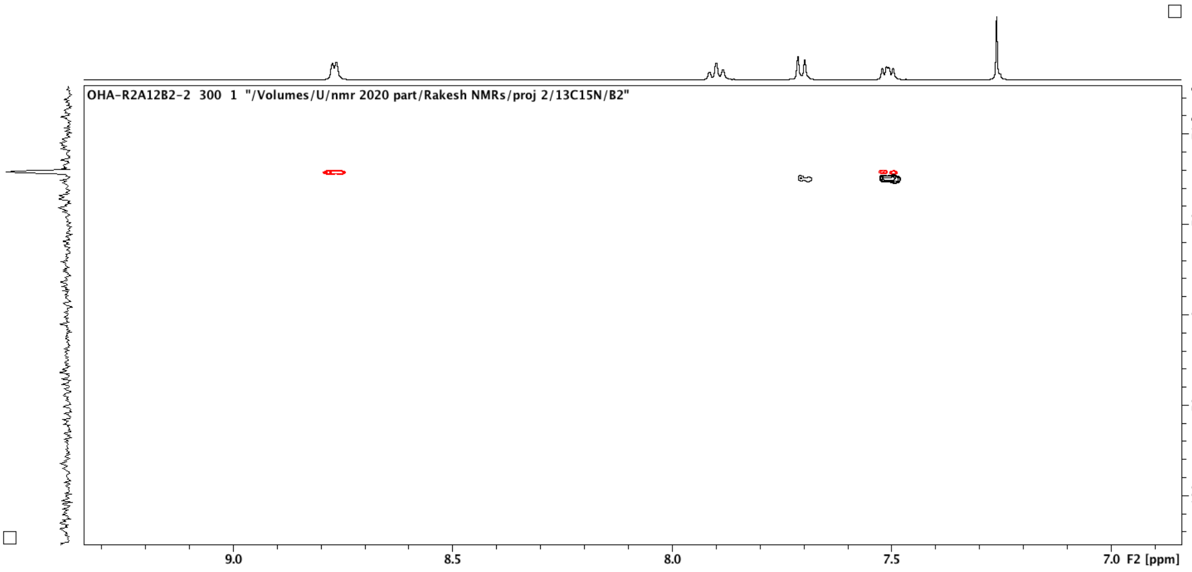

Figure S59. ^{1}H-^{15}N HMBC-NMR Spectra of **6** in black contour and 1:1 complex NIS-**6** in red contour in $CDCl_3$ at 303K.

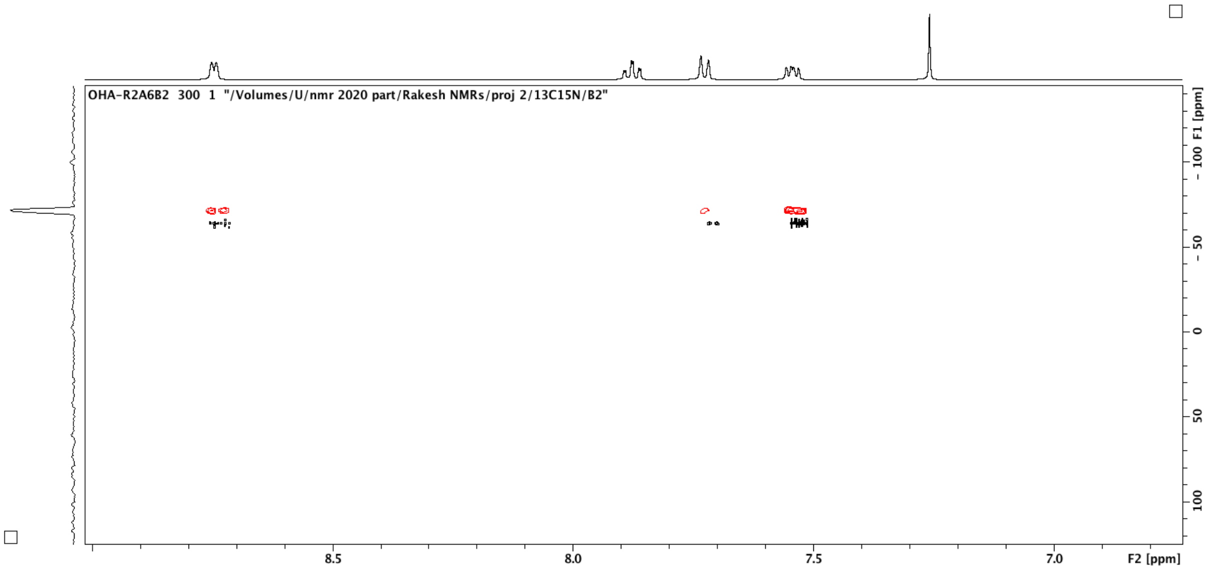

Figure S60. ^{1}H-^{15}N HMBC-NMR Spectra of **7** in black contour and 1:1 complex NIS-**7** in red contour in $CDCl_3$ at 303K.

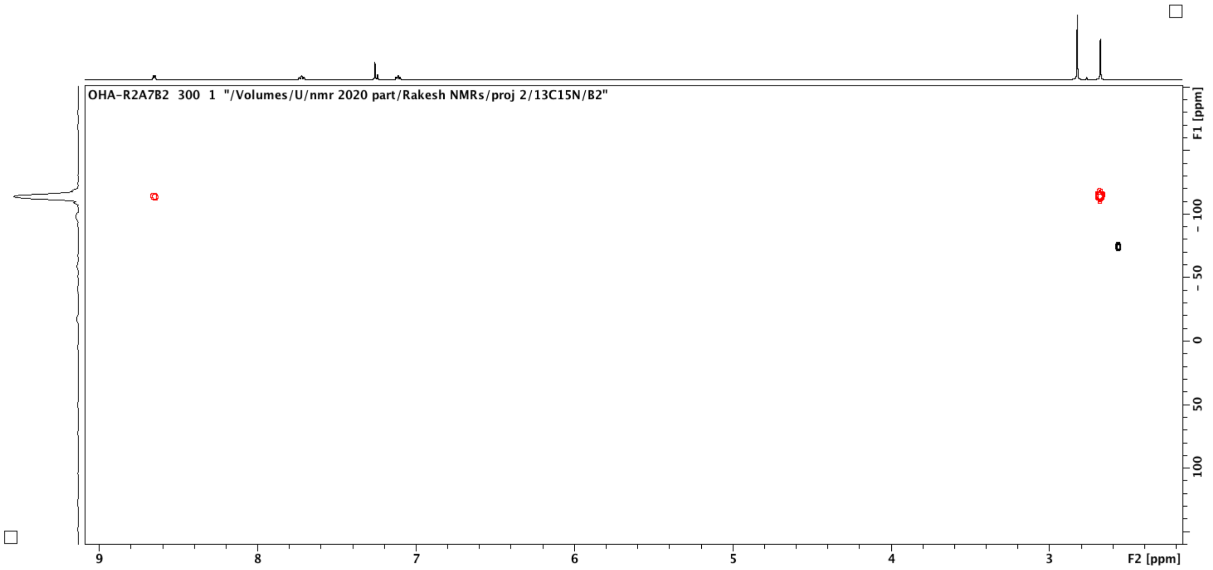

Figure S61. ^{1}H-^{15}N HMBC-NMR Spectra of **8** in black contour and 1:1 complex NIS-**8** in red contour in $CDCl_3$ at 303K.

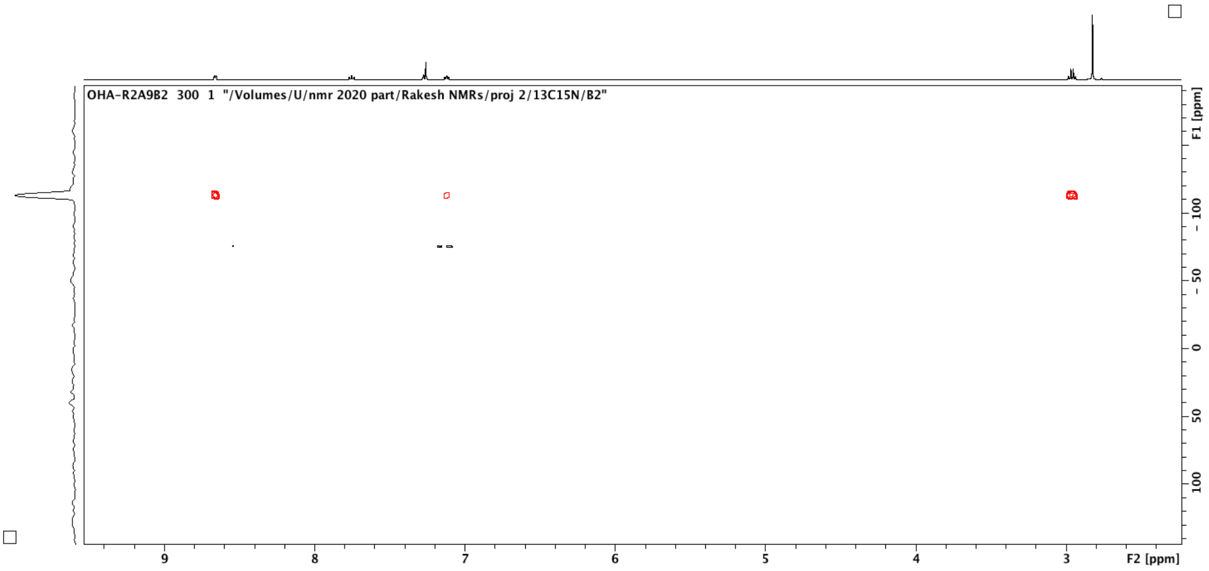

Figure S62. ^{1}H-^{15}N HMBC-NMR Spectra of **9** in black contour and 1:1 complex NIS-**9** in red contour in $CDCl_3$ at 303K.

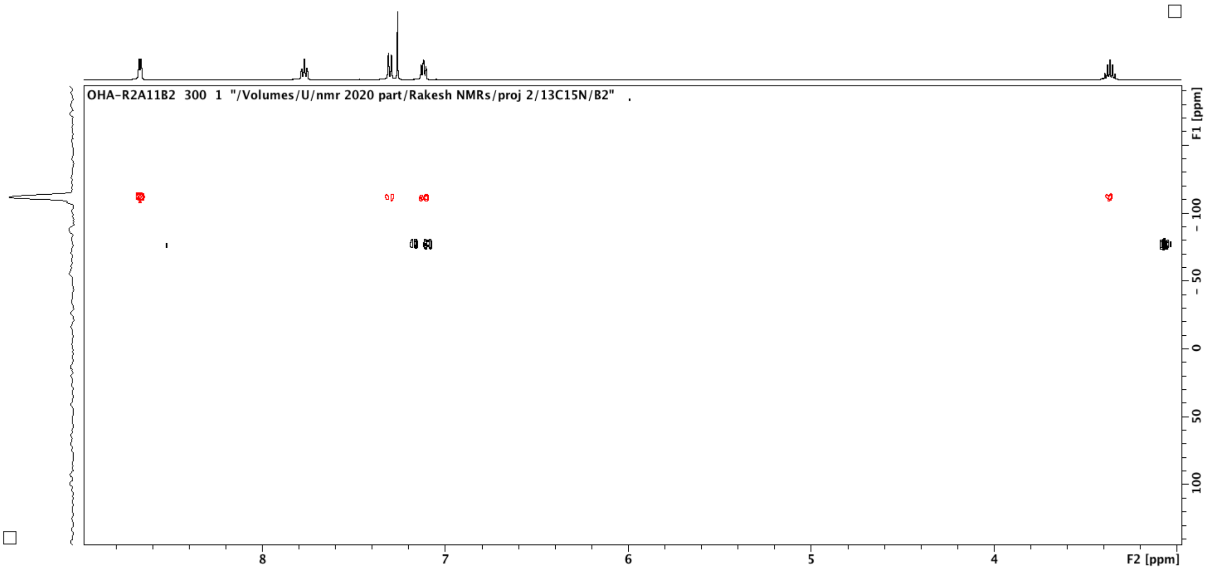

Figure S63. ^{1}H-^{15}N HMBC-NMR Spectra of **10** in black contour and 1:1 complex NIS-**10** in red contour in $CDCl_3$ at 303K.

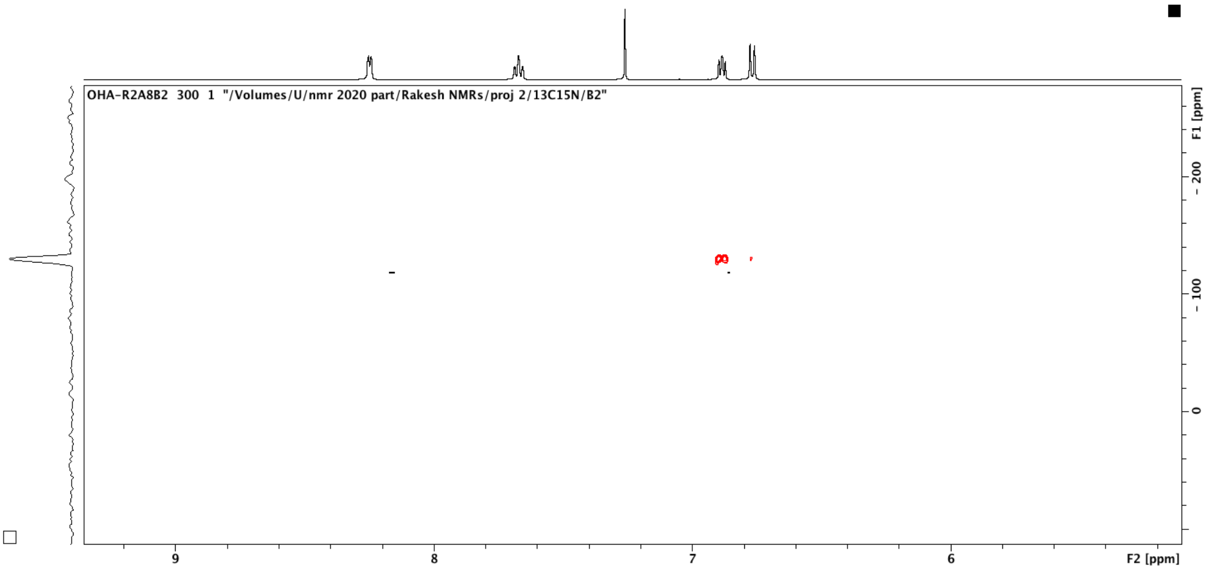

Figure S64. ^{1}H-^{15}N HMBC-NMR Spectra of **11** in black contour and 1:1 complex NIS-**11** in red contour in $CDCl_3$ at 303K.

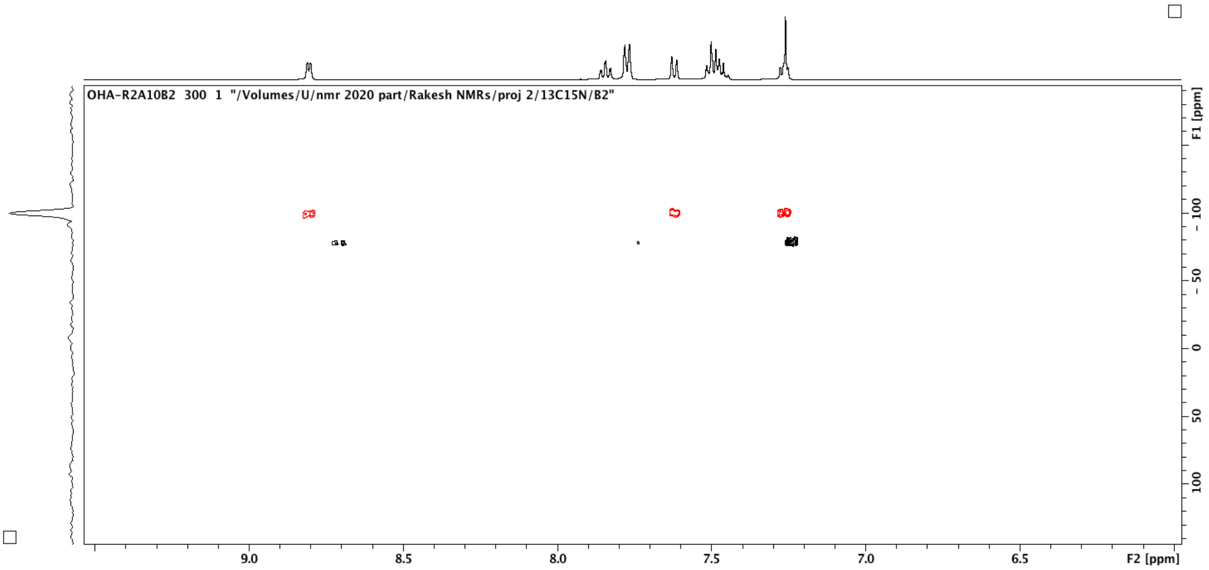

Figure S65. ^{1}H-^{15}N HMBC-NMR Spectra of **12** in black contour and 1:1 complex NIS-**12** in red contour in $CDCl_3$ at 303K.

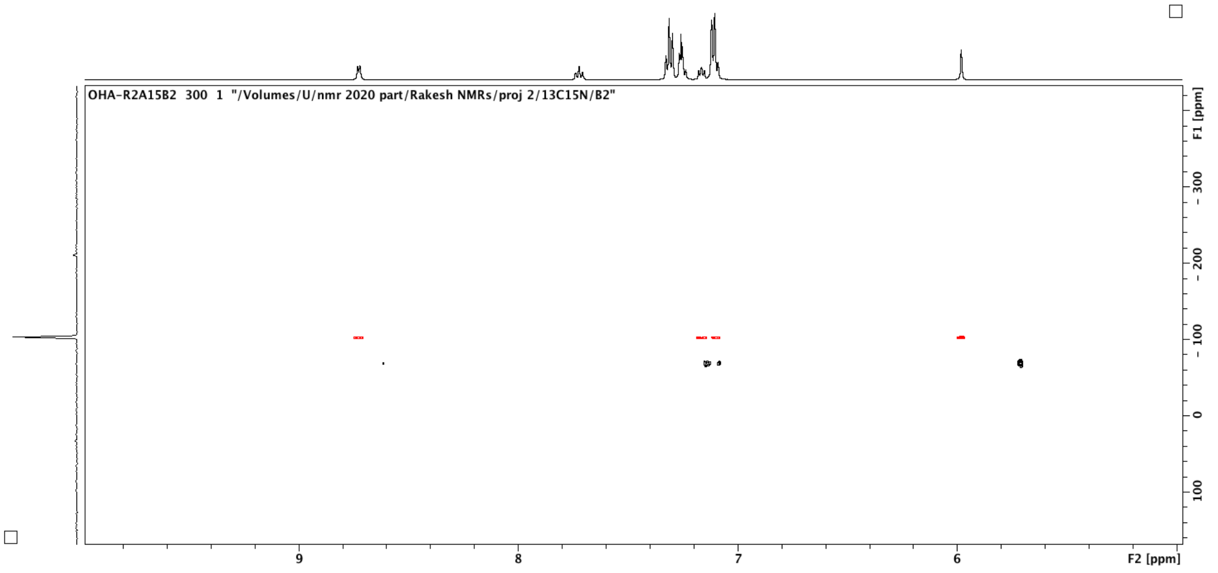

Figure S66. ^{1}H-^{15}N HMBC-NMR Spectra of **13** in black contour and 1:1 complex NIS-**13** in red contour in $CDCl_3$ at 303K.

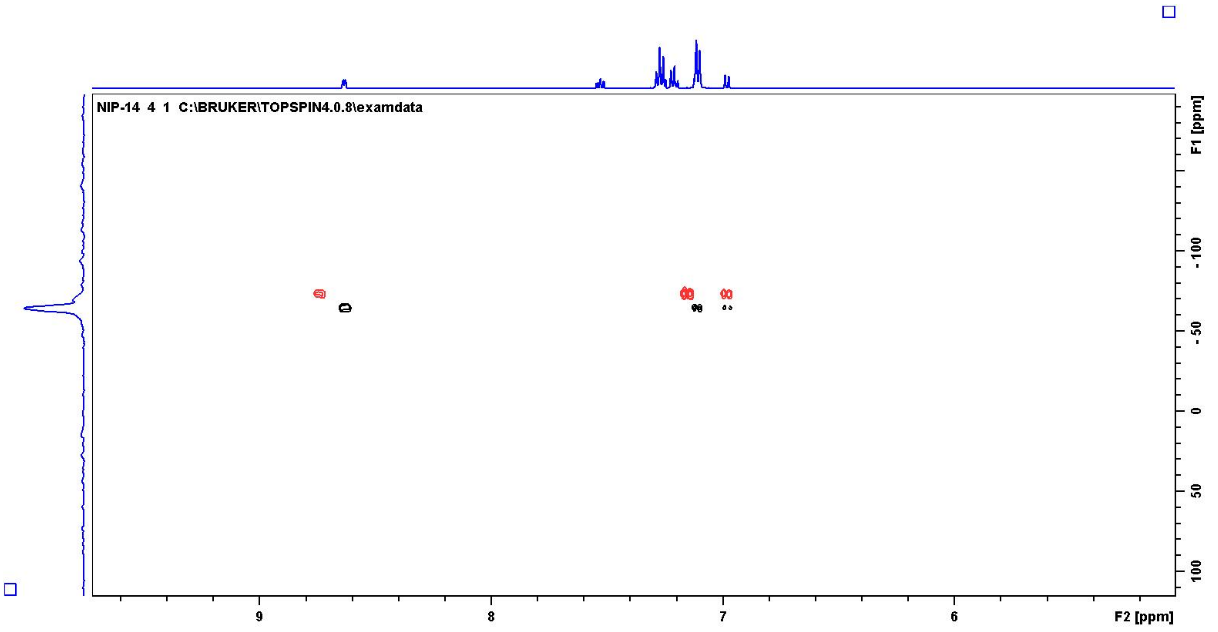

Figure S67. ^{1}H-^{15}N HMBC-NMR Spectra of **14** in black contour and 1:1 complex NIS-**14** in red contour in $CDCl_3$ at 303K.

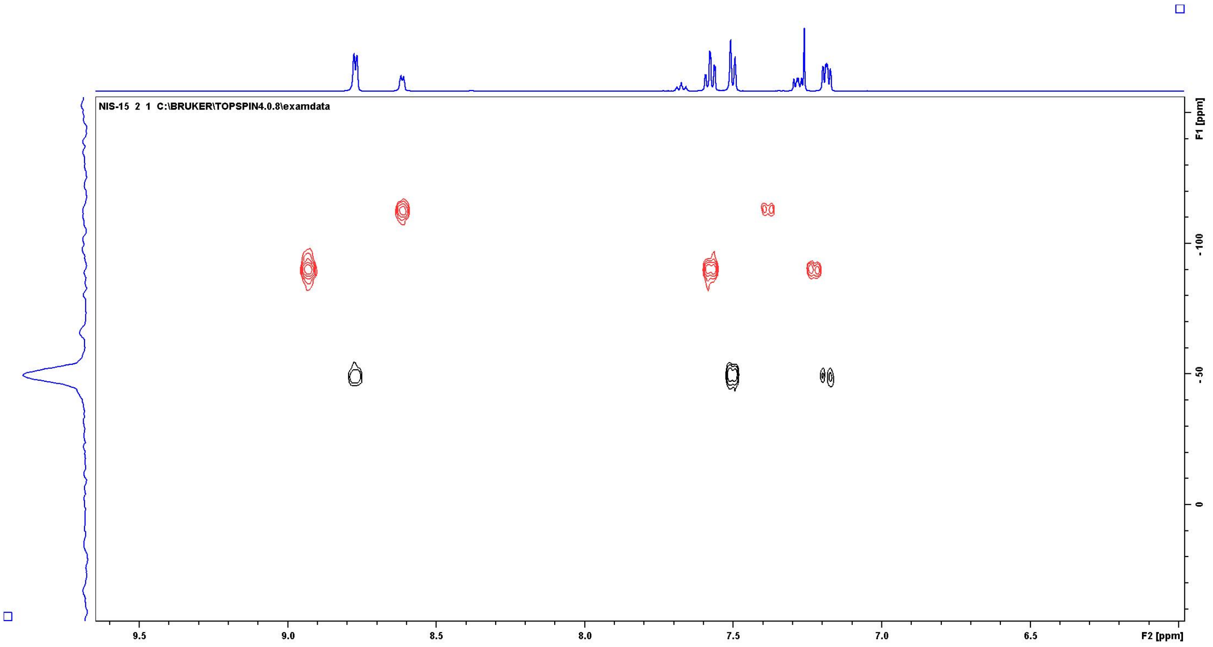

Figure S68. ^{1}H-^{15}N HMBC-NMR Spectra of **15** in black contour and 1:1 complex NIS-**15** in red contour in $CDCl_3$ at 303K.

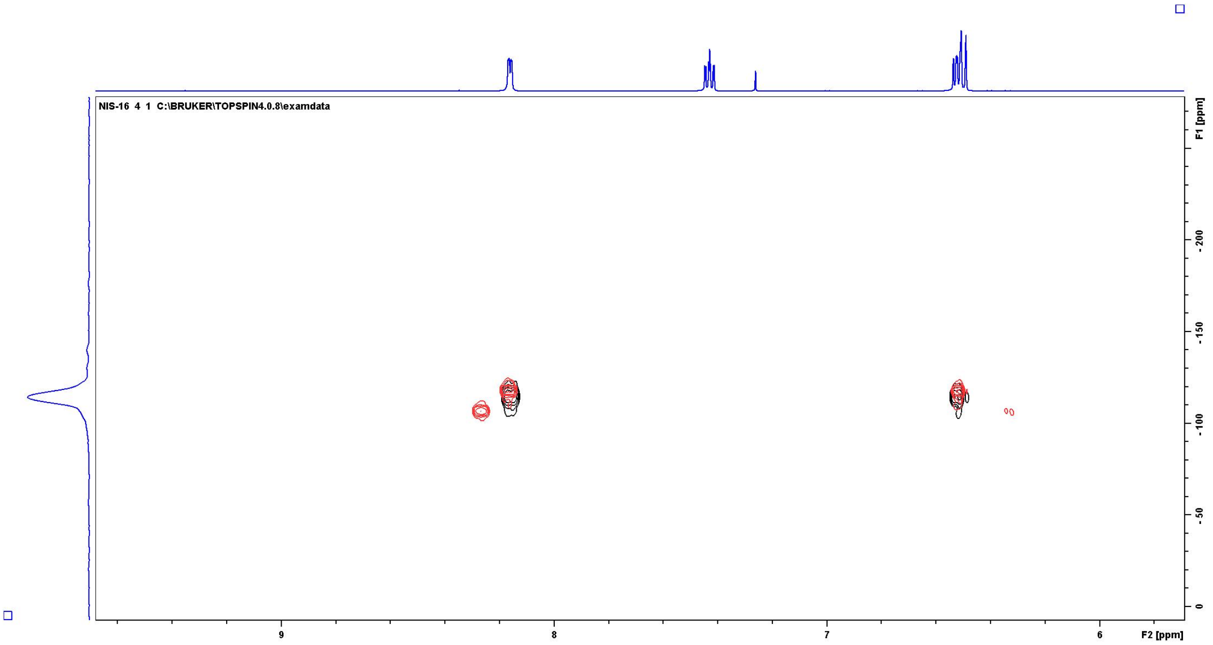

Figure S69. ^{1}H-^{15}N HMBC-NMR Spectra of **16** in black contour and 1:1 complex NIS-**16** in red contour in $CDCl_3$ at 303K.

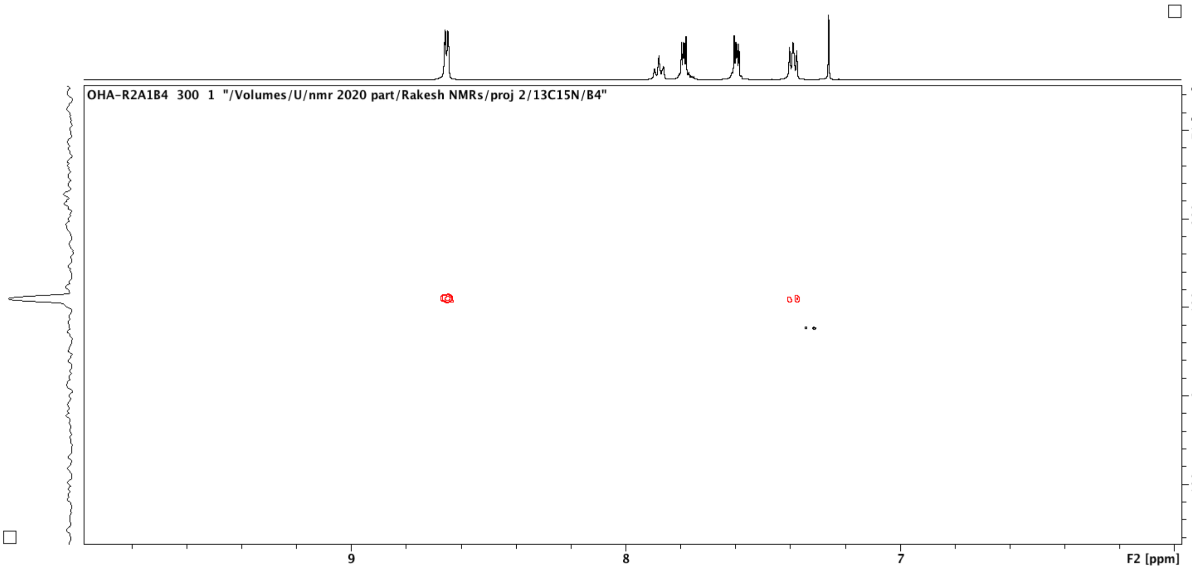

Figure S70. ^{1}H-^{15}N HMBC-NMR Spectra of **1** in black contour and 1:1 complex NIP-**1** in red contour in $CDCl_3$ at 303K.

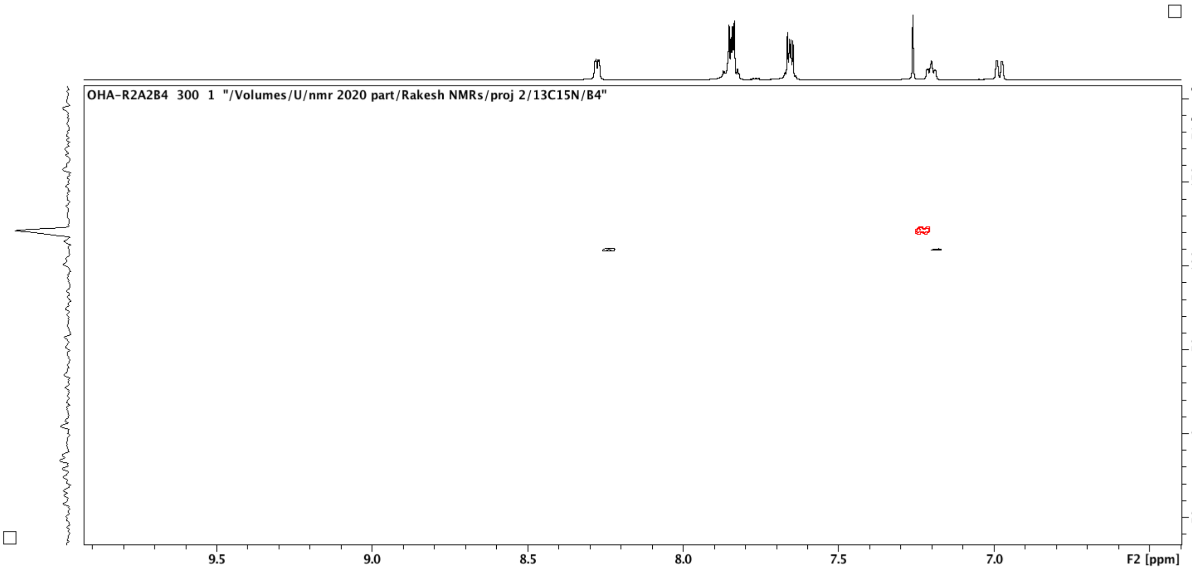

Figure S71. ^{1}H-^{15}N HMBC-NMR Spectra of **2** in black contour and 1:1 complex NIP-**2** in red contour in $CDCl_3$ at 303K.

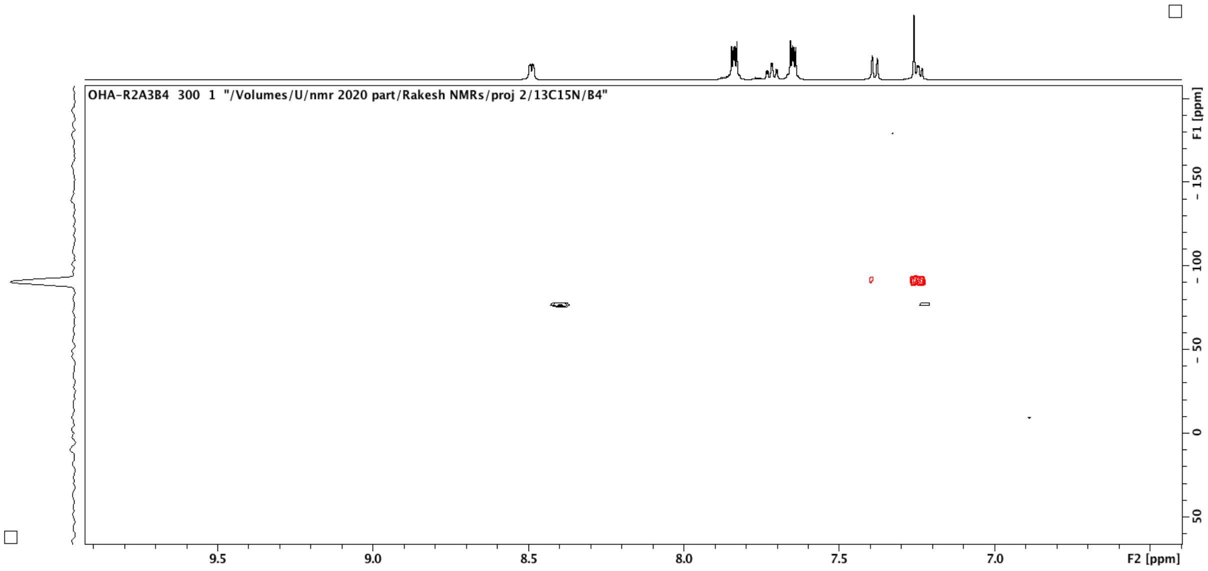

Figure S72. ^{1}H-^{15}N HMBC-NMR Spectra of **3** in black contour and 1:1 complex NIP-**3** in red contour in $CDCl_3$ at 303K.

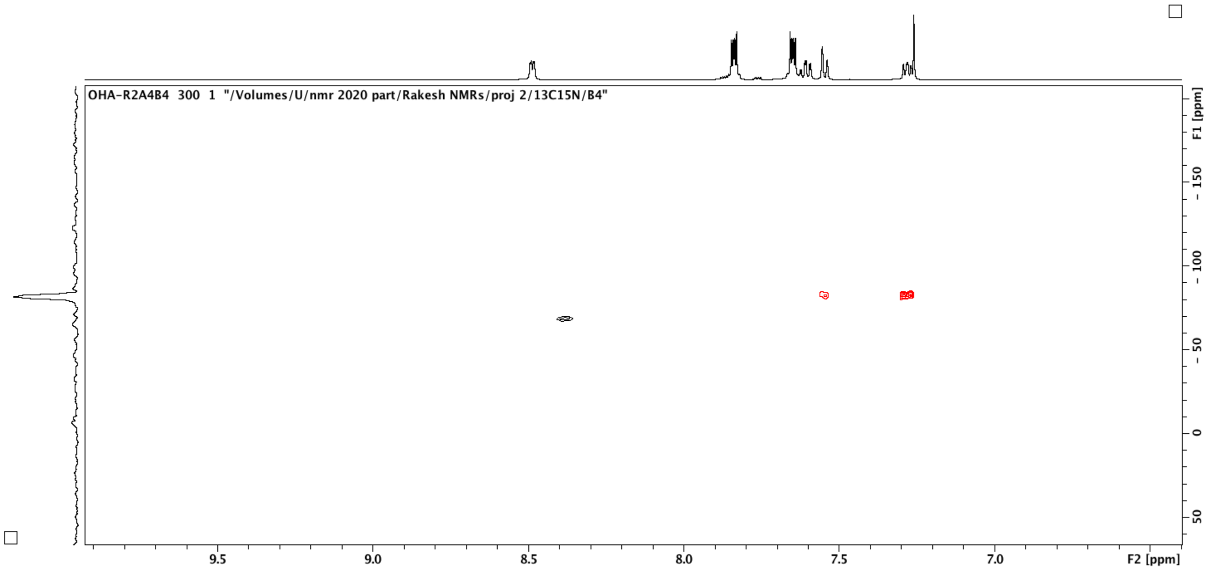

Figure S73. ^{1}H-^{15}N HMBC-NMR Spectra of **4** in black contour and 1:1 complex NIP-**4** in red contour in $CDCl_3$ at 303K.

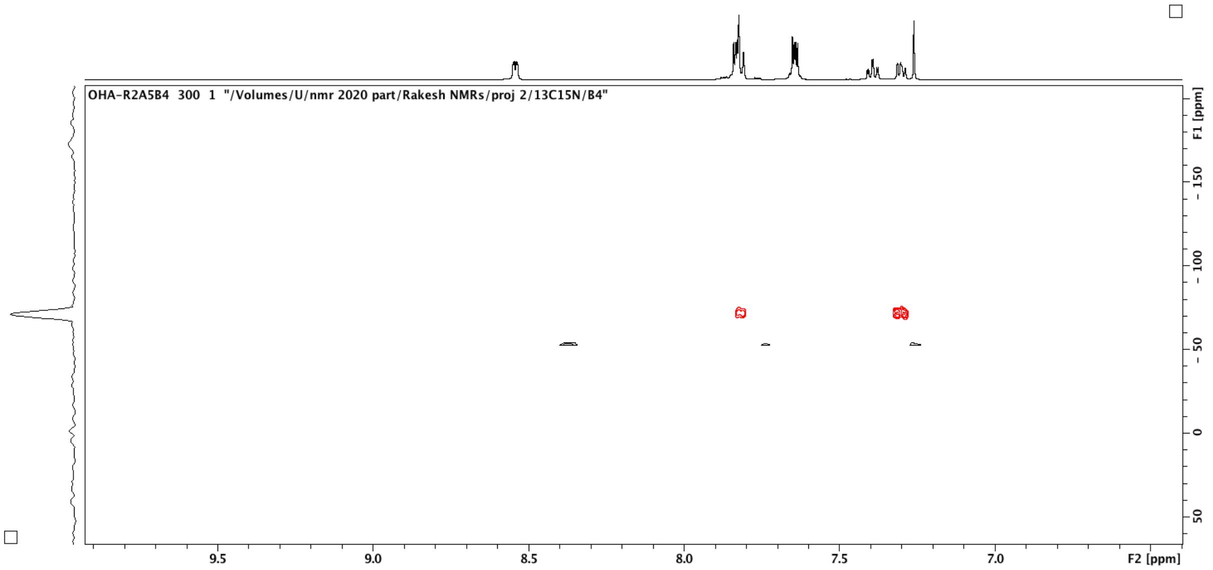

Figure S74. ^{1}H-^{15}N HMBC-NMR Spectra of **5** in black contour and 1:1 complex NIP-**5** in red contour in $CDCl_3$ at 303K.

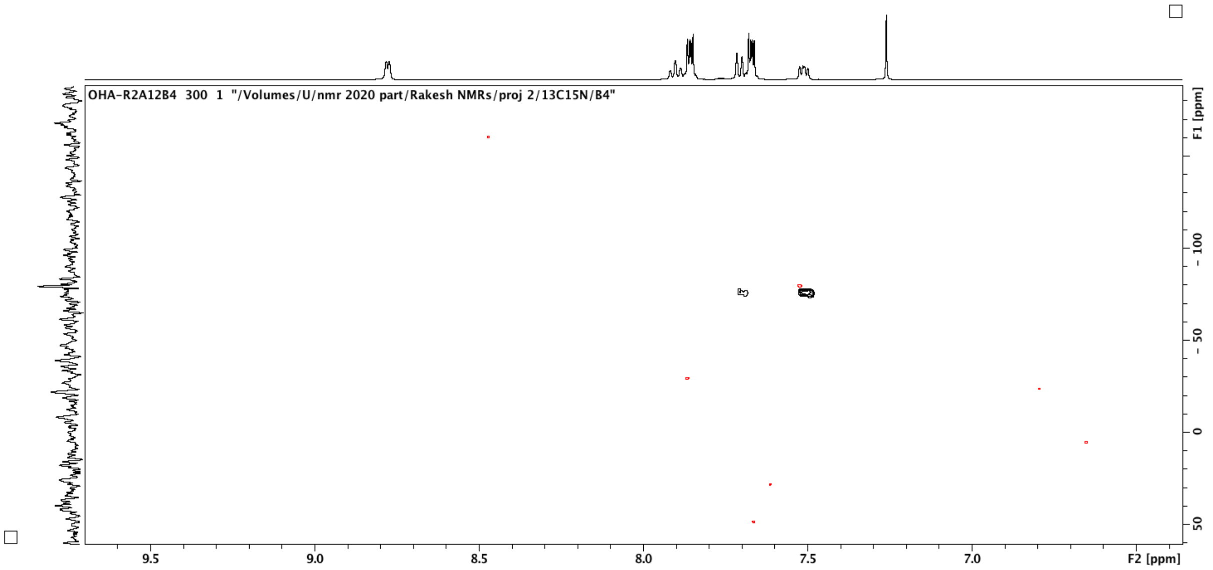

Figure S75. ^{1}H-^{15}N HMBC-NMR Spectra of Compound **6** in black contour and 1:1 complex NIP-**6** in red contour in $CDCl_3$ at 303K.

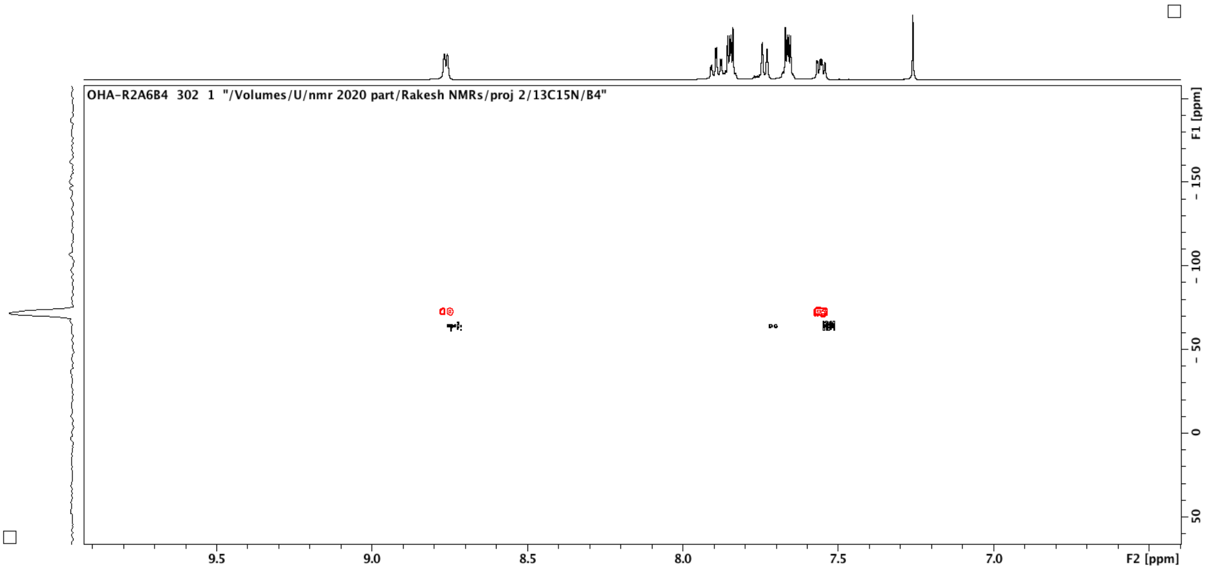

Figure S76. ^{1}H-^{15}N HMBC-NMR Spectra of Compound **7** in black contour and 1:1 complex NIP-**7** in red contour in $CDCl_3$ at 303K.

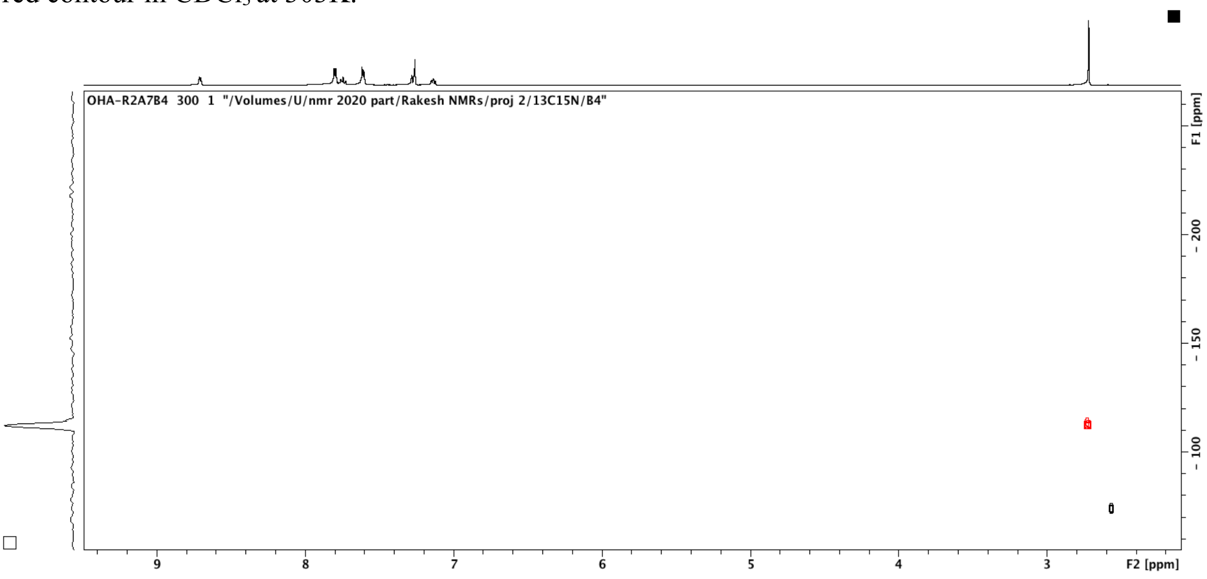

Figure S77. ^{1}H-^{15}N HMBC-NMR Spectra of **8** in black contour and 1:1 complex NIP-**8** in red contour in $CDCl_3$ at 303K.

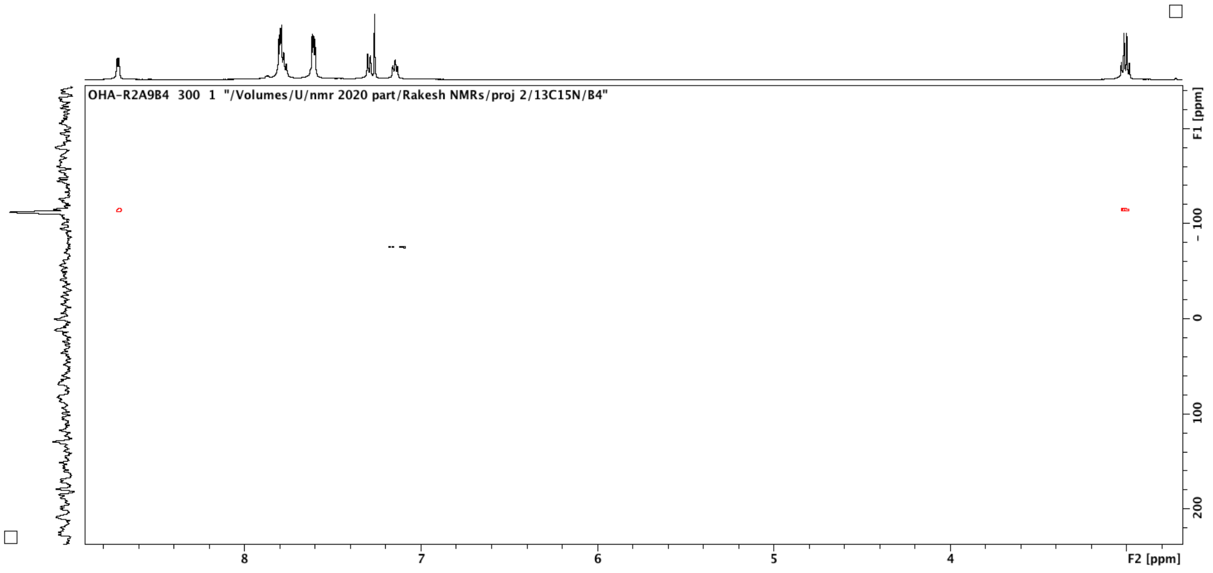

Figure S78. ^{1}H-^{15}N HMBC-NMR Spectra of **9** in black contour and 1:1 complex NIP-**9** in red contour in $CDCl_3$ at 303K.

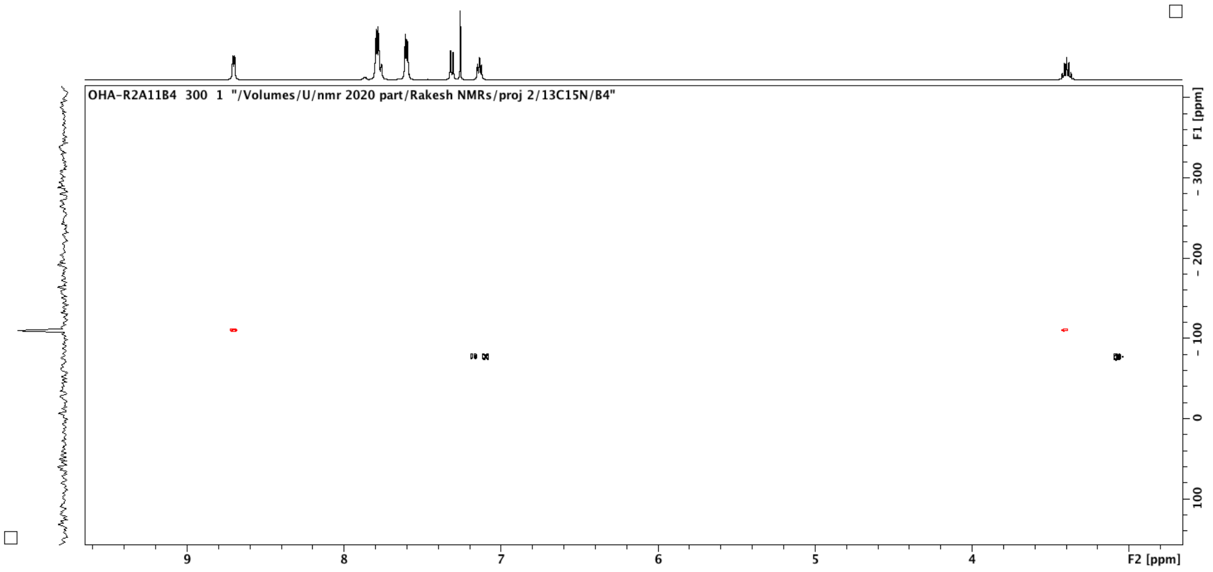

Figure S79. ^{1}H-^{15}N HMBC-NMR Spectra of **10** in black contour and 1:1 complex NIP-**10** in red contour in $CDCl_3$ at 303K.

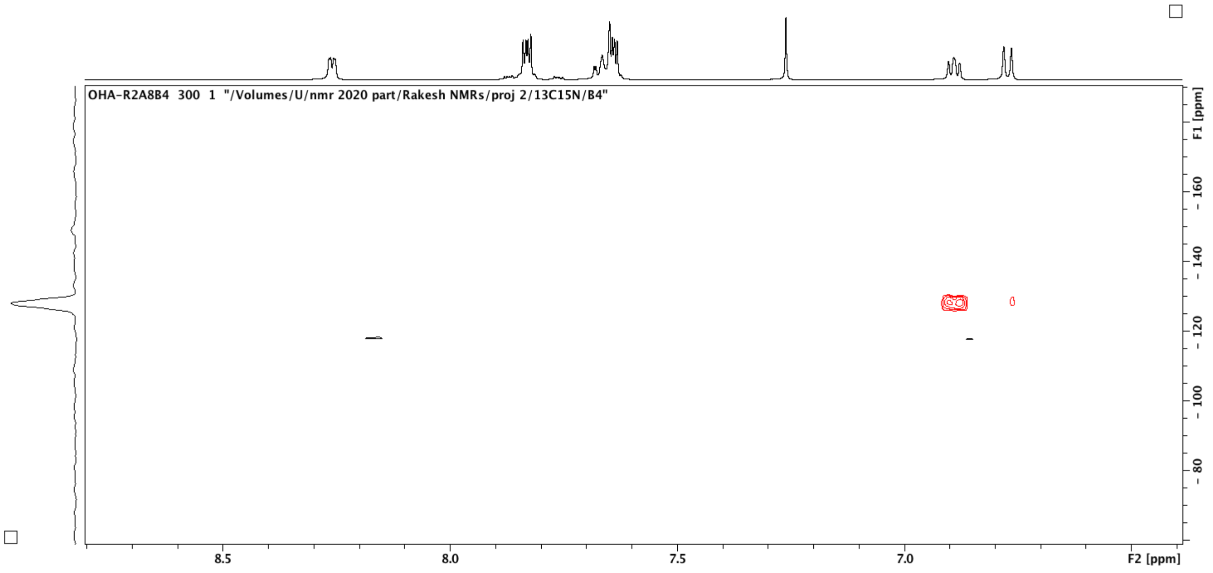

Figure S80. ^{1}H-^{15}N HMBC-NMR Spectra of **11** in black contour and 1:1 complex NIP-**11** in red contour in $CDCl_3$ at 303K.

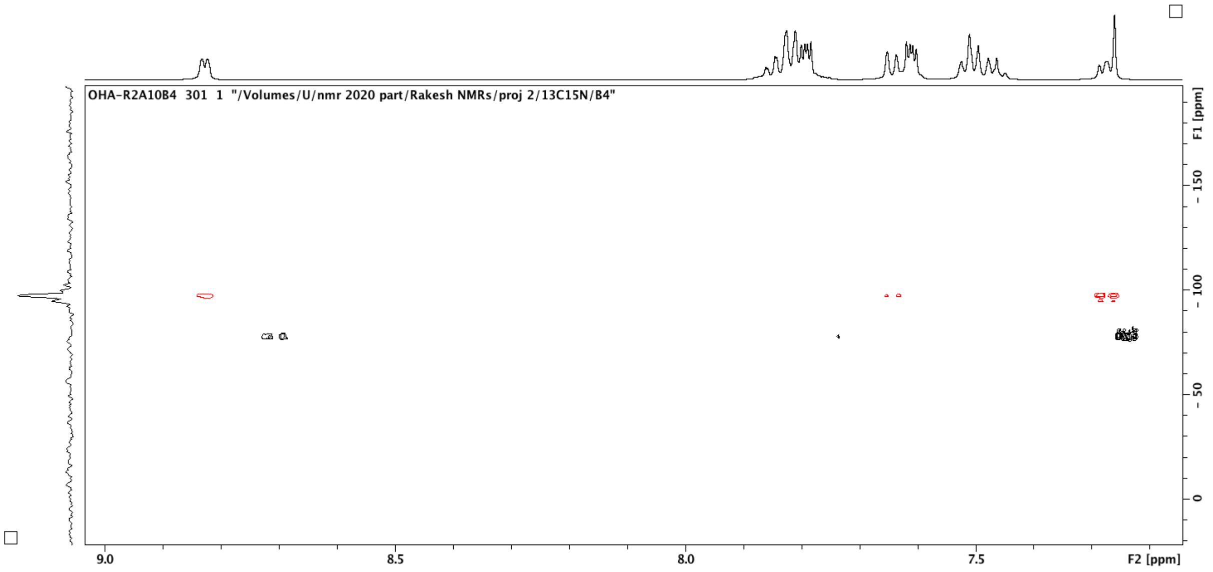

Figure S81. ^{1}H-^{15}N HMBC-NMR Spectra of **12** in black contour and 1:1 complex NIP-**12** in red contour in $CDCl_3$ at 303K.

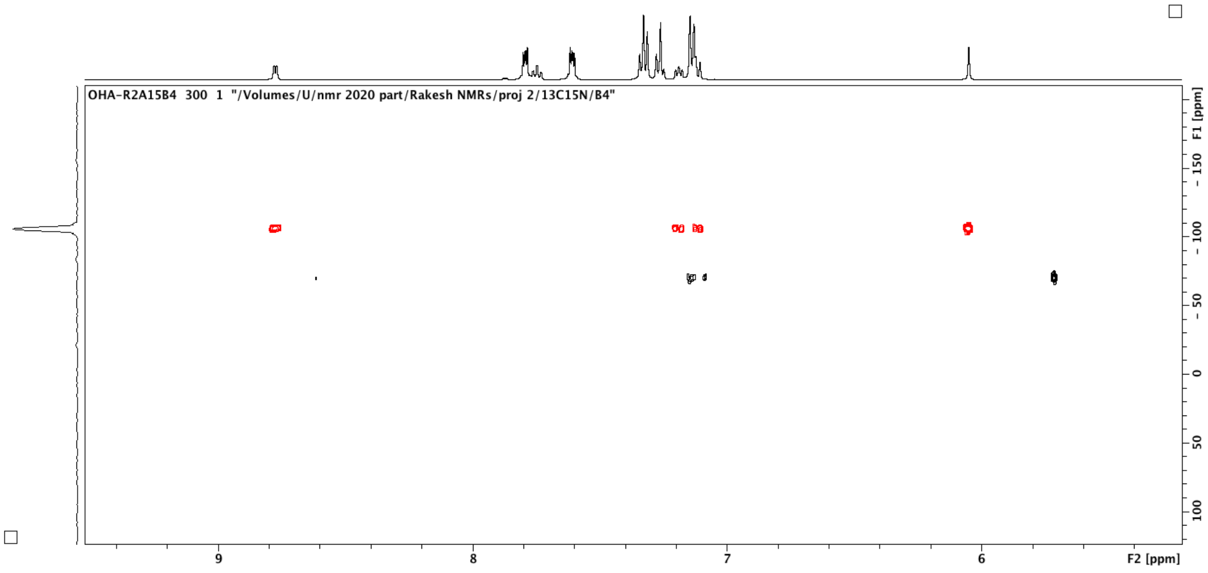

Figure S82. ^{1}H-^{15}N HMBC-NMR Spectra of **13** in black contour and 1:1 complex NIP-**13** in red contour in $CDCl_3$ at 303K.

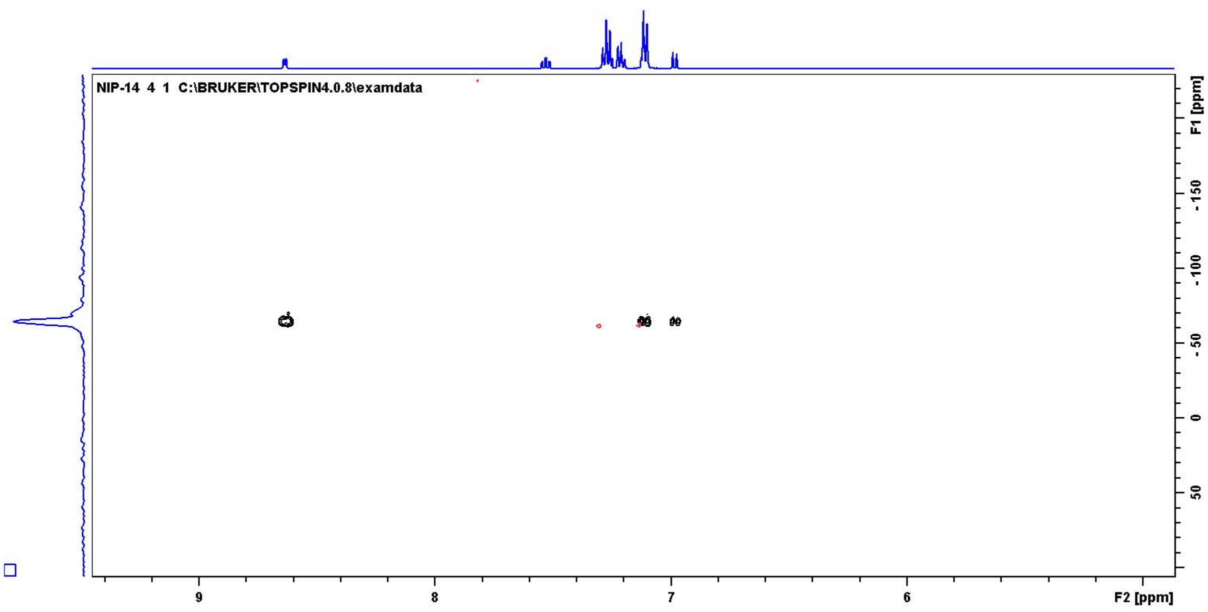

Figure S83. ^{1}H-^{15}N HMBC-NMR Spectra of **14** in black contour and 1:1 complex NIP-**14** in red contour in $CDCl_3$ at 303K.

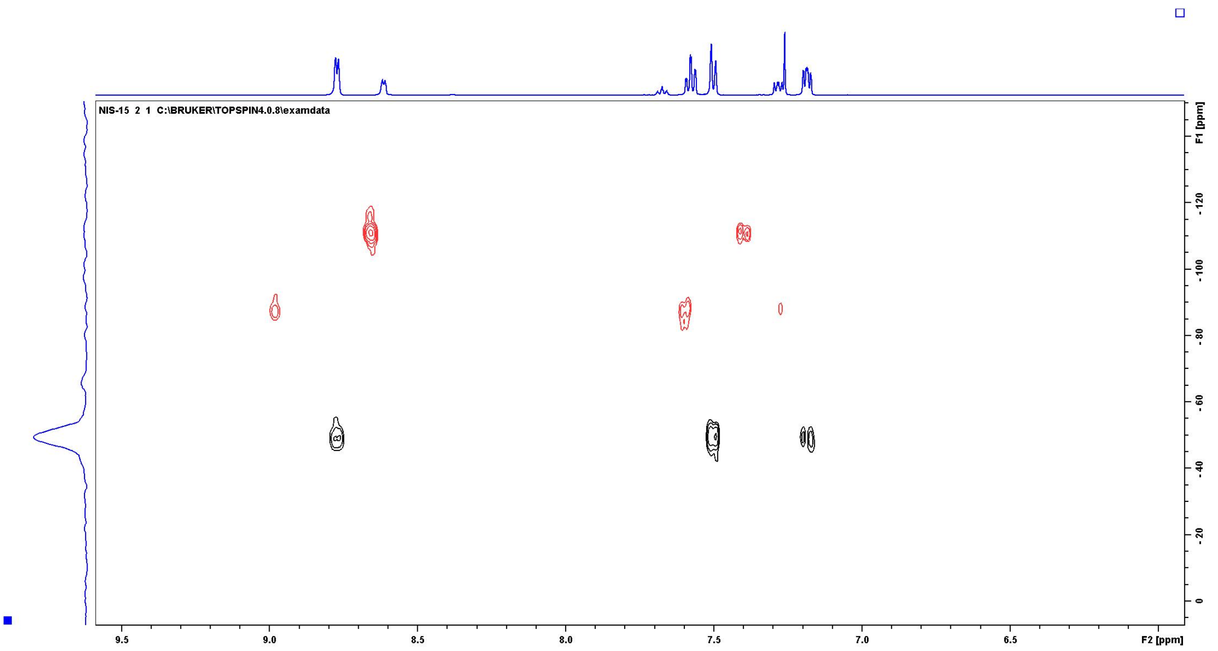

Figure S84. ^{1}H-^{15}N HMBC-NMR Spectra of **15** in black contour and 1:1 complex NIP-**15** in red contour in $CDCl_3$ at 303K.

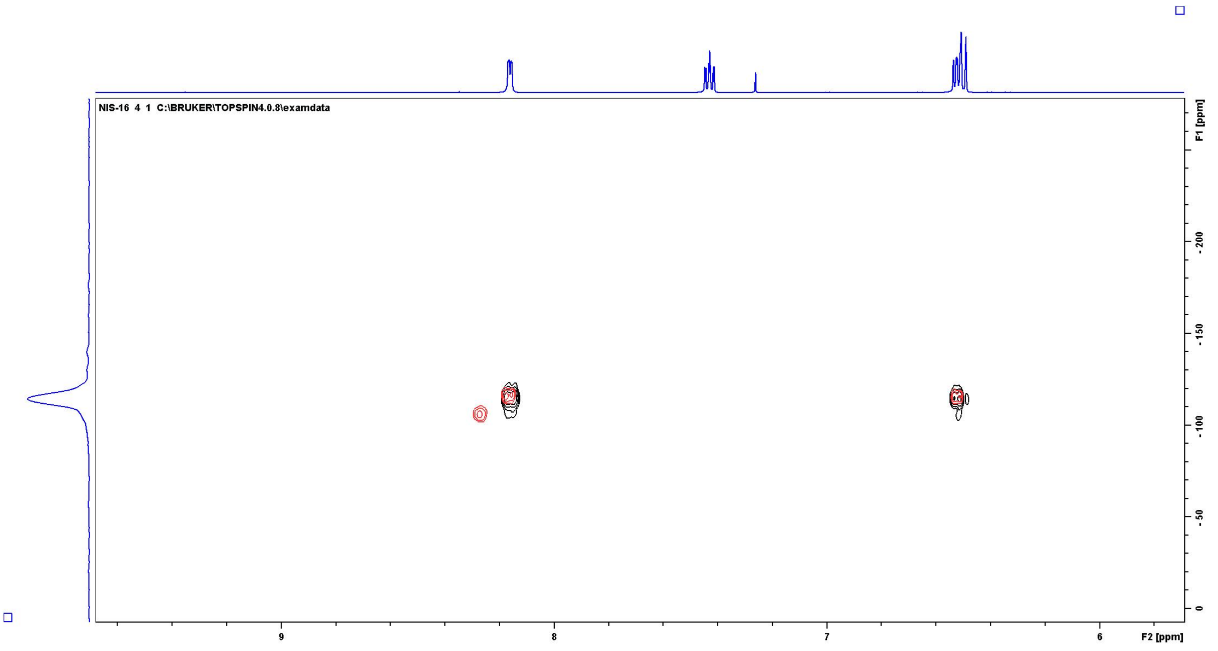

Figure S85. ^{1}H-^{15}N HMBC-NMR Spectra of **16** in black contour and 1:1 complex NIP-**16** in red contour in $CDCl_3$ at 303K.

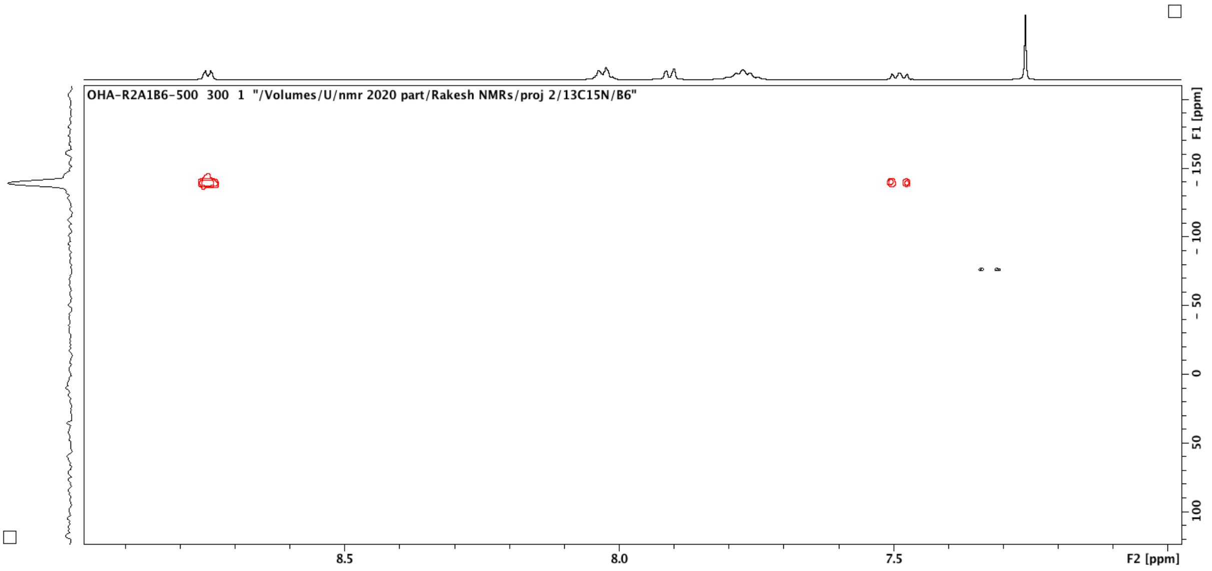

Figure S86. ^{1}H-^{15}N HMBC-NMR Spectra of **1** in black contour and 1:1 complex NISac-**1** in red contour in $CDCl_3$ at 303K.

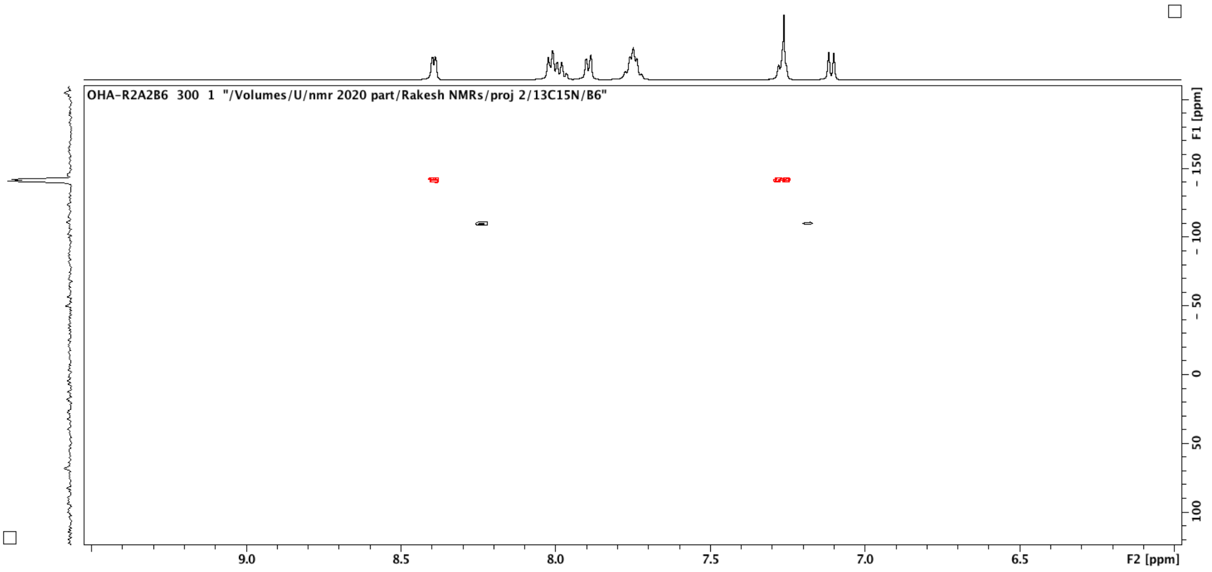

Figure S87. ^{1}H-^{15}N HMBC-NMR Spectra of **2** in black contour and 1:1 complex NISac-**2** in red contour in $CDCl_3$ at 303K.

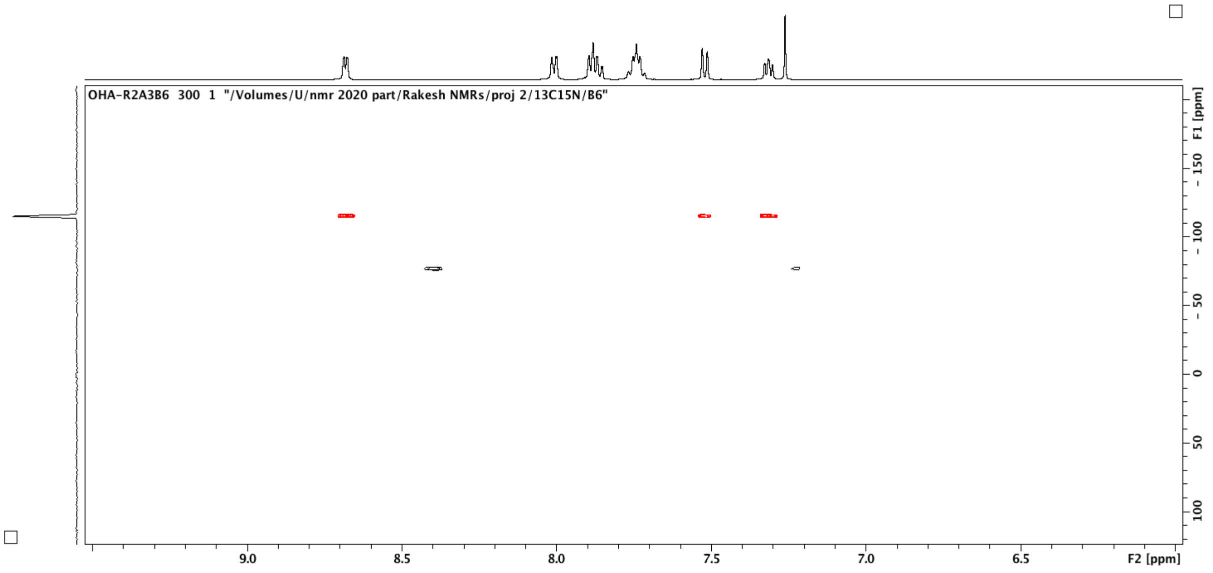

Figure S88. ^{1}H-^{15}N HMBC-NMR Spectra of **3** in black contour and 1:1 complex NISac-**3** in red contour in $CDCl_3$ at 303K.

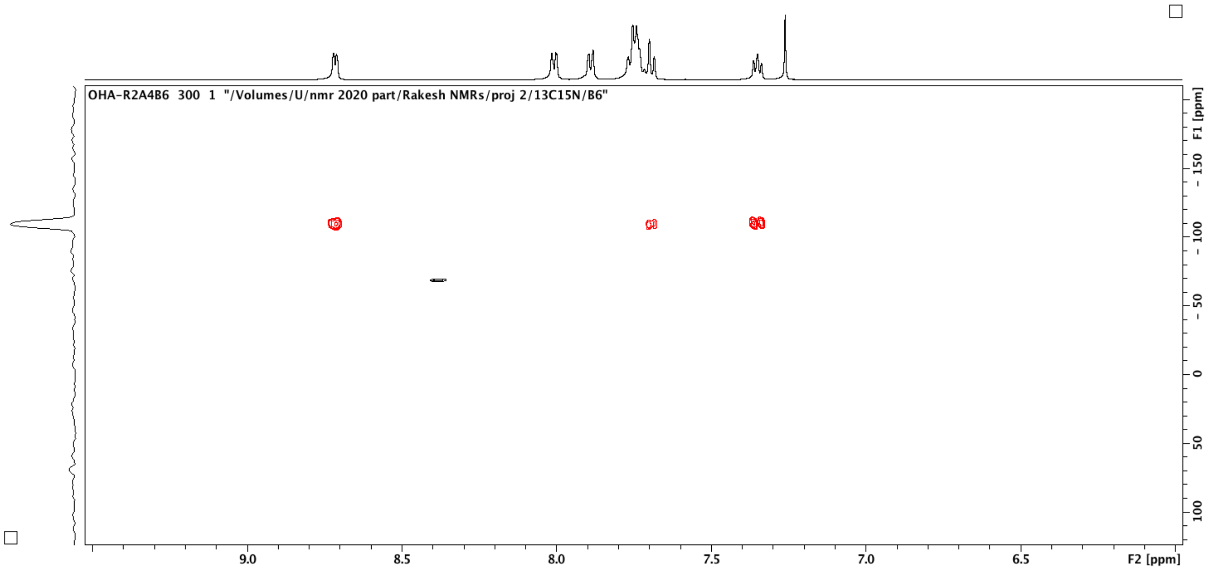

Figure S89. ^{1}H-^{15}N HMBC-NMR Spectra of **4** in black contour and 1:1 complex NISac-**4** in red contour in $CDCl_3$ at 303K.

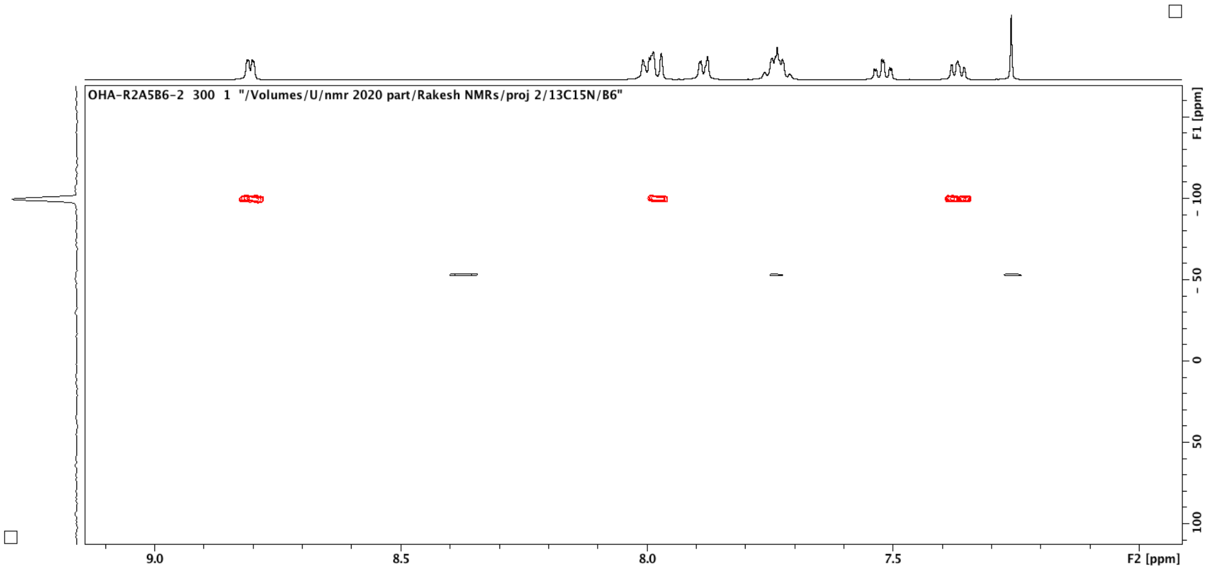

Figure S90. ^{1}H-^{15}N HMBC-NMR Spectra of **5** in black contour and 1:1 complex **NISac-5** in red contour in $CDCl_3$ at 303K.

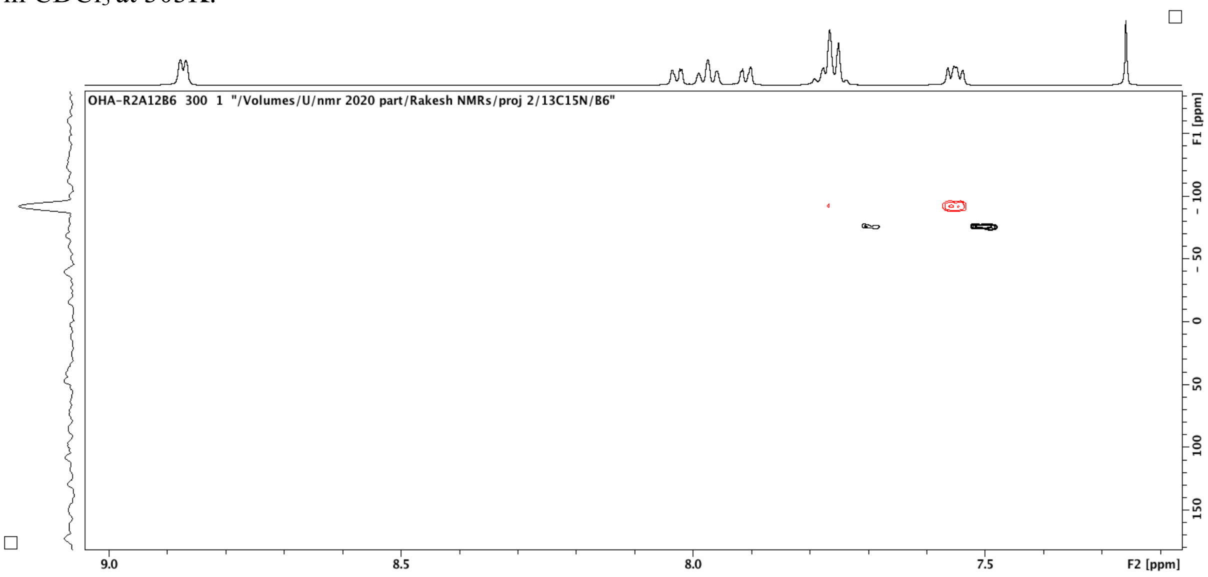

Figure S91. ^{1}H-^{15}N HMBC-NMR Spectra of **6** in black contour and 1:1 complex **NISac-6** in red contour in $CDCl_3$ at 303K.

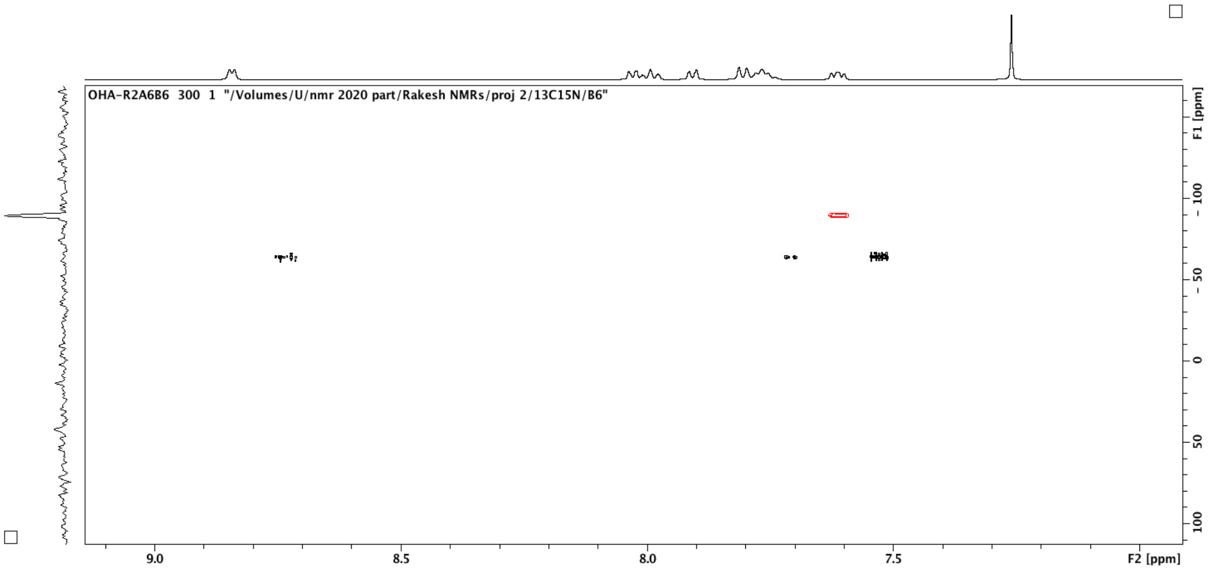

Figure S92. ^{1}H-^{15}N HMBC-NMR Spectra of **7** in black contour and 1:1 complex **NISac-7** in red contour in $CDCl_3$ at 303K.

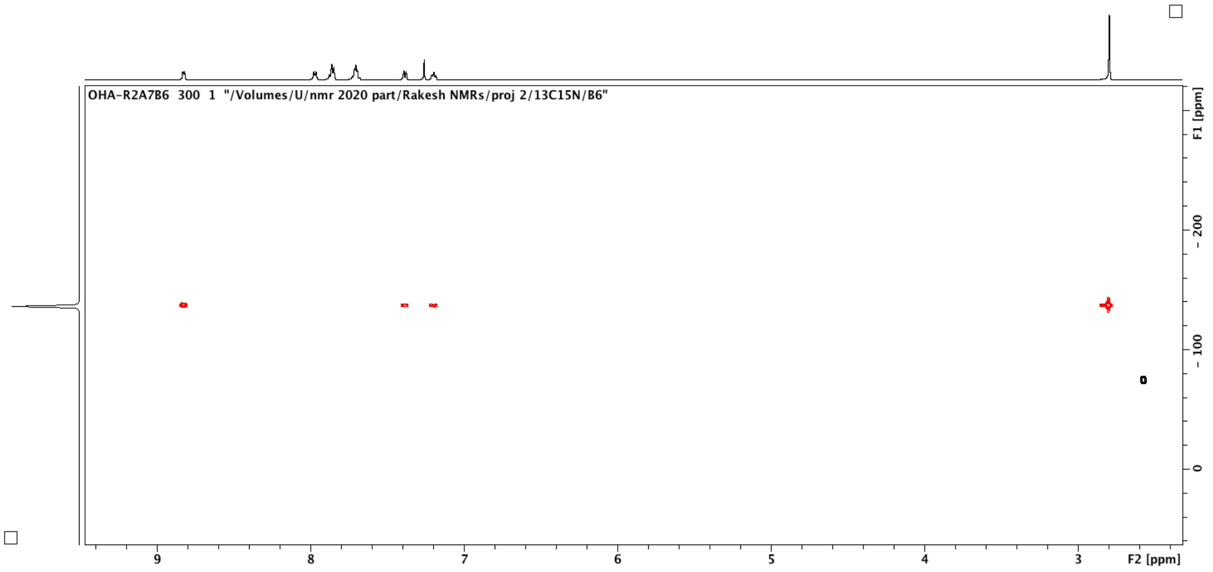

Figure S93. ^{1}H-^{15}N HMBC-NMR Spectra of **8** in black contour and 1:1 complex NISac-**8** in red contour in $CDCl_3$ at 303K.

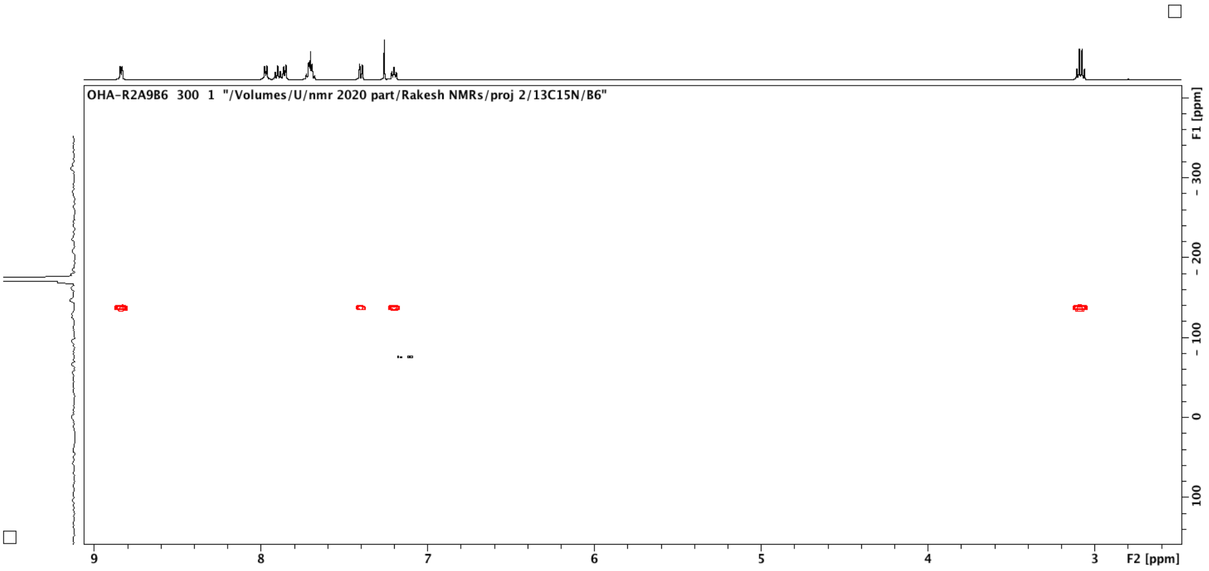

Figure S94. ^{1}H-^{15}N HMBC-NMR Spectra of **9** in black contour and 1:1 complex NISac-**9** in red contour in $CDCl_3$ at 303K.

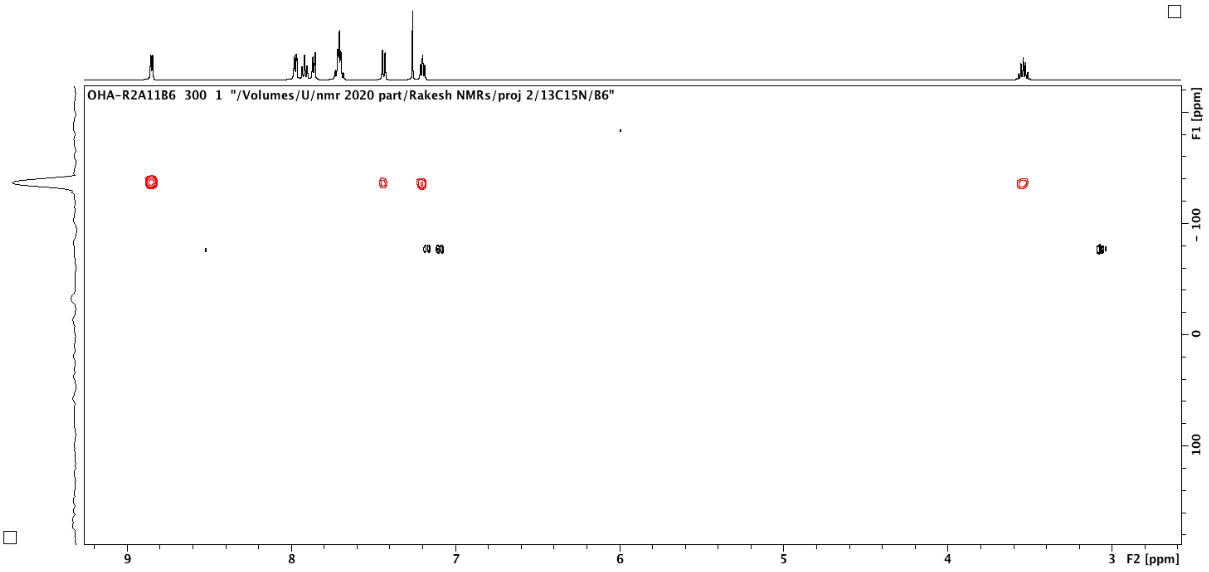

Figure S95. ^{1}H-^{15}N HMBC-NMR Spectra of **10** in black contour and 1:1 complex NISac-**10** in red contour in $CDCl_3$ at 303K.

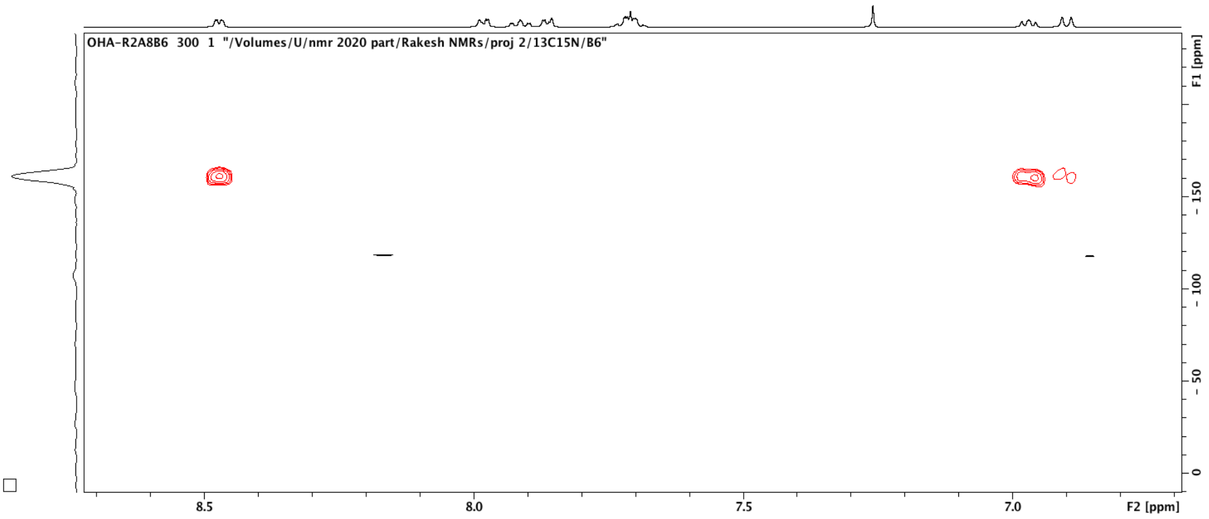

Figure S96. ^{1}H-^{15}N HMBC-NMR Spectra of **11** in black contour and 1:1 complex NISac-**11** in red contour in $CDCl_3$ at 303K.

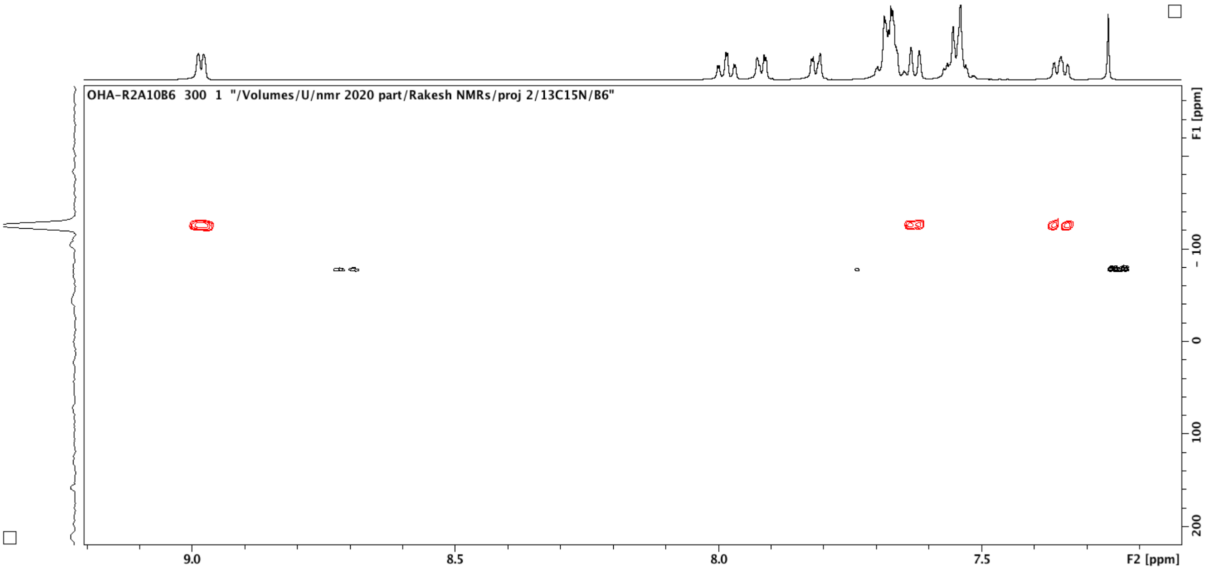

Figure S97. ^{1}H-^{15}N HMBC-NMR Spectra of **12** in black contour 1:1 complex NISac-**12** in red contour in $CDCl_3$ at 303K.

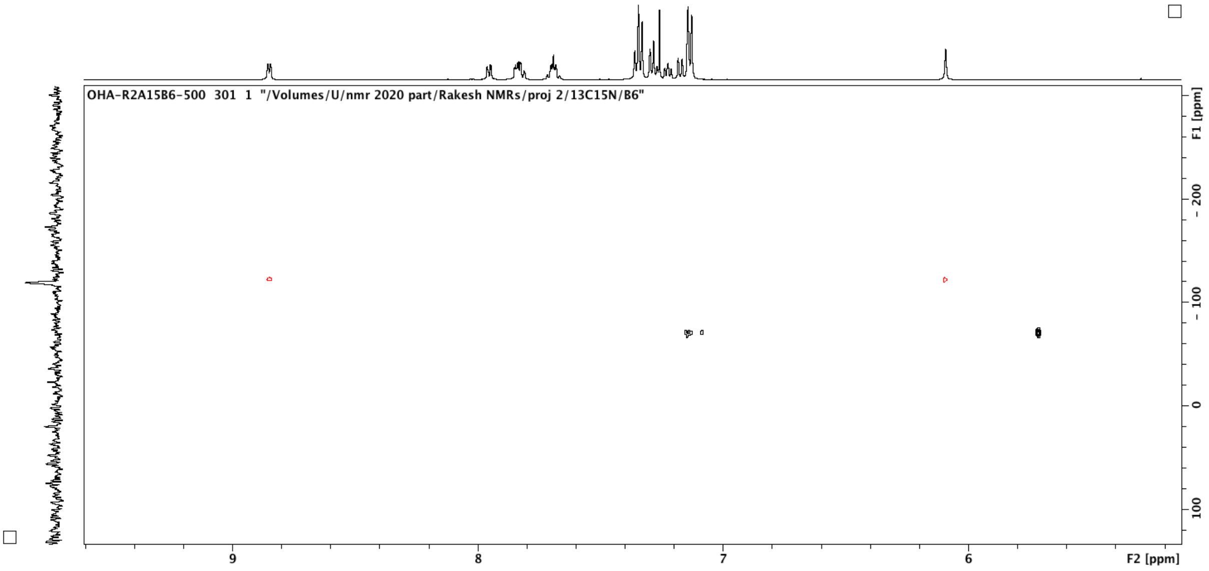

Figure S98. ^{1}H-^{15}N HMBC-NMR Spectra of **13** in black contour and 1:1 complex NISac-**13** in red contour in $CDCl_3$ at 303K.

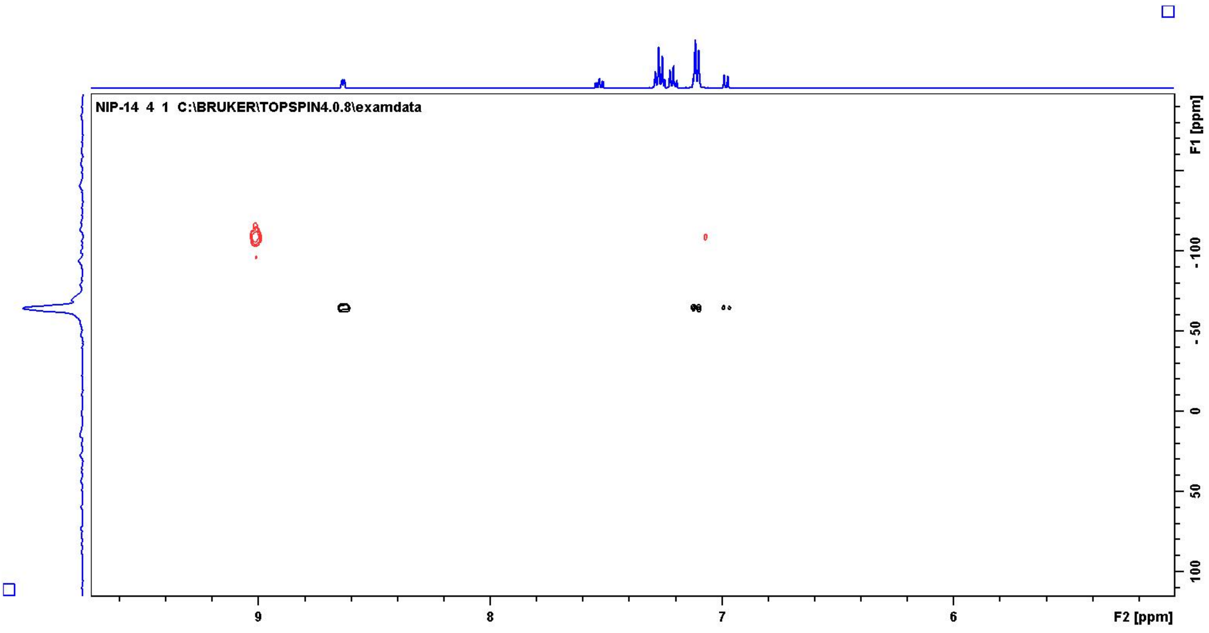

Figure S99. ^{1}H-^{15}N HMBC-NMR Spectra of **14** in black contour and 1:1 complex NISac-**14** in red contour in $CDCl_3$ at 303K.

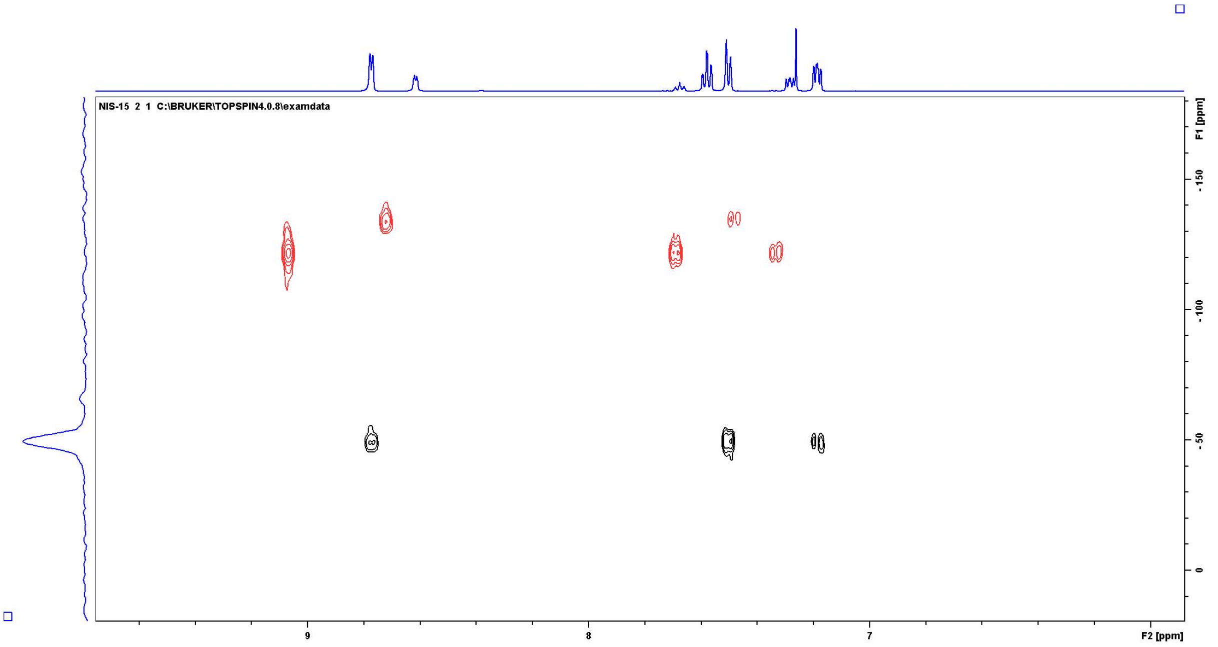

Figure S100. ^{1}H-^{15}N HMBC-NMR Spectra of **15** in black contour and 1:1 complex NISac-**15** in red contour in $CDCl_3$ at 303K.

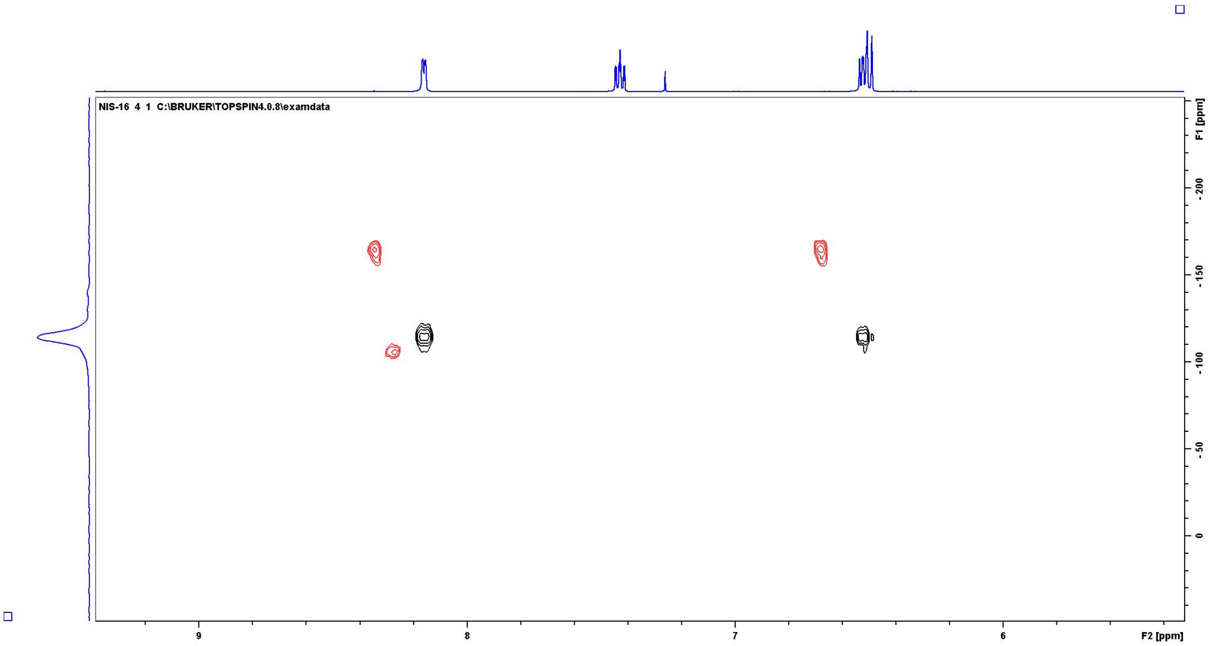

Figure S101. ^{1}H-^{15}N HMBC-NMR Spectra of **16** in black contour and 1:1 complex NISac-**16** in red contour in $CDCl_3$ at 303K.

6. Solution ^{1}H NMR association constants in $CDCl_3$

6.1. ^{1}H Titration experiments for NBS-Z

6.1.1. ^{1}H Titration experiments for NBS-1[a]

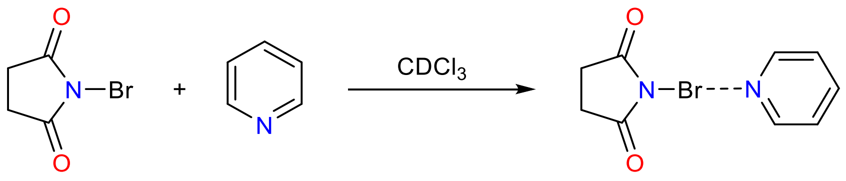

Parameter (bounds)	Optimized	Error	Initial
K (0 → ∞)	4.02 M^{-1}	± 1.0153%	1.00 M^{-1}

[a] See: link for BindFit v0.5 results.

6.1.2. ^{1}H Titration experiments for NBS-2[a]

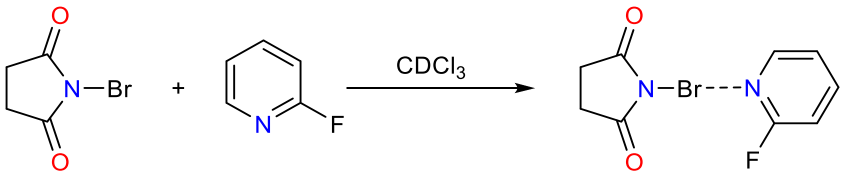

Parameter (bounds)	Optimized	Error	Initial
K (0 → ∞)	0.14 M^{-1}	± 1.7155%	1.00 M^{-1}

[a] See: link for BindFit v0.5 results.

6.1.3. ^{1}H Titration experiments for NBS-3[a]

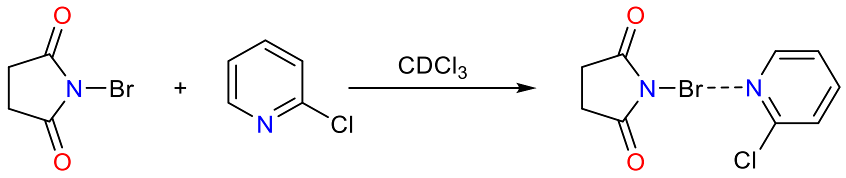

Parameter (bounds)	Optimized	Error	Initial
K (0 → ∞)	0.93 M^{-1}	± 4.2996%	1.00 M^{-1}

[a] See: link for BindFit v0.5 results.

6.1.4. ^{1}H Titration experiments for NBS-4[a]

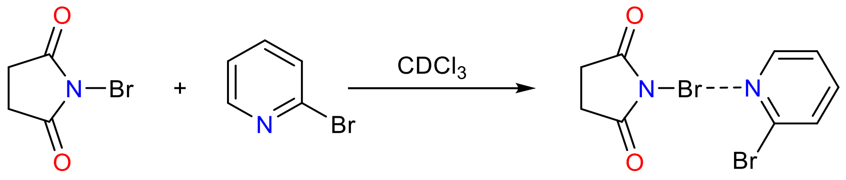

Parameter (bounds)	Optimized	Error	Initial
K (0 → ∞)	9.31 e-5 M^{-1}	± 0.6538%	1.00 M^{-1}

[a] See: link for BindFit v0.5 results.

6.1.5. ^{1}H Titration experiments for NBS-5[a]

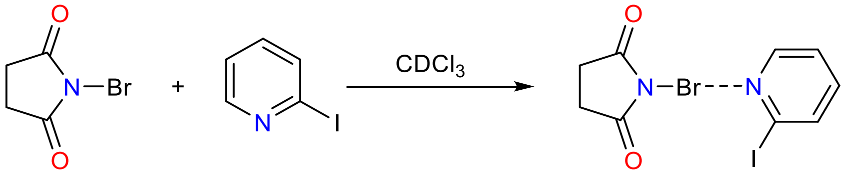

Parameter (bounds)	Optimized	Error	Initial
K (0 → ∞)	1.85 e-5 M^{-1}	± 3.8367e-3%	1.00 M^{-1}

[a] See: link for BindFit v0.5 results.

6.1.6. ^{1}H Titration experiments for NBS-6[a]

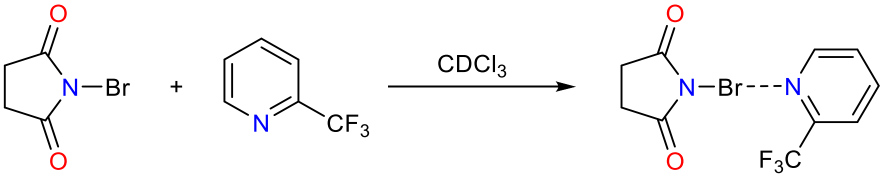

Parameter (bounds)	Optimized	Error	Initial
K (0 → ∞)	2.02 M^{-1}	± 2.8176%	1.00 M^{-1}

[a] See: link for BindFit v0.5 results.

6.1.7. ^{1}H Titration experiments for NBS-7[a]

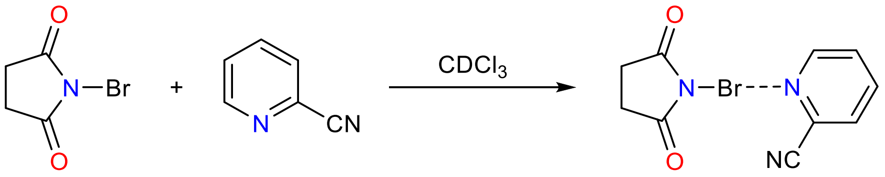

Parameter (bounds)	Optimized	Error	Initial
K (0 → ∞)	0.29 M^{-1}	± 3.0793%	1.00 M^{-1}

[a] See: link for BindFit v0.5 results.

6.1.8. ^{1}H Titration experiments for NBS-8[a]

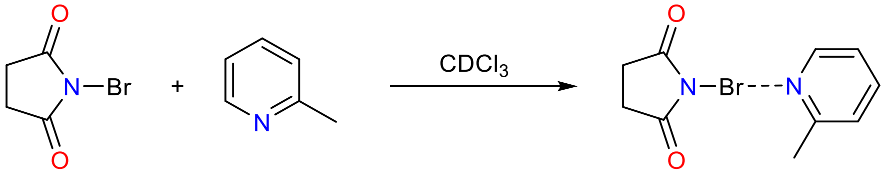

Parameter (bounds)	Optimized	Error	Initial
K (0 → ∞)	5.40 M^{-1}	± 0.3821%	1.00 M^{-1}

[a] See: link for BindFit v0.5 results.

6.1.9. ^{1}H Titration experiments for NBS-9[a]

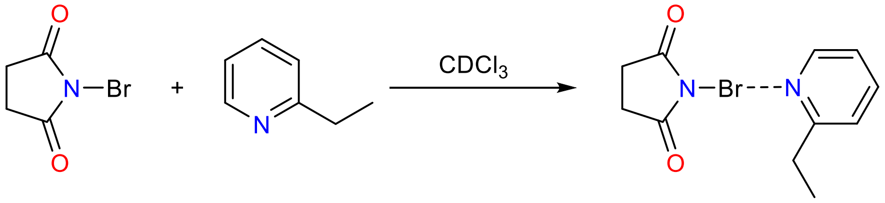

Parameter (bounds)	Optimized	Error	Initial
K (0 → ∞)	4.26 M^{-1}	± 0.7283%	1.00 M^{-1}

[a] See: link for BindFit v0.5 results.

6.1.10. ^{1}H Titration experiments for NBS-10[a]

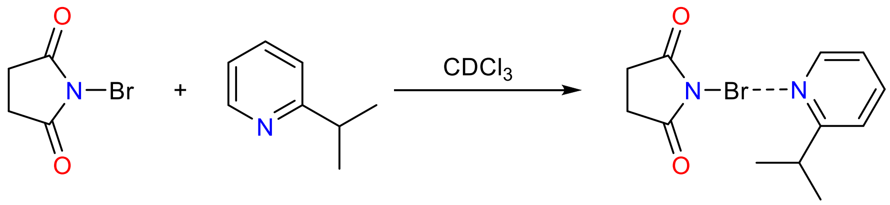

Parameter (bounds)	Optimized	Error	Initial
K (0 → ∞)	2.16 M^{-1}	± 0.3106%	1.00 M^{-1}

[a] See: link for BindFit v0.5 results.

6.1.11. ^{1}H Titration experiments for NBS-11[a]

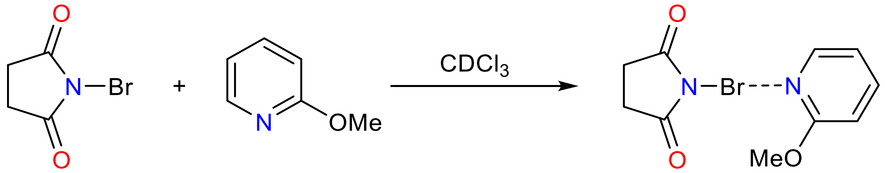

Parameter (bounds)	Optimized	Error	Initial
K (0 → ∞)	0.54 M^{-1}	± 1.9841%	1.00 M^{-1}

[a] See: link for BindFit v0.5 results.

6.1.12. ^{1}H Titration experiments for NBS-12[a]

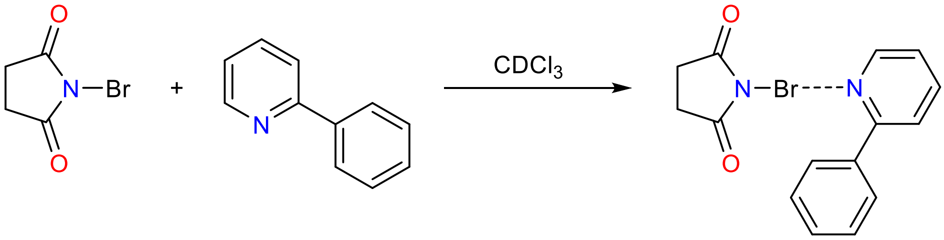

Parameter (bounds)	Optimized	Error	Initial
K (0 → ∞)	3.60e-2 M^{-1}	± 0.6035%	1.00 M^{-1}

[a] See: link for BindFit v0.5 results.

6.1.13. ^{1}H Titration experiments for NBS-13[a]

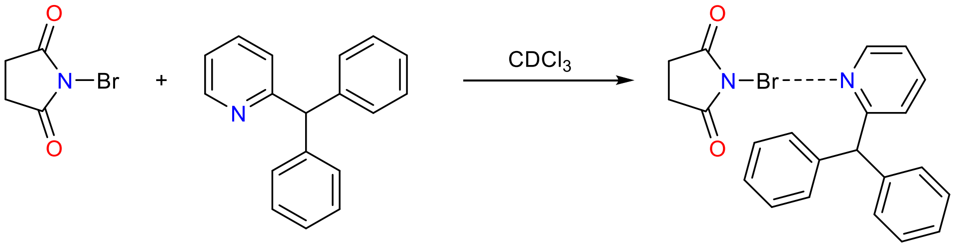

Parameter (bounds)	Optimized	Error	Initial
K (0 → ∞)	1.56 M^{-1}	± 0.5826%	1.00 M^{-1}

[a] See: link for BindFit v0.5 results.

6.1.14. ^{1}H Titration experiments for NBS-14[a]

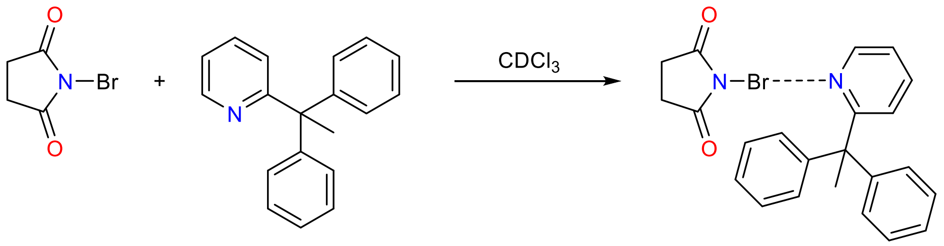

Parameter (bounds)	Optimized	Error	Initial
K (0 → ∞)	1.56 M^{-1}	± 0.5826%	1.00 M^{-1}

[a] See: link for BindFit v0.5 results.

6.1.15. ^{1}H Titration experiments for NBS-15[a]

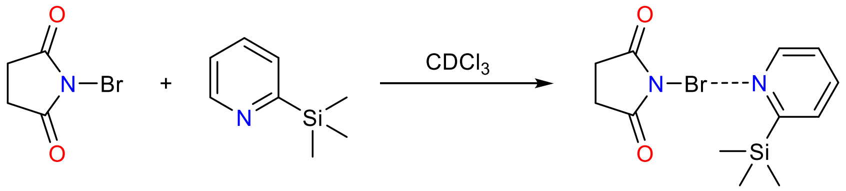

Parameter (bounds)	Optimized	Error	Initial
K (0 → ∞)	2.51 M^{-1}	± 0.3821%	1.00 M^{-1}

[a] See: link for BindFit v0.5 results.

6.2. ^{1}H Titration experiments for NBP-Z

6.2.1. ^{1}H Titration experiments for NBP-1[a]

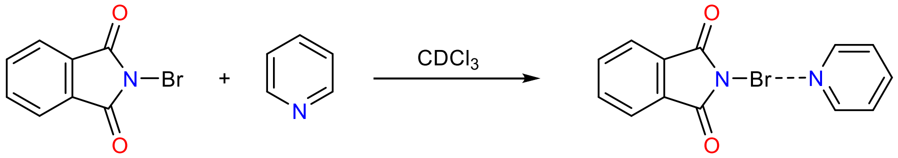

Parameter (bounds)	Optimized	Error	Initial
K (0 → ∞)	2.34 M^{-1}	± 0.3782%	1.00 M^{-1}

[a] See: link for BindFit v0.5 results.

6.2.2. ^{1}H Titration experiments for NBP-2[a]

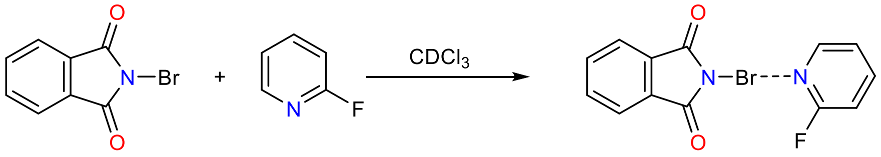

Parameter (bounds)	Optimized	Error	Initial
K (0 → ∞)	1.59e-4 M^{-1}	± 0.7428%	1.00 M^{-1}

[a] See: link for BindFit v0.5 results.

6.2.3. ^{1}H Titration experiments for NBP-3[a]

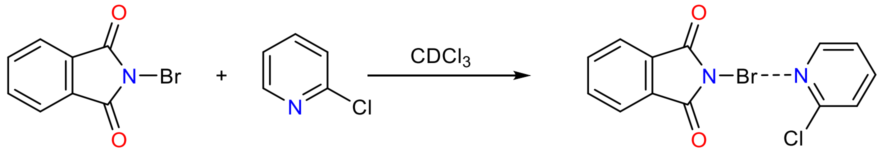

Parameter (bounds)	Optimized	Error	Initial
K (0 → ∞)	1.22e-4 M^{-1}	± 0.2997%	1.00 M^{-1}

[a] See: link for BindFit v0.5 results.

6.2.4. ^{1}H Titration experiments for NBP-4[a]

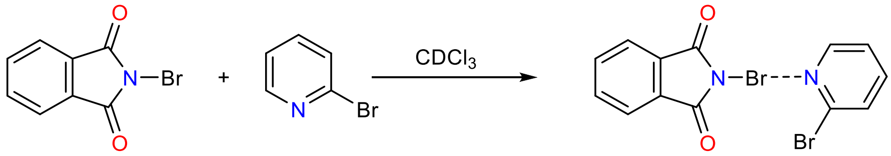

Parameter (bounds)	Optimized	Error	Initial
K (0 → ∞)	0.25 M^{-1}	± 0.6637%	1.00 M^{-1}

[a] See: link for BindFit v0.5 results.

6.2.5. ^{1}H Titration experiments for NBP-5[a]

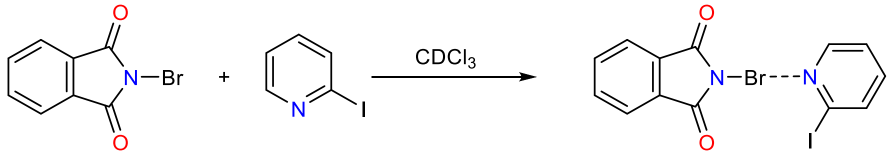

Parameter (bounds)	Optimized	Error	Initial
K (0 → ∞)	1.10e-4 M^{-1}	± 0.0873%	1.00e-4 M^{-1}

[a] See: link for BindFit v0.5 results.

6.2.6. ^{1}H Titration experiments for NBP-6[a]

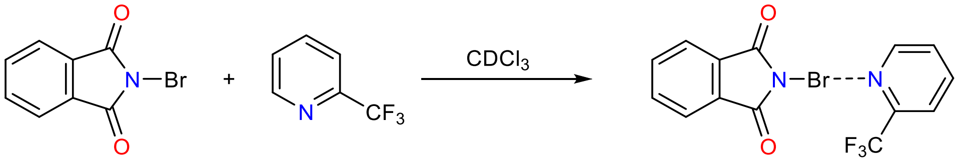

Parameter (bounds)	Optimized	Error	Initial
K (0 → ∞)	1.02e-4 M^{-1}	± 0.1388%	1.00e-4 M^{-1}

[a] See: link for BindFit v0.5 results.

6.2.7. ^{1}H Titration experiments for NBP-7[a]

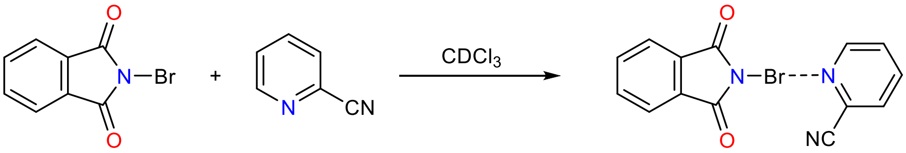

Parameter (bounds)	Optimized	Error	Initial
K (0 → ∞)	1.71e-4 M^{-1}	± 1.2154%	0.10 M^{-1}

[a] See: link for BindFit v0.5 results.

6.2.8. [1]H Titration experiments for NBP-8[a]

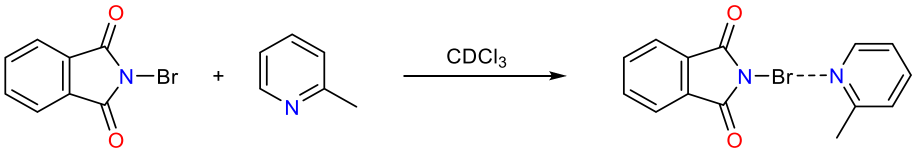

Parameter (bounds)	Optimized	Error	Initial
K (0 → ∞)	3.98 M^{-1}	± 0.1659%	1.00 M^{-1}

[a] See: link for BindFit v0.5 results.

6.2.9. [1]H Titration experiments for NBP-9[a]

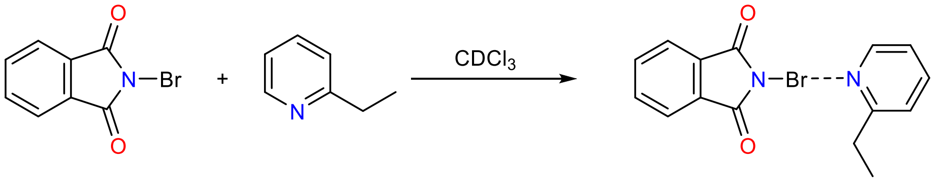

Parameter (bounds)	Optimized	Error	Initial
K (0 → ∞)	1.76 M^{-1}	± 0.3071%	1.00 M^{-1}

[a] See: link for BindFit v0.5 results.

6.2.10. [1]H Titration experiments for NBP-10[a]

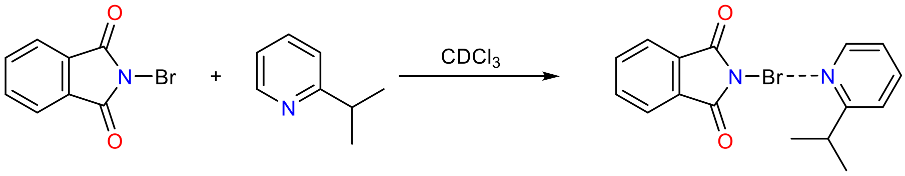

Parameter (bounds)	Optimized	Error	Initial
K (0 → ∞)	1.44 M^{-1}	± 0.1446%	1.00 M^{-1}

[a] See: link for BindFit v0.5 results.

6.2.11. [1]H Titration experiments for NBP-11[a]

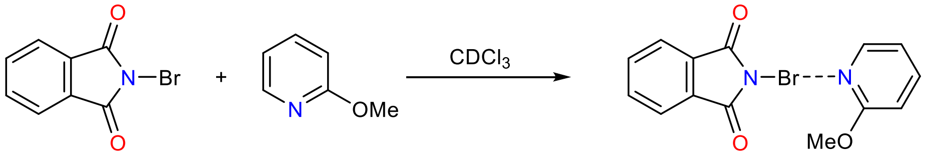

Parameter (bounds)	Optimized	Error	Initial
K (0 → ∞)	9.80e-5 M^{-1}	± 0.0838%	0.10 M^{-1}

[a] See: link for BindFit v0.5 results.

6.2.12. [1]H Titration experiments for NBP-12[a]

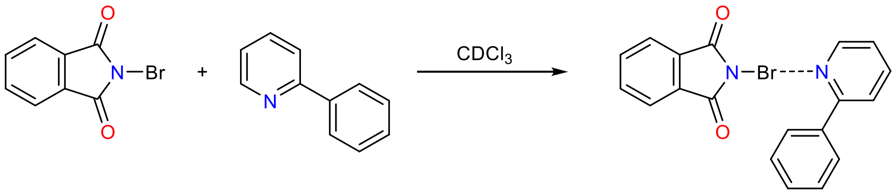

Parameter (bounds)	Optimized	Error	Initial
K (0 → ∞)	7.63e-5 M^{-1}	± 0.1360%	1.00 M^{-1}

[a] See: link for BindFit v0.5 results.

6.2.13. ¹H Titration experiments for NBP-13[a]

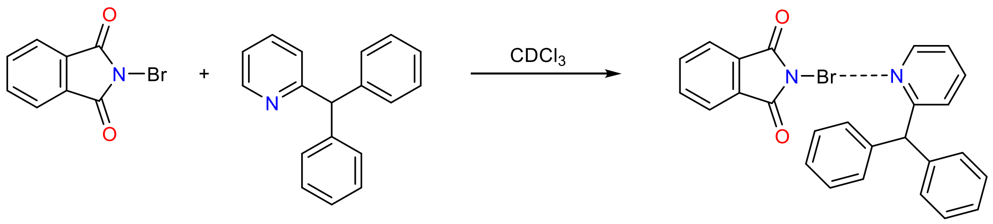

Parameter (bounds)	Optimized	Error	Initial
K (0 → ∞)	1.73 M^{-1}	± 0.5241%	1.00 M^{-1}

a See: link for BindFit v0.5 results.

6.2.14. ¹H Titration experiments for NBP-14[a]

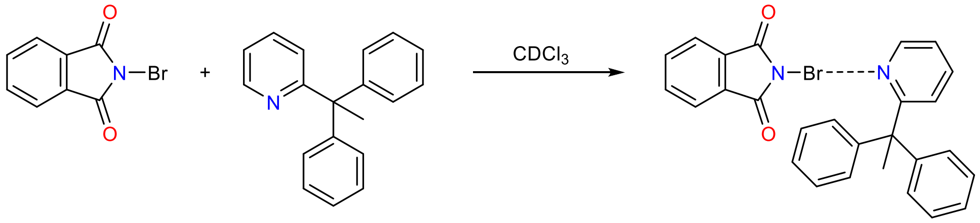

Parameter (bounds)	Optimized	Error	Initial
K (0 → ∞)	1.73 M^{-1}	± 0.5241%	1.00 M^{-1}

a See: link for BindFit v0.5 results.

6.2.15. ¹H Titration experiments for NBP-15[a]

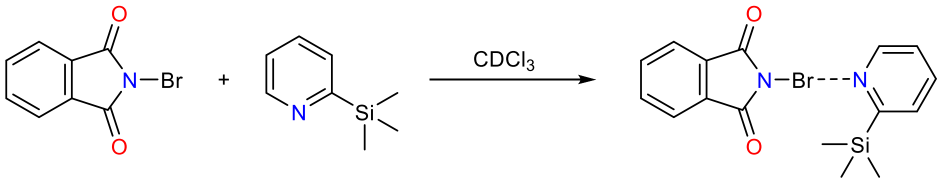

Parameter (bounds)	Optimized	Error	Initial
K (0 → ∞)	1.86 M^{-1}	± 0.2434%	1.00 M^{-1}

a See: link for BindFit v0.5 results.

6.3. ¹H Titration experiments for NBSac-Z (1-15)

6.3.1. ¹H Titration experiments for NBSac-1[a]

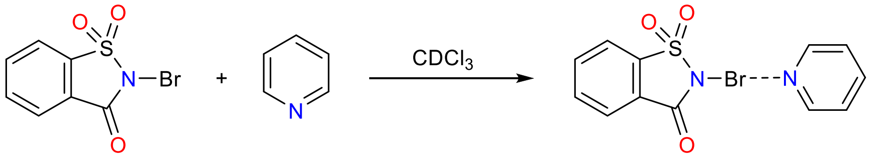

Parameter (bounds)	Optimized	Error	Initial
K (0 → ∞)	423.78 M^{-1}	± 4.3343%	100.00 M^{-1}

a See: link for BindFit v0.5 results.

6.3.2. [1]H Titration experiments for NBSac-2[a]

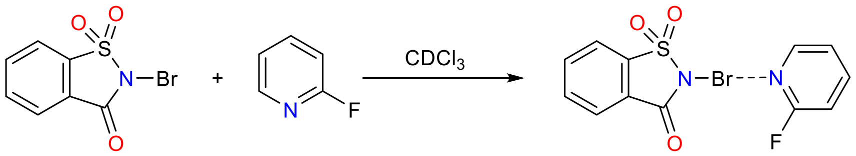

Parameter (bounds)	Optimized	Error	Initial
K (0 → ∞)	1.17 M^{-1}	± 0.8769%	1.00 M^{-1}

[a] See: link for BindFit v0.5 results.

6.3.3. [1]H Titration experiments for NBSac-3[a]

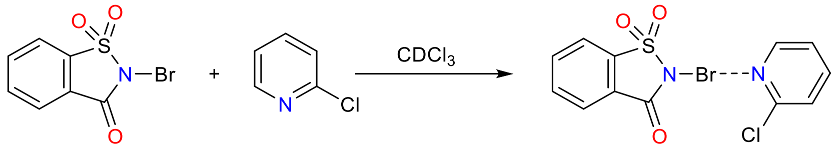

Parameter (bounds)	Optimized	Error	Initial
K (0 → ∞)	5.31 M^{-1}	± 0.3510%	1.00 M^{-1}

[a] See: link for BindFit v0.5 results.

6.3.4. [1]H Titration experiments for NBSac-4[a]

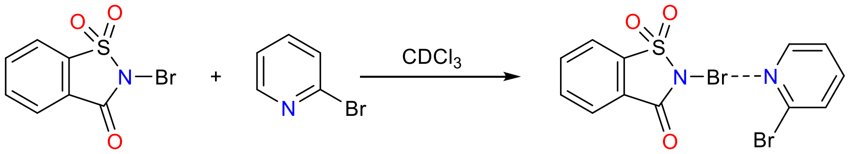

Parameter (bounds)	Optimized	Error	Initial
K (0 → ∞)	5.03 M^{-1}	± 0.3709%	1.00 M^{-1}

[a] See: link for BindFit v0.5 results.

6.3.5. [1]H Titration experiments for NBSac-5[a]

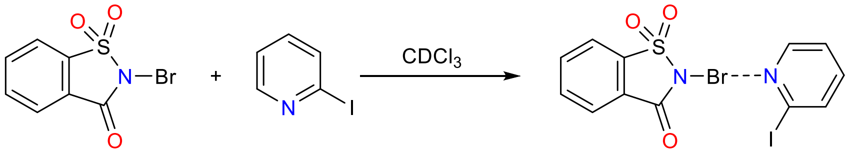

Parameter (bounds)	Optimized	Error	Initial
K (0 → ∞)	14.29 M^{-1}	± 0.5387%	10.00 M^{-1}

[a] See: link for BindFit v0.5 results.

6.3.6. [1]H Titration experiments for NBSac-6[a]

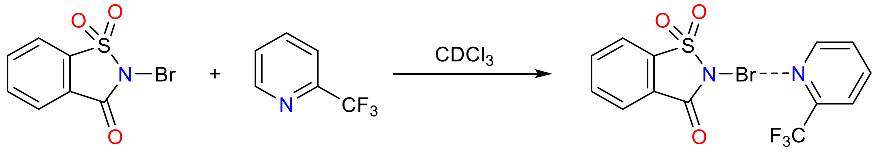

Parameter (bounds)	Optimized	Error	Initial
K (0 → ∞)	2.72 M^{-1}	± 1.6387%	1.00 M^{-1}

[a] See: link for BindFit v0.5 results.

6.3.7. ^{1}H Titration experiments for NBSac-7[a]

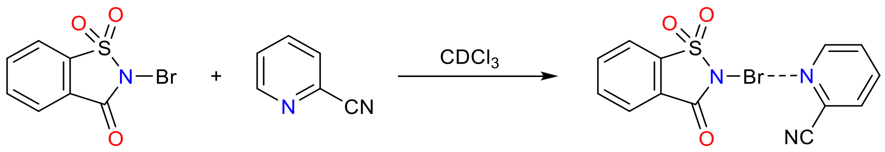

Parameter (bounds)	Optimized	Error	Initial
K (0 → ∞)	0.40 M^{-1}	± 0.3756%	1.00 M^{-1}

[a] See: link for BindFit v0.5 results.

6.3.8. ^{1}H Titration experiments for NBSac-8[a]

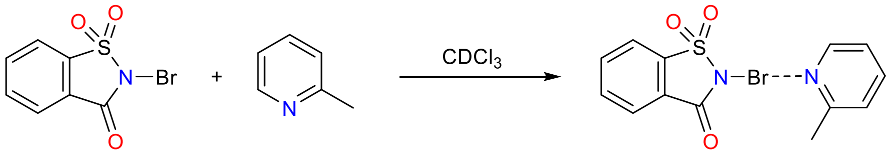

Parameter (bounds)	Optimized	Error	Initial
K (0 → ∞)	330.50 M^{-1}	± 2.5802%	100.00 M^{-1}

[a] See: link for BindFit v0.5 results.

6.3.9. ^{1}H Titration experiments for NBSac-9[a]

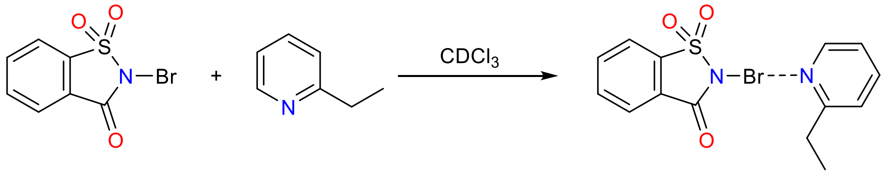

Parameter (bounds)	Optimized	Error	Initial
K (0 → ∞)	455.41 M^{-1}	± 1.9234%	100.00 M^{-1}

[a] See: link for BindFit v0.5 results.

6.3.10. ^{1}H Titration experiments for NBSac-10[a]

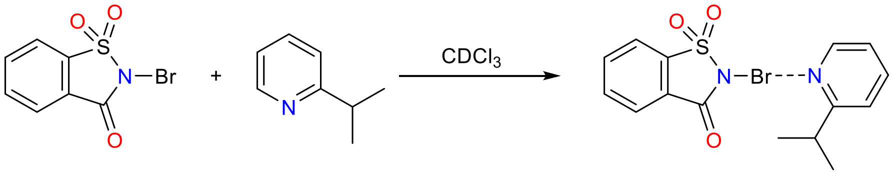

Parameter (bounds)	Optimized	Error	Initial
K (0 → ∞)	209.79 M^{-1}	± 2.1042%	1.00 M^{-1}

[a] See: link for BindFit v0.5 results.

6.3.11. ¹H Titration experiments for NBSac-11[a]

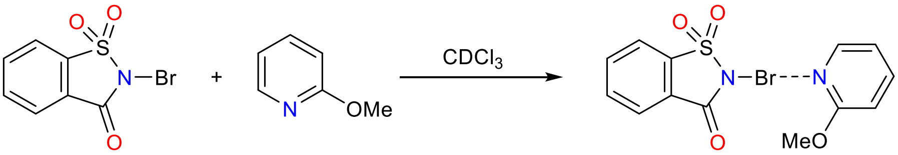

Parameter (bounds)	Optimized	Error	Initial
K (0 → ∞)	3.67 M^{-1}	± 0.4597%	1.00 M^{-1}

[a] See: link for BindFit v0.5 results.

6.3.12. ¹H Titration experiments for NBSac-12[a]

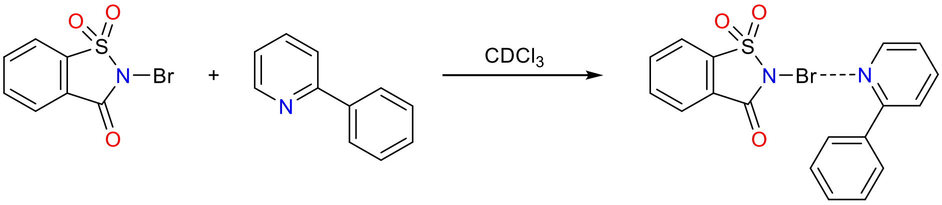

Parameter (bounds)	Optimized	Error	Initial
K (0 → ∞)	21.29 M^{-1}	± 0.9460%	100.00 M^{-1}

[a] See: link for BindFit v0.5 results.

6.3.13. ¹H Titration experiments for NBSac-13[a]

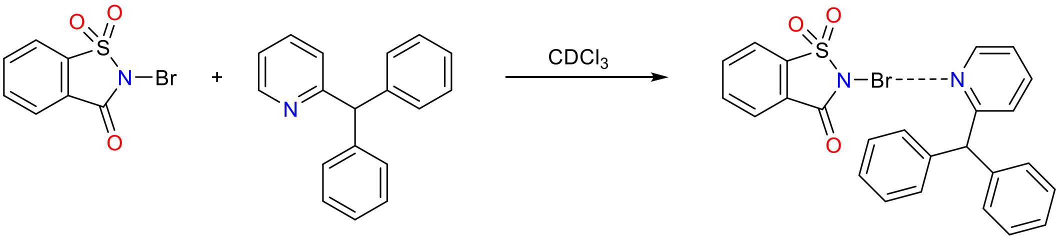

Parameter (bounds)	Optimized	Error	Initial
K (0 → ∞)	147.07 M^{-1}	± 0.9839%	100.00 M^{-1}

[a] See: link for BindFit v0.5 results.

6.3.14. ¹H Titration experiments for NBSac-14[a]

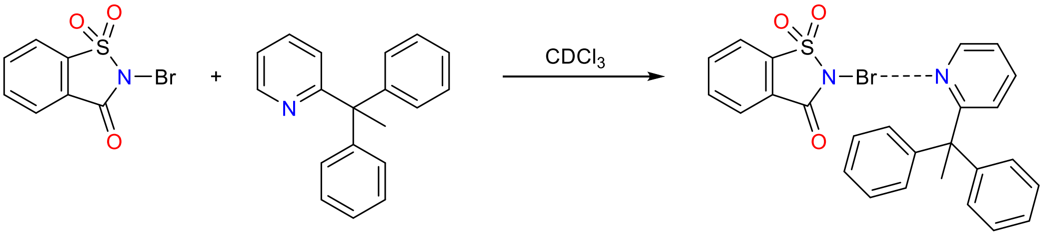

Parameter (bounds)	Optimized	Error	Initial
K (0 → ∞)	147.07 M^{-1}	± 0.9839%	100.00 M^{-1}

[a] See: link for BindFit v0.5 results.

6.3.15. [1]H Titration experiments for NBSac-15[a]

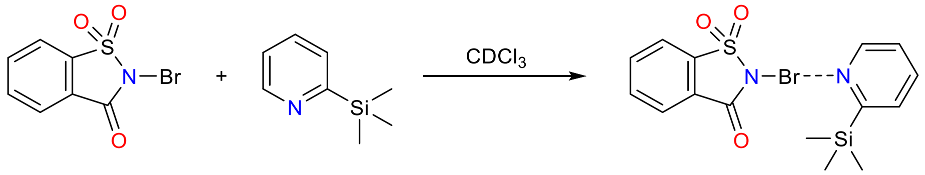

Parameter (bounds)	Optimized	Error	Initial
K (0 → ∞)	394.09 M^{-1}	± 2.9606%	100.00 M^{-1}

[a] See: link for BindFit v0.5 results.

6.4. [1]H Titration experiments for NIS-Z (1-15)

6.4.1. [1]H Titration experiments for NIS-1[a]

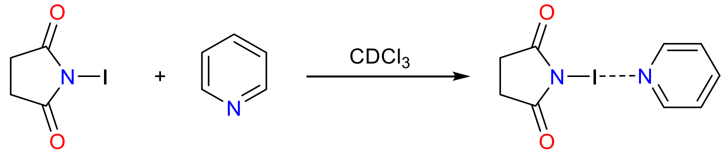

Parameter (bounds)	Optimized	Error	Initial
K (0 → ∞)	1470.69 M^{-1}	± 48.7749%	1000.00 M^{-1}

[a] See: link for BindFit v0.5 results.

6.4.2. [1]H Titration experiments for NIS-2[a]

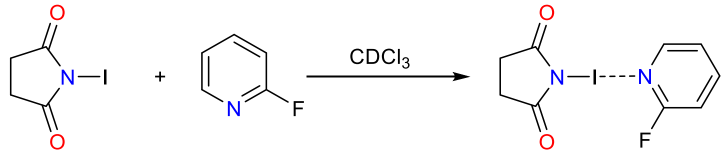

Parameter (bounds)	Optimized	Error	Initial
K (0 → ∞)	0.14 M^{-1}	± 1.7155%	1.00 M^{-1}

[a] See: link for BindFit v0.5 results.

6.4.3. [1]H Titration experiments for NIS-3[a]

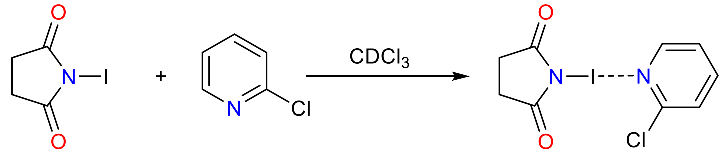

Parameter (bounds)	Optimized	Error	Initial
K (0 → ∞)	0.93 M^{-1}	± 4.2996%	1.00 M^{-1}

[a] See: link for BindFit v0.5 results.

6.4.4. ^{1}H Titration experiments for NIS-4[a]

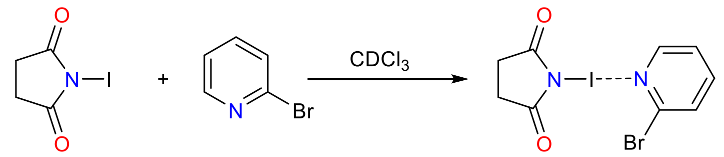

Parameter (bounds)	Optimized	Error	Initial
K (0 → ∞)	9.31 e-5 M^{-1}	± 0.6538%	1.00 M^{-1}

[a] See: link for BindFit v0.5 results.

6.4.5. ^{1}H Titration experiments for NIS-5[a]

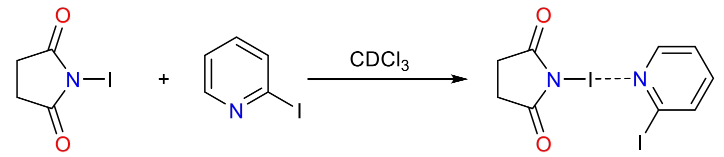

Parameter (bounds)	Optimized	Error	Initial
K (0 → ∞)	1.85 e-5 M^{-1}	± 3.8367e-3%	1.00 M^{-1}

[a] See: link for BindFit v0.5 results.

6.4.6. ^{1}H Titration experiments for NIS-6[a]

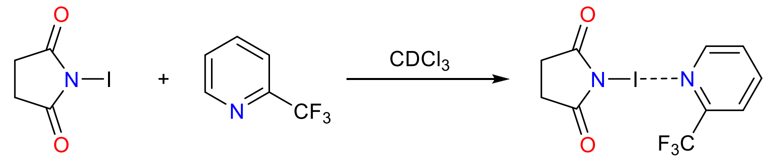

Parameter (bounds)	Optimized	Error	Initial
K (0 → ∞)	2.02 M^{-1}	± 2.8176%	1.00 M^{-1}

[a] See: link for BindFit v0.5 results.

6.4.7. ^{1}H Titration experiments for NIS-7[a]

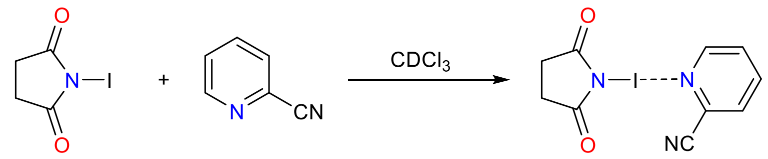

Parameter (bounds)	Optimized	Error	Initial
K (0 → ∞)	11.92 M^{-1}	± 0.6619%	1.00 M^{-1}

[a] See: link for BindFit v0.5 results.

6.4.8. ^{1}H Titration experiments for NIS-8[a]

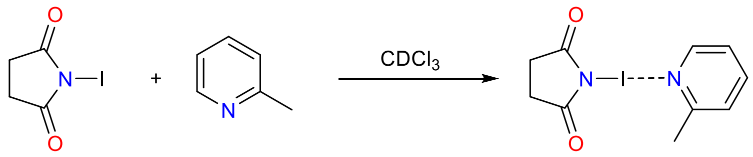

Parameter (bounds)	Optimized	Error	Initial
K (0 → ∞)	3493.51 M^{-1}	± 44.4667%	1000.00 M^{-1}

[a] See: link for BindFit v0.5 results.

6.4.9. ^{1}H Titration experiments for NIS-9[a]

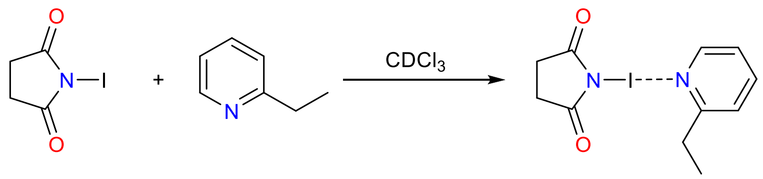

Parameter (bounds)	Optimized	Error	Initial
K (0 → ∞)	3397.65 M^{-1}	± 47.4420%	1000.00 M^{-1}

[a] See: link for BindFit v0.5 results.

6.4.10. ^{1}H Titration experiments for NIS-10[a]

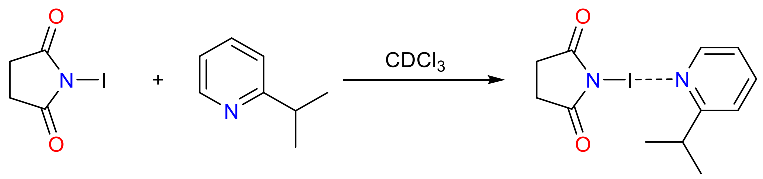

Parameter (bounds)	Optimized	Error	Initial
K (0 → ∞)	588.41 M^{-1}	± 27.2024%	100.00 M^{-1}

[a] See: link for BindFit v0.5 results.

6.4.11. ^{1}H Titration experiments for NIS-11[a]

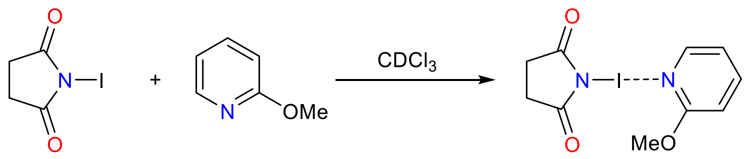

Parameter (bounds)	Optimized	Error	Initial
K (0 → ∞)	0.54 M^{-1}	± 1.9841%	1.00 M^{-1}

[a] See: link for BindFit v0.5 results.

6.4.12. ^{1}H Titration experiments for NIS-12[a]

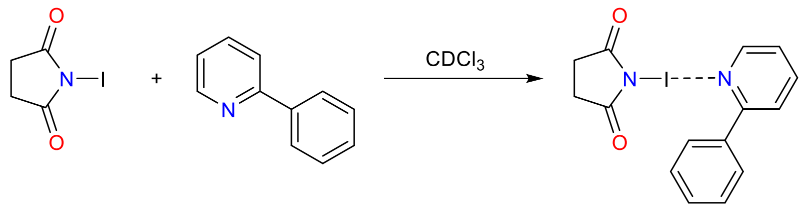

Parameter (bounds)	Optimized	Error	Initial
K (0 → ∞)	3.60e-2 M^{-1}	± 0.6035%	1.00 M^{-1}

[a] See: link for BindFit v0.5 results.

6.4.13. ¹H Titration experiments for NIS-13[a]

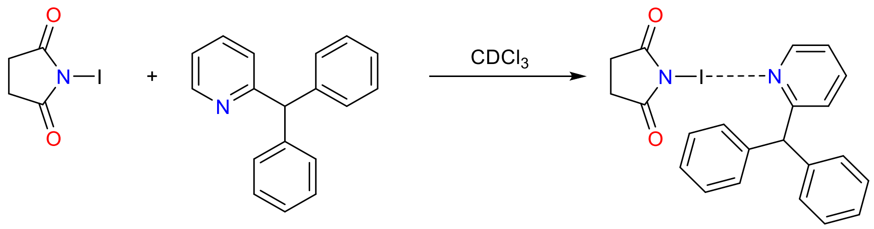

Parameter (bounds)	Optimized	Error	Initial
K (0 → ∞)	489.16 M^{-1}	± 12.0930%	100.00 M^{-1}

[a] See: link for BindFit v0.5 results.

6.4.14. ¹H Titration experiments for NIS-14[a]

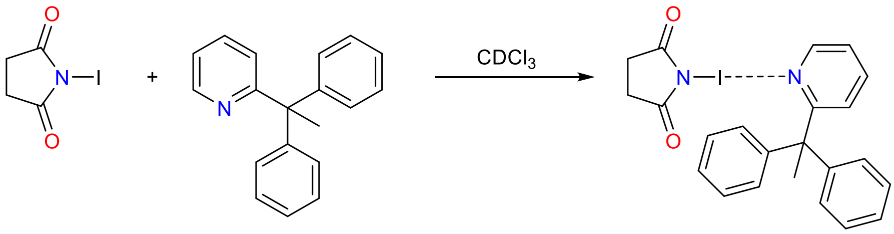

Parameter (bounds)	Optimized	Error	Initial
K (0 → ∞)	489.16 M^{-1}	± 12.0930%	100.00 M^{-1}

[a] See: link for BindFit v0.5 results.

6.4.15. ¹H Titration experiments for NIS-15[a]

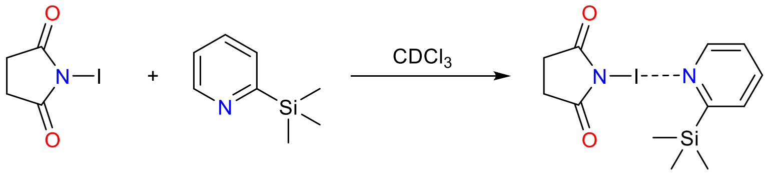

Parameter (bounds)	Optimized	Error	Initial
K (0 → ∞)	521.76 M^{-1}	± 16.7211%	100.00 M^{-1}

[a] See: link for BindFit v0.5 results.

6.5. ¹H Titration experiments for NIP-Z

6.5.1. ¹H Titration experiments for NIP-1[a]

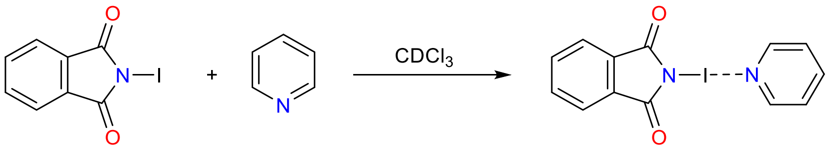

Parameter (bounds)	Optimized	Error	Initial
K (0 → ∞)	1123.54 M^{-1}	± 13.8345%	1000.00 M^{-1}

[a] See: link for BindFit v0.5 results.

6.5.2. ^{1}H Titration experiments for NIP-2[a]

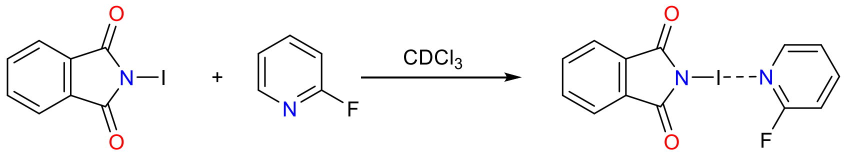

Parameter (bounds)	Optimized	Error	Initial
K (0 → ∞)	11.28 M^{-1}	± 0.1680%	1.00 M^{-1}

[a] See: link for BindFit v0.5 results.

6.5.3. ^{1}H Titration experiments for NIP-3[a]

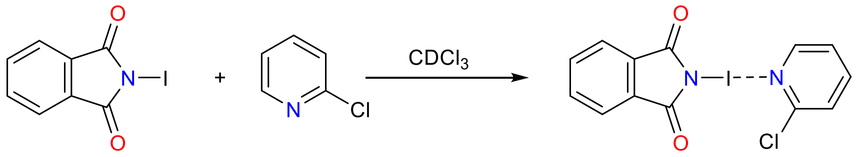

Parameter (bounds)	Optimized	Error	Initial
K (0 → ∞)	23.09 M^{-1}	± 0.2044%	10.00 M^{-1}

[a] See: link for BindFit v0.5 results.

6.5.4. ^{1}H Titration experiments for NIP-4[a]

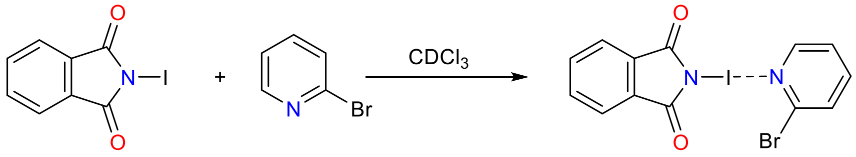

Parameter (bounds)	Optimized	Error	Initial
K (0 → ∞)	17.86 M^{-1}	± 0.2676%	10.00 M^{-1}

[a] See: link for BindFit v0.5 results.

6.5.5. ^{1}H Titration experiments for NIP-5[a]

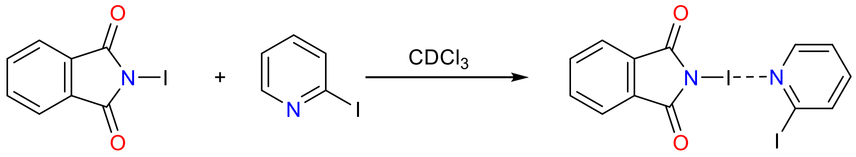

Parameter (bounds)	Optimized	Error	Initial
K (0 → ∞)	56.17 M^{-1}	± 0.2821%	10.00 M^{-1}

[a] See: link for BindFit v0.5 results.

6.5.6. ^{1}H Titration experiments for NIP-6[a]

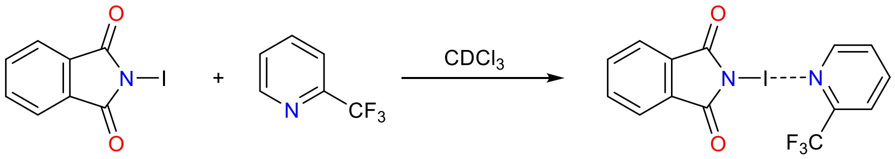

Parameter (bounds)	Optimized	Error	Initial
K (0 → ∞)	6.45 M^{-1}	± 0.7056%	10.00 M^{-1}

[a] See: link for BindFit v0.5 results.

6.5.7. ^{1}H Titration experiments for NIP-7[a]

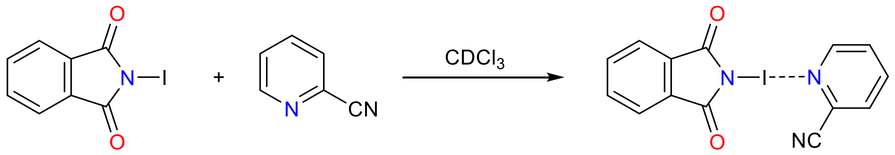

Parameter (bounds)	Optimized	Error	Initial
K (0 → ∞)	14.90 M^{-1}	± 0.6524%	10.00 M^{-1}

[a] See: link for BindFit v0.5 results.

6.5.8. ^{1}H Titration experiments for NIP-8[a]

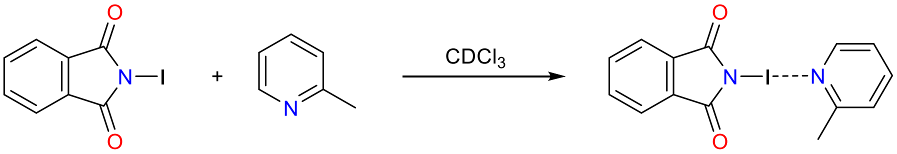

Parameter (bounds)	Optimized	Error	Initial
K (0 → ∞)	2790.09 M^{-1}	± 14.2909%	1000.00 M^{-1}

[a] See: link for BindFit v0.5 results.

6.5.9. ^{1}H Titration experiments for NIP-9[a]

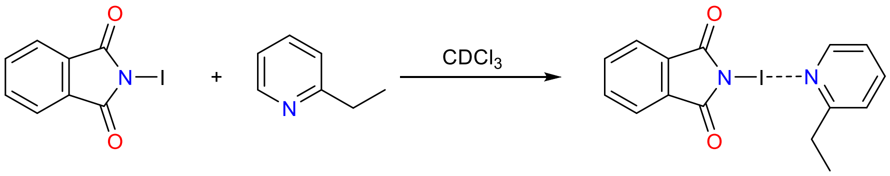

Parameter (bounds)	Optimized	Error	Initial
K (0 → ∞)	506.03 M^{-1}	± 7.4095%	100.00 M^{-1}

[a] See: link for BindFit v0.5 results.

6.5.10. ^{1}H Titration experiments for NIP-10[a]

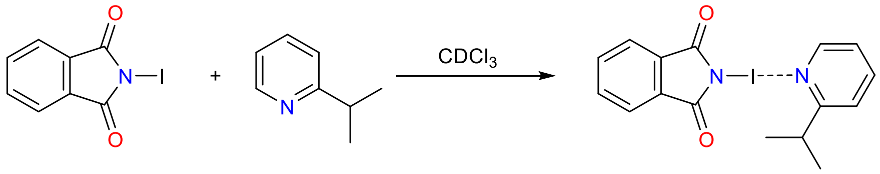

Parameter (bounds)	Optimized	Error	Initial
K (0 → ∞)	617.11 M^{-1}	± 5.5849%	100.00 M^{-1}

[a] See: link for BindFit v0.5 results.

6.5.11. ^{1}H Titration experiments for NIP-11[a]

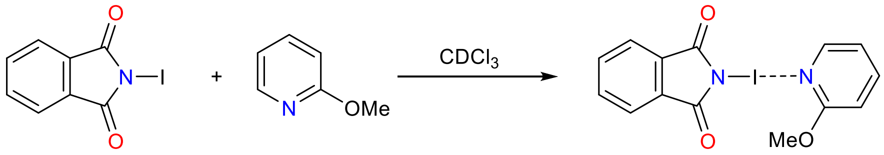

Parameter (bounds)	Optimized	Error	Initial
K (0 → ∞)	21.78 M^{-1}	± 0.47188%	10.00 M^{-1}

[a] See: link for BindFit v0.5 results.

6.5.12. [1]H Titration experiments for NIP-12[a]

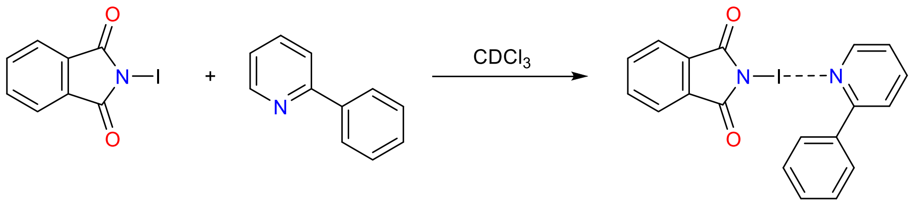

Parameter (bounds)	Optimized	Error	Initial
K (0 → ∞)	59.22 M^{-1}	± 1.6816%	10.00 M^{-1}

[a] See: link for BindFit v0.5 results.

6.5.13. [1]H Titration experiments for NIP-13[a]

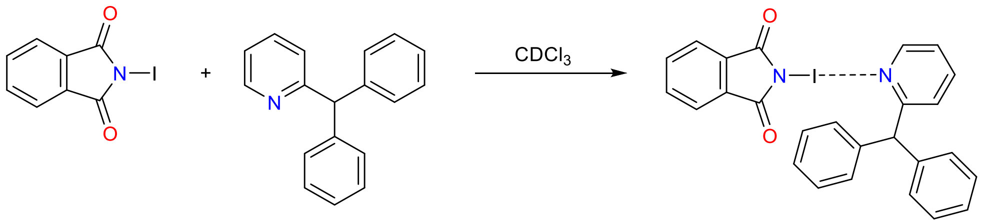

Parameter (bounds)	Optimized	Error	Initial
K (0 → ∞)	374.71 M^{-1}	± 2.9105%	100.00 M^{-1}

[a] See: link for BindFit v0.5 results.

6.5.14. [1]H Titration experiments for NIP-14[a]

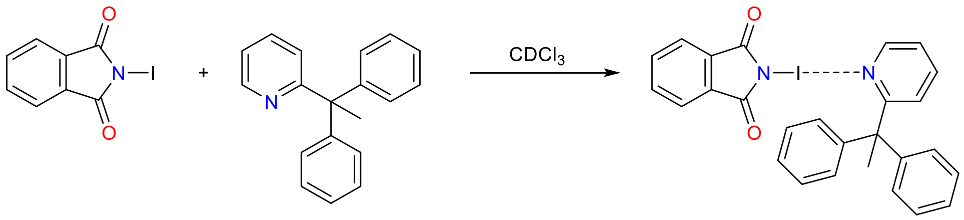

Parameter (bounds)	Optimized	Error	Initial
K (0 → ∞)	374.71 M^{-1}	± 2.9105%	100.00 M^{-1}

[a] See: link for BindFit v0.5 results.

6.5.15. [1]H Titration experiments for NIP-15[a]

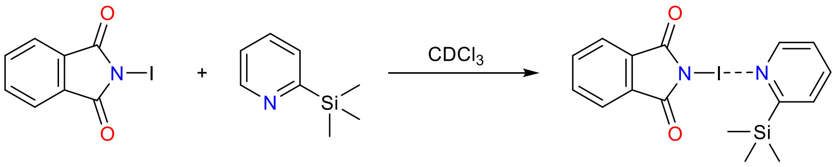

Parameter (bounds)	Optimized	Error	Initial
K (0 → ∞)	290.94 M^{-1}	± 5.4769%	10.00 M^{-1}

[a] See: link for BindFit v0.5 results.

6.6. ^{1}H Titration experiments for NISac-Z

6.6.1. ^{1}H Titration experiments for NISac-1[a]

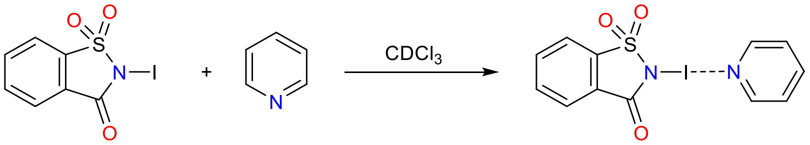

Parameter (bounds)	Optimized	Error	Initial
K (0 → ∞)	2610.15 M^{-1}	± 11.8584%	1000.00 M^{-1}

[a] See: link for BindFit v0.5 results.

6.6.2. ^{1}H Titration experiments for NISac-2[a]

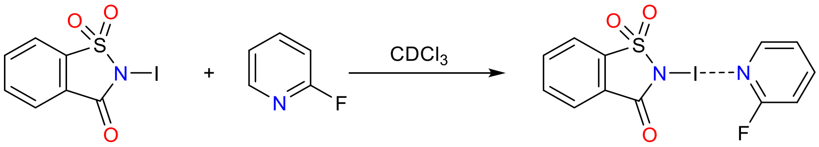

Parameter (bounds)	Optimized	Error	Initial
K (0 → ∞)	1099.59 M^{-1}	± 1.8770%	1000.00 M^{-1}

[a] See: link for BindFit v0.5 results.

6.6.3. ^{1}H Titration experiments for NISac-3[a]

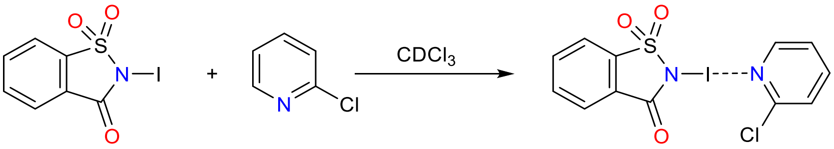

Parameter (bounds)	Optimized	Error	Initial
K (0 → ∞)	1423.90 M^{-1}	± 3.3000%	1000.00 M^{-1}

[a] See: link for BindFit v0.5 results.

6.6.4. ^{1}H Titration experiments for NISac-4[a]

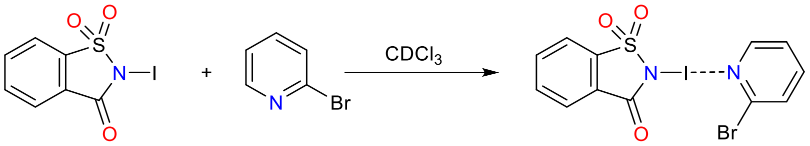

Parameter (bounds)	Optimized	Error	Initial
K (0 → ∞)	1472.40 M^{-1}	± 2.6246%	1000.00 M^{-1}

[a] See: link for BindFit v0.5 results.

6.6.5. ^{1}H Titration experiments for NISac-5[a]

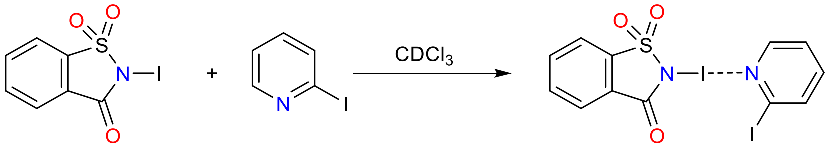

Parameter (bounds)	Optimized	Error	Initial
K (0 → ∞)	44271.80 M^{-1}	± 175.1732%	1000.00 M^{-1}

[a] See: link for BindFit v0.5 results.

6.6.6. ^{1}H Titration experiments for NISac-6[a]

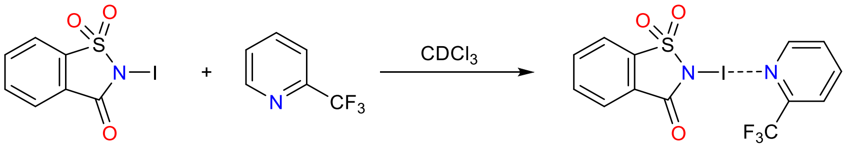

Parameter (bounds)	Optimized	Error	Initial
K (0 → ∞)	81.72 M^{-1}	± 1.4025%	100.00 M^{-1}

[a] See: link for BindFit v0.5 results.

6.6.7. ^{1}H Titration experiments for NISac-7[a]

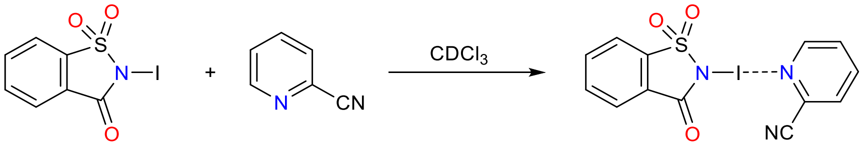

Parameter (bounds)	Optimized	Error	Initial
K (0 → ∞)	388.55 M^{-1}	± 1.7069%	100.00 M^{-1}

[a] See: link for BindFit v0.5 results.

6.6.8. ^{1}H Titration experiments for NISac-8[a]

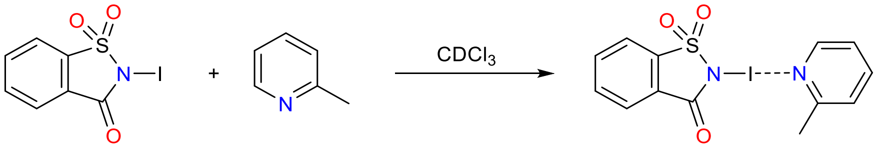

Parameter (bounds)	Optimized	Error	Initial
K (0 → ∞)	14521.51 M^{-1}	± 26.1383%	1000.00 M^{-1}

[a] See: link for BindFit v0.5 results.

6.6.9. ^{1}H Titration experiments for NISac-9[a]

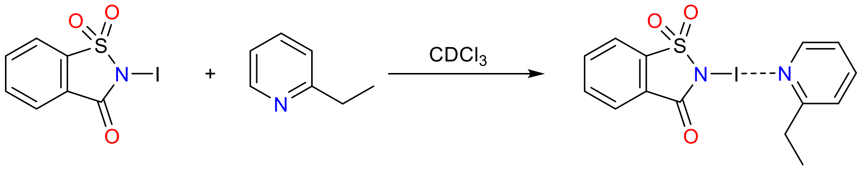

Parameter (bounds)	Optimized	Error	Initial
K (0 → ∞)	2535.49 M^{-1}	± 5.7089%	1000.00 M^{-1}

[a] See: link for BindFit v0.5 results.

6.6.10. ^{1}H Titration experiments for NISac-10[a]

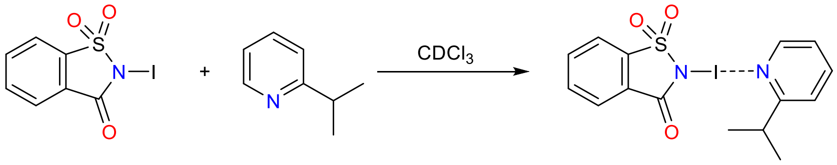

Parameter (bounds)	Optimized	Error	Initial
K (0 → ∞)	3249.64 M^{-1}	± 11.4627%	1000.00 M^{-1}

[a] See: link for BindFit v0.5 results.

6.6.11. ^{1}H Titration experiments for NISac-11[a]

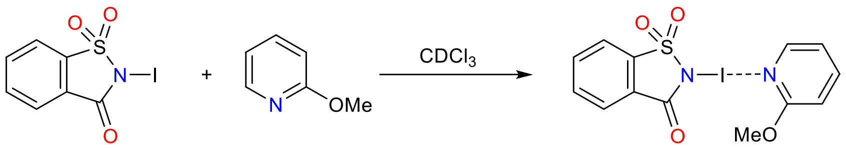

Parameter (bounds)	Optimized	Error	Initial
K (0 → ∞)	1545.32 M^{-1}	± 9.2064%	1000.00 M^{-1}

[a] See: link for BindFit v0.5 results.

6.6.12. ^{1}H Titration experiments for NISac-12[a]

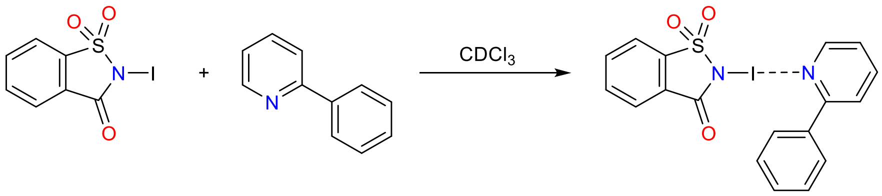

Parameter (bounds)	Optimized	Error	Initial
K (0 → ∞)	125281.27 M^{-1}	± 375.2407%	1000.00 M^{-1}

[a] See: link for BindFit v0.5 results.

6.6.13. ^{1}H Titration experiments for NISac-13[a]

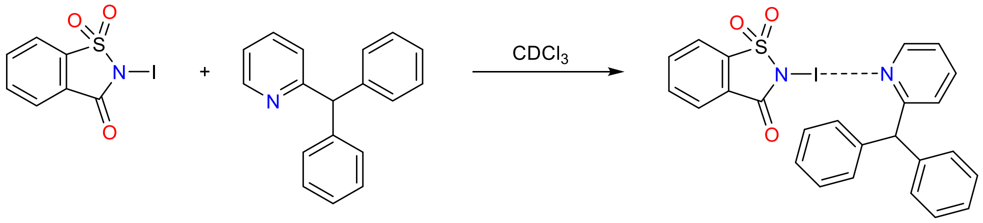

Parameter (bounds)	Optimized	Error	Initial
K (0 → ∞)	25495.07 M^{-1}	± 168.4289%	1000.00 M^{-1}

[a] See: link for BindFit v0.5 results.

6.6.14. ^{1}H Titration experiments for NISac-14[a]

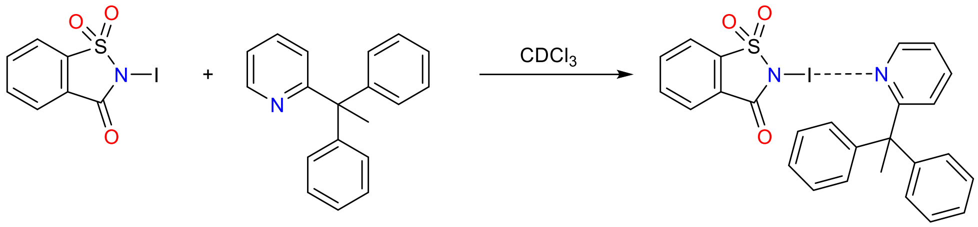

Parameter (bounds)	Optimized	Error	Initial
K (0 → ∞)	25495.07 M^{-1}	± 168.4289%	1000.00 M^{-1}

[a] See: link for BindFit v0.5 results.

6.6.15. [1]H Titration experiments for NISac-15[a]

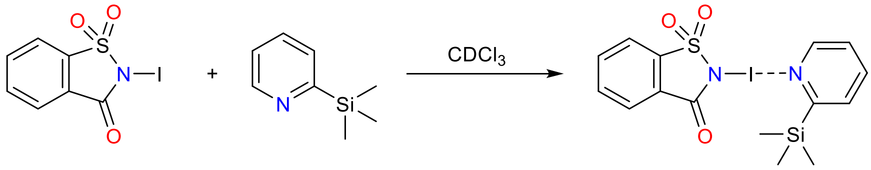

Parameter (bounds)	Optimized	Error	Initial
K (0 → ∞)	144459.18 M^{-1}	± 297.3053%	1000.00 M^{-1}

[a] See: link for BindFit v0.5 results.

6.6.16. [1]H Titration experiments for NBS-8[a,b]

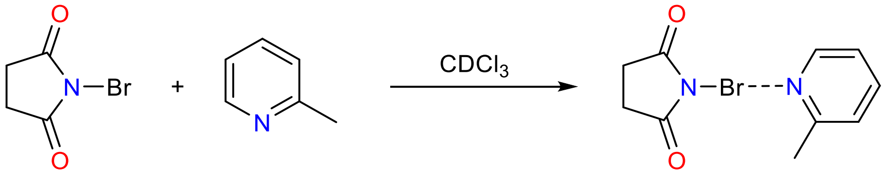

Parameter (bounds)	Optimized	Error	Initial
K (0 → ∞)	21.14 M^{-1}	± 1.7549%	100 M^{-1}

[a]For BindFit v0.5 results, see: http://app.supramolecular.org/bindfit/view/30cf3404-e228-4974-b77d-7eddbc81e331; [b]Stock solution concentration of ligand **8** is 1M

6.2.17. [1]H Titration experiments for NBP-8[a,b]

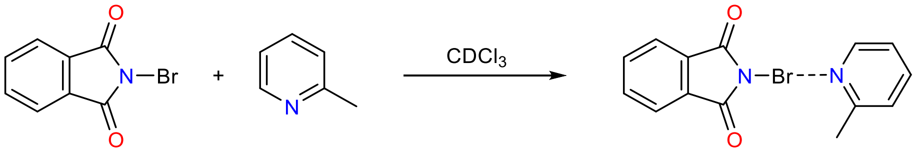

Parameter (bounds)	Optimized	Error	Initial
K (0 → ∞)	19.2 M^{-1}	± 1.0611%	100 M^{-1}

[a]For BindFit v0.5 results, see http://app.supramolecular.org/bindfit/view/013f1a8d-359f-447e-aff3-68676f8ce7fe ; [b]Stock solution concentration of ligand **8** is 1M

6.3.18. [1]H Titration experiments for NBSac-8[a,b]

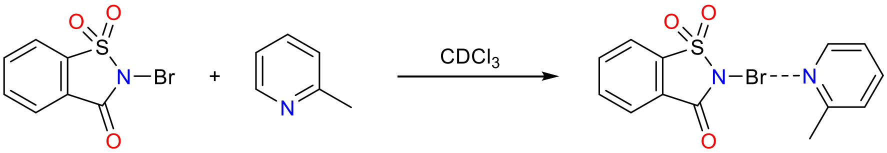

Parameter (bounds)	Optimized	Error	Initial
K (0 → ∞)	422.99 M^{-1}	± 10.6095%	100.00 M^{-1}

[a]For BindFit v0.5 results, see http://app.supramolecular.org/bindfit/view/974090c2-14da-492a-b36f-c1175cd943bc ; [b]Stock solution concentration of ligand **8** is 1M

7. ^{15}N NMR of halogenation reaction of NISac-16 in acetone-d_6

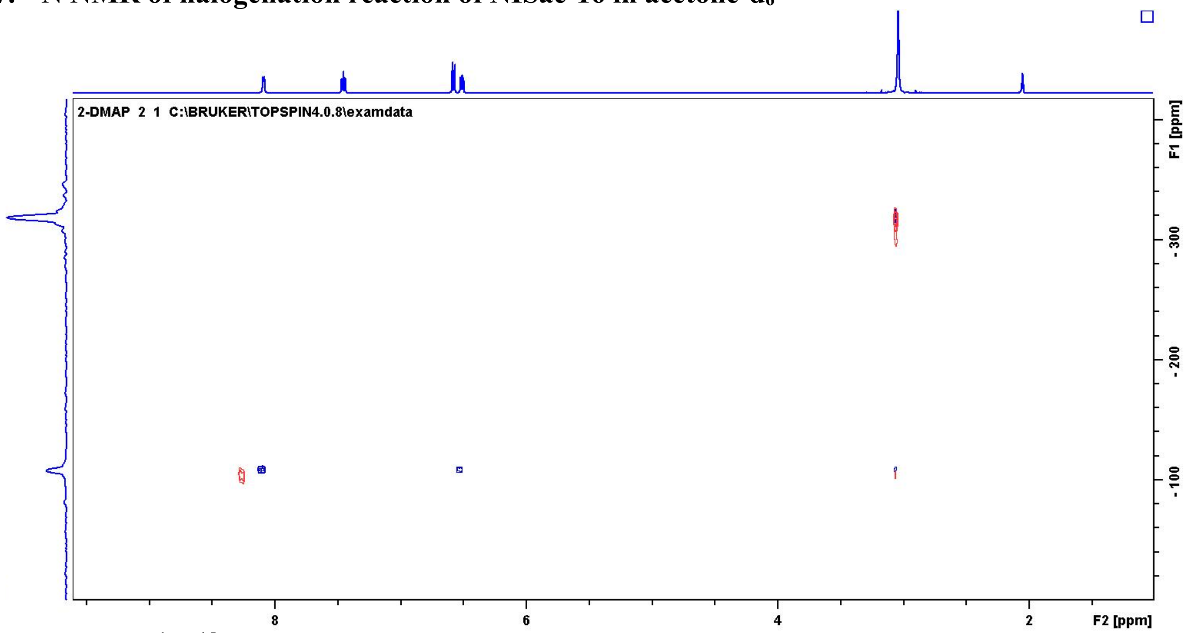

Figure S102. ^{1}H-^{15}N HMBC-NMR Spectra of **16** in blue contour and 1:1 equivalent mixture of NISac-**16** in red contour measured in acetone-d_6 at 303K.

10). References

(1) N. Lebegue, S. Gallet, N. Flouquet, P. Carato, B. Pfeiffer, P. Renard, S. Léonce, A. Pierré, P. Chavatte, P. Berthelot, *J. Med. Chem.* **2005**, *48* (23), 7363–7373.

(2) B.T. Sargent, E.J. Alexanian, *J. Am. Chem. Soc.* **2016**, *138* (24), 7520–7523.

(3) J. Berná, J. Crowley, S.M. Goldup, K.D. Hänni, A.-L. Lee, D. A. Leigh, *Angew. Chemie Int. Ed.* **2007**, *46* (30), 5709–5713.

(4) S. M. Goldup, D. A. Leigh, P. J. Lusby, R. T. McBurney, A. M. Z. Slawin, *Angew. Chemie Int. Ed.* **2008**, *47* (37), 6999–7003.

(5) *Rigaku Oxford Diffr. 2016. Version 1.171.38.41.*

(6) Z. Otwinowski, W. Minor, In *Macromolecular Crystallography Part A*; W. Charles, J. B. T.-M.Carter, Ed.; Academic Press, 1997, Vol. 276, pp 307–326.

(7) G. M. Sheldrick, *Acta Crystallogr. Sect. A* **2008**, *64* (1), 112–122.

(8) G. M. Sheldrick, *Acta Crystallogr. Sect. C* **2015**, *71* (1), 3–8.

(9) O. V. Dolomanov, L. J. Bourhis, R. J. Gildea, J. A. K. Howard, H. Puschmann, *J. Appl. Crystallogr.* **2009**, *42* (2), 339–341.

(10) (a) J. P. Perdew, K. Burke, M. Ernzerhof, *Phys. Rev. Lett.* **1996**, *77*, 3865–3868; *Phys. Rev. Lett.* **1997**, *78*, 1396; (b) J. P. Perdew, M. Ernzerhof, K. Burke, *J. Chem. Phys.* **1996**, *105*, 9982–9985; (c) C. Adamo, V. Barone, *J. Chem. Phys.* **1999**, *110*, 6158–6170.

(11) (a) F. Weigend, M. Haser, H. Patzelt, R. Ahlrichs, *Chem. Phys. Lett.* **1998**, *294*, 143–152; (b) F. Weigend, R. Ahlrichs, *Phys. Chem. Chem. Phys.*, 2005, **7**, 3297–3305.

(12) (a) S. Grimme, J. Antony, S. Ehrlich, H. Krieg, *J. Chem. Phys.* **2010**, *132*, 154104-19; (b) S. Grimme, S. Ehrlich, L. Goerigk, *J. Comput. Chem.* **2011**, *32*, 1456–1465

(13) S. F. Boys, F. Bernardi, *Mol. Phys.*, **1970**, *19*, 553–566.

(14) Gaussian 16, Revision C.01, M. J. Frisch, G. W. Trucks, H. B. Schlegel, G. E. Scuseria, M. A. Robb, J. R. Cheeseman, G. Scalmani, V. Barone, G. A. Petersson, H. Nakatsuji, X. Li, M. Caricato, A. V. Marenich, J. Bloino, B. G. Janesko, R. Gomperts, B. Mennucci, H. P. Hratchian, J. V. Ortiz, A. F. Izmaylov, J. L. Sonnenberg, D. Williams-Young, F. Ding, F. Lipparini, F. Egidi, J. Goings, B. Peng, A. Petrone, T. Henderson, D. Ranasinghe, V. G. Zakrzewski, J. Gao, N. Rega, G. Zheng, W. Liang, M. Hada, M. Ehara, K. Toyota, R. Fukuda, J. Hasegawa, M. Ishida, T. Nakajima, Y. Honda, O. Kitao, H. Nakai, T. Vreven, K. Throssell, J. A. Montgomery, Jr., J. E. Peralta, F. Ogliaro, M. J. Bearpark, J. J. Heyd, E. N. Brothers, K. N. Kudin, V. N. Staroverov, T. A. Keith, R. Kobayashi, J. Normand, K. Raghavachari, A. P. Rendell, J. C. Burant, S. S. Iyengar, J. Tomasi, M. Cossi, J. M. Millam, M. Klene, C. Adamo, R. Cammi, J. W. Ochterski, R. L. Martin, K. Morokuma, O. Farkas, J. B. Foresman, and D. J. Fox, Gaussian, Inc., Wallingford CT, 2016.
(15) E. Cancès, B. Mennucci, J. Tomasi, *J Chem Phys* **1997**, *107*, 3032–3041.
(16) B. Mennucci, E. Cancès, J. Tomasi, *J Phys Chem B* **1997**, *101*, 10506–10517.
(17) G. Scalmani, M. J. Frisch, *J Chem Phys* **2010**, *132*, 114110.
(18) J. Tomasi, B. Mennucci, R. Cammi, *Chem Rev* **2005**, *105*, 2999–3094.
(19) P. Thordarson, *Chem Soc Rev* **2011**, *40*, 1305–1323.
